# 1,4-Benzo[*b*]dithiine Derivatives Accessed via Ring Expansion of 1,3-Dithioles

**DOI:** 10.3390/molecules31183301

**Published:** 2026-09-17

**Authors:** Dawid T. Leja, Wojciech J. Depa, Maja Morawiak, Agata Bajek-Bil, Jaromir B. Lechowicz, Grażyna Groszek

**Affiliations:** 1 ICN Polfa Rzeszów S.A., Bausch Health Companies Inc., Przemysłowa 2, 35-105 Rzeszów, Poland; 2 Institute of Organic Chemistry, Polish Academy of Sciences, Kasprzaka 44/52, 01-224 Warszawa, Poland; wojciech.depa@icho.edu.pl (W.J.D.); maja.morawiak@icho.edu.pl (M.M.); 3 Faculty of Chemistry, Rzeszow University of Technology, 6 Powstańców Warszawy Ave., 35-029 Rzeszów, Poland; abajek@prz.edu.pl

**Keywords:** heterocycles, 1,3-dithiole, dithioacetal, 1,4-benzo[*b*]dithiine, ring expansion

## Abstract

Herein, we disclose an efficient route to the synthesis of 2-bromobenzo[*b*][1,4]dithiines from benzo[*d*][1,3]dithioles via ring expansion by introducing a carbon fragment of methyl or methylene groups into the heterocyclic rings promoted by bromine (51–94%). The use of a methyl substituent on an aromatic ring leads to the formation of an inseparable, equimolar mixture of regioisomers. Additionally, a 4-methoxyacetophenone derivative yielded unprecedented dimeric products, whose structure was confirmed by single-crystal X-ray crystallography. We propose a reaction mechanism, rationalizing the formation of the observed regioisomers.

## 1. Introduction

Dithioacetals are a versatile group of organosulfur compounds, typically derived from aldehydes or ketones in the presence of thiols [1]. Their most famous application in synthetic methodology is undoubtedly the Corey–Seebach reaction—the *umpolung* (polarity reversal) of a carbonyl group, where they serve as masked acyl anion equivalents [2,3,4,5]. Nonetheless, their use extends to biological applications [6,7,8,9,10] as well as their role as key intermediates in ring-expansion reactions that enable the synthesis of sulfur-heterocycles [11,12]. Among these, 1,4-dithiines constitute an important scaffold [13], and they can serve as antagonists of the human galanin hGAL-1 receptor [14], or as anthelmintics [15], while their tetroxide derivatives also show potent biological activity [16], an example being dimethipin, a commercial plant growth regulator. Synthetically, they can be deployed as a masked double bond, allowing for the preservation of the *cis* configuration of olefins via chemoselective desulfurization reactions [17]. Remarkably, they can serve as allylic alcohol anion equivalents [18] for the construction of sugar cores [19], or alternatively as allyl cation equivalents to facilitate (3 + 2) cycloaddition reactions [20], or as dienophiles in (4 + 2) cycloadditions [21], similar to their oxidized sulfone forms [22,23,24]. To date, many synthetic pathways to obtain this class of heterocycles have been developed [25,26]; however, they primarily focus on 2,3-dihydro-1,4-dithiines [27,28,29,30,31,32,33,34,35]. Their benzene-fused analogs, 1,4-benzo[*b*]dithiines, have not been explored as extensively. Examples of their synthesis include the oxidation of 1,3-benzodithioles with lead tetraacetate [36], ring expansion by Sulphur migration using toluene-*p*-sulphonic acid or trifluoroacetic acid [37], the reaction of 1,8-diketones with Lawesson’s reagent [38], reactions with in situ-generated benzdithiete [39], transformations from pentathiepins [40], the aromatization of cyclic ketones [32], reactions from phenylene tetrasulfide in the presence of a molybdenum catalyst [41], the reaction of ethynylbenziodoxolone with thiols [42], the Cu-catalyzed diarylthiolation of ynones [43], and a very recent method by Schneider utilizing 2-iodoaryl alkynyl sulfides [44]. In our effort to expand the chemistry of 1,4-dithiines, we wondered if the ring expansion reaction of dithioacetals derived from aromatic 1,2-dithiols—analogous to the prevalent ethane-1,2-dithiol-derived dithioacetals—could serve as a platform to obtain 1,4-benzo[*b*]dithiines.

## 2. Results and Discussion

### 2.1. Chemistry

**Scheme 1:**
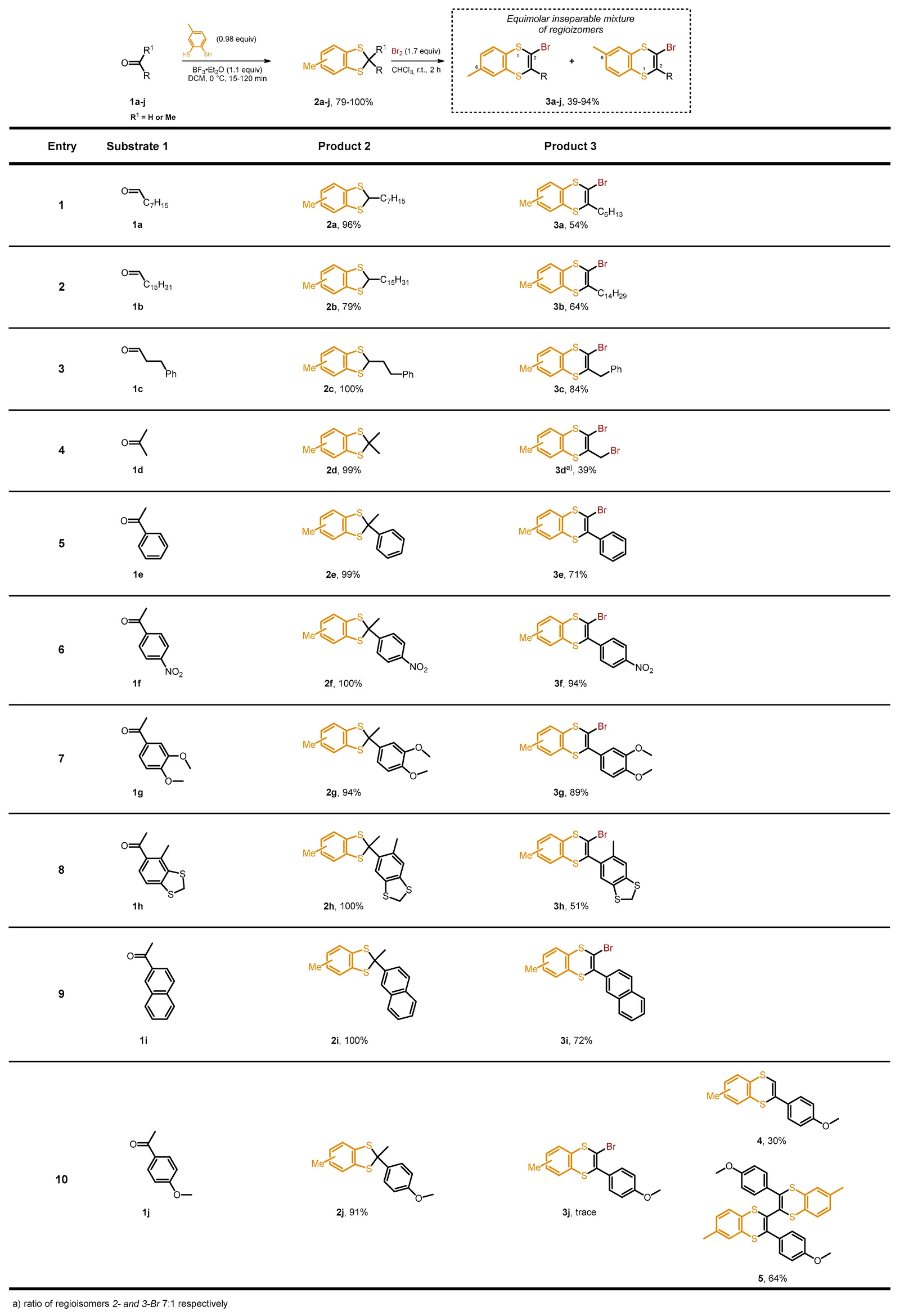
Substrate scope of the dithioacetalization reaction followed by a ring expansion reaction leading to benzo[*b*][1,4]dithiine derivatives.

We have started our investigation from dithioacetalization of aldehydes and acetophenone derivatives with 4-methylbenzene-1,2-dithiol, catalyzed by boron trifluoride etherate [45,46]. A series of dithioles **2** (**a**–**j**) were obtained in yields ranging from 79% to 100% (Scheme 1). It should be noted that, for aldehydes (**a**–**c**) and acetophenone derivatives (**e**–**j**), a new stereogenic center is formed and the resulting dithiole derivatives are obtained as racemic mixtures. Next, we have carried out the ring expansion reaction, promoted by bromine—conditions previously described by Caputo et al. [28,47]—for obtaining the derivatives of 1,4-dithiines for further broad utilization as intermediates in organic synthesis. We have isolated a series of benzo[*b*][1,4]dithiine derivatives **3** (**a**–**c**; and **e**–**i**) as an equimolar mixture of regioisomers (2-bromo and 3-bromo). In cases where it was possible, based on 2D-NMR spectra analysis, we were able to assign protons and carbon atoms to a respective regioisomer. Alkyl, heterocyclic or aryl groups, including those bearing electron-donating or electron-withdrawing substituents, were well tolerated (51–94%). On the other hand, the acetone derivative did not yield the expected product; instead, we have isolated a dibrominated product **3d** (39%), presumably formed by the substitution of one of the hydrogen atoms of the methyl group by the bromine. Interestingly, the molar ratio of **3d** regioisomers is 7:1 (estimated from the NMR spectrum), in contrast to the previous results.

**Figure 1:**
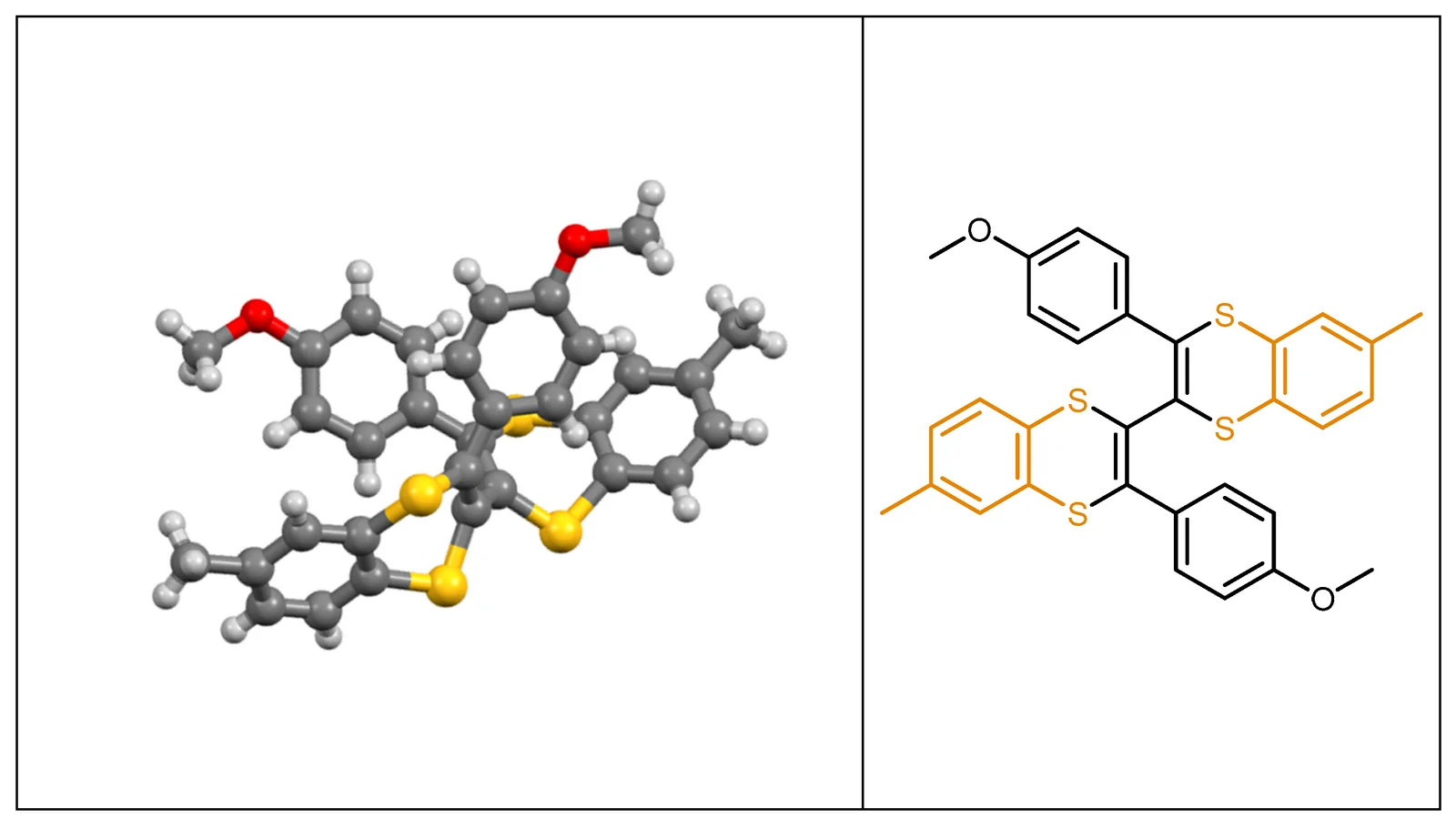
Crystal data of 5. CCDC 2571235 C_32_H_26_O_2_S_4_, *M_r_* = 570.77, triclinic, *P*-1 (No. 2), a = 9.6769(11) Å, b = 11.1216(13) Å, c = 14.2025(17) Å, *α* = 104.035(10)^°^, *β* = 93.139(9)°, *γ* = 101.083(10)^°^, *V* = 1447.0(3) Å^3^, *T* = 293.15(10) K, *Z* = 2, *Z*′ = 1, *μ*(Cu K*_α_*) = 3.232, 4940 reflections measured, 3832 unique (*R_int_* = 0.0182) which were used in all calculations. The final *wR*_2_ was 0.2184 (all data) and *R*_1_ was 0.0654 (I > 2(I)).

Unexpectedly, the 4-methoxyacetophenone derivative **2j** did not yield **3j** upon ring expansion, but instead it yielded two new products: the dithiine **4** and the dimeric product **5** (Scheme 1). Compounds **4** and **5** were obtained in a 1:2 molar ratio (per mmol of **2j** consumed), which we could separate by means of column chromatography on silica gel. As expected, **4** is a mixture of regioisomers in a 1:1 ratio. Additionally, we were able to grow a single crystal of compound **5** that was suitable for X-ray crystallography measurement, which unambiguously confirmed its structure (Figure 1).

**Scheme 2:**
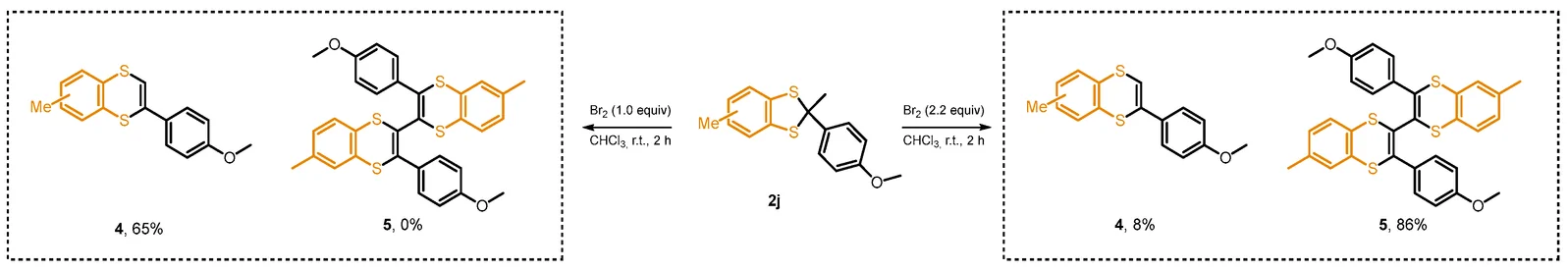
4-Methoxyacetophenone derivative ring expansion vs. dimerization.

**Scheme 3:**
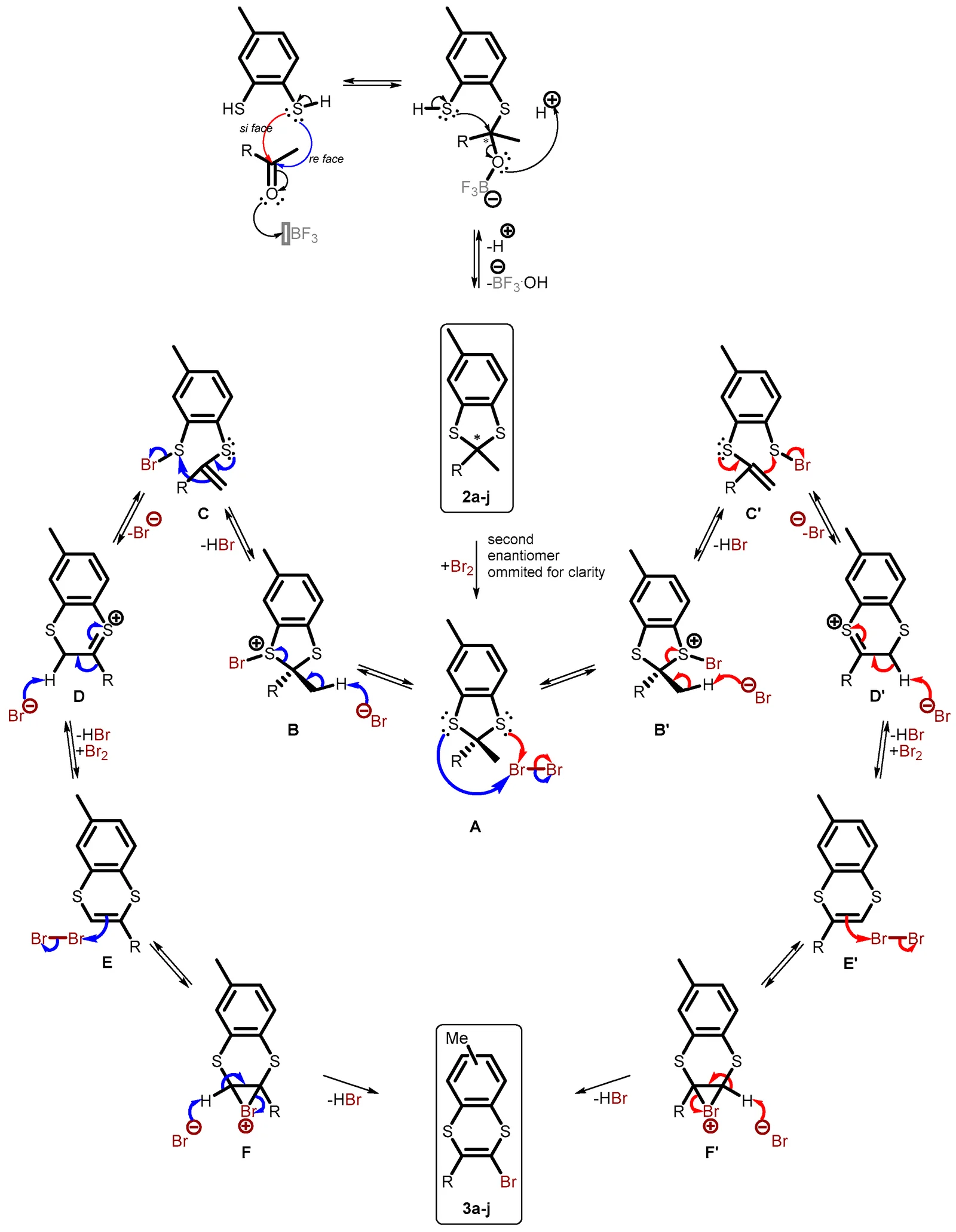
Mechanistic reaction pathway of a benzo[*d*][1,3]dithiole derivative with bromine. An asterisk (*) is the standard notation for an asymmetric or chiral carbon atom in organic chemistry.

To gain more insight into the formation of products **4** and **5,** we conducted the ring expansion reaction by altering the amount of bromine (Scheme 2). When one equivalent of bromine was used, we only obtained product **4** with a yield of 65%, and we managed to recover 28% of **2j** (estimated from a NMR spectrum). Once we increased the loading of bromine to 2.2 equivalents, only 8% of 4 remained, with dimerization to **5** in an excellent yield of 87%. Considering the influence of the amount of bromine used on the yield of product **4**, it should be assumed that, similarly to what was observed in all other cases studied, it undergoes further bromination to the intermediate bromonium cation (structure **F** in Scheme 3). It is attacked competitively by the bromide anion and by another, strongly nucleophilic—due to the influence of the *p*-methoxyphenyl substituent—molecule **4** and it undergoes quick dimerization to **5** (for the probable mechanism, see Supplementary Materials Figure S12).

**Scheme 4:**
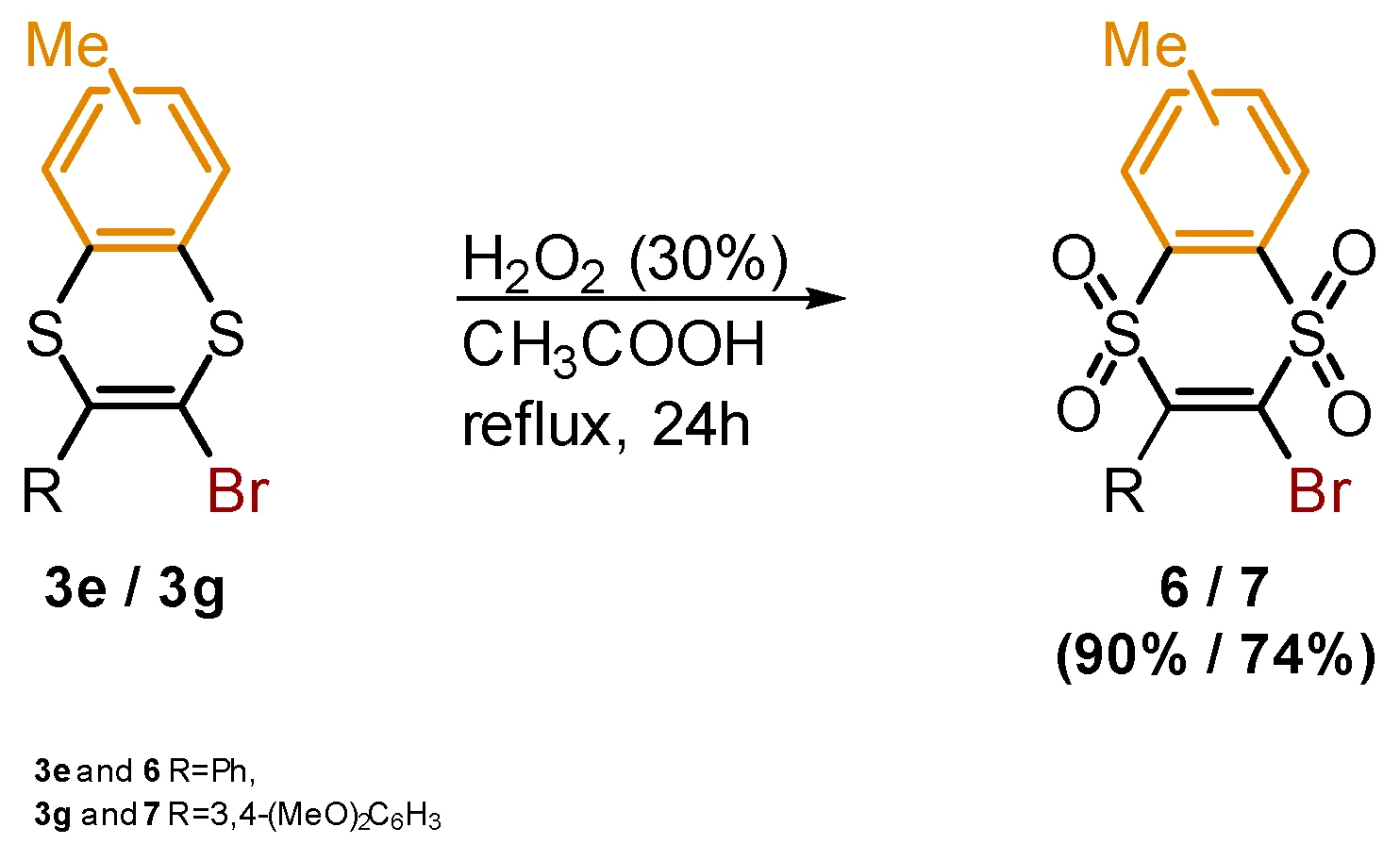
Exhaustive oxidation of dithiine **3e** and **3g** to respective tetroxides.

**Figure 2:**
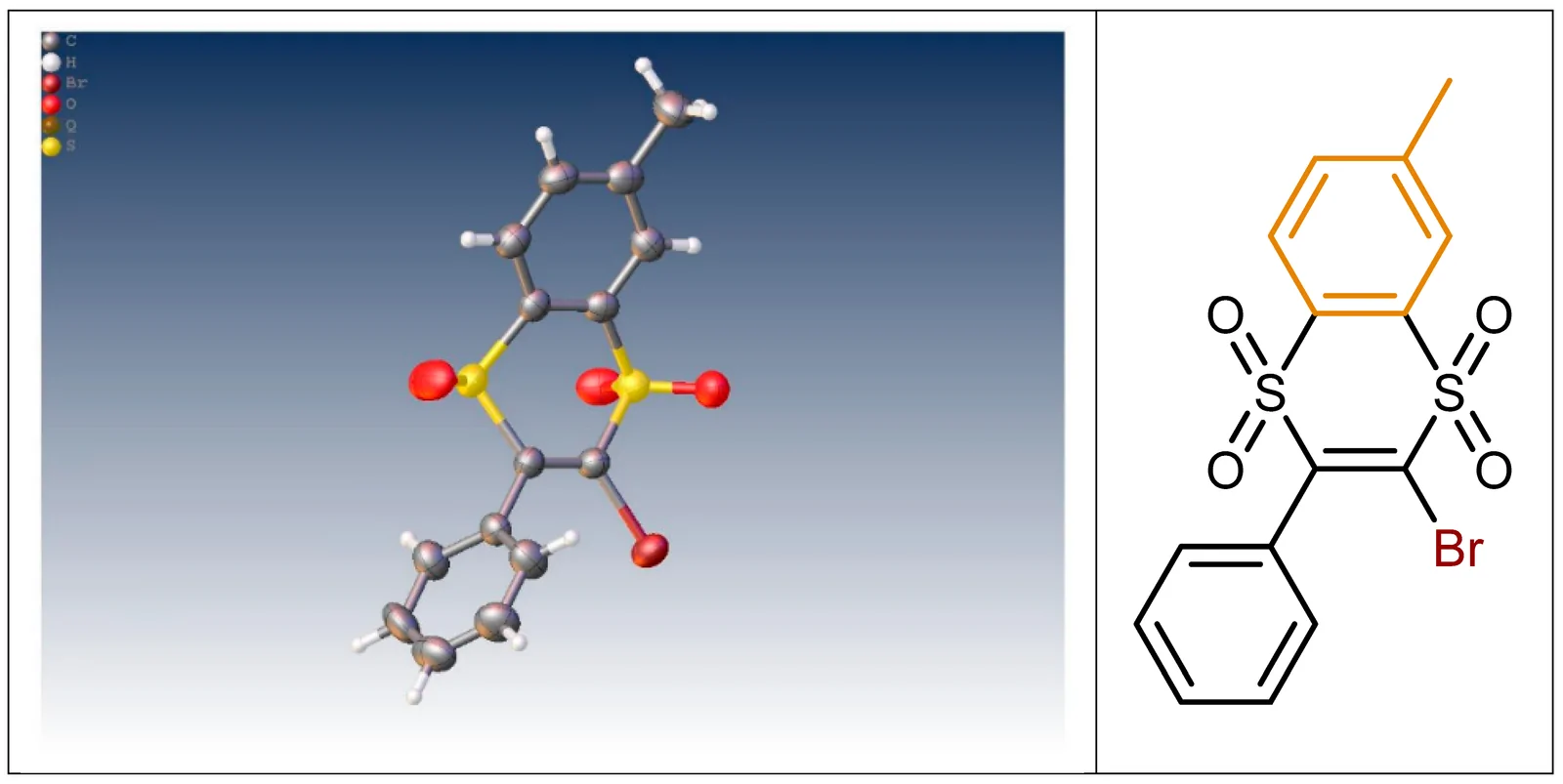
Crystal data of 6, CCDC 2571234 C_15_H_11_BrO_4_S_2_, *M_r_* = 399.27, orthorhombic, *Pca*2_1_ (No. 29), a = 7.41370(10) Å, b = 12.4876(2) Å, c = 16.3887(2) Å, *α* = *β* = *γ* = 90°, *V* = 1517.25(4) Å ^3^, *T* = 293.15(10) K, *Z* = 4, *Z*′ = 1, *μ*(Cu K*_α_*) = 6.409, 14,034 reflections measured, 2537 unique (*R_int_* = 0.0320) which were used in all calculations. The final *wR*_2_ was 0.0782 (all data) and *R*_1_ was 0.0288 (I > 2(I)).

Chromatographic methods proved futile for separating the obtained mixture of regioisomers. Thus we attempted separation by fractional crystallization using the product **3f** (see Supplementary Materials for details). We managed to enrich a single regioisomer of **3f** up to 7:1 molar ratio; however, we were unable to obtain a pure, single regioisomer. Next, we attempted the derivatization of dithiine **3e** and **3g** through exhaustive oxidation of sulfide groups to sulfone (Scheme 4). We obtained **6** with a yield of 90% (R = Ph) and tetroxide derivative **7** with a yield of 74% (R = 3,4-(MeO)_2_C_6_H_3_-). For the first compound we successfully grew a single crystal suitable for X-ray analysis, and even more fortuitously, it was of a single regioisomer 3-bromo-, (Figure 2), while all crystallization attempts for compound **7** were unsuccessful.

### 2.2. Mechanistic Considerations

The mechanism of expansion of the 1,3-dithiolane/dithiole ring is well established in the literature [11,47]. It is worth noting that 4-methylbenzene-1,2-dithiol leads to the formation of racemic dithioacetals from nonsymmetric ketones, as two such possible paths for activating the sulfur can be envisioned for dithiolane **A** (Scheme 3). They diverge into twin intermediates **B/B′,** and while the methyl group increases the electron density at the *para* position, in principle stabilizing intermediate **B′**, its effect is too weak to discriminate against the other pathway; as such, all the compounds (except the acetone derivative) were obtained as an equimolar mixture of regioisomers. Then, by the intermediates **C/C′** and **D/D′,** dithiine **E** and **E′** are formed and we managed to isolate one of such derivatives (**4**) when **2j** was used as a substrate. Otherwise, the strongly acidic environment and oxidative nature of bromine prompts the formation of bromonium cations **F** and **F′,** which are subsequently attacked by bromide (except for **2j**, where **F′** is attacked by the nucleophilic dithiin **E′**), resulting in formation of **3a**–**3i**.

Considering the reaction outliers, starting from the acetone derivative **3d**, we presume that the expected monobrominated product is initially formed, but the acidic protons at the methyl group undergo subsequent abstraction by bromide (for a reaction mechanistic proposal see Supplementary Materials Figure S5). We cannot fully explain the predominance of one of the regioisomers (1:7 molar ratio, estimated from ^1^H-NMR spectrum). Likely, the weak inductive effect of the methyl group in the *para* position of the aromatic fragment causes the divergence.

## 3. Experimental Section

General information on the substrates used (materials), analytical techniques used (methods) and the experimental procedures and characterization data for all obtained compounds are given in Supplementary Materials.

### General Procedure for the Synthesis of Dithiine Derivatives, ***3a**–**3j***

The reactions were performed on a 1–15 mmol scale, at room temperature. To a solution of the starting dithioacetal in dry chloroform (10 mL per 1 mmol of substrate), under argon atmosphere, was added, dropwise, a solution of bromine (1.7 equiv) in dry chloroform (5 mL per 1 mmol of bromine) with a constant argon flow through the reaction flask, to remove released HBr as a byproduct, which was then neutralized. The reaction mixture was stirred for the next 45 min and quenched by the addition of an aqueous solution of sodium thiosulfate (10%, 50–100 mL per 1 mmol of substrate) and solid sodium bicarbonate (5 g per 1 mmol of substrate; in case of no dissolution, an additional small amount of water was added). The aqueous phase was separated, extracted once with chloroform (same volume as water phase); organic layers were combined, washed with brine, dried over anhydrous sodium sulfate, and then filtered and concentrated under reduced pressure to obtain a crude post-reaction mixture. The product was isolated/purified by flash column chromatography (silica gel, eluent: AcOEt:*n*-hexane) to provide pure product.

## 4. Conclusions

For the first time, benzo[*b*][1,4]dithiine derivatives bearing a substituent at the benzene ring were obtained via bromine-promoted ring expansion of benzo[*d*][1,3]dithioles. The products were obtained as an equimolar and inseparable mixture of regioisomers. The use of the 4-methoxyacetophenone derivative allowed us to isolate nonbrominated dithiine **4** and its dimer **5**, whose structure was confirmed by single-crystal X-ray crystallography. Attempts to separate regioisomers were partially successful via crystallization, while, for tetroxide derivative **6**, we managed to obtain a crystal structure of a single regioisomer.

## Data Availability

The data supporting this article have been included as part of the Supplementary Materials. CIF files for **5** and **6** have been deposited in CCDC, No. 2571235 and 2571234, respectively.

## Supplementary Materials

The following supporting information can be downloaded at: https://www.mdpi.com/article/10.3390/molecules31183301/s1, Figure S1: Chromatogram GC of the compound **2a**; Figure S2: Chromatogram GC of the compound **3b**; Figure S3: Chromatogram GC of the compound **3c**; Figure S4: Chromatogram GC of the compound **3d**; Figure S5: Probable mechanism of formation **3d**; Figure S6: Chromatogram GC of the compound **3e**; Figure S7: Chromatogram GC of the compound **3f;** Figure S8: Fragmentation **3h** to ion *m*/*z* = [M-H]^+^; Figure S9: Chromatogram GC of the compound **3i**; Figure S10: Crystal Data of **5**, CCDC 2571235; Figure S11: Chromatogram GC of the compound **5**; Figure S12: Probable mechanism of formation **5**; Figure S13: Crystal Data of **6**, CCDC 2571234; Scheme S1: Synthesis of 1-(6-methylbenzo[*d*][1,3]dithiol-5-yl)ethan-1-one, **1h**; Scheme S2: Synthesis of compounds **6** and **7**. References [48,49,50,51,52,53,54] are cited in the supplementary materials.

## References

[1] Greene T.W., Wuts P.G.M. (1999). Protective Groups in Organic Synthesis.

[2] Corey E.J., Seebach D. (1965). Carbanionen Der 1,3-Dithiane, Reagentien Zur C–C-Verknüpfung Durch Nucleophile Substitution Oder Carbonyl-Addition. Angew. Chem..

[3] Corey E.J., Seebach D. (1965). Carbanions of 1,3-Dithianes. Reagents for C-C Bond Formation by Nucleophilic Displacement and Carbonyl Addition. Angew. Chem. Int. Ed. Engl..

[4] Gröbel B.-T., Seebach D. (1977). Umpolung of the Reactivity of Carbonyl Compounds Through Sulfur-Containing Reagents. Synthesis.

[5] Yus M., Nájera C., Foubelo F. (2003). The Role of 1,3-Dithianes in Natural Product Synthesis. Tetrahedron.

[6] Lhassani M., Chavignon O., Chezal J.-M., Teulade J.-C., Chapat J.-P., Snoeck R., Andrei G., Balzarini J., De Clercq E., Gueiffier A. (1999). Synthesis and Antiviral Activity of Imidazo [1,2-a]Pyridines. Eur. J. Med. Chem..

[7] Pandey S., Suryawanshi S.N., Gupta S., Srivastava V.M.L. (2005). Chemotherapy of Leishmaniasis Part II: Synthesis and Bioevaluation of Substituted Arylketene Dithioacetals as Antileishmanial Agents. Eur. J. Med. Chem..

[8] Koga H., Tsuji Y., Inoue K., Kanai K., Majima T., Kasai T., Uchida K., Yamaguchi H. (2006). In Vitro Antifungal Activity of Luliconazole against Clinical Isolates from Patients with Dermatomycoses. J. Infect. Chemother..

[9] Koga H., Nanjoh Y., Makimura K., Tsuboi R. (2009). In Vitro Antifungal Activities of Luliconazole, a New Topical Imidazole. Med. Mycol..

[10] Shao J., Bian Y., Wang Q., Mei H., Makarem A., Soloshonok V.A., Han J. (2024). Synthesis of Dithioacetals via Nucleophilic Substitution and Their Antifungal Activity Evaluation. Org. Biomol. Chem..

[11] Wood W.W., Page, P. Organosulfur Chemistry, Synthetic Aspect (1995). Trends in the Chemistry of 1,3- Dithioacetals.

[12] Abdel-Wahab B.F., Shaaban S., El-Hiti G.A. (2018). Synthesis of Sulfur-Containing Heterocycles via Ring Enlargement. Mol. Divers..

[13] Guillaumet G. (1996). 1,4-Dioxins, Oxathiins, Dithiins and Their Benzo Derivatives. Comprehensive Heterocyclic Chemistry II.

[14] Scott M.K., Ross T.M., Lee D.H.S., Wang H.-Y., Shank R.P., Wild K.D., Davis C.B., Crooke J.J., Potocki A.C., Reitz A.B. (2000). 2,3-Dihydro-Dithiin and -Dithiepine-1,1,4,4-Tetroxides: Small Molecule Non-Peptide Antagonists of the Human Galanin hGAL-1 Receptor. Bioorganic Med. Chem..

[15] Draber W., Korte F.W.A.G.K. 1,4 Dithiin-2,3,5,6-Tetracarboximides and Process for Their Preparation. US3364229A, 16 January 1968. https://patents.google.com/patent/US3364229A/en.

[16] Chee G.-L., Bhattarai B., Nash J.D., Madec K., Hasinoff B.B., Brewer A.D. (2013). Chemical Reactivity and Biological Activity of Dihydro-1,4-Dithiin Tetraoxides. Can. J. Chem..

[17] Caputo R., Palumbo G., Pedatella S. (1994). Diastereoselective Desulfurization of 5,6-Dihydro-1,4-Dithiins. Synthesis of Muscalure from *Musca domestica* L. Tetrahedron.

[18] Caputo R., Guaragna A., Palumbo G., Pedatella S. (1997). A New and Versatile Allylic Alcohol Anion and Acyl β-Anion Equivalent for Three-Carbon Homologations. J. Org. Chem..

[19] D’Alonzo D., Palumbo G., Guaragna A., Witczak Z.J., Bielski R. (2016). Multistep Transformations of BIS-Thioenol Ether-Containing Chiral Building Blocks: New Avenues in Glycochemistry. Domino and Intramolecular Rearrangement Reactions as Advanced Synthetic Methods in Glycoscience.

[20] Hullaert J., Winne J.M. (2016). (5,6-Dihydro-1,4-Dithiin-2-Yl)Methanol as a Versatile Allyl-Cation Equivalent in (3+2) Cycloaddition Reactions. Angew. Chem. Int. Ed..

[21] Gülten Ş. (2010). The Synthesis and Characterization of Solvatochromic Maleimide-fused *N*-allyl- and *N*-alkyl-substituted 1,4-dithiines and Diels–Alder Reactions with Anthracene. J. Heterocycl. Chem..

[22] Giacometti A., De Lucchi O., Dilillo F., Cossu S., Peters K., Peters E.-M., von Schnering H.G. (1994). Synthetic Equivalents to Substituted Acetylenesin Cycloaddition Reactions. Deinophilic Reactivity of 2-Methyl-, 2-Phenyl- and 2,3-Trimethylene-1,4-Benzodithiins-1,4-Tetroxides. Tetrahedron.

[23] Cossu S., Battaggia S., De Lucchi O. (1997). Barrelene, a New Convenient Synthesis. J. Org. Chem..

[24] Cossu S., De Lucchi O. (1998). 2-Chloro-1,4-benzodithiin 1,1,4,4-Tetraoxide—A Conjunctive Dienophile for the Preparation of Tetrasubstituted Polycyclic Olefins. Eur. J. Org. Chem..

[25] Fjeldskaar I.R., Rongves P., Skattebol L. (1987). A Convenient Method for the Preparation of Bicyclic Dihydro-1,4-Dioxins, Dihydro-1,4-Oxathiins, Dihydro-1,4-Dithiins and Related Compounds. Acta Chem. Scand. Ser. B.

[26] Zhang X., Zou X., Yan H. (2014). Synthesis of Boron-Fused 1,4-Dithiin via Cobalt-Mediated Disulfuration of Alkyne at the *o*-Carborane-9,12-Dithiolate Unit. Organometallics.

[27] Larsen L., Garner C.D., Joule J.A. (1989). Model Studies Related to the Cofactor of Oxomolybdoenzymes. Part 2. Quinoxalin-2-Yl-Ethane- and-Ethene-1,2-Dithiols. J. Chem. Soc. Perkin Trans. 1.

[28] Caputo R., Ferreri C., Palumbo G. (1991). Reactivity of Ethanediyl *S,S* -Acetals; 2. Synthesis of 2,3-Dihydro-1,4-Dithiins. Synthesis.

[29] Firouzabadi H., Iranpoor N., Karimi B. (1999). Tungsten Hexachloride (WCl_6_) in the Presence of Dimethylsulfoxide Promoted Facile and Efficient One-Pot Ring Expansion-Chlorination Reactions of 1,3-Dithiolanes and 1,3-Dithianes. Synlett.

[30] Firouzabadi H., Iranpoor N., Garzan A., Shaterian H.R., Ebrahimzadeh F. (2005). Facile Ring-Expansion Substitution Reactions of 1,3-Dithiolanes and 1,3-Dithianes Initiated by Electrophilic Reagents to Produce Monohalo-, -cyano-, -azido- and -thiocyanato-1,4-dithiins and -1,4-dithiepins. Eur. J. Org. Chem..

[31] Arote N., Telvekar V., Akamanchi K. (2005). Rapid Method for the Ring Expansion of 1,3-Dithiolanes and 1,3-Dithianes with *Tert* -Butyl Hypochlorite. Synlett.

[32] Murru S., Kavala V., Singh C.B., Patel B.K. (2007). A One-Pot Synthesis of 1,4-Dithiins and 1,4-Benzodithiins from Ketones Using the Recyclable Reagent 1,1′-(Ethane-1,2-Diyl)Dipyridinium Bistribromide (EDPBT). Tetrahedron Lett..

[33] Depa W.J., Buszta N., Guńka P.A., Zachara J., Bajek-Bil A., Groszek G. (2020). Chirality Transfer in the Synthesis of 2,3-Dihydro-1,4-Dithiine Derivatives. Synth. Commun..

[34] Leja D.T., Depa W.J., Guńka P.A., Zachara J., Lechowicz J.B., Groszek G. (2024). New Heterocyclic Organosulfur Compounds Derived from Dithioacetals. Synlett.

[35] Leja D.T., Guńka P.A., Florjan W., Erdem E., Groszek G. (2024). New Organosulfur Compounds Derived by Dithioketalization of Hydroxyacetone and Its Tosylate Derivative—Mechanistic Considerations. CT&B.

[36] Aromdee C., Cole E.R., Crank G. (1983). Oxidations with Lead Tetraacetate. IV. Oxidations of 1,3-Benzodithioles. Aust. J. Chem..

[37] Blatcher P., Warren S., Ncube S., Pelter A., Smith K. (1978). Ring Expansion by Sulphur Migration: Synthesis of 2-Alkylidene-1,4-benzodithians and 1,4-Benzodithiins. Tetrahedron Lett..

[38] Ozturk T. (1996). An Unusual Reaction of Lawesson’s Reagent with 1,8-Diketones: A Synthesis of Fused 1,4-Dithiins and Thiophenes. Tetrahedron Lett..

[39] Kobayashi K., Koyama E., Goto M., Noda C., Furukawa N. (2000). Dealkylation of a 1,2-Bis(Benzylthio)Benzene Derivative: Generation of Benzodithiete or Its Equivalent via a Dithia Dication. Chem. Commun..

[40] Amelichev S.A., Konstantinova L.S., Obruchnikova N.V., Rakitin O.A., Rees C.W. (2006). Synthesis of 1,4-Dithiins from Pentathiepins. Org. Lett..

[41] Harrison D.J., Fekl U. (2009). Catalytic Production of Sulfur Heterocycles (Dihydrobenzodithiins): A New Application of Ligand-Based Alkene Reactivity. Chem. Commun..

[42] Liu B., Alegre-Requena J.V., Paton R.S., Miyake G.M. (2020). Unconventional Reactivity of Ethynylbenziodoxolone Reagents and Thiols: Scope and Mechanism. Chem. Eur. J..

[43] Zhao Y.-M., Wang X., Guo Z.-Y., Li H., Zhang J.-T., Xie M.-H. (2022). Cu-Catalyzed Diarylthiolation of Ynones with Aryl Iodides and Elemental Sulfur: An Access to Tetrasubstituted (Z)-1,2-Bis(Arylthio)Alkenes and Benzo[*b*][1,4]Dithiines. J. Org. Chem..

[44] Paixao D.B., Kessler A.B., Rodembusch F.S., Stieler R., Rampon D.S., Schneider P.H. (2026). Chemodivergent Synthesis of 1,4-Benzo[*b*]Dithiins and 1,4-Benzodithiafulvenes. Org. Lett..

[45] Fieser L.F. (1954). Preparation of Ethylenethioketals. J. Am. Chem. Soc..

[46] Nakata T., Nagao S., Mori S., Oishi T. (1985). Total synthesis of (+)-pederin. 1. Stereocontrolled synthesis of (+)-benzoylpedamide. Tetrahedron Lett..

[47] Caputo R., Ferreri C., Palumbo G., Capozzi G. (1986). The reaction of ethanediyl S,S-acetals with halogens. Tetrahedron.

[48] Armarego W.L.F., Perrin D.D. (1996). Purification of Laboratory Chemicals.

[49] Ratcliffe R., Rodehorst R. (1970). Improved Procedure for Oxidations with the Chromium Trioxide-Pyridine Complex. J. Org. Chem..

[50] Nicklaus C.M., Phua P.H., Buntara T., Noel S., Heeres H.J., de Vries Johannes G. (2013). Ruthenium/1,1′-Bis(diphenylphosphino)ferrocene-Catalysed Oppenauer Oxidation of Alcohols and Lactonisation of α,ω-Diols using Methyl Isobutyl Ketone as Oxidant. Adv. Synth. Catal..

[51] Nunheim N.F., Kaiser B., Thiel W.R. (2023). Ruthenium(III) Chloride as an Efficient Catalyst for the Selective Oxidation of Fatty Alcohols to Aldehydes. ChemistrySelect.

[52] Lissel M. (1982). Herstellung und Reaktionen einiger Umpolungsreagenzien durch Phasentransfer-Katalyse. Liebigs Ann. Chem..

[53] Gleiter R., Hoffmann H., Irngartinger H., Nixdorf M. (1994). Donor-Acceptor Spiro-Compounds—Syntheses, Structures, and Electronic Properties. Chem. Berichte.

[54] Ueda N., Shimizu H., Kataoka T., Hori M. (1984). Non-stereospecific ring expansions of 5-membered heterocyclicsulfoxides. Tetrahedron Lett..

