# Supplementary material for: 1,4-Benzo[b]dithiine Derivatives Accessed via Ring Expansion of 1,3-Dithioles
Source: Molecules. 2026 Sep 17;31(18):3301. doi: 10.3390/molecules31183301 (PMC13609536; doi:10.3390/molecules31183301)
Supplement: Supplementary file 1 [file molecules-31-03301-s001.zip › molecules-4542558-supplementary.pdf]

Supporting Materials

1,4-Benzo[*b*]dithiine derivatives accessed *via* ring expansion of 1,3-dithioles

Dawid T. Leja [1,*], Wojciech J, Depa [2], Maja Morawiak [2], Agata Bajek-Bil [3], Jaromir B. Lechowicz [3,*] and Grażyna Groszek [3,*]

1. ICN Polfa Rzeszów S.A., Bausch Health Companies Inc, Przemysłowa 2, 35-105 Rzeszów, Poland,
2. Institute of Organic Chemistry, Polish Academy of Sciences, Kasprzaka 44/52, 01-224 Warszawa, Poland,
3. Faculty of Chemistry, Rzeszow University of Technology, 6 Powstańców Warszawy Ave., 35-029 Rzeszów, Poland.

[*]Corresponding author ggroszek@prz.edu.pl (G.G.); dawid.leja@bauschhealth.com (D.T.L.) jlechow@prz.edu.pl (J.B.L.)

Supplementary Content:

1. General Information ……………………………………………………………..……..S2
2. Materials ……………………………………………………………………..……....S3
3. Experimental procedures ……………………………………………….…….…....S3
   3.1 *Synthesis of **1a**, **1b**, and **1h*** …………………………………………………..…...S3
   3.2 *General procedure for dithioacetals synthesis* ……………………………………S5
   3.3 *General procedure for synthesis of dithiines **3***…………………….………….…….S9
   3.4 *Protocol for synthesis of **3j**, **4**, and **5***…………………………….…..….……....S16
   *3.5 Oxidation of **3e** and **3g*** ………………………………………………….……S18
4. References …………………………………………………………………..……S21
5. Copies of IR, ^{1}H-, ^{13}C-, COSY-, HSQC-, HMBC-NMR ……………………..S22-S139

1. General Information

Unless otherwise stated, all reactions were performed under argon in heat-gun-dried glassware. Reactions were monitored by Thin Layer Chromatography (TLC) on a Merck silica gel 60 F254 plates visualized by UV lamp at 254 nm, immersed in a solution of phosphomolybdic acid (PMA) in EtOH (20% wt) and visualized with heat. Column chromatography was performed on Merck silica gel 60 or SILICYCLE (230-400 mesh/0.040-0.063 μm). In the description of the isolation of products and their purification using the silica gel column chromatography technique, the mass of silica gel is given (g). NMR spectra were measured on a Bruker AVANCE II 500 NMR spectrometer (500 MHz for ^{1}H spectra and 125 MHz for ^{13}C spectra), residual $CHCl_3$ (δ 7.26 ppm) or TMS (δ 0.00 ppm) served as an internal standard. Chemical shifts (δ values) were quoted in parts per million (ppm), the following abbreviations were used to describe resonance signals splitting patterns: s=singlet, br s=broad singlet, d=doublet, dd= doublet of doublets, t=triplet, td=triplet doublets, m=multiplet, p=pentet, ovl=overlapped, app=apparent. Coupling constants (*J* values) were reported in Hertz (Hz). Infrared spectra were measured on a Nicolet 8700 spectrophotometer (ATR or KBr). High resolution mass spectra (HRMS) were measured on a Bruker Impact II instrument (ESI) and a Waters SYNAPT G2-S HDMS instrument using electrospray ionization (ESI) or Atmospheric Pressure Chemical Ionization (APCI). Melting points were obtained on a MEL-TEMP® apparatus and are uncorrected. Single-crystal X-ray diffraction were obtained on a Rigaku XtaLab Synergy-S equipped with a micro-focus X-ray source (MoKα/CuKα radiation). In the description of the isolation of products and their purification using the silica gel column chromatography technique, the mass of silica gel is given (g). All liquid physical mixtures are given as mixtures by volume (v/v). Despite rigorous drying under vacuum, in ^{1}H NMR spectra of some samples a δ 5.30 ppm resonance signal of residual dichloromethane (DCM) is present. Gas chromatography analysis were performed on a Shimadzu GC-2010 Plus with helium as the carrier gas and a Zebron ZB 5MSi column, coupled to a flame ionisation detector (GC-FID).

All GCs were recorded with the following parameters:

Pressure: 121.8 kPA; Total flow: 30.3 mL/min; Column flow: 1.30 mL/min; Linear velocity: 33.1 cm/s; Purge flow: 3.0 mL/min; Split ratio: 20.0; Total program time: 22.39 min.

With the following method (print screen below):

Rate [°C/min]	Temperature [°C]	Hold time [min]
-	100	1.00
40	180	1.50
40	260	1.50
45	300	1.00
50	325	12.00

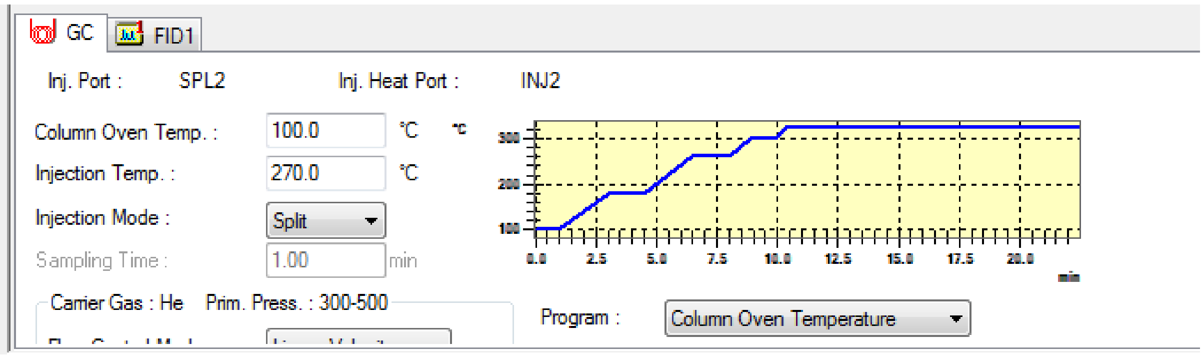

2. Materials

Commercially available reagents were used without purification. The solvents used in the reactions were dried according to the literature [1]. **Boron trifluoride etherate (**for synthesis), was purchased from Sigma-Aldrich. **Bromine** was dried over conc. H_2SO_4, **4-methylbenzene-1,2-dithiol** was available in the Faculty of Chemistry reagent warehouse.

Aldehyde **1c** and methyl ketones **1d**, **1e**, **1f**, **1g**, **1i** and **1j** were available commercially.

3. Experimental procedures

*3.1. Synthesis of **1a**, **1b**, and **1h***

The oxidation of octan-1-ol or hexadecan-1-ol to the corresponding aldehydes were carried out with the Collins reagent according to the procedure described in the literature [2] in scale 20 and 10 mmol respectively.

Representative synthesis. To a solution of pyridine (9.61 g; 9.8 mL; 121.6 mmol) in dry DCM (150 mL) was added CrO_3 (6.01 g; 60.0 mmol), and the resulting mixture was stirred for 30 min and then hexadecan-1-ol (10.0 mmol) was added in one portion. After additional 30 min of stirring occurring precipitate was filtered out, clear reaction mixture was washed in following order with: 5% water solution of NaOH (100 mL); 5% water solution of HCl (2x100 mL); saturated water solution of $NaHCO_3$ (100 mL); brine (150 mL). Organic layer was then dried over $MgSO_4$, concentrated under reduced pressure and the product was isolated by column chromatography (33-fold silica gel excess, *n*-hexane:AcOEt, 49:1).

Spectral data of **octanal**, **1a**, (1.48 g, 11.54 mmol, yield 56% as colorless liquid):

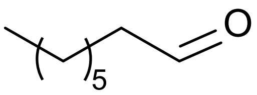

^{1}H NMR ($CDCl_3$, 500 MHz): δ=9.77 (d, *J* 0.8, 1H); 2.42 (td ovl, J_1 7.6, J_2 0.8; 2H); 1.63 (p, *J* 7.1; 2H); 1.32-1.28 (m, 8H); 0.88 (t, *J* 6.6, 3H).

^{13}C-NMR ($CDCl_3$, 125 MHz): δ=203.0; 43.9; 31.6; 29.1; 29.0; 22.6; 22.1; 14.1.

Spectral data are consistent with the literature [3].

Spectral data of **hexadecanal**, **1b**, (1.70 g, 7.07 mmol, yield 71% as colorless oil):

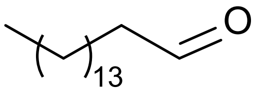

^{1}H-NMR ($CDCl_3$, 500 MHz): δ=9.76 (s, 1H); 2.42 (t app, *J* 7.3, 2H); 1.62 (p, *J* 7.1, 2H); 1.30-1.26 (m, 24H); 0.88 (t, *J* 6.7, 3H).
^{13}C-NMR ($CDCl_3$, 125 MHz): δ=203.0; 43.9; 31.9; 29.7; 29.7; 29.7; 29.6; 29.4; 29.4; 29.2; 22.7; 22.1; 14.1.

Spectral data are consistent with the literature [4].

Synthesis of ***1-(6-methylbenzo[d][1,3]dithiol-5-yl)ethan-1-one, 1h***, (Scheme 1.).

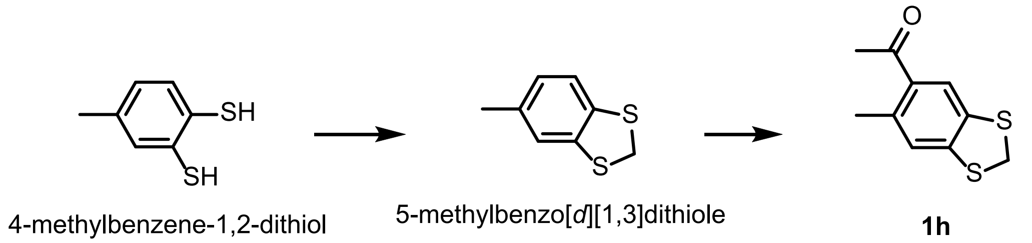

Scheme S1. Synthesis of 1-(6-methylbenzo[*d*][1,3]dithiol-5-yl)ethan-1-one, **1h**.

To a suspension of sodium hydroxide (12.00 g; 0.30 mol) and dibromomethane (26.07 g; 10.53 mL; 0.15 mol) in dry DMSO (50 mL) a solution of **4-methylbenzene-1,2-dithiol** (7.81 g; 0.05 mol) in dry DMSO (50 mL) was added dropwise at 100 °C and the resulting mixture was stirred for 30 min. Then it was poured into ice water (750 mL) and extracted with DCM (2x400 mL), combined organic layers were, washed with brine (100 mL), dried over anhydrous Na_2SO_4, filtered and volatiles were removed under reduced pressure. Raw product (11.50 g) was purified by column chromatography on silica gel (20-fold silica gel excess, *n*-hexane:AcOEt, 24:1) yielding:
5-methylbenzo[*d*][1,3]dithiole (4.62 g, 27.45 mmol, yield 54% as a yellowish oil).

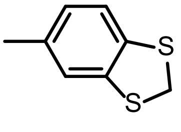

^{1}H NMR (500 MHz, $CDCl_3$): δ=7.09 (d, *J* 7.9, 1H); 7.04 (s, 1H); 6.81 (d, *J* 7.9, 1H); 4.46 (s, 2H); 2.25 (s, 3H).
^{13}C NMR (125 MHz, $CDCl_3$): δ=138.3; 135.6; 134.7; 126.4; 123.1; 122.0; 36.2; 20.9.
Spectral data of the compound are consistent with literature data [5].

To a solution of **5-methylbenzo[*d*][1,3]dithiol** (1.26 g; 7.5 mmol) in TFA (10 mL) an acetic anhydride (1.53g; 15 mmol) was added dropwise and the resulting mixture was stirred for 3

days, then it was quenched with $NaHCO_{3\,(aq)(sat)}$ (150 mL), extracted with AcOEt (2x100 mL). The combined organic layers were washed with brine (50 mL), dried over anhydrous Na_2SO_4, filtered and volatiles were removed under reduced pressure. Raw product (1.45 g) was purified by column chromatography (75-fold silica gel excess, *n*-hexane:AcOEt, 24:1) yielding:

1-(6-methylbenzo[*d*][1,3]dithiol-5-yl)ethan-1-one, **1h**, (0.88 g, 4.18 mmol, yield 56% as a yellow oil).

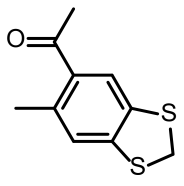

IR (ATR), (cm^{-1}): $\tilde{\nu}$=2968; 2994; 2919; 1668; 1576; 1530 1443; 1351; 1299; 1234; 1135; 1033; 957; 875; 864; 694.
^{1}H NMR (500 MHz, $CDCl_3$): δ=7.54 (s, 1H); 7.08 (s, 1H); 4.53 (s, 2H); 2.52 (s, 3H); 2.45 (s, 3H).
^{13}C NMR (125 MHz, $CDCl_3$): δ=199.7; 143.3; 137.0; 135.6; 134.8; 125.4; 123.0; 36.1; 29.3; 21.6.
HRMS (ESI^+) for $C_{10}H_{11}OS_2$ $[M+H]^+$: calcd: 211.0251; found: 211.0249.

3.2 General procedure for dithioacetals synthesis

To a solution of a carbonyl compound and **4-methylbenzene-1,2-dithiol** (0.98 equiv) in dry DCM (10 mL per 1 mmol of carbonyl compound) a $BF_3{\cdot}Et_2O$ (1.1 equiv per carbonyl compound) was added dropwise at 0 °C, and then the resulting mixture was allowed to warm up to room temperature. After completion of reaction (progress was monitored with TLC) a saturated water solution of $NaHCO_3$ (2 vol. per 1 vol. of reaction mixture) was added, followed by and DCM (1 vol. per 1 vol. of reaction mixture). Layers were separated and water phase extracted with DCM (half vol.). Combined organic layers were dried over anhydrous Na_2SO_4, filtered and volatiles were removed under reduced pressure, and the products were purified by column chromatography on silica gel.

Exemplary method of obtaining **2e**

To a solution of acetophenone (2.43 mL; 20.4 mmol) and **4-methylbenzene-1,2-dithiol** (3.13 g; 20.0 mmol; 0.98 equiv) in dry DCM (100mL) a $BF_3{\cdot}Et_2O$ (2.96 mL; 24.0 mmol; 1.2 equiv) was added dropwise at 0°C, and then the resulting mixture was allowed to warm up to room temperature. After completion of reaction (progress was monitored with TLC, 120 min) a saturated water solution of $NaHCO_3$ (200 mL) was added followed by DCM (100 mL). Layers were separated, and water phase was extracted with DCM (1x100 mL), combined organic layers were dried over anhydrous Na_2SO_4, filtered and volatiles were removed under reduced pressure.

The raw product (6.54 g, yellow oil) was purified by column chromatography on silica gel (30-fold silica gel excess, *n*-hexane:AcOEt, 39:1) yielding:

2,5-dimethyl-2-phenylbenzo[*d*][1,3]dithiole, **2e**, (5.10 g, 19.74 mmol, yield 99% as a yellow oil)

Spectral data of **2-heptyl-5-methylbenzo[*d*][1,3]dithiole**, **2a**, (0.90 g, 3.38 mmol, yield 96% as a colorless oil):

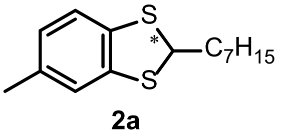

2a

IR (ATR), (cm^{-1}): $\tilde{\nu}$=2953; 2922; 2869; 2853; 1710; 1586; 1457; 1378; 1263; 1140; 1124; 1034; 865; 801; 723; 695.

^{1}H NMR (500 MHz, $CDCl_3$): δ=7.08 (d, *J* 7.8, 1H); 7.04 (s, 1H); 6.82 (d, *J* 7.8, 1H), 4.83 (t, *J* 7.2, 1H); 2.26 (s, 3H); 1.95-1.90 (m, 2H); 1,45-1.42 (m, 2H); 1.27-1.26 (m, 8H); 0.88 (t, *J* 6.7, 3H).

^{13}C NMR (125 MHz, $CDCl_3$): δ=137.5; 135.4; 134.0; 126.3; 123.2; 122.2; 55.0; 39.0; 31.7; 29.1; 29.0; 27.3; 22.6; 20.9; 14.1.

HRMS (ESI^+) for $C_{15}H_{22}S_2Na$ $[M+Na]^+$: calcd: 289.1060; found: 289.1056.

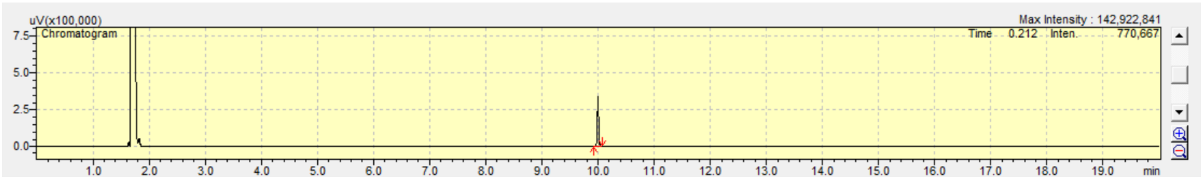

Figure S1. Chromatogram GC of the compound **2a**.

Spectral data of **2-pentadecyl-5-methylbenzo[*d*][1,3]dithiole**, **2b**, (1.01 g, 2.67 mmol, yield 79% as a waxy colorless solid):

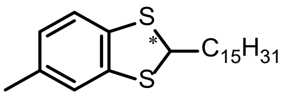

2b

IR (ATR), (cm^{-1}): $\tilde{\nu}$=2954; 2913; 2872; 2849; 1471; 1459; 1436; 1382; 1264; 1141; 1125; 872; 868; 793; 717; 697.

^{1}H NMR (500 MHz, $CDCl_3$): δ=7.08 (d, *J* 7.9, 1H); 7.03 (s, 1H); 6.82 (d, *J* 7.9, 1H), 4.83 (t, *J* 7.2, 1H); 2.26 (s, 3H); 1.94-1.90 (m, 2H); 1,45-1.42 (m, 2H); 1.25 (br s, 24H); 0.88 (t, *J* 6.8, 3H).

^{13}C NMR (125 MHz, $CDCl_3$): δ=137.5; 135.4; 134.0; 126.3; 123.1; 122.2; 55.0; 39.0; 31.9; 29.7; 29.7; 29.7; 29.6; 29.5; 29.4; 29.4; 29.0; 27.3; 22.7; 20.9; 14.1.

mp.: 32-34°C (*n*-hexane).

HRMS (ESI^+) for $C_{23}H_{38}S_2Na$ $[M+Na]^+$: calcd: 401.2312; found: 401.2302.

Spectral data of **5-methyl-2-phenethylbenzo[*d*][1,3]dithiole**, **2c**, (3.92 g, 14.38 mmol, quantitative as a colorless oil):

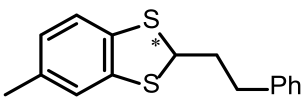

2c

IR (ATR), (cm^{-1}): $\tilde{\nu}$=3060; 3024; 2917; 2854; 1602; 1584; 1496; 1457; 1263; 1140; 1124; 865; 803; 748; 695.
^{1}H NMR (500 MHz, $CDCl_3$): δ=7.33-7.30 (m, 2H); 7.24-7.20 (m, 3H); 7.14 (d, *J* 7.9, 1H); 7.10 (s, 1H); 6.87 (d, *J* 7.9, 1H), 4.77 (t, *J* 7.2, 1H); 2.83 (t, *J* 7.5, 2H); 2.30 (s, 3H); 2.27 (dd, J_1 7.5 J_2 7.2, 2H).

^{13}C NMR (125 MHz, $CDCl_3$): δ=140.4; 137.3; 135.4; 133.7; 128.5; 128.5; 126.3; 126.1; 123.2; 122.2; 53.8; 40.6; 32.9; 20.9.
HRMS (ESI$^+$) for $C_{16}H_{16}S_2Na$ $[M+Na]^+$: calcd: 295.0591; found: 295.0586.

Spectral data of **2,2,5-trimethylbenzo[*d*][1,3]dithiole**, **2d**, (1.84 g, 9.37 mmol, yield 99% as a colorless oil):

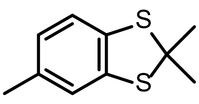

2d

^{1}H NMR (500 MHz, $CDCl_3$): δ=7.06 (d, *J* 7.9, 1H); 7.02 (s, 1H); 6.82 (d, *J* 7.9, 1H), 2.26 (s, 3H), 1.89 (s, 6H).
^{13}C NMR (125 MHz, $CDCl_3$): δ=138.5; 135.4; 135.0; 126.3; 123.4; 122.5; 64.9; 31.4; 20.9.
Spectral data are consistent with the literature [6].

Spectral data of **2,5-dimethyl-2-phenylbenzo[*d*][1,3]dithiole**, **2e**, (5.10 g, 19.74 mmol, yield 99% as a colorless oil):

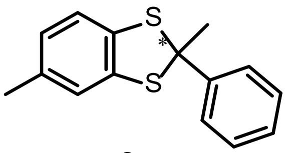

2e

^{1}H NMR (500 MHz, $CDCl_3$): δ=7.82 (d, *J* 7.8, 2H); 7.36-7.33 (m, 2H); 7.30-7.27 (m, 1H), 7.09 (d, *J* 7.9, 1H); 7.04 (s, 1H); 6.86 (d, *J* 7.9, 1H); 2.28 (s, 3H); 2.28 (s, 3H).
^{13}C NMR (125 MHz, $CDCl_3$): δ=142.1; 138.0; 135.8; 134.5; 128.3; 128.0; 126.8; 126.6; 123.2; 122.2; 70.6; 32.4; 21.0.
Compound was described in the literature [7].

Spectral data of **2,5-dimethyl-2-(4-nitrophenyl)benzo[*d*][1,3]dithiole**, **2f**, (2.91 g, 9.60 mmol, quantitative as a yellow crystals):

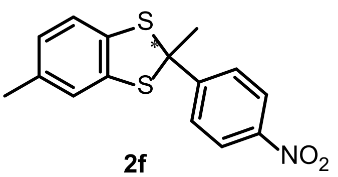

2f

IR (ATR), (cm^{-1}): $\tilde{\nu}$=3103; 3077; 2994; 2914; 1602; 1593; 1508; 1487; 1455; 1347; 1335; 1072; 851; 816; 810; 755; 695.
^{1}H NMR (500 MHz, $CDCl_3$): δ=8.16 (d, *J* 8.8, 2H); 7.98 (d, *J* 8.8, 2H); 7.08 (d, *J* 7.9, 1H); 7.03 (s, 1H); 6.87 (d, *J* 7.9, 1H); 2.28 (s, 3H); 2.27 (s, 3H).
^{13}C NMR (125 MHz, $CDCl_3$): δ=150.7; 147.3; 137.4; 136.3; 134.0; 127.8; 127.1; 123.4; 123.2; 122.3; 69.5; 31.1; 21.0.
mp.: 106-107°C (CH_2Cl_2).
HRMS (ESI$^+$) for $C_{15}H_{13}NO_2S_2Na$ $[M+Na]^+$: calcd: 326.0285; found: 326.0277.

Spectral data of **2-(3,4-dimethoxyphenyl)-2,5-dimethylbenzo[*d*][1,3]dithiole**, **2g**, (1.65 g, 5.18 mmol, yield 94% as a colorless crystal):

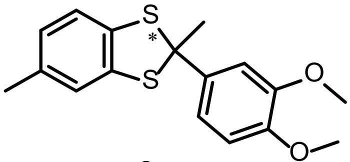

2g

IR (ATR), (cm^{-1}): $\tilde{\nu}$=2967; 2950; 2930; 2838; 1586; 1509; 1455; 1442; 1410; 1333; 1257; 1241; 1169; 1149; 1034; 1022; 910; 861; 801; 771; 694.
^{1}H NMR (500 MHz, $CDCl_3$): δ=7.55 (d, *J* 2.1, 1H); 7.28 (dd, J_1 8.5, J_2 2.1, 1H); 7.08 (d, *J* 7.9, 1H); 7.04 (s, 1H); 6.87 (d, *J* 7.9, 1H); 6.80 (d, *J* 8.5, 1H); 3.90 (s, 3H); 3.88 (s, 3H); 2.29 (s, 3H); 2.28 (s, 3H).
^{13}C NMR (125 MHz, $CDCl_3$): δ=148.9; 148.4; 138.0; 135.8; 134.6; 133.7; 126.6; 123.1; 122.2; 119.1; 111.1; 110.3; 70.5; 56.0; 55.9; 32.2; 21.0.
mp.: 87-89°C (CH_2Cl_2).
HRMS (ESI^+) for $C_{17}H_{19}O_2S_2$ $[M+H]^+$: calcd: 319.0826; found: 319.0824.

Spectral data of **2,5,6'-trimethy-2,5'-bibenzo[*d*][1,3]dithiole**, **2h**, (0.52 g, 1.50 mmol, quantitative yield as a yellow oil):

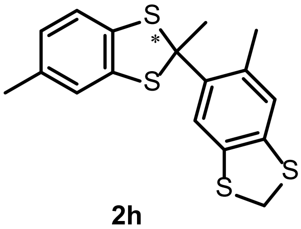

2h

IR (ATR), (cm^{-1}): $\tilde{\nu}$=2967; 2916; 2862; 1456; 1372; 1349; 1262; 1208; 1141; 1123; 1065; 1032; 865; 803; 735; 696.
^{1}H NMR (500 MHz, $CDCl_3$): δ=7.56 (s, 1H); 7.08 (d, *J* 7.9, 1H); 7.04 (s, 1H); 7.03 (s, 1H); 6.85 (d, *J* 7.9, 1H); 4.47 (s, 2H); 2.62 (s, 3H); 2.27 (s, 3H); 2.25 (s, 3H).
^{13}C NMR (125 MHz, $CDCl_3$): δ=138.4; 137.7; 136.8; 135.8; 135.6; 135.0; 134.2; 126.5; 126.3; 123.1; 122.2; 121.4; 70.6; 36.3; 32.8; 22.9; 21.0.
HRMS (ESI^+) for $C_{17}H_{17}S_4$ $[M+H]^+$: calcd: 349.0208; found: 349.0213.

Spectral data of **2,5-dimethyl-2-(naphthalen-2-yl)benzo[*d*][1,3]dithiole**, **2i**, (2.96 g, 9.60 mmol, quantitative as a colorless oil):

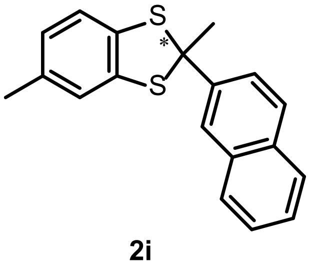

2i

IR (ATR), (cm^{-1}): $\tilde{\nu}$=3050; 2975; 2924; 1594; 1504; 1455; 1430; 1372; 1276; 1188; 1123; 1088; 1061; 953; 895; 879; 862; 825; 806; 770; 752; 694.
^{1}H NMR (500 MHz, $CDCl_3$): δ=8.19 (br s, 1H); 8.10 (dd, J_1 8.7, J_2 1.6, 1H); 7.88-7.83 (m, 3H); 7.53-7.50 (m, 2H); 7.14 (d, *J* 7.9, 1H); 7.09 (s, 1H); 6.88 (d, *J* 7.9, 1H), 2.43 (s, 3H); 2.31 (s, 3H).
^{13}C NMR (125 MHz, $CDCl_3$): δ=139.3; 138.0; 135.8; 134.5; 132.8; 132.7; 128.3; 127.9; 127.5; 126.6; 126.5; 126.4; 125.4; 123.1; 122.2; 70.7; 32.0; 21.0.
mp.: 125-127°C (acetone).
HRMS (ESI^+) for $C_{19}H_{16}S_2Na$ $[M+Na]^+$: calcd: 331.0586; found: 331.0582.

Spectral data of **2-(4-methoxyphenyl)-2,5-dimethylbenzo[*d*][1,3]dithiole**, **2j**, (2.74 g, 9.50 mmol, yield 91% as a colorless crystal):

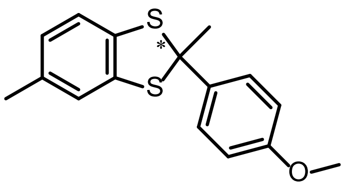

2j

IR (ATR), (cm^{-1}): $\tilde{\nu}$=2969; 2921; 2840; 1604; 1578; 1510; 1454; 1441; 1298; 1249; 1182; 1118; 1073; 1027; 839; 823; 809; 799; 746; 693.

^{1}H NMR (500 MHz, $CDCl_3$): δ=7.81 (d, *J* 8.8, 2H); 7.10 (d, *J* 7.9, 1H); 7.05 (s, 1H); 6.89 (d, *J* 8.8, 2H); 6.88 (d, *J* 7.9, 1H); 3.82 (s, 3H); 2.31 (s, 3H); 2.30 (s, 3H).

^{13}C NMR (125 MHz, $CDCl_3$): δ=159.2; 138.1; 135.7; 134.6; 133.6; 128.3; 126.6; 123.1; 122.2; 113.4; 70.2; 55.3; 32.3; 21.0.

mp.: 74-75°C (acetone).

HRMS (ESI$^+$) for $C_{16}H_{16}OS_2Na$ $[M+Na]^+$: calcd: 311.0540; found: 311.0534.

*3.3 General procedure for synthesis of dithiines **3***

The reactions were performed on a 1-15 mmol scale at room temperature. To a solution of the starting dithioacetal in dry chloroform (10 mL per 1 mmol of substrate), under argon atmosphere, was added, dropwise, a solution of bromine (1.7 equiv) in dry chloroform (5 mL per 1 mmol of bromine) with a constant argon flow through the reaction flask, to remove released HBr as a byproduct, which was then neutralized in a scrubber. Then an equeous solution of sodium thiosulfate (10%, 50 - 100 mL per 1 mmol of substrate) was added, followed by a solid sodium bicarbonate (5 g per 1 mmol of substrate, in some cases a small amount of water was added to facilitate dissolution). Layers were separated, and water phase was extracted with chloroform (same volume as water phase), combined organic layers were washed with brine, dried over anhydrous sodium sulfate, filtered and concentrated under reduced pressure. Products were purified by flash column chromatography on silica gel (from 50- to 160-fold silica gel excess, *n*-hexane:AcOEt).

Exemplary method of obtaining **3f**.

To a solution of **2,5-dimethyl-2-(4-nitrophenyl)benzo[d][1,3]dithiole**, **2f** (1.52 g, 5.0 mmol) in dry chloroform (100 mL), under argon atmosphere, was added dropwise solution of bromine (440 µL, 8.5 mmol, 1.7 equiv) in dry chloroform (50 ml). Reaction mixture was stirred for the next 45 min and quenched by addition of solid sodium bicarbonate (15 g) and water solution of sodium thiosulfate (10%, 500 mL). Aqueous phase was separated, extracted once with chloroform (250 mL), organic layers were combined, washed with brine (50 mL), dried over anhydrous sodium sulfate, filtered and concentrated under reduced pressure to obtain crude reaction mixture of products (2.10 g) which was purified by flash column chromatography (100-fold silica gel excess, n-hexane:AcOEt 19:1) to obtain mixture of

3-bromo-6-methyl-2-(4-nitrophenyl)benzo[*b*][1,4]dithiine (A), and **2-bromo-6-methyl-3-(4-nitrophenyl)benzo[*b*][1,4]dithiine** (B), **3f**, (1.77 g, 4.68 mmol, yield 94% as a golden crystals).

Spectral data of the regioisomers mixture **3-bromo-2-hexyl-6-methylbenzo[*b*][1,4]dithiine** (A) and **2-bromo-3-hexyl-6-methylbenzo[*b*][1,4]dithiine** (B), **3a**, (0.42 g, 1.22 mmol, yield 54% as a colorless oil).

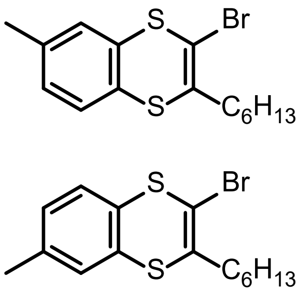

A

B

3a

IR (ATR) (cm^{-1}): $\tilde{\nu}$=2952; 2924; 2854; 1585; 1462; 1377; 1264; 1144; 1125; 865; 799; 759; 724; 695.

^1H NMR (500 MHz, $CDCl_3$):

For regioisomer A: δ=7.00 (s, 1H); 6.99 (d, *J* 7.8, 1H); 6.89 (d, *J* 7.8, 1H); 2.43 (t, *J* 7.3; 2H); 2.27 (s, 3H); 1.60-1.52 (m, 2H); 1.37-1.28 (m, 6H); 0.89 (t, *J* 6.6; 3H).

For regioisomer B: δ= 7.05 (d, *J* 7.8, 1H); 6.94 (s, 1H); 6.88 (d, *J* 7.8, 1H); 2.43 (t, *J* 7.3; 2H); 2.27 (s, 3H); 1.60-1.52 (m, 2H); 1.37-1.28 (m, 6H); 0.89 (t, *J* 6.6; 3H).

^{13}C NMR (125 MHz, $CDCl_3$): δ=136.8; 135.8; 135.7; 135.2; 133.3; 131.8; 129.8; 126.7; 126.6; 122.2; 122.0; 121.4; 121.1; 106.0; 106.0; 39.9; 31.6; 28.2; 27.7.; 22.6; 21.0; 14.1.

HRMS ($APCI^+$) for $C_{15}H_{20}BrS_2$ $[M+H]^+$: calcd: 343.0190; found: 343.0198.

Spectral data of the regioisomers mixture **3-bromo-6-methyl-2-tetradecyl benzo[*b*] [1,4]dithiine** and **2-bromo-6-methyl-3-tetradecylbenzo[*b*][1,4]dithiine**, **3b**, (0.58 g, 1.27 mmol, yield 64% as a waxy solid).

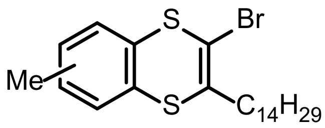

3b

IR (ATR) (cm^{-1}): $\tilde{\nu}$=2963; 2951; 2917; 2868; 2849; 1587; 1463; 1376; 1143; 1127; 873; 855; 804; 798; 719; 695; 686.

^1H NMR (500 MHz, $CDCl_3$): δ=7.02-6.98 (m, 2H); 6.89 (d, 1H, *J* 7.9); 2.43 (t, *J* 7.3, 2H); 2.28 (s, 3H); 1.62-1.53 (m, 2H); 1.39-1.18 (m, 22H); 0.88 (t, *J* 6.8; 3H).

^{13}C NMR (125 MHz, $CDCl_3$): δ=135.8; 126.7; 122.1; 121.3; 39.9; 31.9; 29.7; 29.7; 29.6; 29.5; 29.4; 29.4; 28.5; 27.8; 22.7; 21.0; 14.1.

mp.: 32-34°C.

HRMS ($APCI^+$) for $C_{23}H_{36}BrS_2$ $[M+H]^+$: calcd: 455.1442; found: 455.1444.

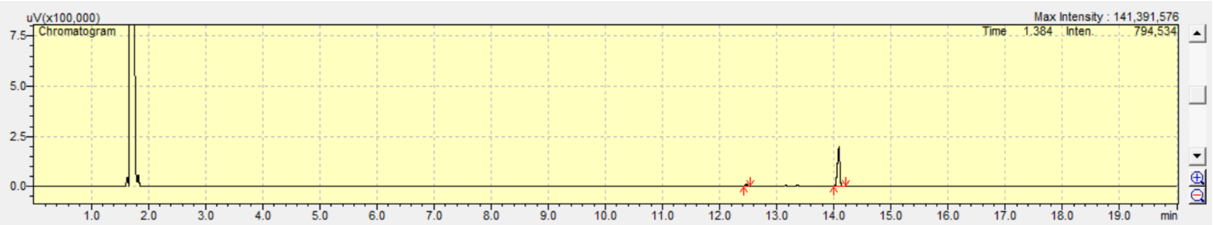

Figure S2. Chromatogram GC of the compound **3b**.

Spectral data of the regioisomers mixture **2-benzyl-3-bromo-6-methylbenzo[*b*][1,4]dithiine** (A) and **3-benzyl-2-bromo-6-methylbenzo[*b*][1,4]dithiine** (B), **3c**, (1.46 g, 4.18 mmol, yield 84% as a colorless crystal):

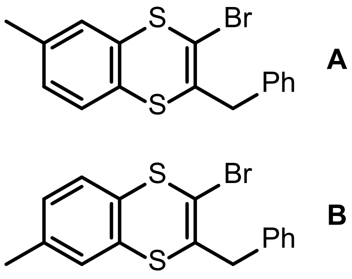

3c

IR (ATR) (cm^{-1}): $\tilde{\nu}$=3065; 3029; 2915; 1588; 1494; 1462; 1450; 1420; 1145; 1125; 1029; 972; 810; 801; 733; 693.

^{1}H NMR (500 MHz, $CDCl_3$):

For regioisomer A: δ=7.33-7.24 (m, 5H); 7.08 (d, *J* 7.9, 1H); 6.96 (s, 1H); 6.91 (d, *J* 7.9, 1H); 3.81 (s, 2H); 2.29 (s, 3H)

For regioisomer B: δ=7.33-7.24 (m, 5H); 7.03 (s, 1H); 7.02 (d, *J* 8.0, 1H); 6.92 (d, *J* 8.0, 1H); 3.81 (s, 2H); 2.29 (s, 3H).

^{13}C NMR (125 MHz, $CDCl_3$): δ=137.1; 136.5; 136.0; 135.9; 135.2; 133.1; 132.1; 131.8; 128.6; 128.5; 127.0; 126.9; 126.8; 122.3; 122.1; 121.5; 121.2; 103.2; 103.1; 45.7; 45.7; 21.0; 21.0.

^{1}H NMR (500 MHz, acetone-d_6):

For mixture of regioisomers: δ=7.47-7.31 (m, 7H); 7.17 and 7.16 (2d ovl, *J* 6.7 and 6.6, 1H); 3.97 (d, *J* 2.2, 2H); 2.42 (s, 3H).

mp.: 100-102°C (acetone, with decomp.)

HRMS ($APCI^+$) for $C_{16}H_{14}BrS_2$ $[M+H]^+$: calcd: 348.9720; found: 348.9725.

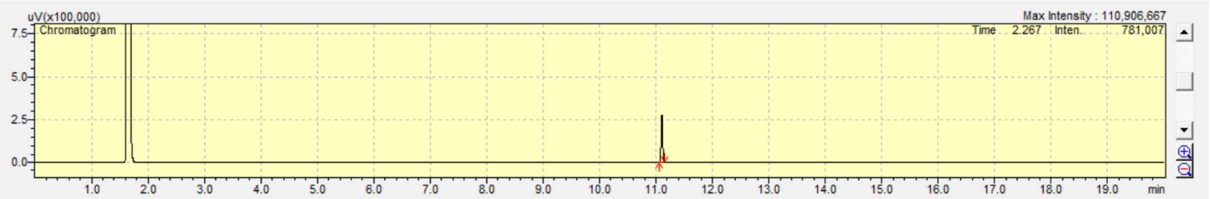

Figure S3. Chromatogram GC of the compound **3c**.

Spectral data of the regioisomers mixture **2-bromo-3-(bromomethyl)-6-methylbenzo[*b*][1,4]dithiine** (A) and **3-bromo-2-(bromomethyl)-6-methylbenzo[*b*][1,4]dithiine** (B), **3d**, (A/B : 7/1), (0.69 g, 1.96 mmol, yield 39% as a colorless crystal):

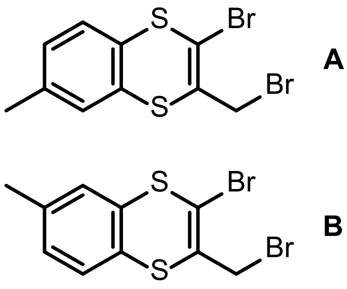

3d (A/B : 7/1)

IR (ATR) (cm^{-1}): $\tilde{\nu}$=2978; 2913; 2858; 1464; 1400; 1259; 1228; 1177; 1105; 1045; 889; 871; 827; 804; 754; 688; 680.

^{1}H NMR (500 MHz, $CDCl_3$):

For regioisomer A: δ=7.27 (d, *J* 7.9, 1H); 7.15 (s, 1H); 6.94 (d, *J* 7.9, 1H); 3.68 (s, 2H); 2.30 (s, 3H).

For regioisomer B: δ=7.23 (s, 1H); 7.22 (d, *J* 8.3, 1H); 6.97 (d, *J* 8.3, 1H); 3.68 (s, 2H); 2.30 (s, 3H).

^{13}C NMR (125 MHz, $CDCl_3$):

For regioisomer A: δ=138.3; 137.1; 132.8; 129.7; 129.6; 127.9; 127.4; 78.6; 35.5; 21.0.

For regioisomer B: δ=138.5; 136.5; 133.0; 130.5; 129.6; 127.8; 127.4; 78.3; 35.4; 20.9.

mp.: 90-93°C (acetone).

HRMS (APCI$^+$) for $C_{10}H_9Br_2S_2$ $[M+H]^+$: calcd: 350.8512; found: 350.8507.

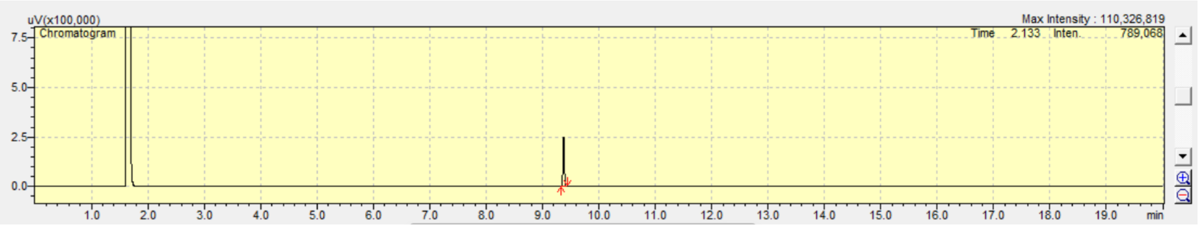

Figure S4. Chromatogram GC of the compound **3d**.

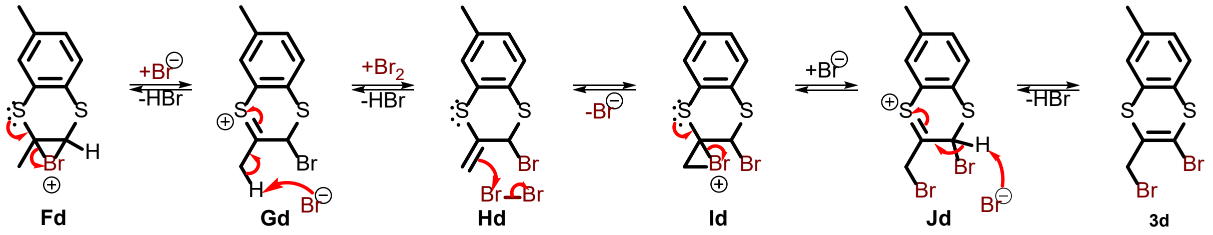

Figure S5. Probable mechanism of formation **3d** (numbering of the substructures correspond to **Scheme 3** in main text).

Spectral data of the regioisomers mixture **2-bromo-6-methyl-3-phenylbenzo[*b*][1,4]dithiine** and **3-bromo-6-methyl-2-phenylbenzo[*b*][1,4]dithiine**, **3e**, (3.40 g, 10.15 mmol, yield 71% as a colorless crystal):

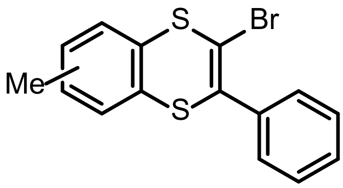

3e

IR (ATR) (cm^{-1}): $\tilde{\nu}$=3078; 3051; 2915; 1487; 1461; 1439; 1375; 1253; 1140; 1075; 1030; 835; 809; 766; 732; 701; 690.

^{1}H NMR (500 MHz, $CDCl_3$): δ=7.46 (d, *J* 7.4, 2H); 7.39-7.27 (m, 5H); 7.13 (d, *J* 7.9, 1H), 2.36 (s, 3H).

^{13}C NMR (125 MHz, $CDCl_3$): δ=138.7; 138.6; 137.6; 137.2; 135.5; 135.0; 132.0; 131.6; 129.8; 129.8; 129.3; 129.1; 128,8; 128.5; 128.4; 128.2; 127.7; 127.6; 105.6; 105.2; 20.9; 20.9.

mp.: 94-96°C (CH_2Cl_2: *n*-pentane 1:1 (*v/v*)).

HRMS (APCI$^+$) for $C_{15}H_{11}BrS_2$ $[M^{+\cdot}]$: calcd: 333.9488; found: 333.9486.

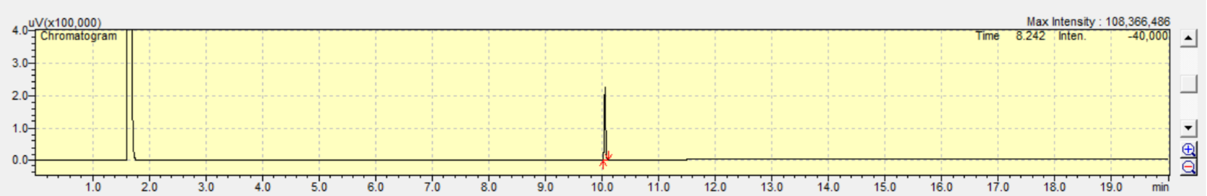

Figure S6. Chromatogram GC of the compound **3e**.

Spectral data of the equimolar regioisomers mixture **3-bromo-6-methyl-2-(4-nitrophenyl)benzo[*b*][1,4]dithiine** (A) and **2-bromo-6-methyl-3-(4-nitrophenyl)benzo[*b*] [1,4]dithiine** (B), **3f**, (1.78 g, 4.68 mmol, yield 94% as a golden crystals):

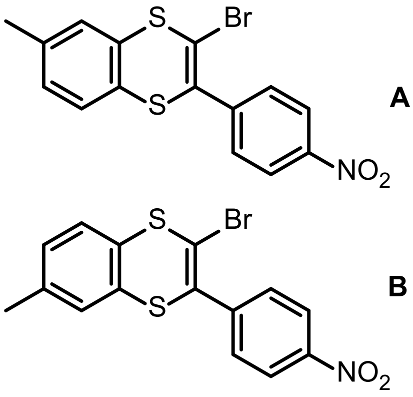

3f (1:1)

IR (ATR) (cm^{-1}): $\tilde{\nu}$=3101; 3072; 2920; 2847; 1588; 1516; 1462; 1347; 1254; 1111; 1013; 947; 859; 851; 824; 805; 763; 739; 688; 670.

^{1}H NMR (500 MHz, $CDCl_3$): δ=8.23 (d, *J* 8.7, 2H); 7.65 (d, *J* 8.7, 2H); 7.35 (d, *J* 8.3, 2H); 7.31 (s, 1H); 7.17 (d, *J* 8.3, 1H); 2.38 (s, 3H).

^{13}C NMR (125 MHz, $CDCl_3$): 147.6; 144.10; 144.08; 139.2; 139.1; 135.2; 134.9; 134.4; 134.1; 131.8; 130.92; 130.90; 130.7; 129.6; 129.5; 128.7; 128.6; 127.8; 127.79; 123.5; 109.4 109.0; 20.94; 20.91.

mp.: 172-174°C (acetone).

HRMS (APCI$^+$) for $C_{15}H_{11}BrNO_2S_2$ $[M+H]^+$: calcd: 379.9415; found: 379.9410.

Elemental analysis (A/B : 1/1) for $C_{15}H_{10}BrNO_2S_2$ (MW= 380.27); calcd: C, 47.38; H, 2.65; Br, 21.01; N, 3.68; S, 16.86.; found: C, 47.36; H, 2.71; Br, 21.09; N, 3.67; S, 16.89.

Spectral data of the regioisomers mixture **3-bromo-6-methyl-2-(4-nitrophenyl) benzo[*b*][1,4]dithiine** (A) and **2-bromo-6-methyl-3-(4-nitrophenyl)benzo[*b*] [1,4]dithiine** (B), **3f**, (A/B : 8.33/1):

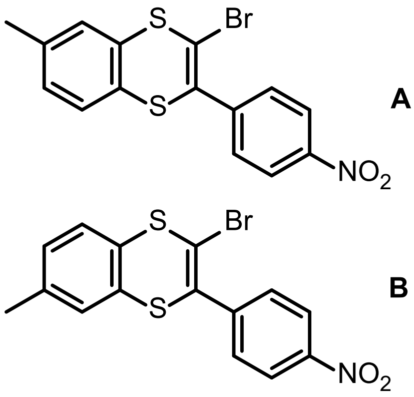

3f (A/B : 8.33/1)

^{1}H NMR (500 MHz, $CDCl_3$);

For regioisomer A: δ=8.23 (d, *J* 8.7, 2H); 7.65 (d, *J* 8.7, 2H); 7.35 (d, *J* 8.3, 2H); 7.31 (s, 1H) 7.17 (d, *J* 8.3, 1H); 2.38 (s, 3H).

For regioisomer B: δ=8.23 (d, *J* 8.7, 2H); 7.64 (d, *J* 8.7, 2H); 7.36 (d, *J* 8.3, 2H); 7.30 (s, 1H) 7.17 (d, *J* 8.3, 1H); 2.38 (s, 3H).

^{13}C NMR (125 MHz, $CDCl_3$):

For regioisomer A: δ=147.6; 144.1; 139.2; 134.4; 134.1; 131.8; 130.9; 129.5; 128.6; 127.8; 123.5; 109.4; 20.9.

For regioisomer B: δ= 147.6; 144.2; 139.1; 135.2; 134.9; 130.9; 130.7;129.6; 128.7; 127.8; 123.5; 109.0; 20.9.

Elemental analysis (A/B : 8.33/1) for $C_{15}H_{10}BrNO_2S_2$ (MW= 380.27); calcd: C, 47.38; H, 2.65; Br, 21.01; N, 3.68; S, 16.86.; found: C, 47.44; H, 2.65; Br, 20.94; N, 3.57; S, 16.97.

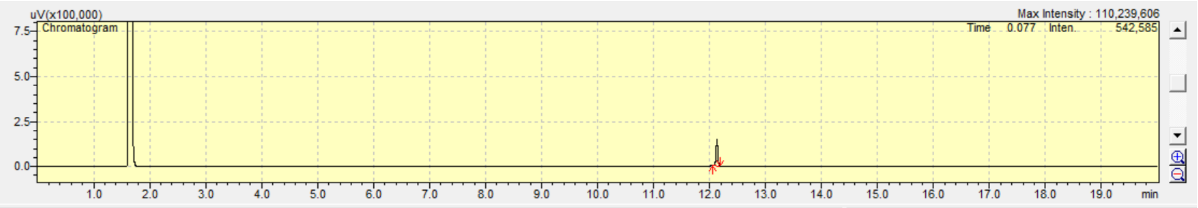

Figure S7. Chromatogram GC of the compound **3f (A/B : 8.33/1)**.

Spectral data of the regioisomers mixture **3-bromo-2-(3,4-dimethoxyphenyl)-6-methylbenzo[*b*][1,4]dithiine** (A) and **2-bromo-3-(3,4-dimethoxyphenyl)-6-methylbenzo[*b*][1,4]dithiine** (B), **3g**, (1.41 g, 3.57 mmol, 89%):

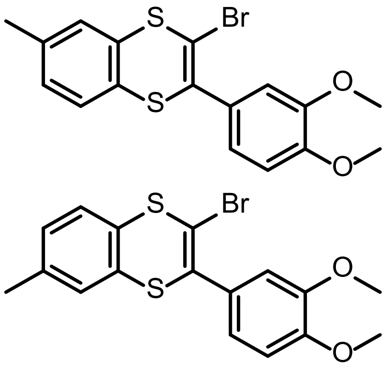

A

B

3g (1:1)

IR (ATR) (cm^{-1}): $\tilde{\nu}$=3004; 2927; 2828; 1598; 1584; 1510; 1464; 1440; 1404; 1326; 1258; 1240; 1163; 1138; 1041; 1021; 980; 905; 848; 811; 793; 766; 735; 698; 688.
^{1}H NMR (500 MHz, $CDCl_3$):
For regioisomer A: δ=7.32 (d, *J* 7.9, 1H); 7.30 (s, 1H); 7.13 (d, *J* 7.9, 1H); 7.07 (d, *J* 8.3, 1H); 7.01 (s, 1H); 6.87 (d, *J* 8.3, 1H); 3.91 (s, 3H); 3.90 (s, 3H); 2.36 (s, 3H).
For regioisomer B: δ=7.36 (d, *J* 7.9, 1H); 7.27 (s, 1H); 7.13 (d, *J* 7.9, 1H); 7.07 (d, *J* 8.3, 1H); 7.01 (s, 1H); 6.87 (d, *J* 8.3, 1H); 3.91 (s, 3H); 3.90 (s, 3H); 2.36 (s, 3H).
^{13}C NMR (125 MHz, $CDCl_3$): 149.5; 148.4; 138.7; 138.6; 137.9; 137.5; 135.5; 135.2; 132.1; 131.8; 129.8; 129.2; 129.1; 128.5; 128.4; 127.7; 127.6; 122.9; 122.8; 113.0; 113.0; 110.6; 104.0; 103.7; 56.0; 55.9; 20.9; 20.9.
mp.: 117-121°C (acetone, with decomp.).
HRMS ($APCI^+$) for $C_{17}H_{16}O_2S_2Br$ $[M+H]^+$: calcd: 394.9775; found: 394.9776.

Spectral data of the regioisomers mixture **2-bromo-6-methyl-3-(6-methylbenzo[*d*][1,3]dithiol-5-yl)benzo[*b*][1,4]dithiine** (A) and **3-bromo-6-methyl-2-(6-methylbenzo[*d*][1,3]dithiol-5-yl)benzo[*b*][1,4]dithiine** (B), **3h**, (0.22 g, 0.51mmol, yield 51%):

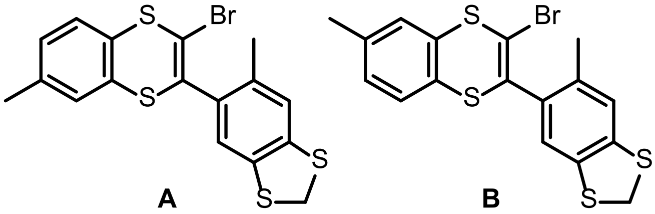

3h (1:1)

IR (ATR) (cm^{-1}): $\tilde{\nu}$=2962; 2915; 1461; 1448; 1258; 1084; 1015; 909; 863; 800; 753; 693; 686; 668.
^{1}H NMR (500 MHz, $CDCl_3$):
For regioisomer A: δ=7.28 (d, *J* 7.9, 1H); 7.22 (s, 1H); 7.11 (d, *J* 7.9, 1H); 7.08 (s, 1H); 6.99 (s, 1H); 4.51 (d, *J* 13.6, 1H); 4.49 (d, *J* 13.6, 1H); 2.35 (s, 3H); 2.09 (s, 3H).
For regioisomer B: δ=7.30 (d, *J* 7.9, 1H); 7.22 (s, 1H); 7.11 (d, *J* 7.9, 1H); 7.08 (s, 1H); 6.99 (s, 1H); 4.51 (d, *J* 13.6, 1H); 4.49 (d, *J* 13.6, 1H); 2.35 (s, 3H); 2.10 (s, 3H).

^{13}C NMR (125 MHz, CDCl$_3$): δ=139.5; 138.8; 138.5; 136.1; 135.8; 135.8; 135.6; 135.1; 134.9; 134.8; 134.2; 134.2; 131.5; 131.4; 129.4; 129.1; 128.6; 128.3; 127.7; 127.6; 123.9; 122.8; 108.3; 108.0; 36.3; 20.9; 20.9; 19.3; 19.3.
HRMS (APCI-TOF) for $C_{17}H_{12}BrS_4$ $[M-H]^+$: calcd: 422.9005; found: 422.9003.

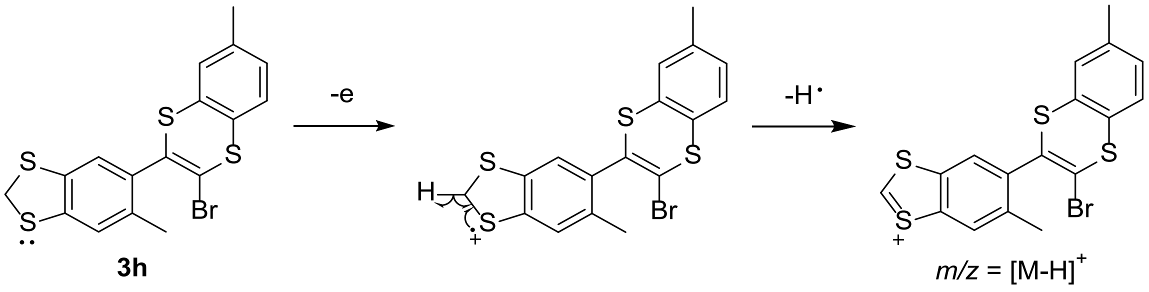

Figure S8. Fragmentation **3h** to ion *m/z* = $[M-H]^+$.

Spectral data of the regioisomers mixture **2-bromo-6-methyl-3-(naphthalen-2-yl) benzo[*b*][1,4]dithiine** and **3-bromo-6-methyl-2-(naphthalen-2-yl)benzo[*b*][1,4]dithiine**, **3i**, (1.39 g, 3.61 mmol, yield 72% as a colorless crystal):

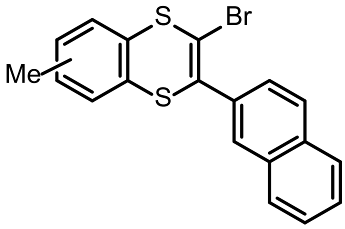

3i

IR (ATR) (cm^{-1}): $\tilde{\nu}$=3056; 2972; 2916; 2858; 1597; 1504; 1463; 1375; 1257; 1122; 934; 897; 850; 818; 813; 790; 740; 729; 688;
^{1}H NMR (500 MHz, CDCl$_3$): δ=7.96 (br s, 1H); 7.86-7.82 (m, 3H); 7.55 (d, *J* 7.9, 1H); 7.52-7.48 (m, 3H); 7.40-7.29 (m, 2H); 7.14 (d, *J* 7.9, 1H); 2.38 and 2.37 (2s, 3H).
^{13}C NMR (125 MHz, CDCl$_3$): δ=138.8; 138.7; 137.7; 137.2; 135.5; 135.1; 134.9; 133.1; 132.8; 132.1; 131.7; 129.6; 129.6; 129.3; 129.2; 128.6; 128.5; 128.3; 127.8; 127.7; 127.7; 127.7; 127.0; 126.9; 126.9; 126.5; 105.8; 105.5; 20.9; 20.9.
mp.: 128-131°C and 138-141°C (CH_2Cl_2:acetone 1:1 (*v/v*)).
HRMS (APCI$^+$) for $C_{19}H_{14}BrS_2$ $[M+H]^+$: calcd: 384.9720; found: 384.9715.

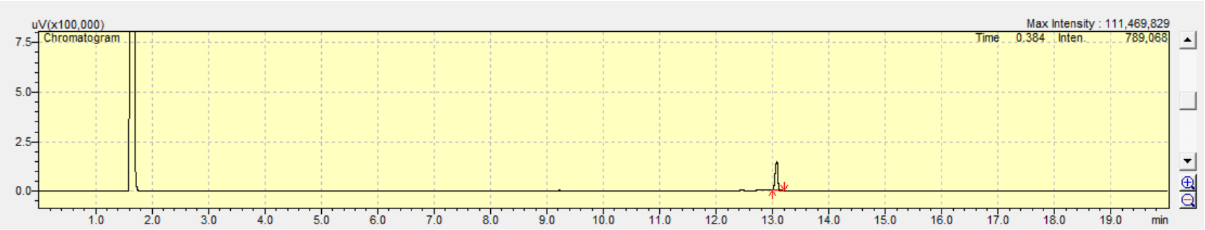

Figure S9. Chromatogram GC of the compound **3i**.

3.4 *Protocol for synthesis of* ***3j****, 4, and* ***5****:*

3.4.1 With methodology outlined in subsection 3.3 General synthesis for obtaining dithiine ***3*** *– with* ***1****.7 equiv of Bromine, were obtained:*

- mixture of regioisomers **3-bromo-2-(4-methoxyphenyl)-6-methylbenzo[*b*][1,4]dithiine** and **2-bromo-3-(4-methoxyphenyl)-6-methylbenzo[*b*][1,4]dithiine**, **3j**, (trace);
- mixture of regioisomers **2-(4-methoxyphenyl)-6-methylbenzo[*b*][1,4]dithiine** and **2-(4-methoxyphenyl)-7-methylbenzo[*b*][1,4]dithiine**, **4**, 0.44 g, (1.52 mmol, yield 30%);
- **3,3'-bis(4-methoxyphenyl)-6,6'-dimethyl-2,2'-bibenzo[*b*][1,4]dithiine**, **5**, 0.91 g, (1.60 mmol, yield 64%).

Spectral data of: **2-bromo-3-(4-methoxyphenyl)-6-methylbenzo[*b*][1,4]dithiine**, **3j**, mixture of regioisomers **2-(4-methoxyphenyl)-6-methylbenzo[*b*][1,4]dithiine** and **2-(4-methoxyphenyl)-7-methylbenzo[*b*][1,4]dithiine**, **4**, **3,3'-bis(4-methoxyphenyl)-6,6'-dimethyl-2,2'-bibenzo[*b*][1,4]dithiine**, **5**, (as a colorless solid):

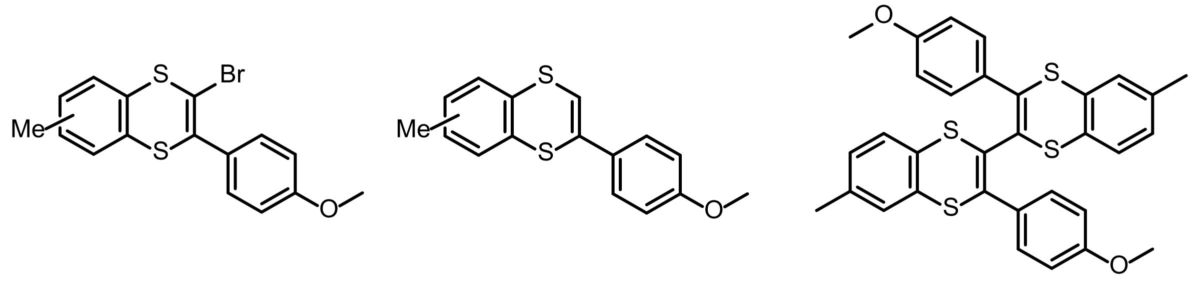

3j (trace) **4 (30%)** **5 (64%)**

Mixture of **4** and compound **5** (0.43:1 molar ratio):
^{1}H NMR (500 MHz, $CDCl_3$):
For equimolar mixture of regioisomers **4**: δ=7.54 (d, *J* 8.8, 2H); 7.33 (d, *J* 7.9, 1H); 7.25 – 7.22 (m, 2H); 7.18 (s, 1H) 7.03 (d, *J* 7.8, 1H); 6.86 (d, *J* 8.8, 2H); 6.53 (d, *J* 8.3, 1H); 3.80 (s, 3H); 2.31 (s, 3H).

For compound **5** δ=7.43 (d, *J* 8.6, 4H); 7.31 (d, *J* 7.9, 2H); 7.28 (s, 2H); 7.10 (d, *J* 7.9, 2H); 6.88 (d, *J* 8.6, 4H); 3.81 (s, 6H); 2.34 (s, 6H).
^{13}C NMR (125 MHz, $CDCl_3$): δ=160.0; 159.9; 140.9; 140.5; 138.6; 138.5;138.0; 137.9; 137.7; 137.4; 135.6; 135.2; 135.2; 134.4; 132.2; 131.8; 131.7; 131.3; 131.3; 131.0; 129.9; 129.6; 129.2; 129.1; 129.1; 128.7; 128.5; 128.4; 128.4; 128.3; 128.2; 127.6; 127.6; 127.5; 116.4; 115.9; 113.9; 113.5; 103.8; 103.5; 55.3; 55.3; 20.9; 20.9.

HRMS (APCI$^+$) for $C_{16}H_{14}BrOS_2$ (**3j**) $[M+H]^+$: calcd: 364.9669; found: 364.9666.
HRMS (APCI$^+$) for $C_{16}H_{15}OS_2$ (**4**) $[M+H]^+$: calcd: 287.0564; found: 287.0567.
HRMS (APCI$^+$) for $C_{32}H_{27}O_2S_4$ (**5**) $[M+H]^+$: calcd: 571.0892; found: 571.0894.

Spectral data of **3,3'-bis(4-methoxyphenyl)-6,6'-dimethyl-2,2'-bibenzo[*b*][1,4]dithiine**, **5**, (0.35 g, 0.61 mmol, yield 25% after crystallization as colorless crystals):

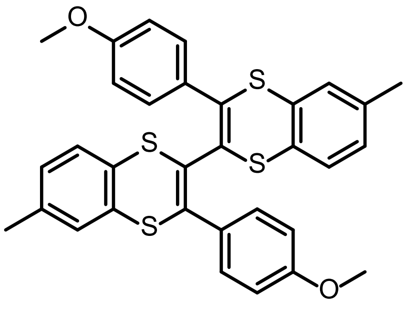

5

IR (ATR) (cm^{-1}): $\tilde{\nu}$=3071; 3003; 2892; 2829; 1605; 1550; 1503; 1463; 1294; 1243; 1173; 1110; 1036; 847; 822; 805; 744; 699; 689.

^{1}H NMR (500 MHz, $CDCl_3$): δ=7.43 (d, *J* 8.6, 4H); 7.31 (d, *J* 7.9, 2H); 7.29 (s, 2H); 7.12 (d, *J* 7.9, 2H); 6.89 (d, *J* 8.6, 4H); 3.83 (s, 6H); 2.36 (s, 6H).

^{13}C NMR (125 MHz, $CDCl_3$): δ=159.9; 138.6; 137.5; 135.3; 132.2; 131.4; 129.6; 129.1; 128.4; 127.7; 113.5; 103.9; 55.3; 20.9.

mp.: 133-135°C (acetone).

HRMS (APCI$^+$) for $C_{32}H_{27}O_2S_4$ $[M+H]^+$: calcd: 571.0892; found: 571.0894.

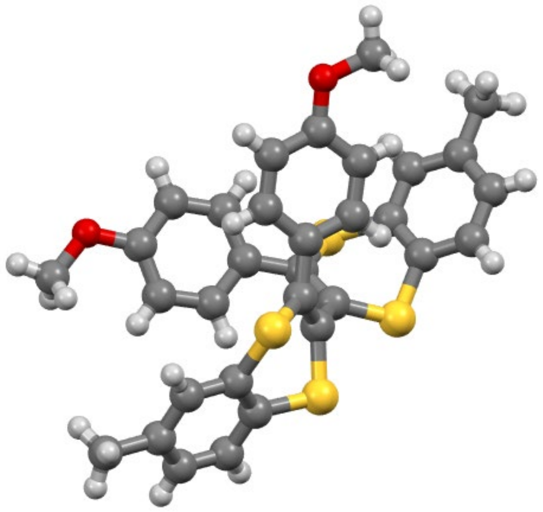

Figure S10. **Crystal Data of 5, CCDC 2571235**
$C_{32}H_{26}O_2S_4$, M_r = 570.77, triclinic, P-1 (No. 2), a = 9.6769(11) Å, b = 11.1216(13) Å, c = 14.2025(17) Å, α = 104.035(10)°, β = 93.139(9)°, γ = 101.083(10)°, V = 1447.0(3) Å^3, T = 293.15(10) K, Z = 2, Z' = 1, μ(Cu K_α) = 3.232, 4940 reflections measured, 3832 unique (R_{int} = 0.0182) which were used in all calculations. The final wR_2 was 0.2184 (all data) and R_1 was 0.0654 (I > 2(I)).

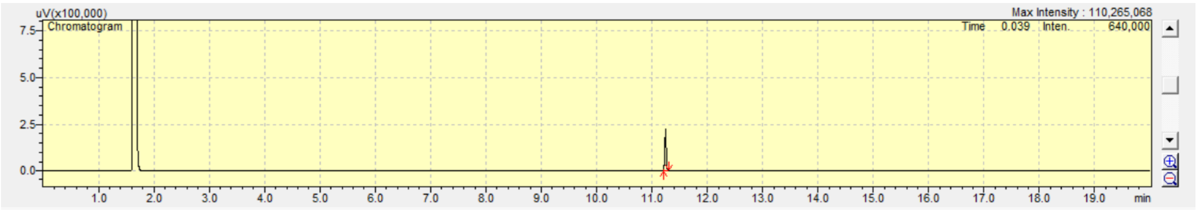

Figure S11. Chromatogram GC of the compound **5**.

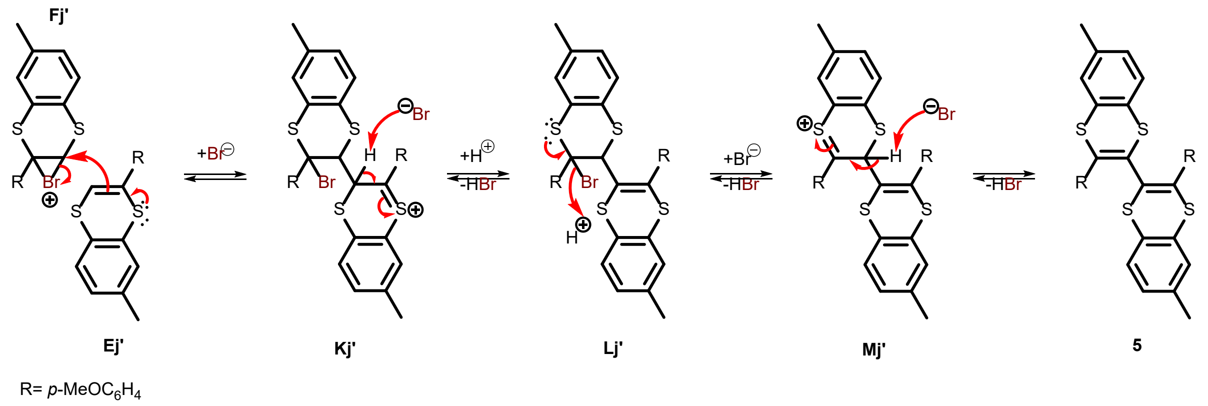

Figure S12. Probable mechanism of formation **5** (numbering of the substructures correspond to **Scheme 3** in main text).

*3.4.2 According to general synthesis for obtaining dithiine **3** – with **1.0** equiv of Bromine, were obtained:*

- mixture of regioisomers **2-(4-methoxyphenyl)-6-methylbenzo[*b*][1,4]dithiine** and **2-(4-methoxyphenyl)-7-methylbenzo[*b*][1,4]dithiine**, **4**, (0.19 g, 0.65 mmol, yield 65% as a colorless crystal)
- substrate of **2-(4-methoxyphenyl)-2,5-dimethylbenzo[*d*][1,3]dithiole**, **2j**, (0.08g, 0.28 mmol, recovery 28%).

Spectral data are consistent with the descriptions above.

*3.4.3 According to general synthesis for obtaining dithiine **3** – with **2.2** equiv of Bromine, were obtained:*

- mixture of regioisomers **2-(4-methoxyphenyl)-6-methylbenzo[*b*][1,4]dithiine** and **2-(4-methoxyphenyl)-7-methylbenzo[*b*][1,4]dithiine**, **4**, (0.02 g, 0.08 mmol, yield 8% as a colorless crystal);
- **3,3'-bis(4-methoxyphenyl)-6,6'-dimethyl-2,2'-bibenzo[*b*][1,4]dithiine**, **5**, (0.25 g, 0.44 mmol, yield 87% as a colorless crystal).

Spectral data are consistent with the descriptions above.

*3.5 Oxidation of **3e** and **3g***

To a suspension of 1,4-benzo[*b*]dithiine derivative, **3e**/**3g** (0.50 mmol) in a glacial acetic acid (20 mL) a solution of H_2O_2 (30% H_2O, 4.58 g; 40 mmol H_2O_2; 80.0 equiv) was added and the resulting mixture was stirred at reflux for 24 h (Note: solids dissolves before reaching the boiling point). Then, the reaction mixture was cooled down and quenched with water (100 ml), the precipitated product was filtered off, washed with water and purified by recrystallization from CH_2Cl_2:acetone (1:1 (*v/v*)).

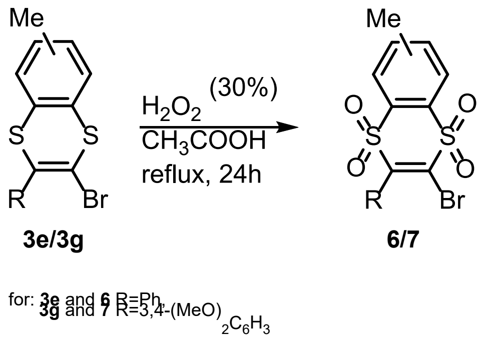

Scheme S2. Synthesis of compounds **6** and **7**.

Spectral data for mixture of the regioisomers **2-bromo-6-methyl-3-phenylbenzo[*b*][1,4]dithiine 1,1,4,4-tetraoxide** (A) and **3-bromo-6-methyl-2-phenylbenzo[*b*][1,4]dithiine 1,1,4,4-tetraoxide** (B), **6**, (0.18 g, 0.45mmol, yield 90% as a colorless crystal):

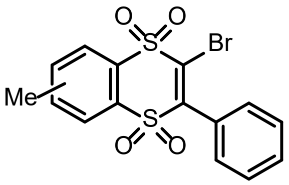

6

IR (ATR) (cm^{-1}): $\tilde{\nu}$=3062; 1584; 1488; 1444; 1333; 1326; 1298; 1210; 1165; 1140; 1115; 1074; 969; 842; 830; 779; 692; 679.

^{1}H NMR (500 MHz, $CDCl_3$);

For regioisomer A: δ=8.13 (d, *J* 8.1; 1H); 8.00 (s, 1H); 7.67 (d ovl, 1H); 7.59-7.47 (m, 5H); 2.58 (s, 3H).

For regioisomer B: δ=8.08 (d, *J* 8.1, 1H); 8.05 (s, 1H); 7.67 (d ovl, 1H); 7.59-7.47 (m, 5H); 2.57 (s, 3H).

^{13}C NMR (125 MHz, $CDCl_3$): δ=151.4; 151.1; 146.3; 145.9; 138.1; 136.4; 135.4; 134.8; 134.5; 133.7; 133.4; 133.0; 131.3; 129.5; 129.1; 128.0; 126.7; 126.6; 126.5; 126.4; 21.8; 21.8.

mp.: 269-272°C (acetone).

HRMS ($APCI^+$) for $C_{15}H_{12}BrO_4S_2$ $[M+H]^+$: calcd: 398.9360; found: 398.9354.

After crystallization (0.08 g, 0.20 mmol, 40% yield). the structure of obtained single crystal of regioisomer **3-bromo-6-methyl-2-phenylbenzo[*b*][1,4]dithiine 1,1,4,4-tetraoxide** (B) was elucidated using X-ray diffraction:

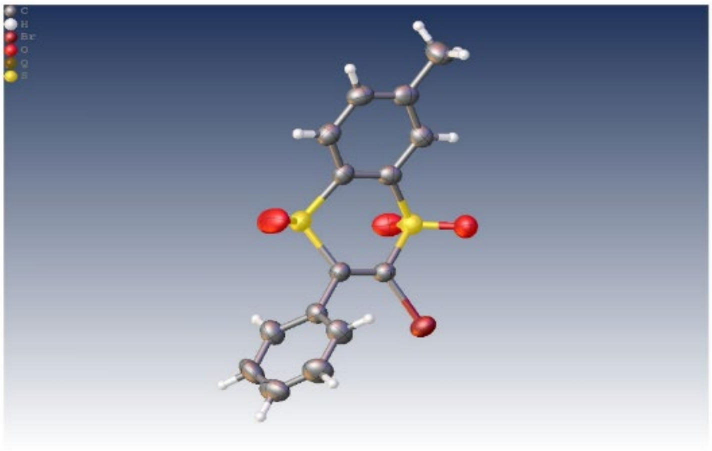

Figure S13. **Crystal Data of 6, CCDC 2571234** $C_{15}H_{11}BrO_4S_2$, M_r = 399.27, orthorhombic, $Pca2_1$ (No. 29), a = 7.41370(10) Å, b = 12.4876(2) Å, c = 16.3887(2) Å, $\alpha = \beta = \gamma = 90°$, V = 1517.25(4) Å^3, T = 293.15(10) K, Z = 4, Z' = 1, μ(Cu K_α) = 6.409, 14,034 reflections measured, 2537 unique (R_{int} = 0.0320) which were used in all calculations. The final wR_2 was 0.0782 (all data) and R_1 was 0.0288 (I > 2(I)).

Spectral data of the regioisomer **2-bromo-3-(3,4-dimethoxyphenyl)-6-methylbenzo[*b*][1,4]dithiine 1,1,4,4-tetraoxide** (A) and **3-bromo-2-(3,4-dimethoxyphenyl)-6-methyl benzo[*b*][1,4]dithiine 1,1,4,4-tetraoxide** (B), **7**, (0.17 g, 0.37 mmol, yield 74% as a colorless crystal):

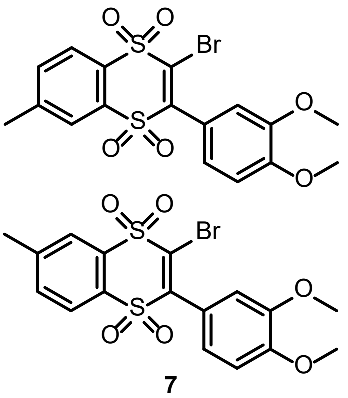

A

B

7

IR (ATR) (cm^{-1}): $\tilde{\nu}$=3022; 2938; 2841; 1595; 1580; 1467; 1442; 1332; 1321; 1268; 1245; 1167; 1138; 1118; 1034; 1018; 989; 911; 877; 825; 811; 768; 692; 672; 669; 661; 656.

^{1}H NMR (500 MHz, $CDCl_3$):

For regioisomer A: δ=8.12 (d, *J* 8.1; 1H); 8.01 (s, 1H); 7.66 (d ovl, 1H); 7.07 (s, 1H); 7.01 (s, 1H); 6.96 (s, 1H); 3.95 (s, 3H); 3.91 (s, 3H); 2.57 (s, 3H).

For regioisomer B: δ=8.09 (d, *J* 8.0; 1H); 8.05 (s, 1H); 7.67 (d ovl, 1H); 7.09 (s, 1H); 6.99 (s, 1H); 6.96 (s, 1H); 3.95 (s, 3H); 3.91 (s, 3H); 2.58 (s, 3H).

^{13}C NMR (125 MHz, $CDCl_3$): δ=151.5; 151.3; 151.0; 149.3; 146.3; 145.9; 138.0; 136.5; 135.3; 134.8; 134.5; 133.8; 133.0; 132.6; 126.7; 126.6; 126.5; 126.4; 123.1; 119.8; 112.1; 111.3; 56.2; 56.0; 21.8; 21.8.

mp.: 178-181°C (acetone).

HRMS ($APCI^+$) for $C_{17}H_{16}BrO_6S_2$ $[M+H]^+$: calcd: 458.9572; found: 458.9569.

4. References

1. Armarego, W.L.F.; Perrin, D.D. *Purification of Laboratory Chemicals*, 4th Ed.; Butterworth-Heinemann: Oxford, UK, **1996**.

2. Ratcliffe R., Rodehorst, R., Improved Procedure for Oxidations with the Chromium Trioxide-Pyridine Complex, *J. Org. Chem.*, **1970**, *35(11),* 4000-4002. https://doi.org/10.1021/jo00836a108

3. Nicklaus, C.M., Phua, P.H., Buntara, T., Noel, S., Heeres, H. J., Johannes G. de Vries, Ruthenium/1,1′-Bis(diphenylphosphino)ferrocene-Catalysed Oppenauer Oxidation of Alcohols and Lactonisation of α,ω-Diols using Methyl Isobutyl Ketone as Oxidant, *Adv. Synth. Catal.,* **2013**, *355(14-15)*, 2713-2718. https://doi.org/10.1002/adsc.201300438

4. Nunheim, N. F., Kaiser, B. Thiel, W. R., Ruthenium(III) Chloride as an Efficient Catalyst for the Selective Oxidation of Fatty Alcohols to Aldehydes, *Chemistry Select*, **2023**, *8(12)*, 1-5. DOI:10.1002/slct.202300346

5. Manfred Lissel, Herstellung und Reaktionen einiger Umpolungsreagenzien durch Phasentransfer-Katalyse. *Liebigs Ann. Chem.*, **1982**, *9*, 1589-1601. https://doi.org/10.1002/jlac.198219820902

6. Gleiter, R.; Hoffmann, H.; Irngartinger, H.; Nixdorf, M. Donor-Acceptor Spiro-Compounds - Syntheses, Structures, and Electronic Properties, *Chem. Ber.,* **1994**, *127(11)*, 2215-2224. DOI: 10.1002/CBER.1491271121

7. Ueda, N.; Shimizu, H.; Kataoka, T.; Hori, M. Non-stereospecific ring expansions of 5-membered heterocyclic sulfoxides. *Tetrahedron Lett.*, **1984**, *25(7)*, 757-760. https://doi.org/10.1016/S0040-4039(01)80018-6

5. Copies of IR, ^{1}H-, ^{13}C-, COSY-, HSQC-, HMBC-NMR

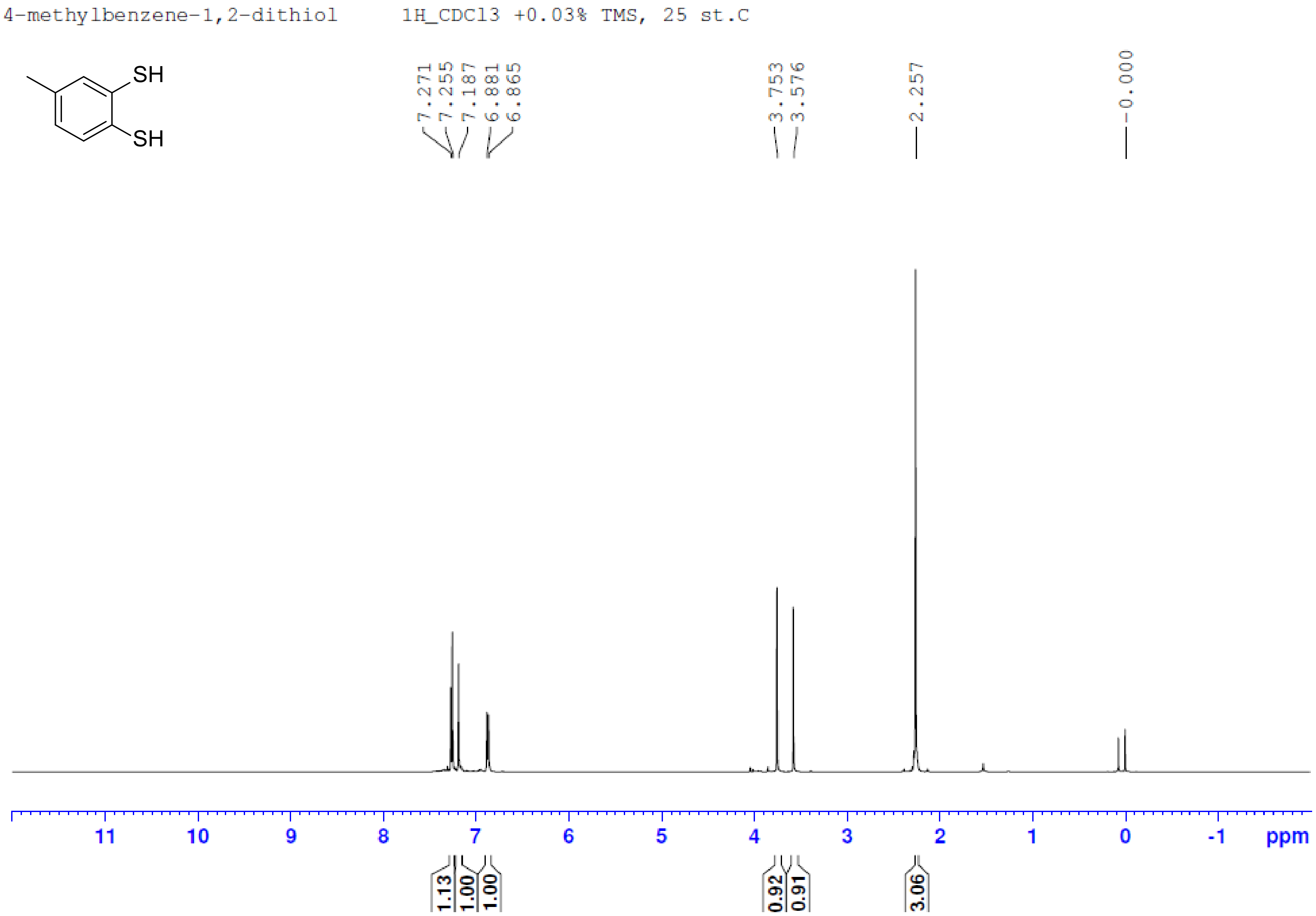

4-methylbenzene-1,2-dithiol 13C_CDCl3 + 0.03% TMS, 25 st.C

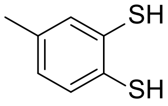

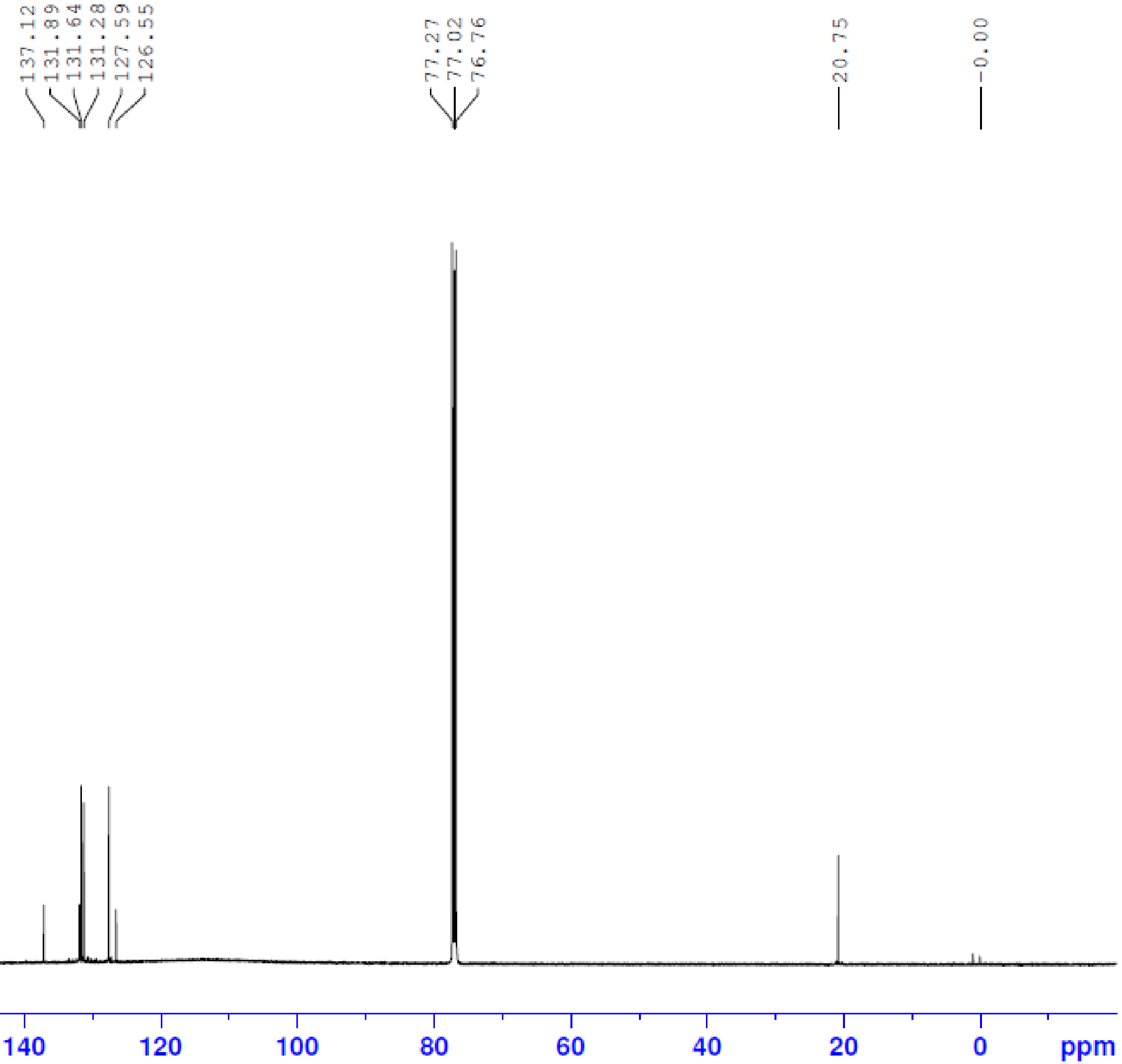

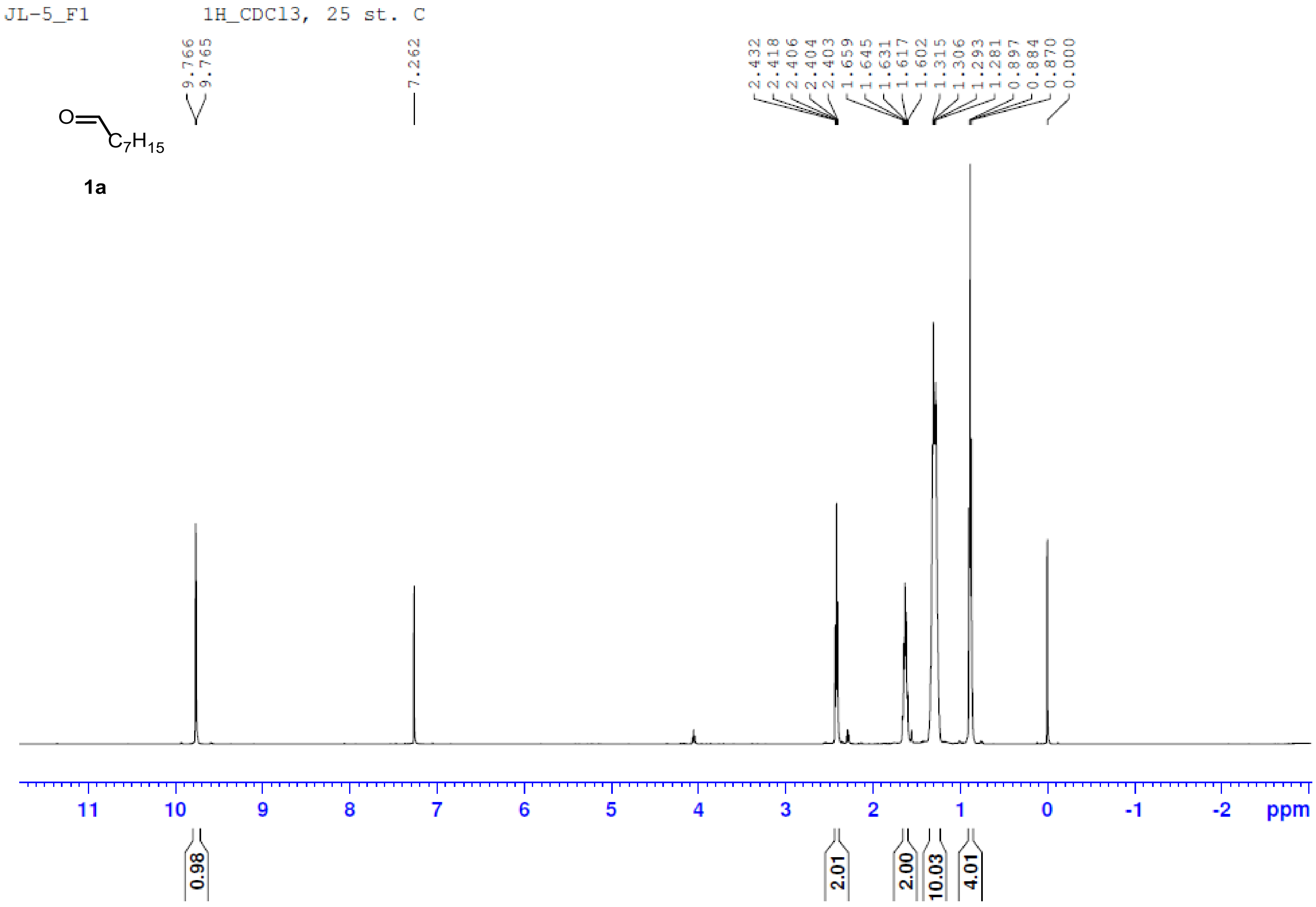
JL-5_F1
1H_CDCl3, 25 st. C
9.766
9.765
7.262
2.432
2.418
2.406
2.404
2.403
1.659
1.645
1.631
1.617
1.602
1.315
1.306
1.293
1.281
0.897
0.884
0.870
0.000
O
C7H15
1a
11
10
9
8
7
6
5
4
3
2
1
0
-1
-2
ppm
0.98
2.01
2.00
10.03
4.01

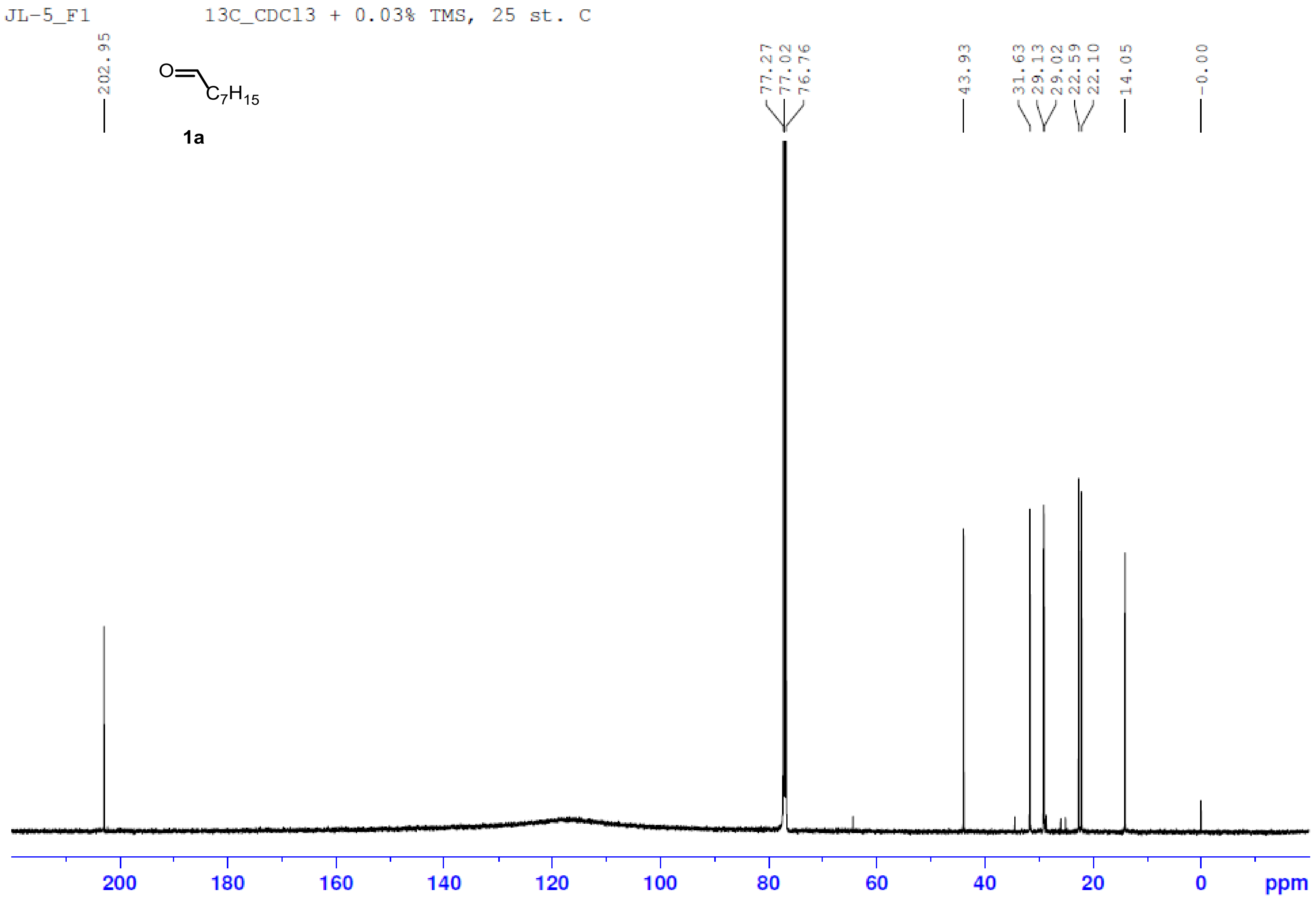
JL-5_F1
13C_CDCl3 + 0.03% TMS, 25 st. C
202.95
O
C_7H_{15}
1a
77.27
77.02
76.76
43.93
31.63
29.13
29.02
22.59
22.10
14.05
-0.00
200
180
160
140
120
100
80
60
40
20
0
ppm

JL-1_F1 1H_CDCl3, 25 st. C

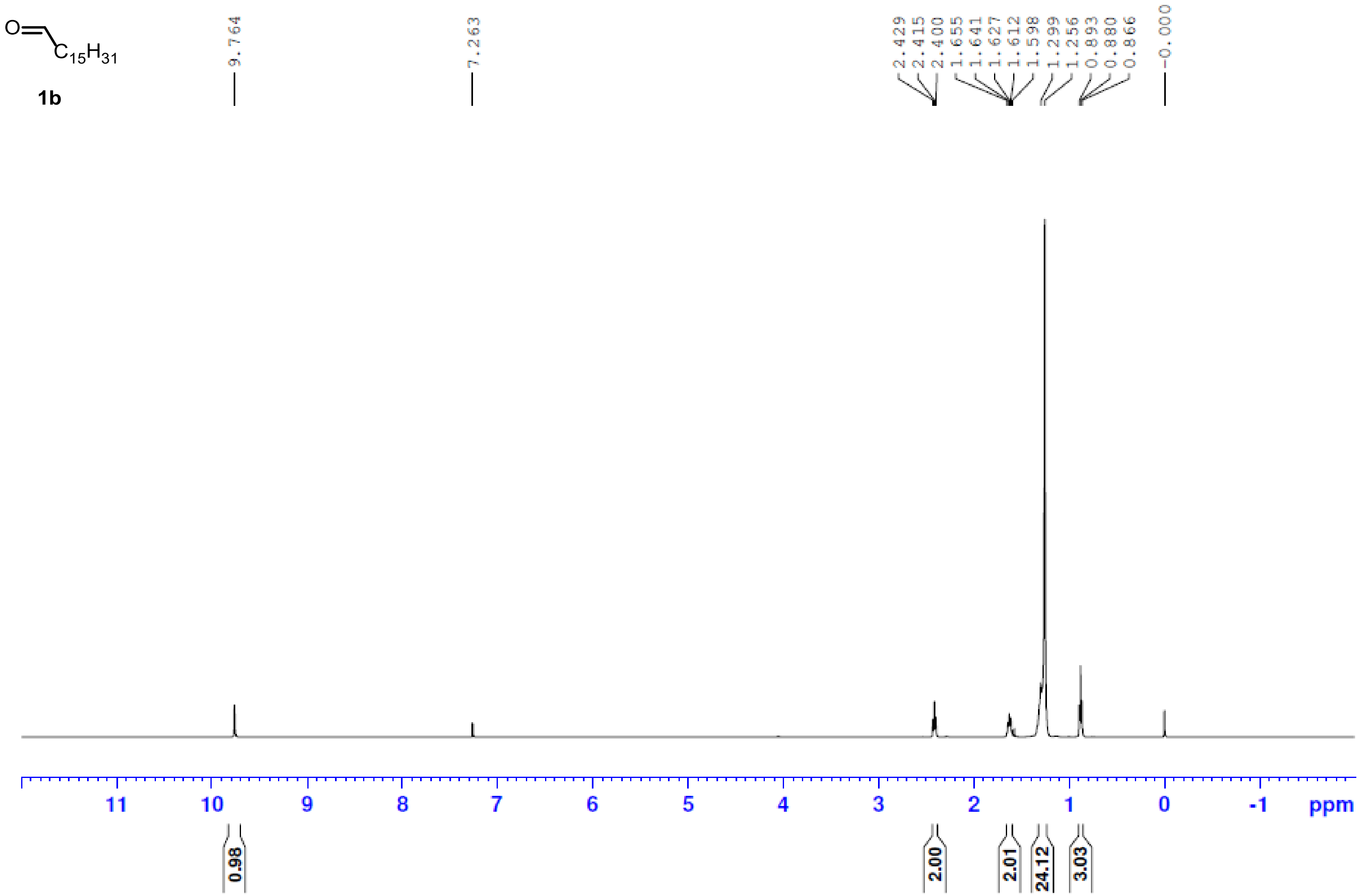

1b

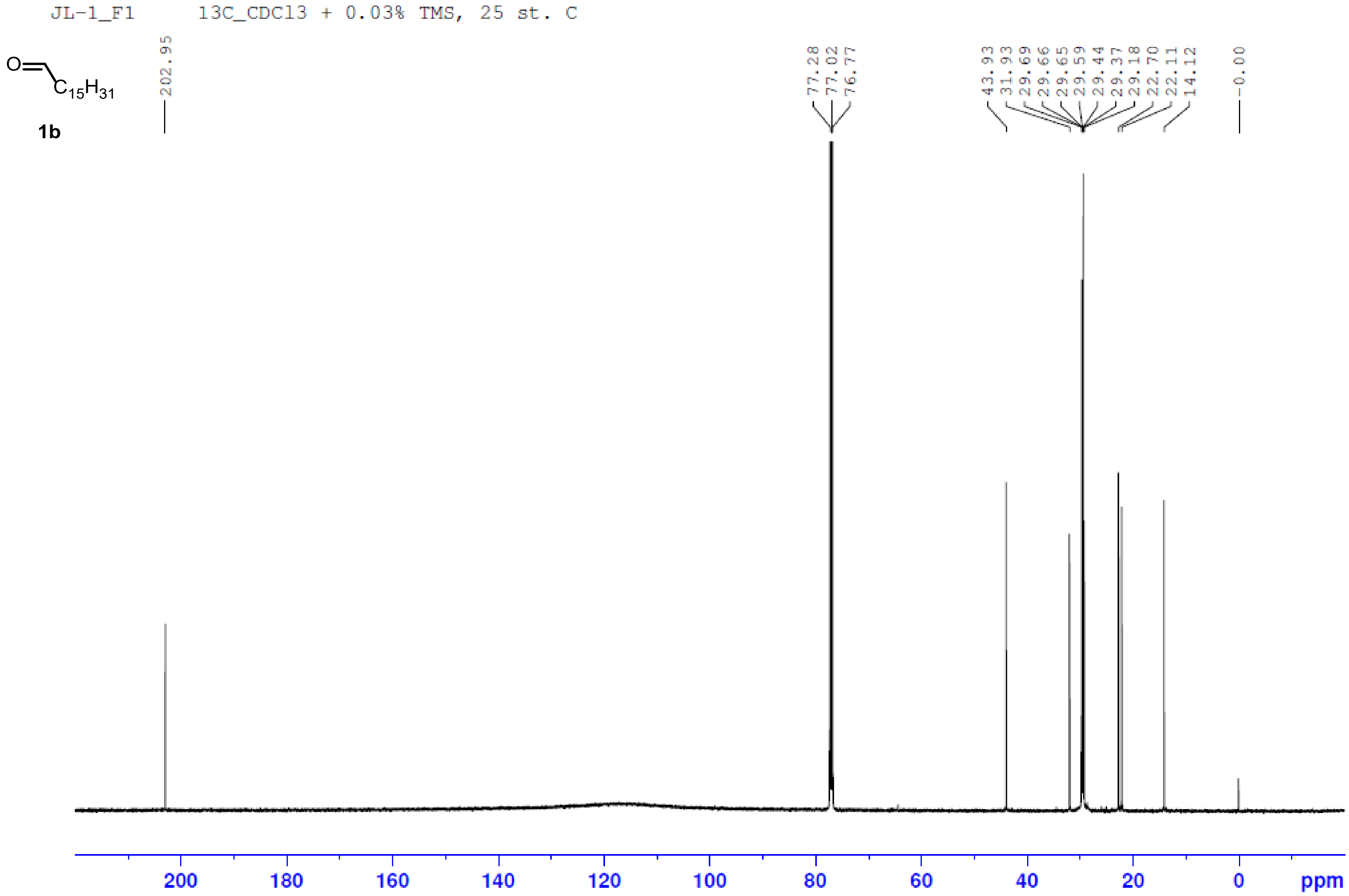
JL-1_F1 13C_CDCl3 + 0.03% TMS, 25 st. C
O
C15H31
202.95
77.28
77.02
76.77
43.93
31.93
29.69
29.66
29.65
29.59
29.44
29.37
29.18
22.70
22.11
14.12
-0.00
200
180
160
140
120
100
80
60
40
20
0
ppm

1b

DL-262F1F1P 1H_CDCl3 + 0.03% TMS, 25 st. C

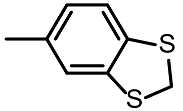

5-methylbenzo[*d*][1,3]dithiole

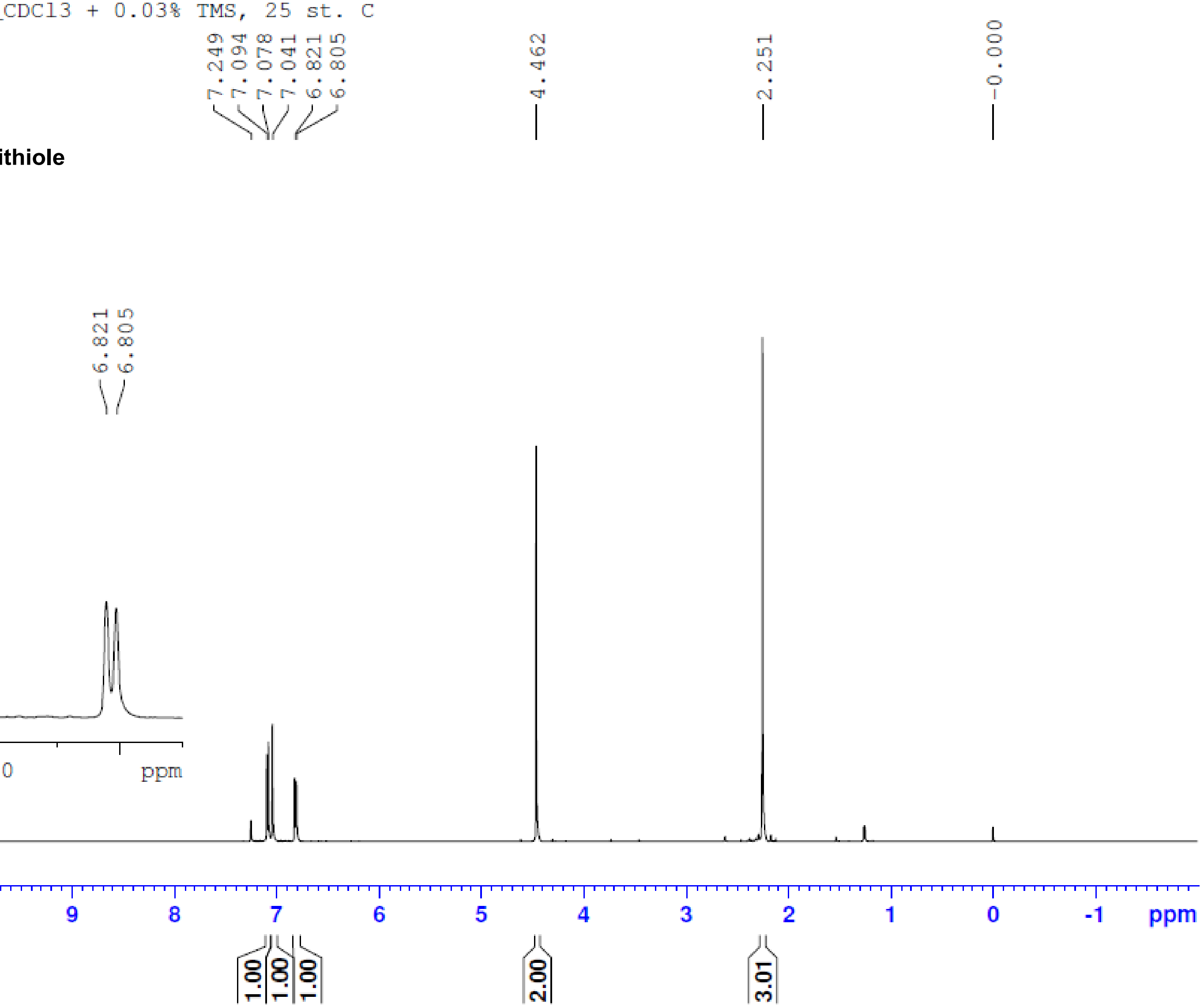

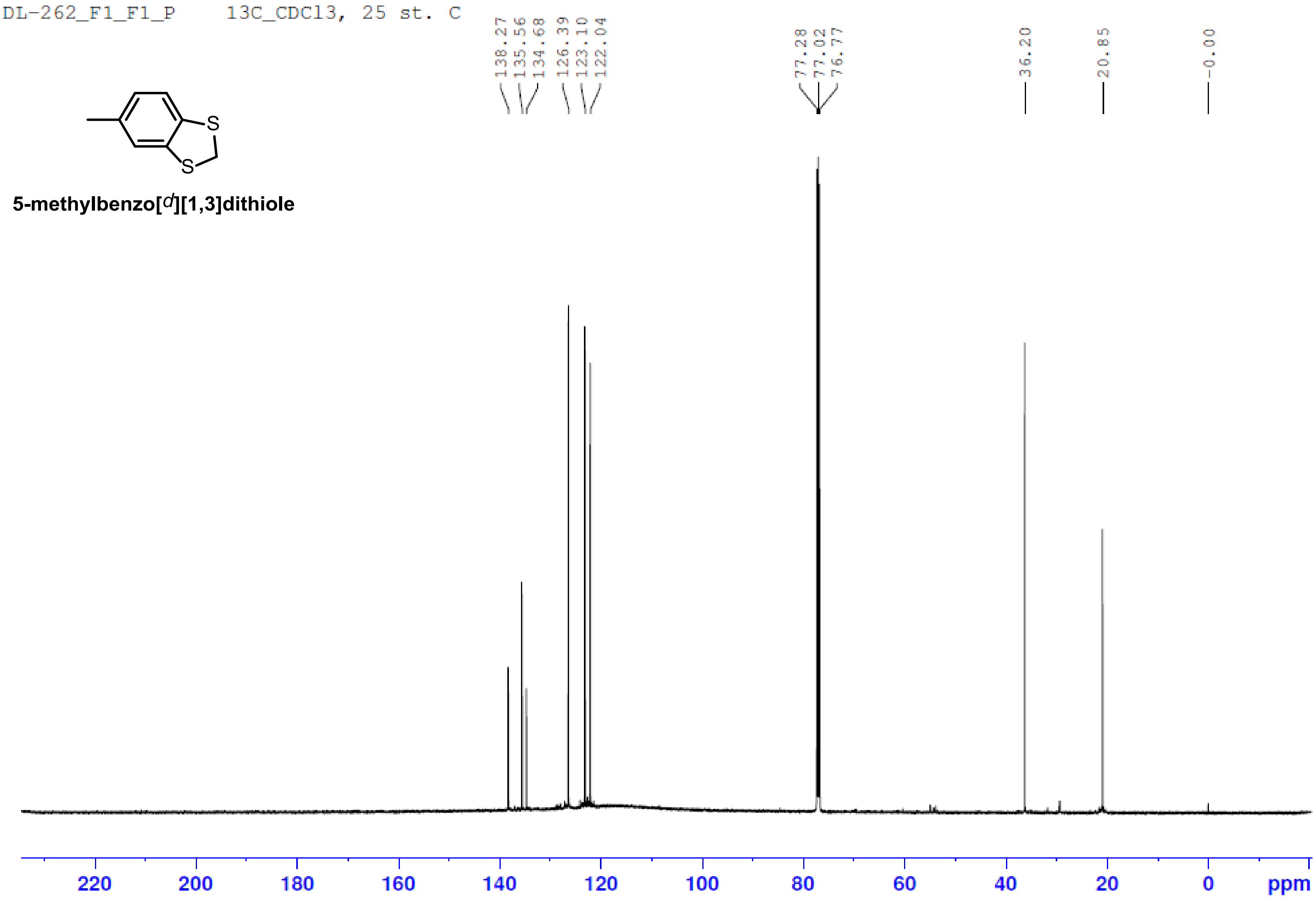
DL-262_F1_F1_P 13C_CDCl3, 25 st. C
138.27
135.56
134.68
126.39
123.10
122.04
77.28
77.02
76.77
36.20
20.85
-0.00
S
S
5-methylbenzo[d][1,3]dithiole
220
200
180
160
140
120
100
80
60
40
20
0
ppm

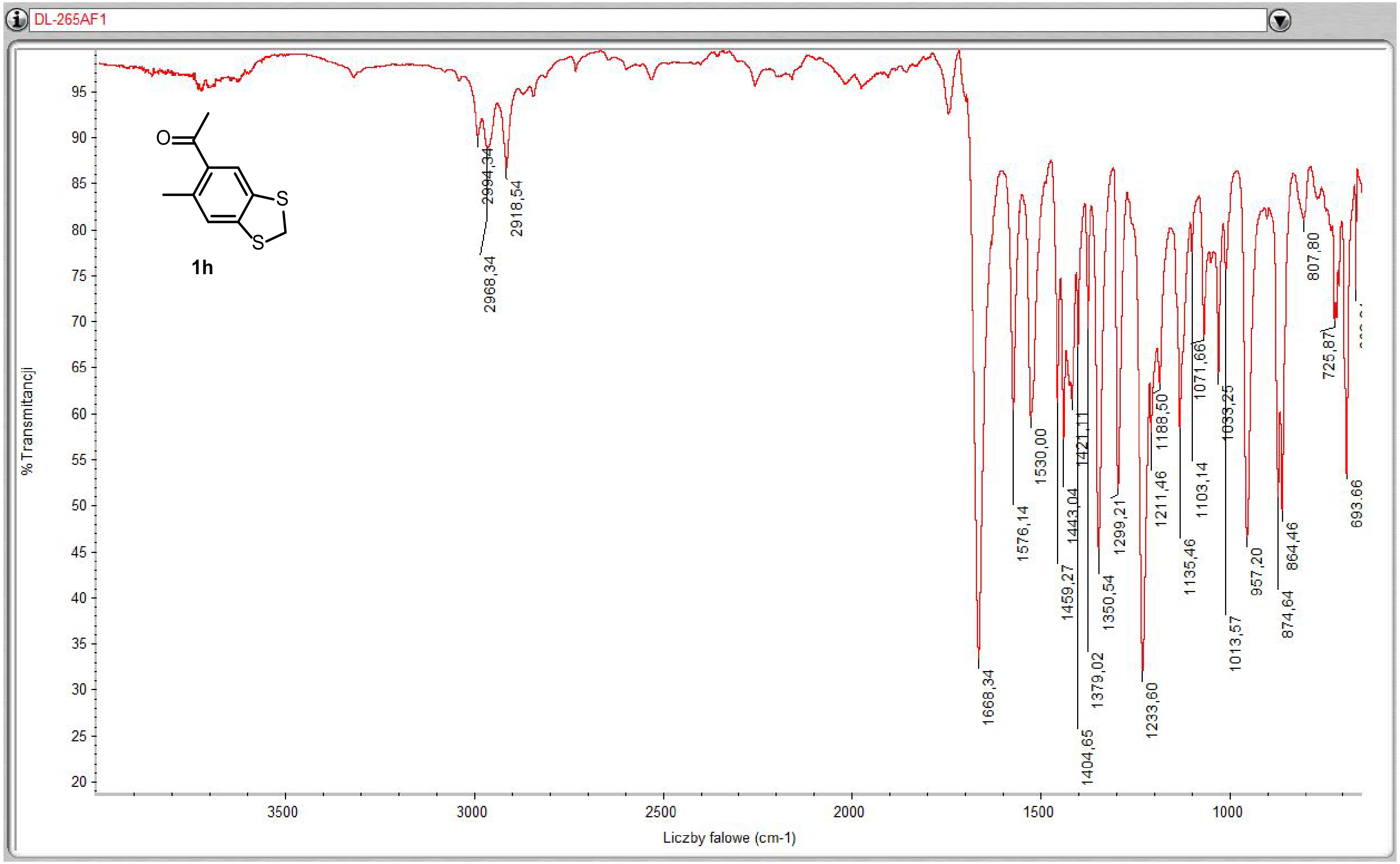

DL-265AF1
% Transmitancji
Liczby falowe (cm-1)
1h
2994,34
2968,34
2918,54
1668,34
1576,14
1530,00
1459,27
1443,04
1421,11
1404,65
1379,02
1350,54
1299,21
1233,60
1211,46
1188,50
1135,46
1103,14
1071,66
1033,25
1013,57
957,20
874,64
864,46
807,80
725,87
693,66

DL-265_A_F1 1H_CDCl3 + 0.03% TMS, 25 st. C

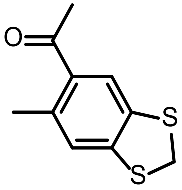

1h

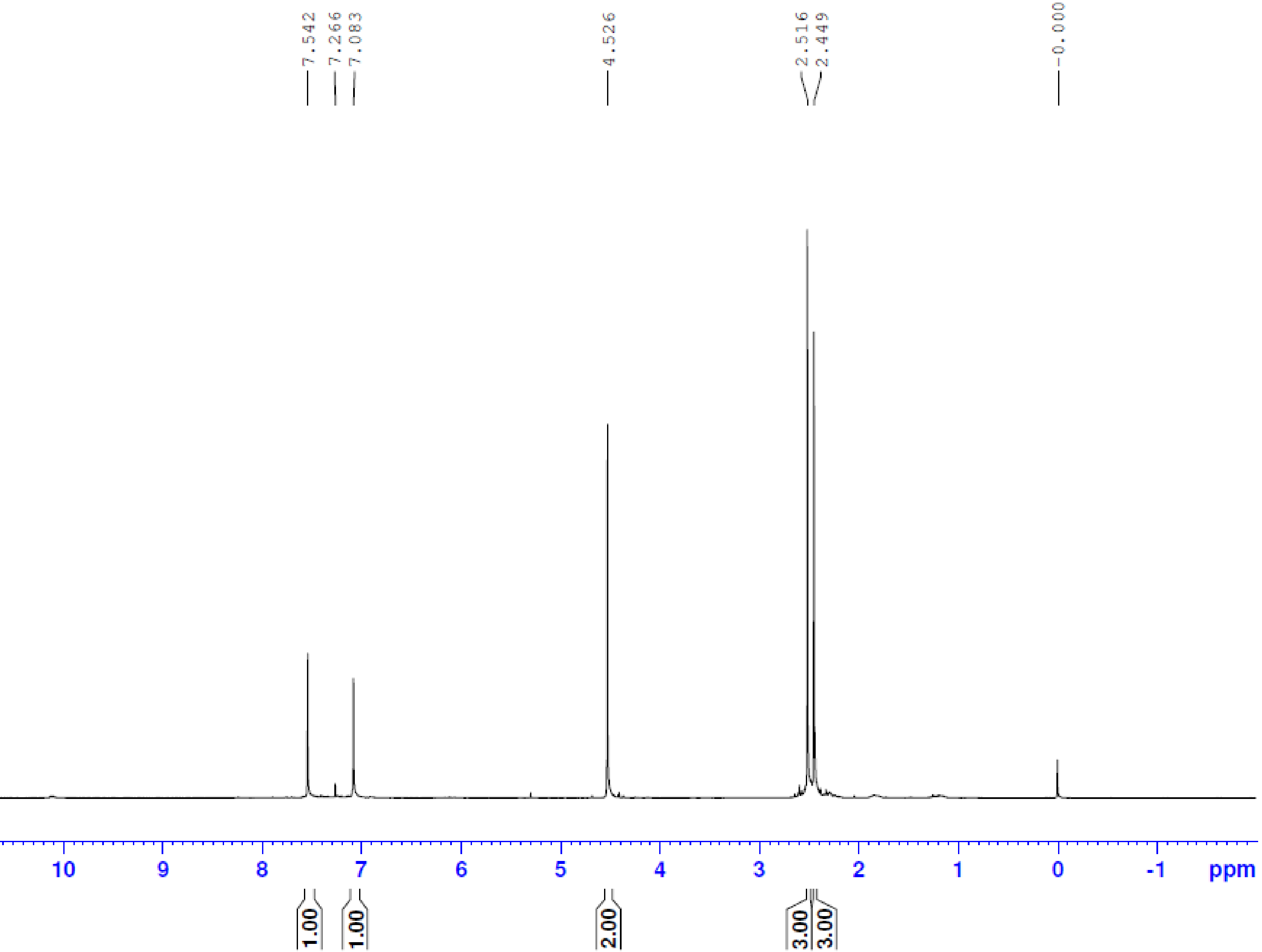

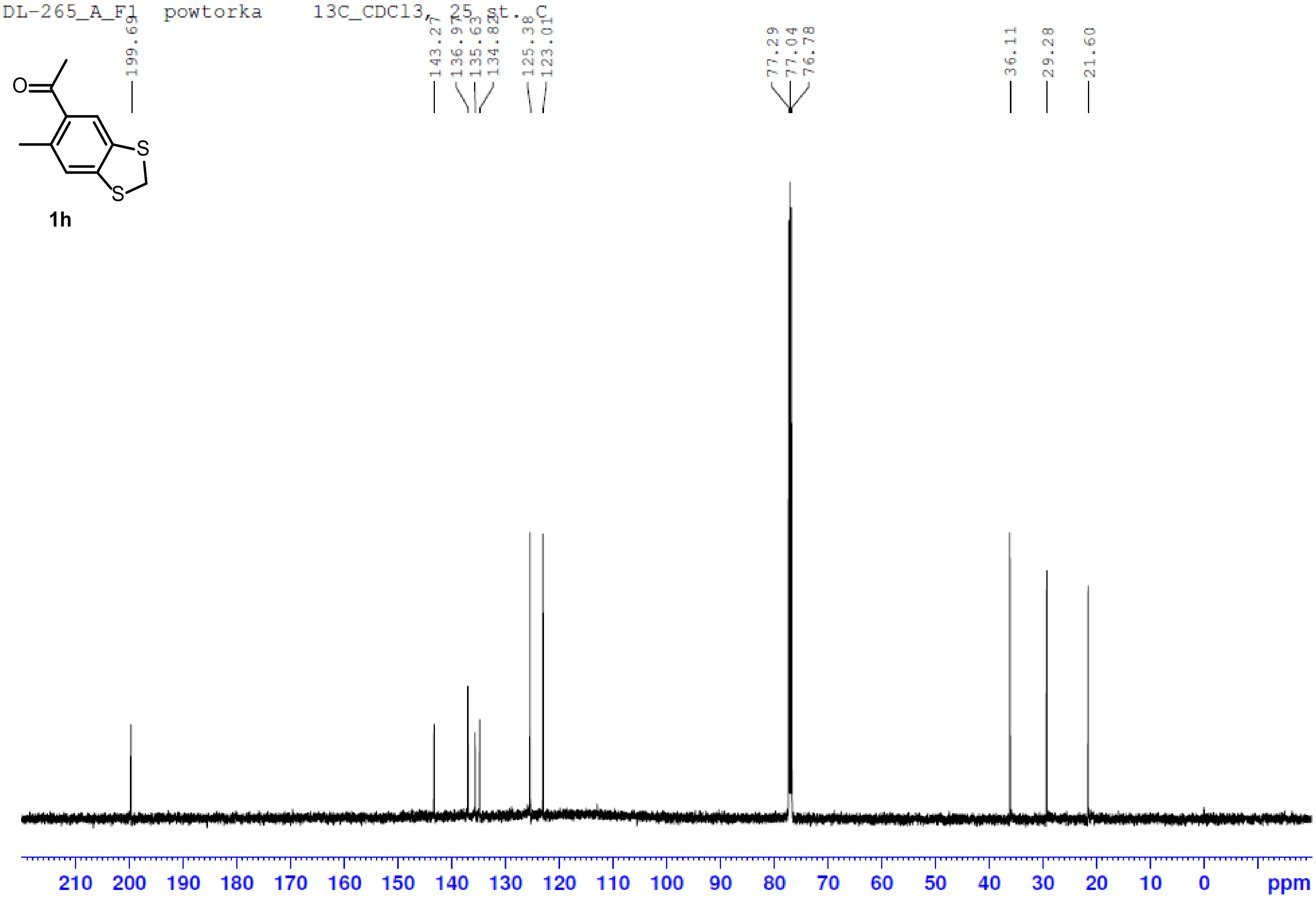
DL-265_A_F1 powtorka 13C_CDCl3, 25 st. C
199.69
143.27
136.97
135.63
134.82
125.38
123.01
77.29
77.04
76.78
36.11
29.28
21.60
O
S
S
1h
210 200 190 180 170 160 150 140 130 120 110 100 90 80 70 60 50 40 30 20 10 0 ppm

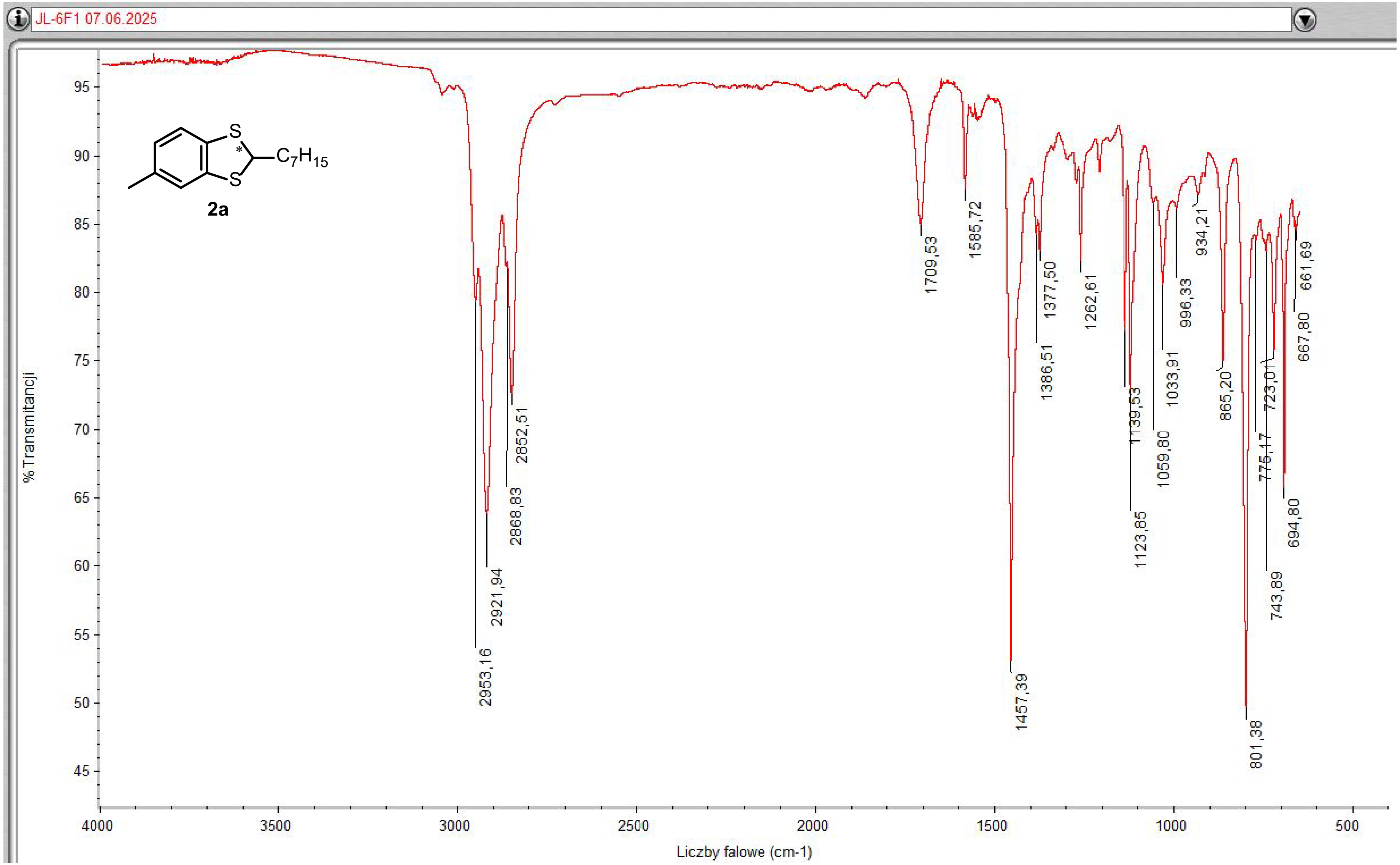
JL-6F1 07.06.2025
C_7H_{15}
2a
% Transmitancji
Liczby falowe (cm-1)
2953,16
2921,94
2868,83
2852,51
1709,53
1585,72
1457,39
1386,51
1377,50
1262,61
1139,53
1123,85
1059,80
1033,91
996,33
934,21
865,20
801,38
775,17
743,89
723,01
694,80
667,80
661,69

JL-6_F1 1H_CDCl3 + 0.03% TMS, 25 st. C

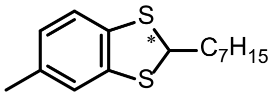

2a

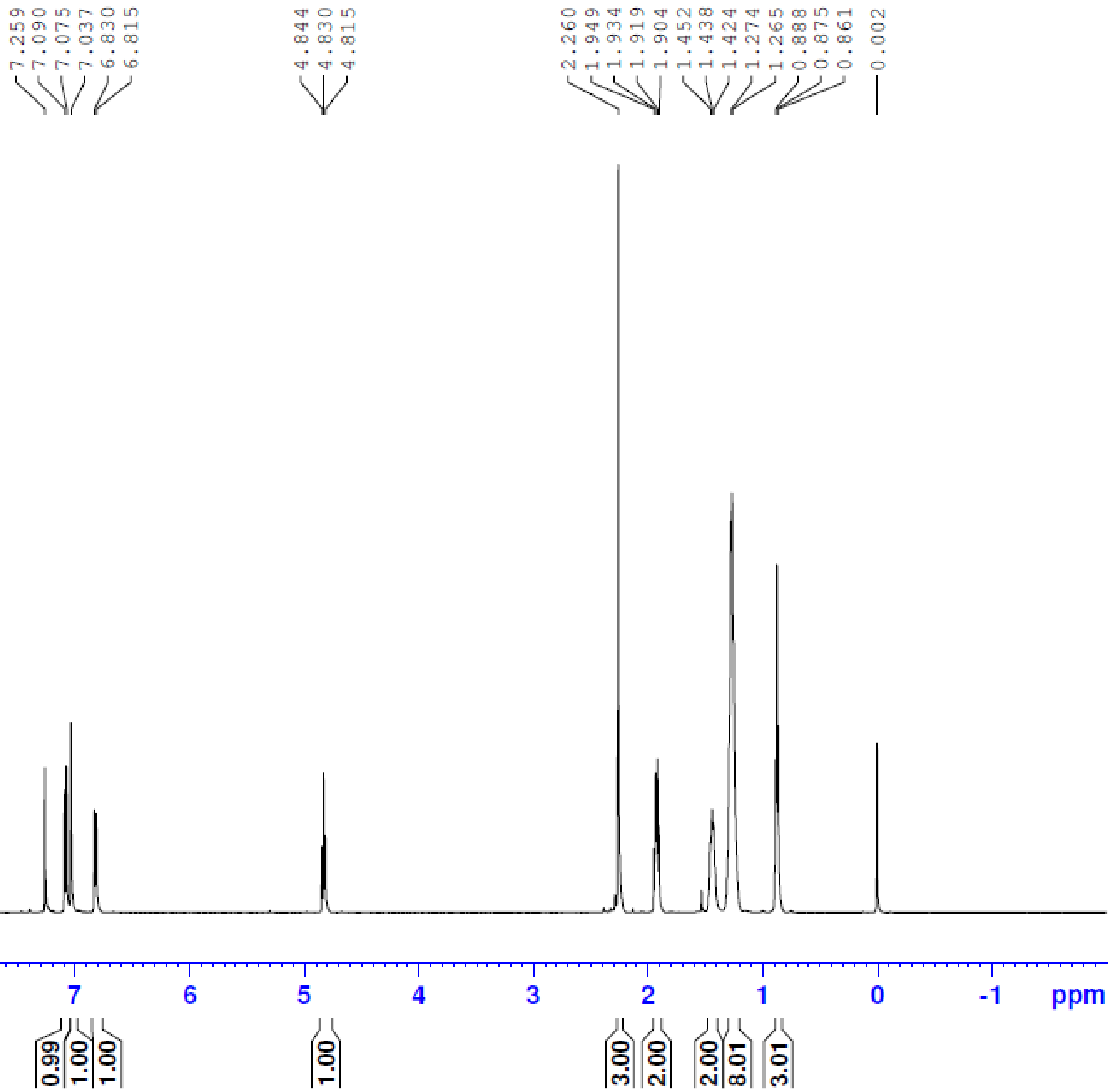

JL-6_F1 13C_CDCl3, 25 st. C

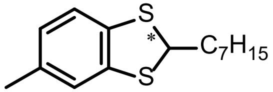

2a

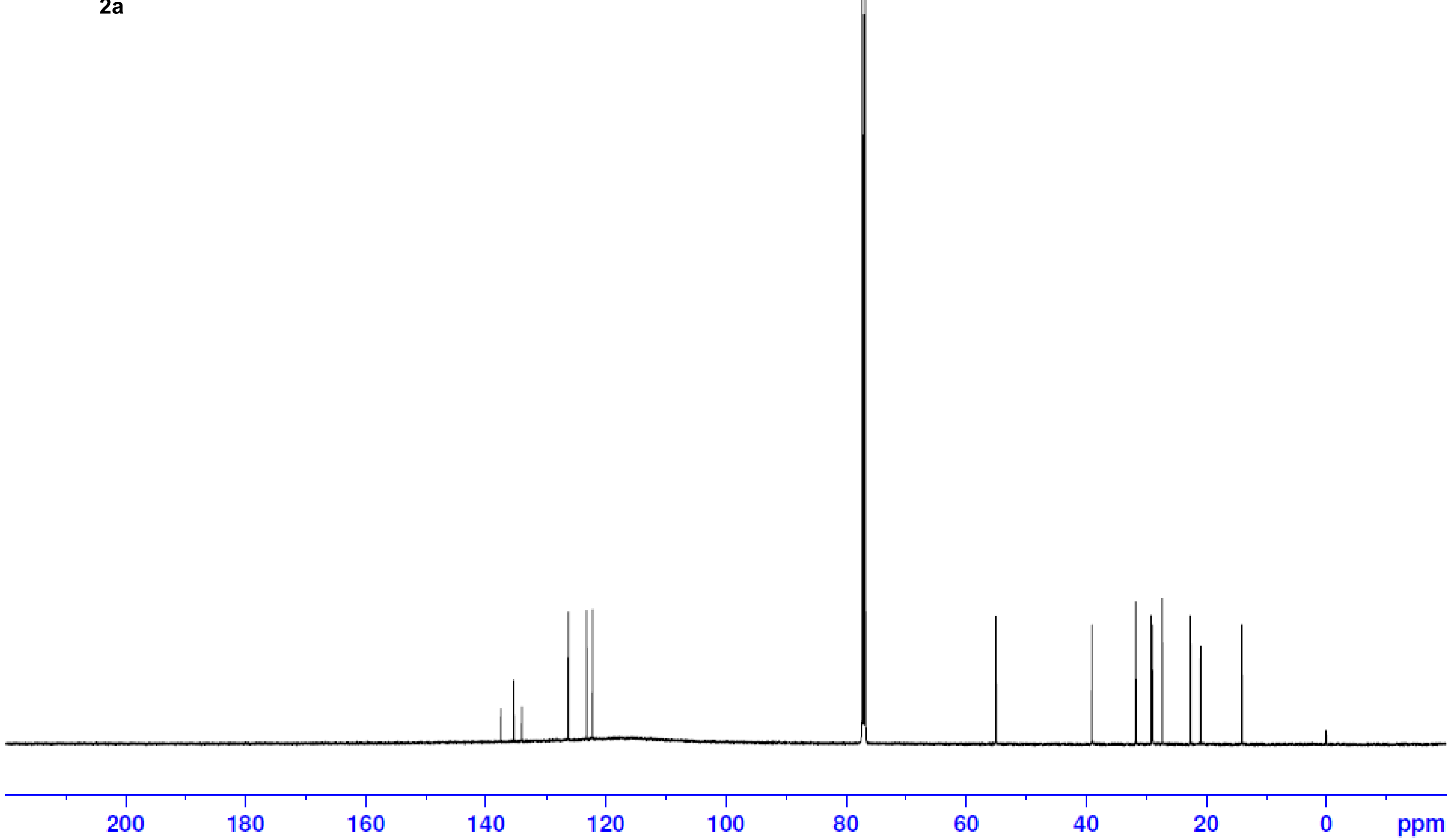

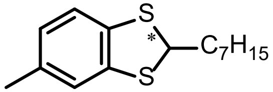
S
S
*
C7H15

2a

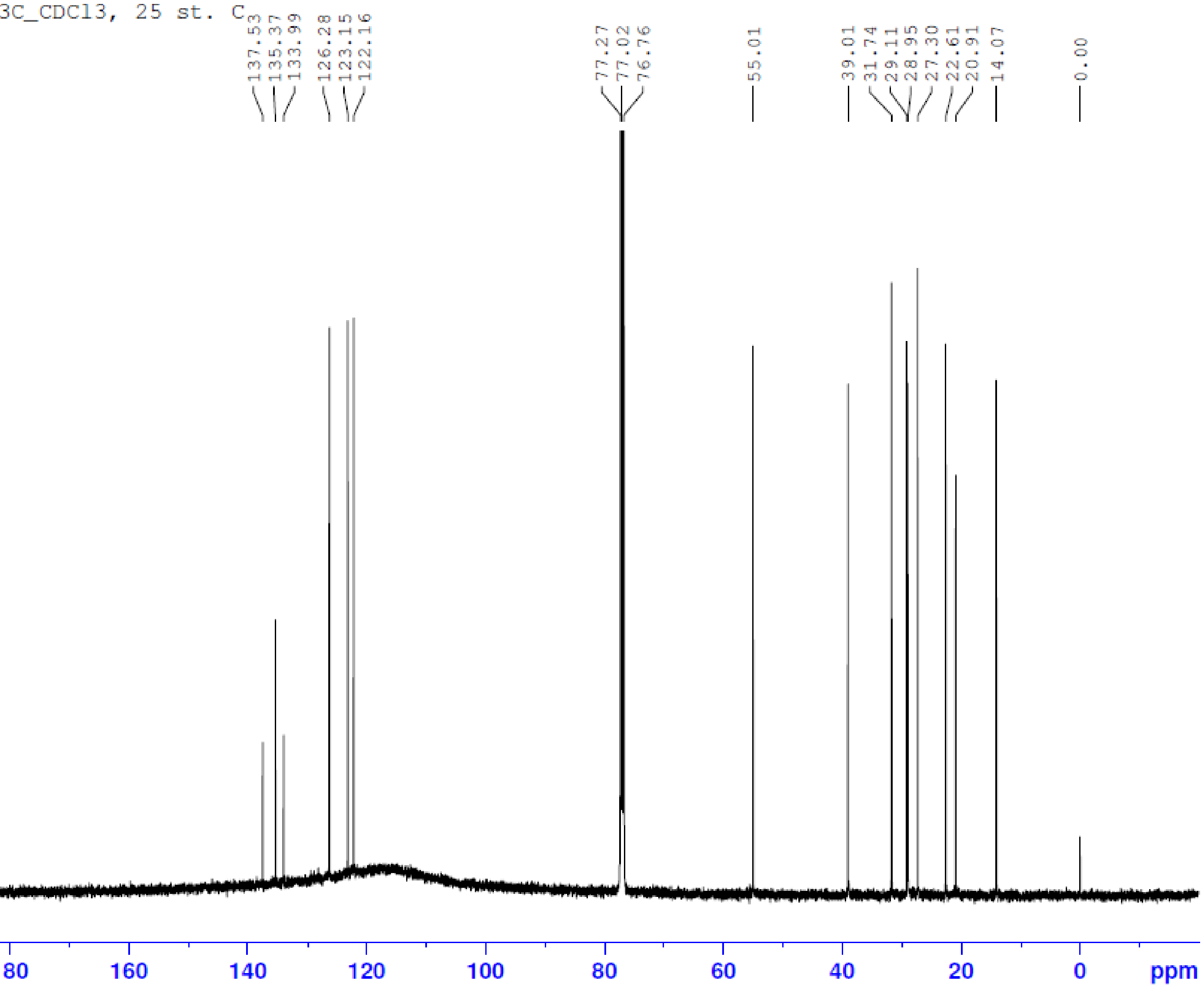
JL-6_F1
13C_CDCl3, 25 st. C
137.53
135.37
133.99
126.28
123.15
122.16
77.27
77.02
76.76
55.01
39.01
31.74
29.11
28.95
27.30
22.61
20.91
14.07
0.00
200
180
160
140
120
100
80
60
40
20
0
ppm

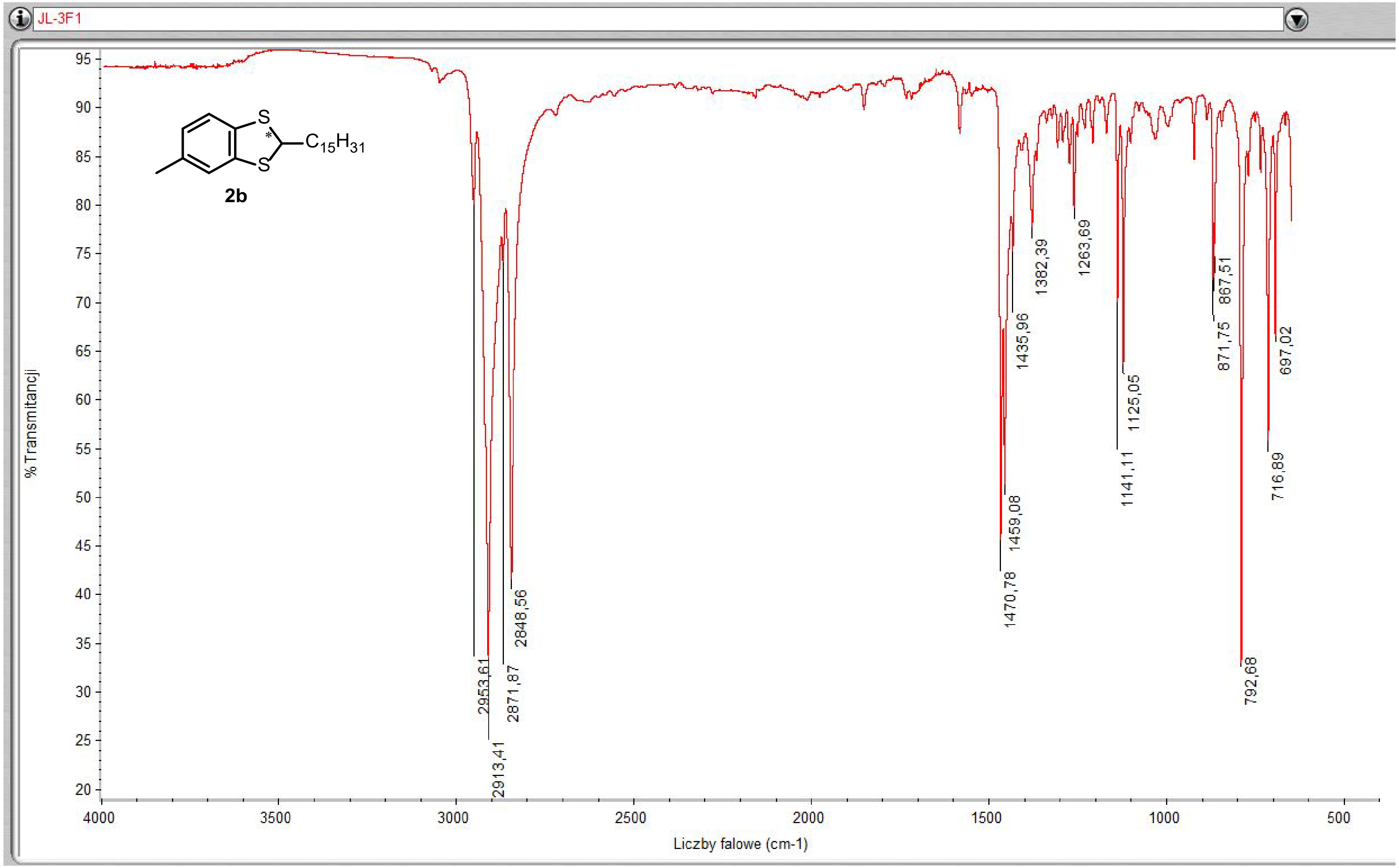
JL-3F1
%Transmitancji
Liczby falowe (cm-1)
2b
2953,61
2913,41
2871,87
2848,56
1470,78
1459,08
1435,96
1382,39
1263,69
1141,11
1125,05
871,75
867,51
792,68
716,89
697,02

JL-3_F1 1H_CDCl3 + 0.03% TMS, 25 st. C

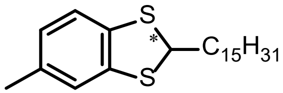

2b

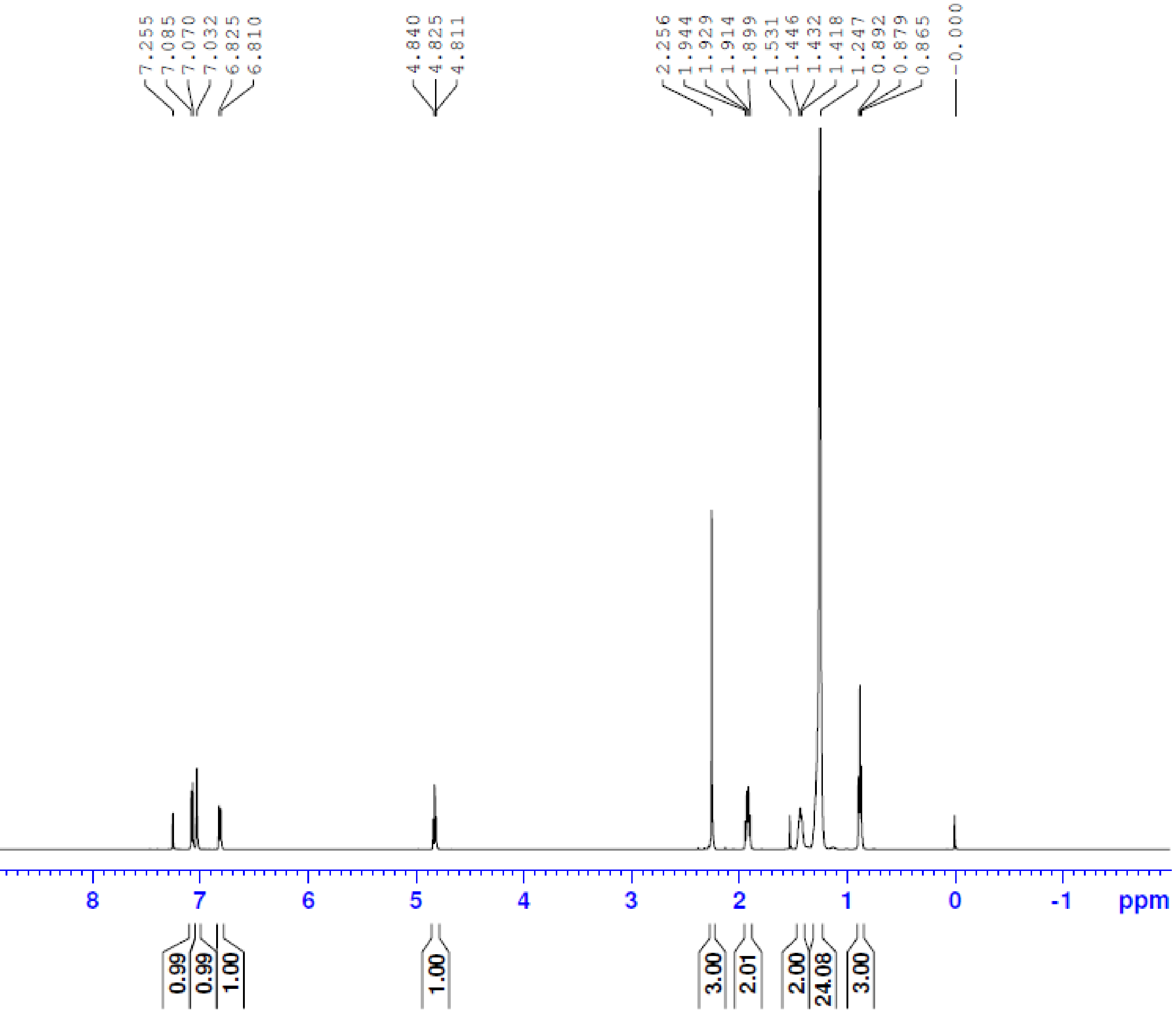

JL-3_F1 13C_CDCl3 + 0.03% TMS, 25 st. C

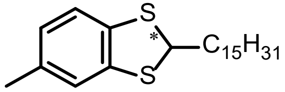

2b

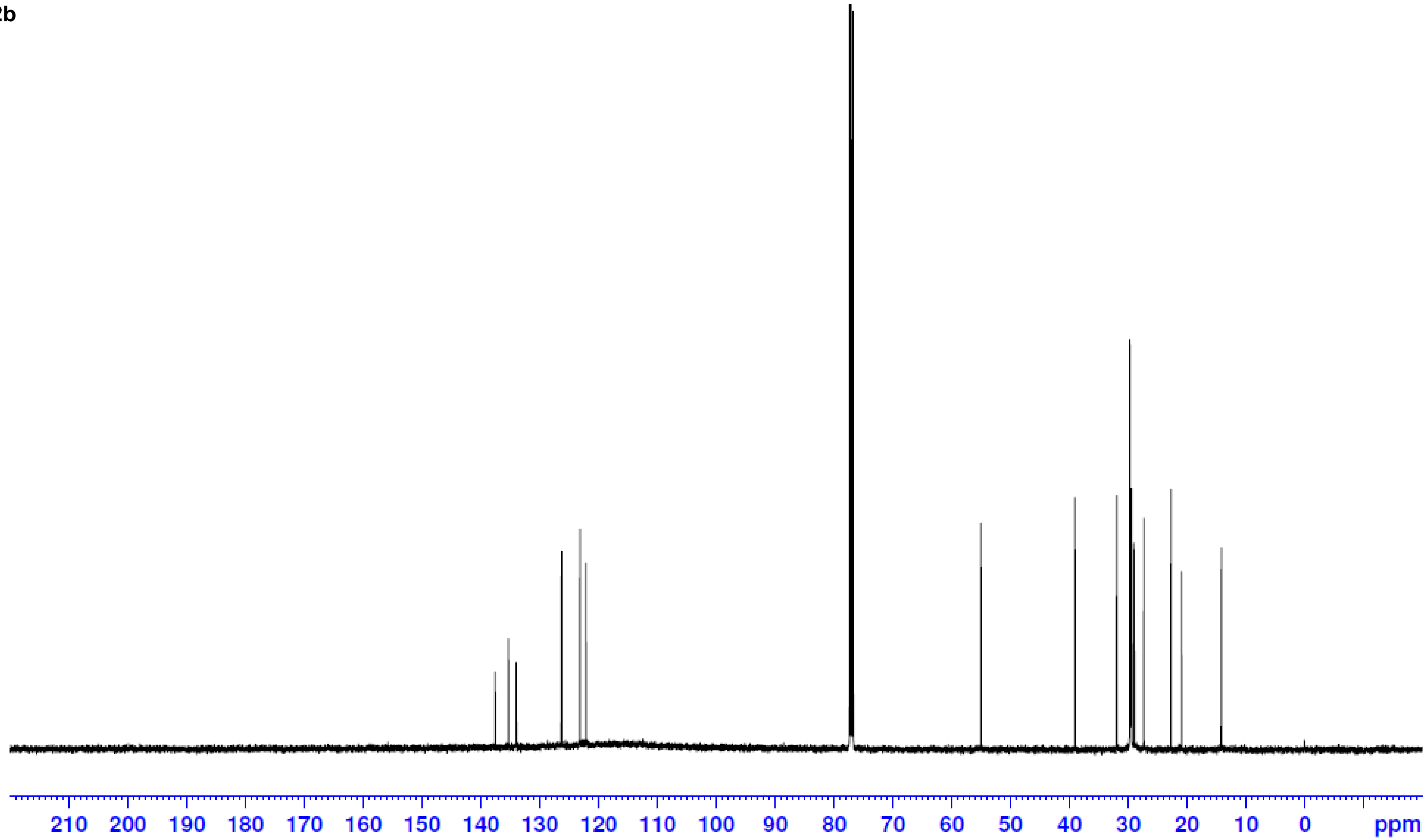

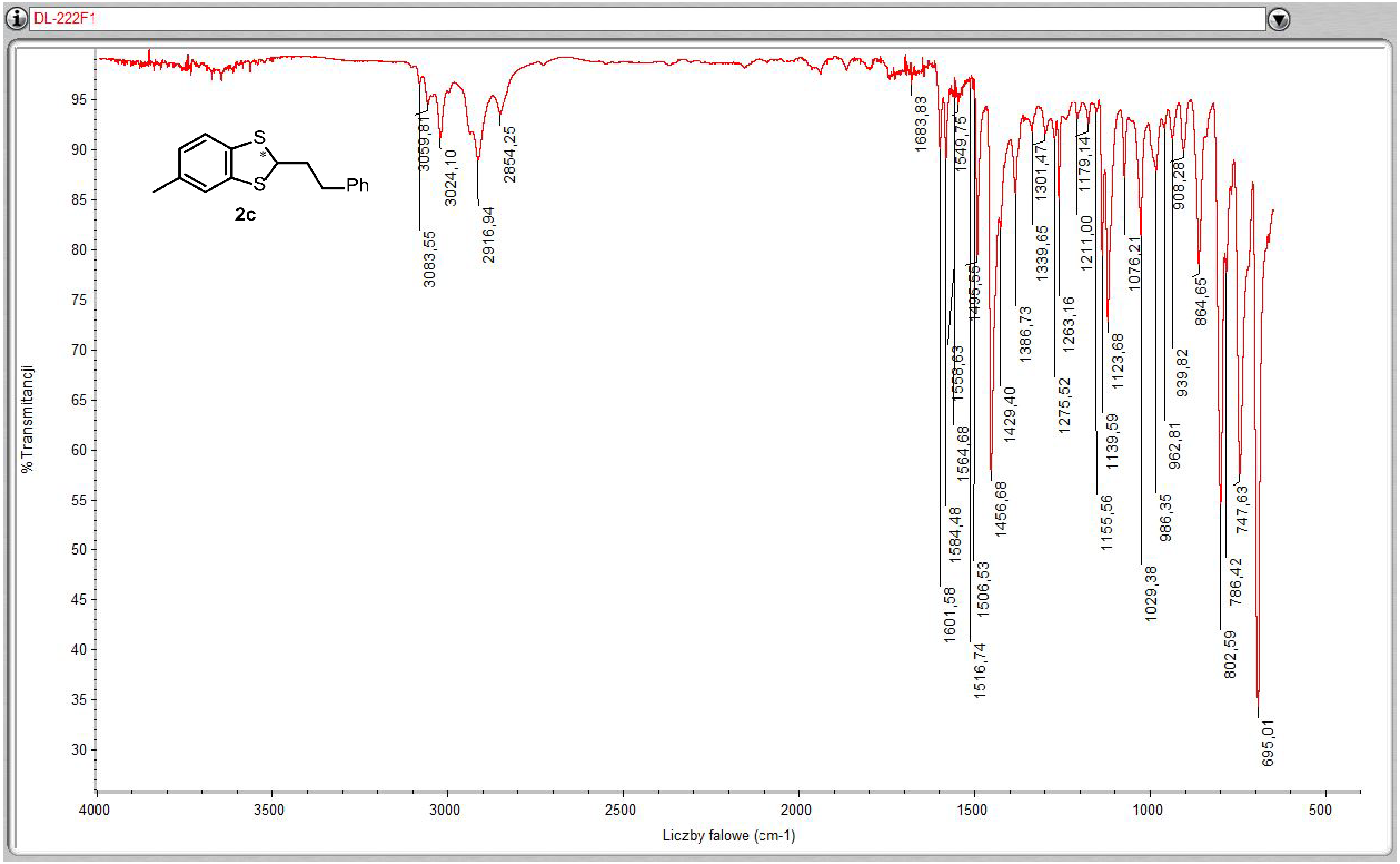

DL-222F1
2c
Ph
S
S
%Transmitancji
Liczby falowe (cm-1)
3083,55
3059,81
3024,10
2916,94
2854,25
1683,83
1601,58
1584,48
1564,68
1558,63
1549,75
1516,74
1506,53
1495,55
1456,68
1429,40
1386,73
1339,65
1301,47
1275,52
1263,16
1211,00
1179,14
1155,56
1139,59
1123,68
1076,21
1029,38
986,35
962,81
939,82
908,28
864,65
802,59
786,42
747,63
695,01

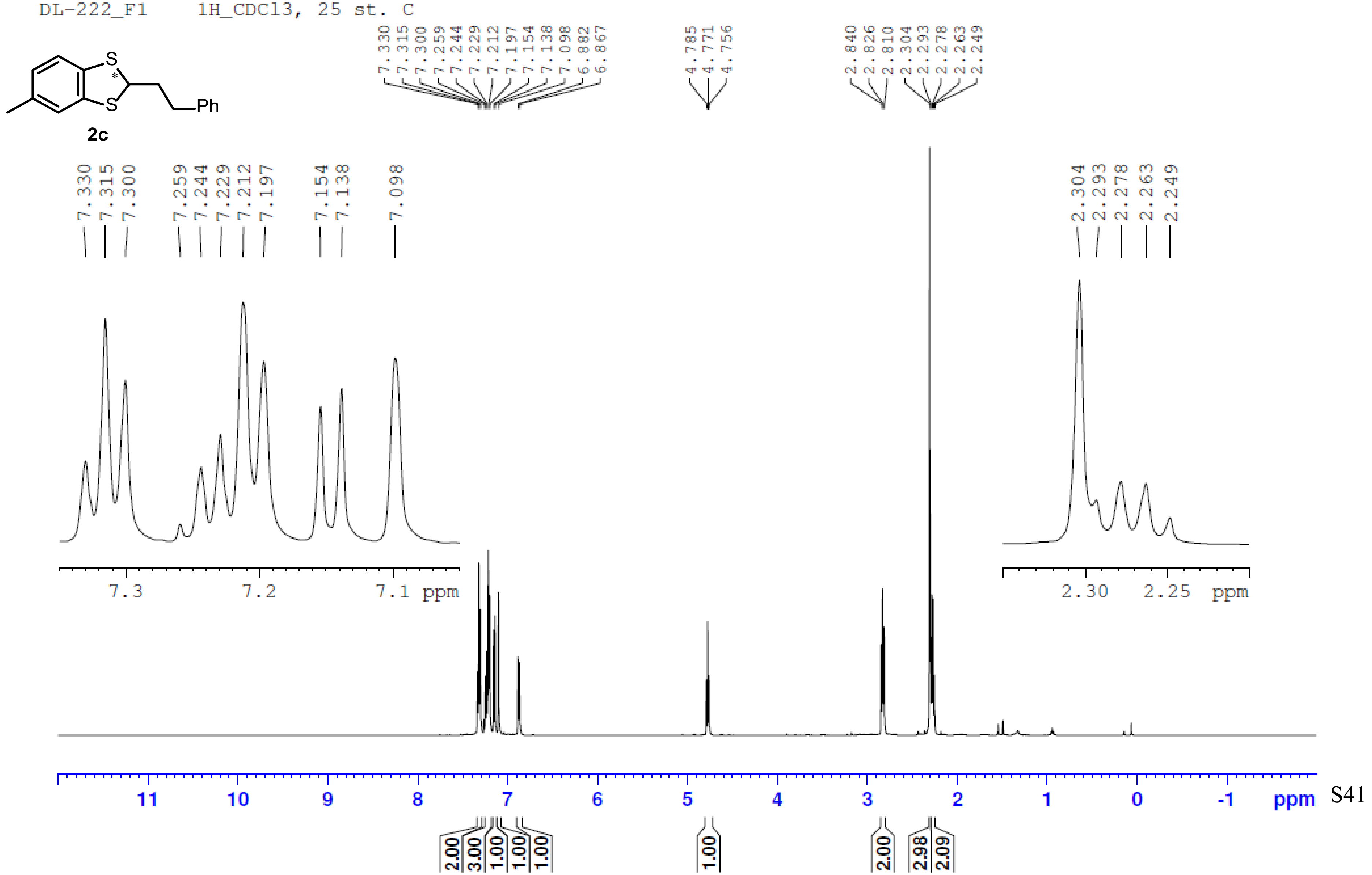
DL-222_F1 1H_CDCl3, 25 st. C
2c
Ph
ppm
S41

DL-222_F1 13C_CDCl3 + 0,03% TMS, 25 st. C

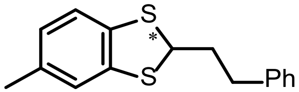

2c

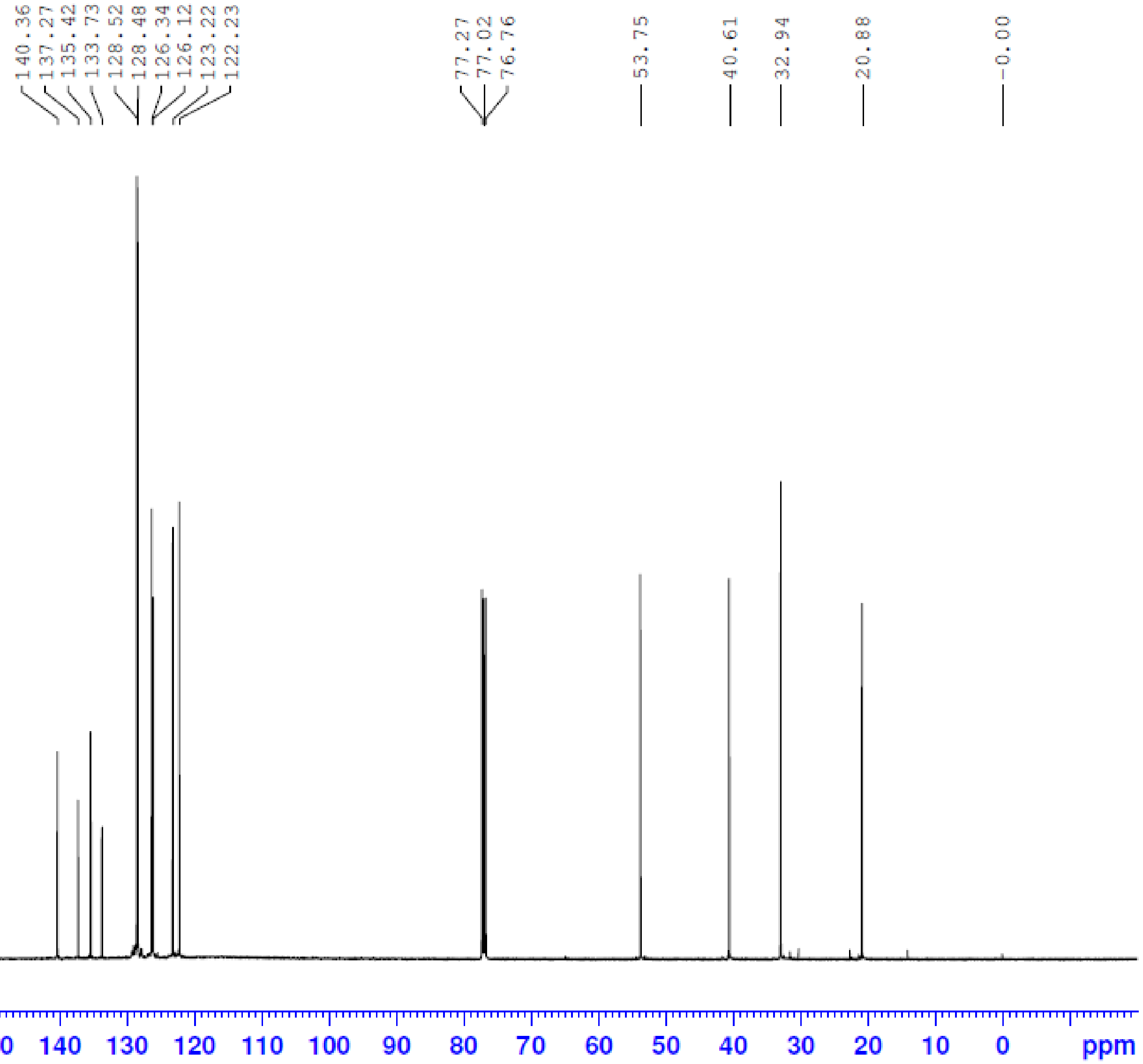

DL-235_F1 1H_CDCl3 + 0.03% TMS, 25 st. C

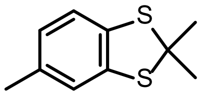

2d

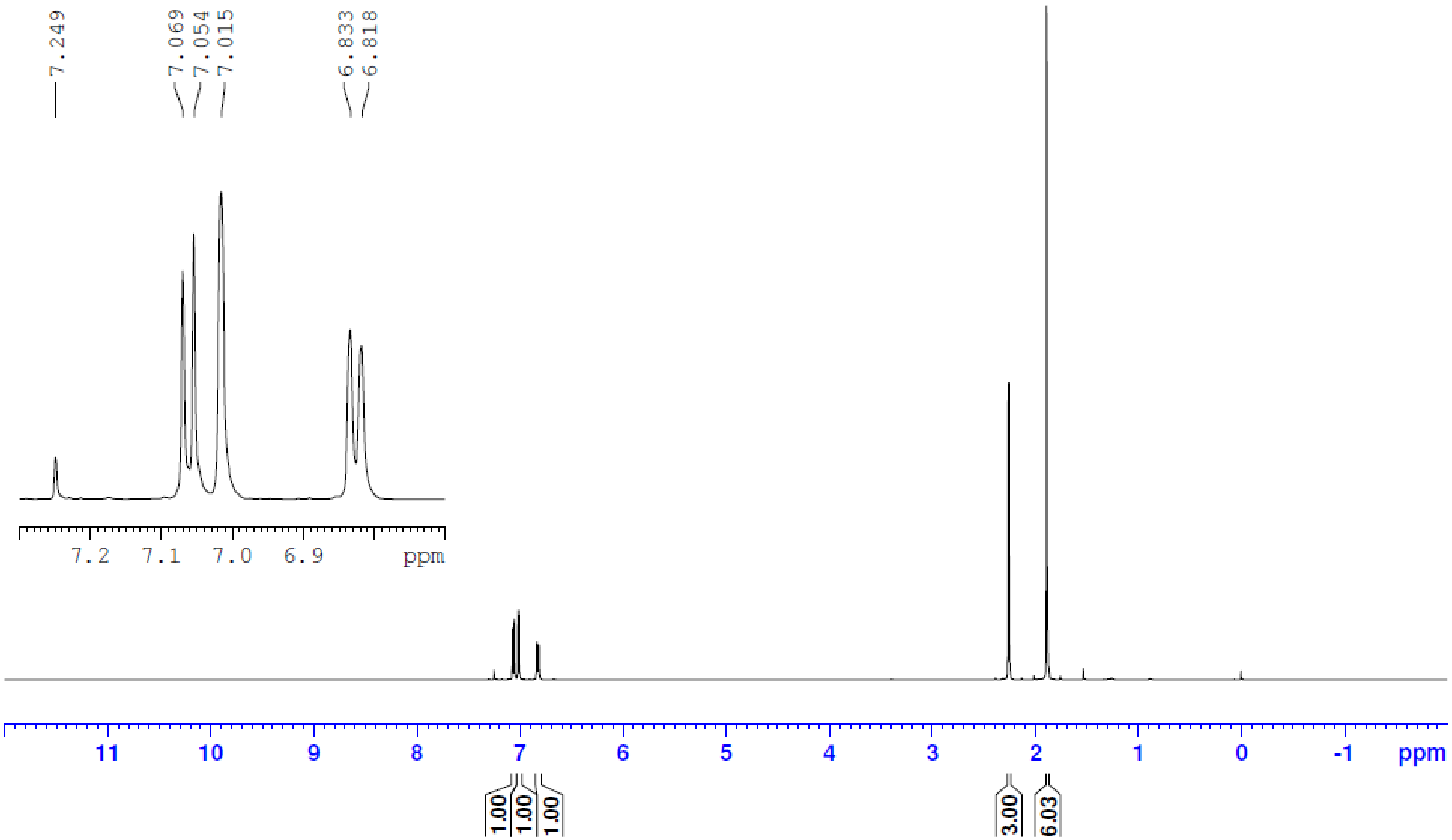

DL-235_F1 13C_CDCl3, 25 st. C

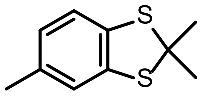

2d

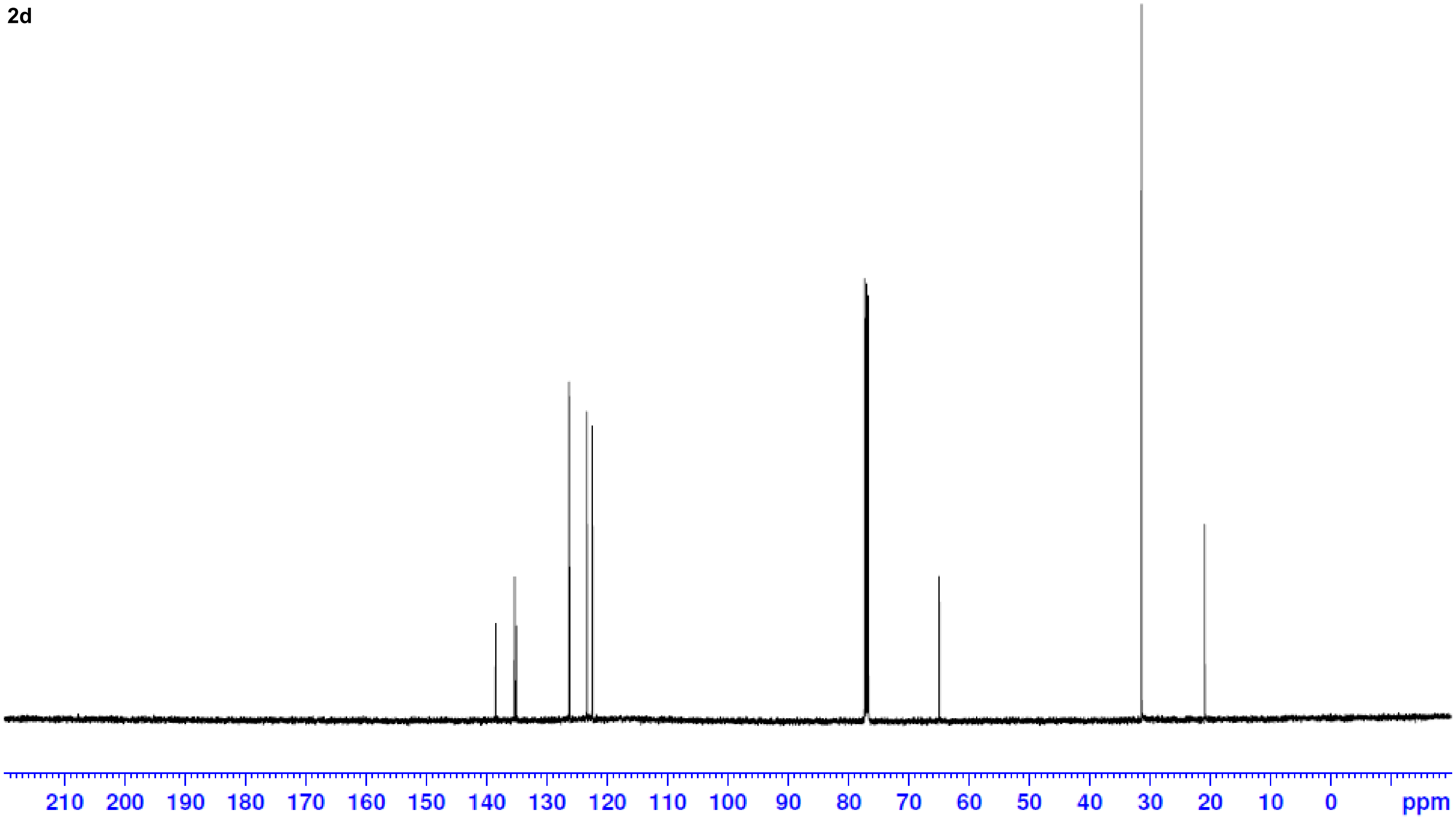

DL-212_F1 1H_CDCl3 + 0.03% TMS, 25 st. C

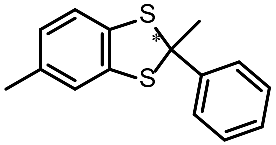

2e

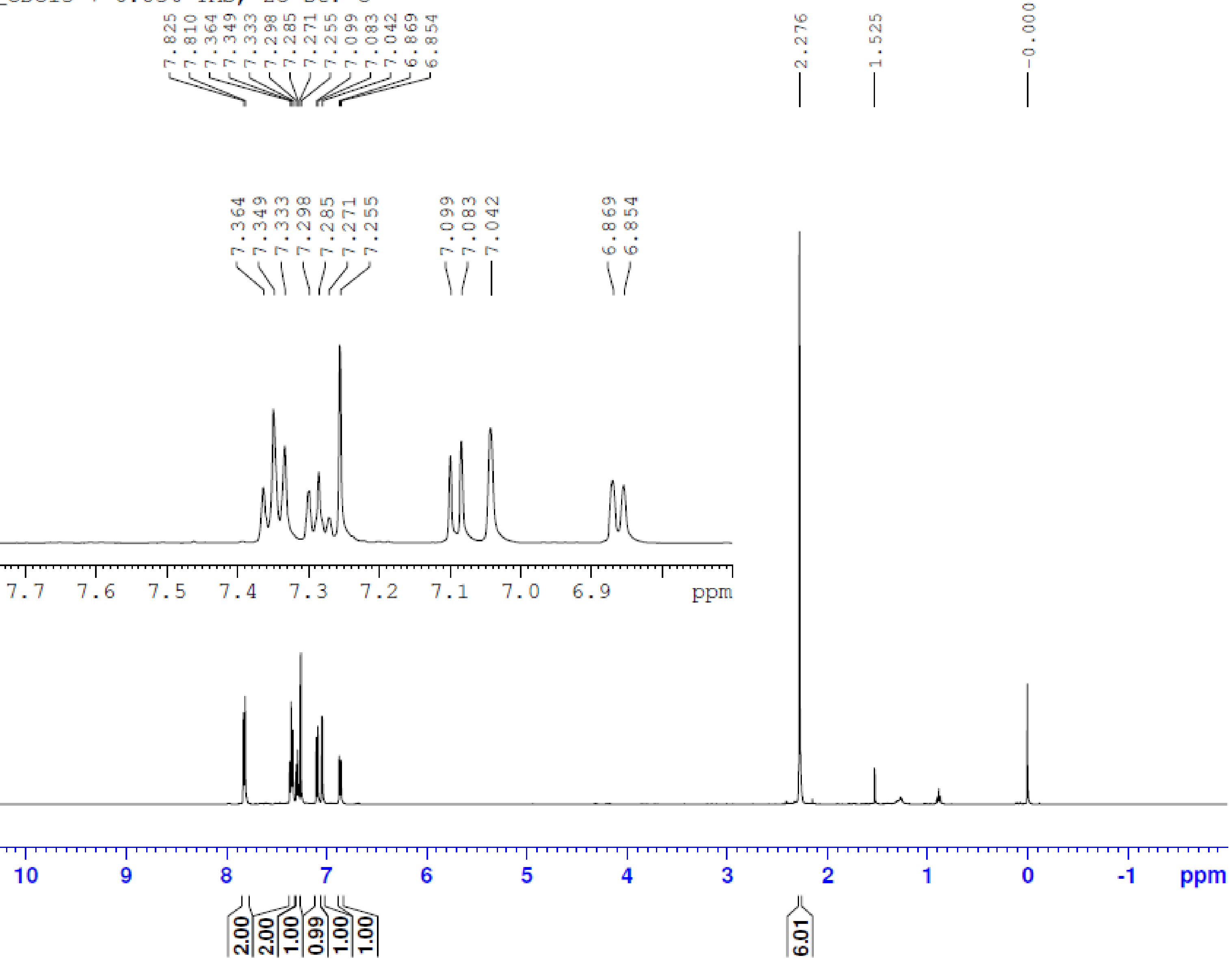

DL-210_F1 13C_CDCl3, 25 st. C

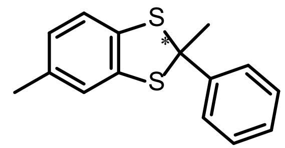

2e

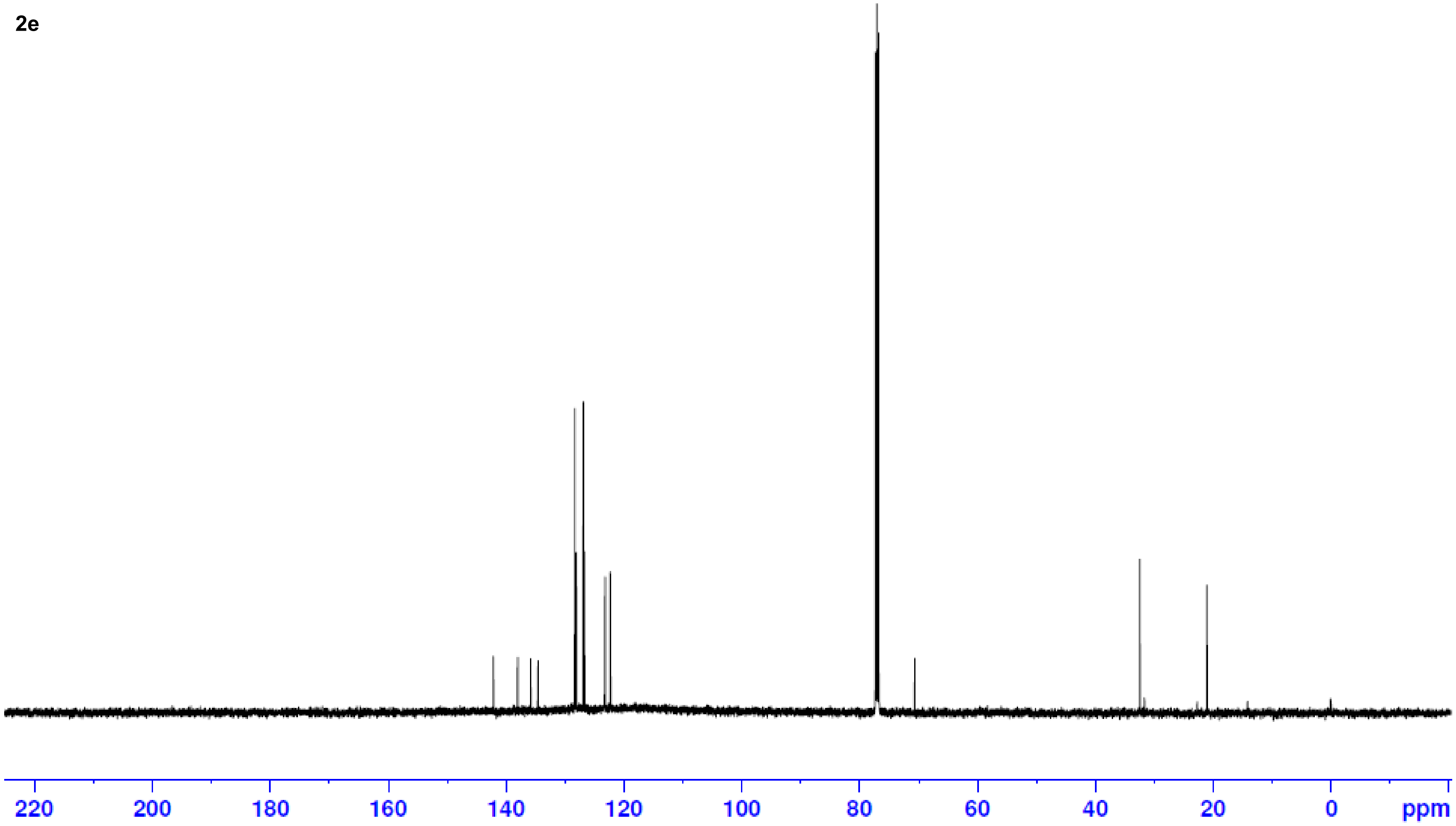

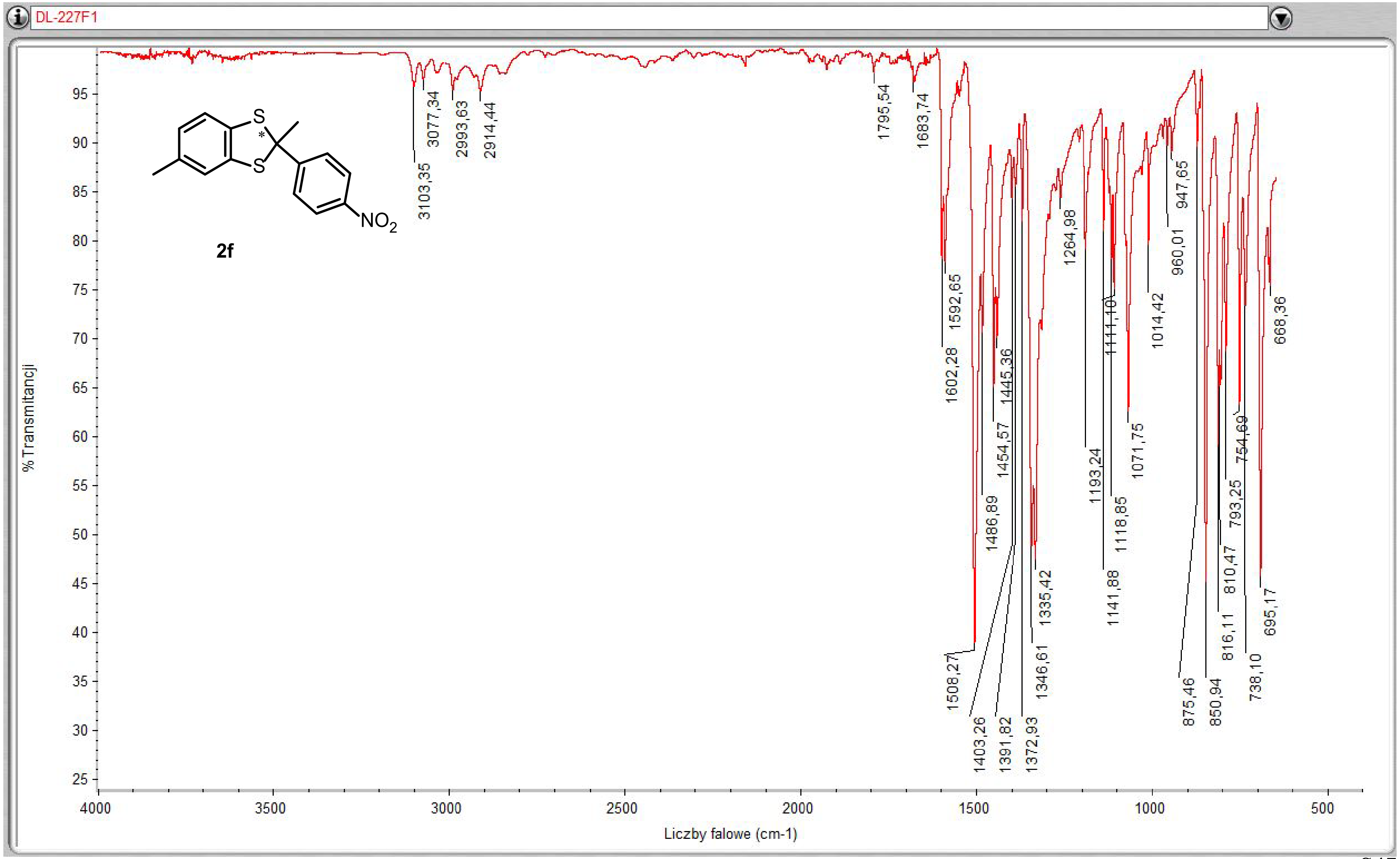
DL-227F1
%Transmitancji
Liczby falowe (cm-1)
2f
NO2
3103,35
3077,34
2993,63
2914,44
1795,54
1683,74
1602,28
1592,65
1508,27
1486,89
1454,57
1445,36
1403,26
1391,82
1372,93
1346,61
1335,42
1264,98
1193,24
1141,88
1118,85
1111,10
1071,75
1014,42
960,01
947,65
875,46
850,94
816,11
810,47
793,25
754,69
738,10
695,17
668,36

DL-227_F1 1H_CDCl3 +0.03% TMS, 25 st. C

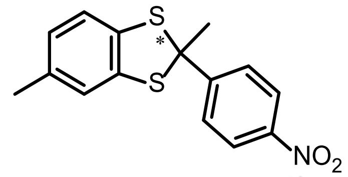

2f

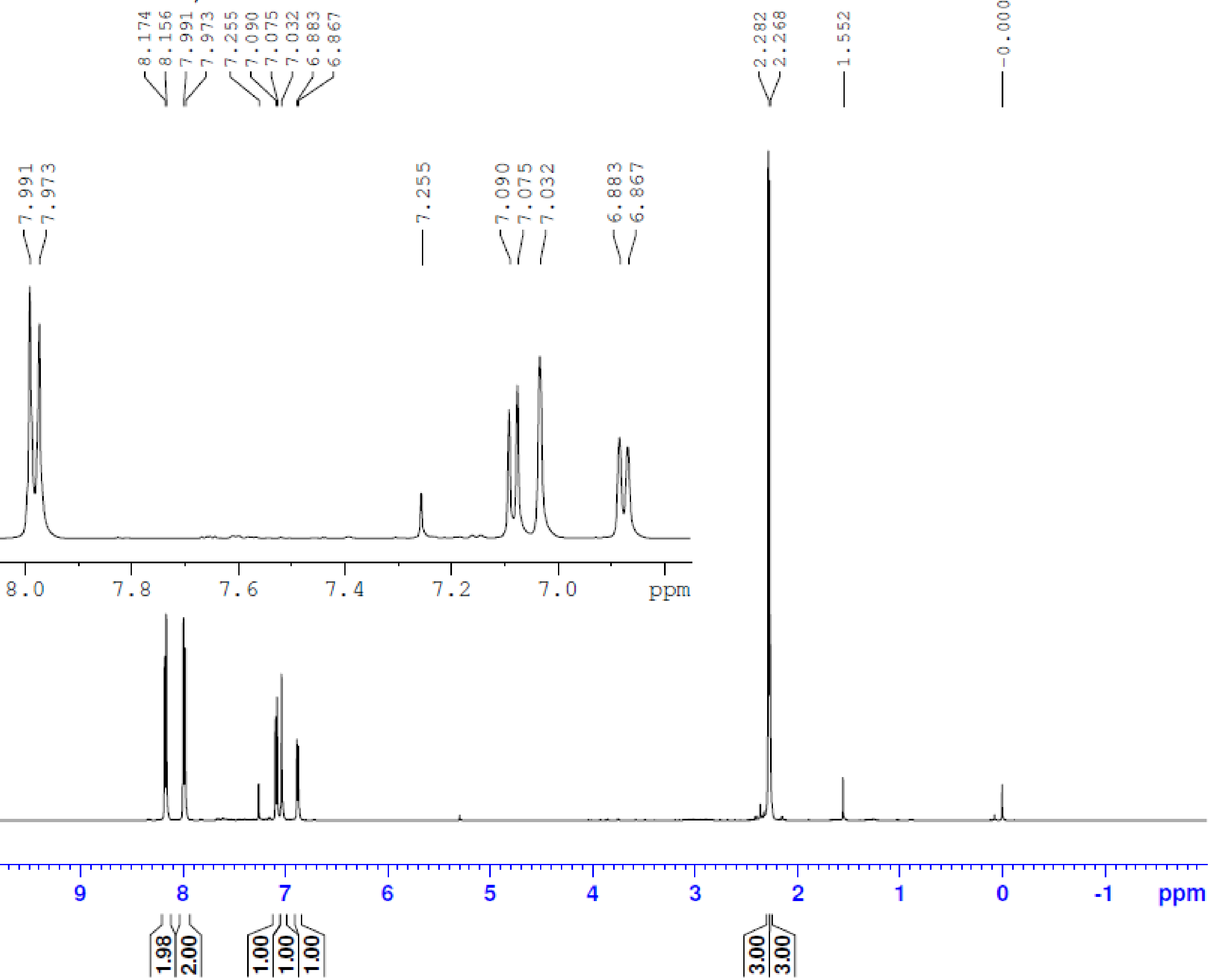

DL-227_F1   13C_CDCl3 + 0.03% TMS, 25 st. C

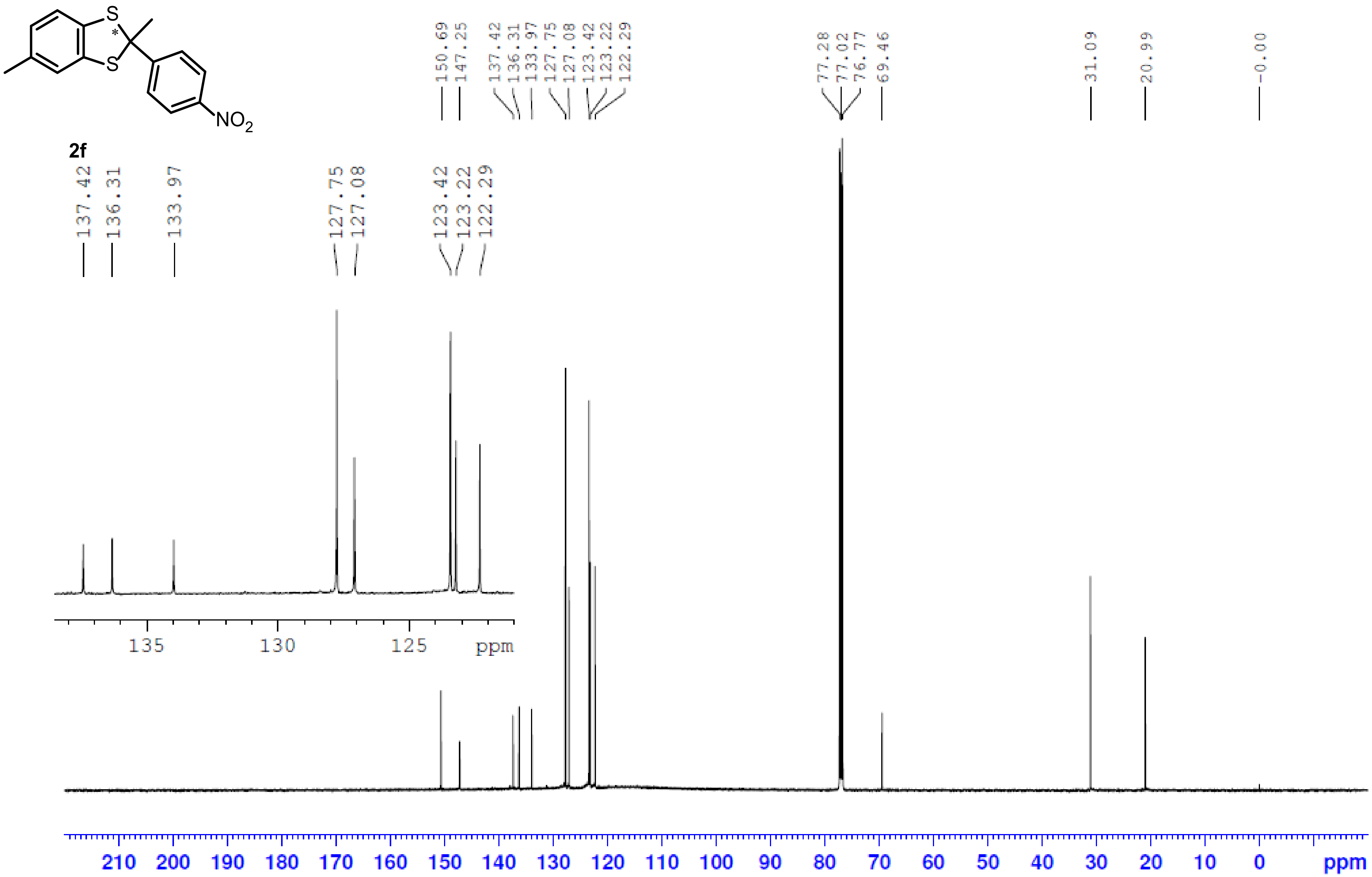

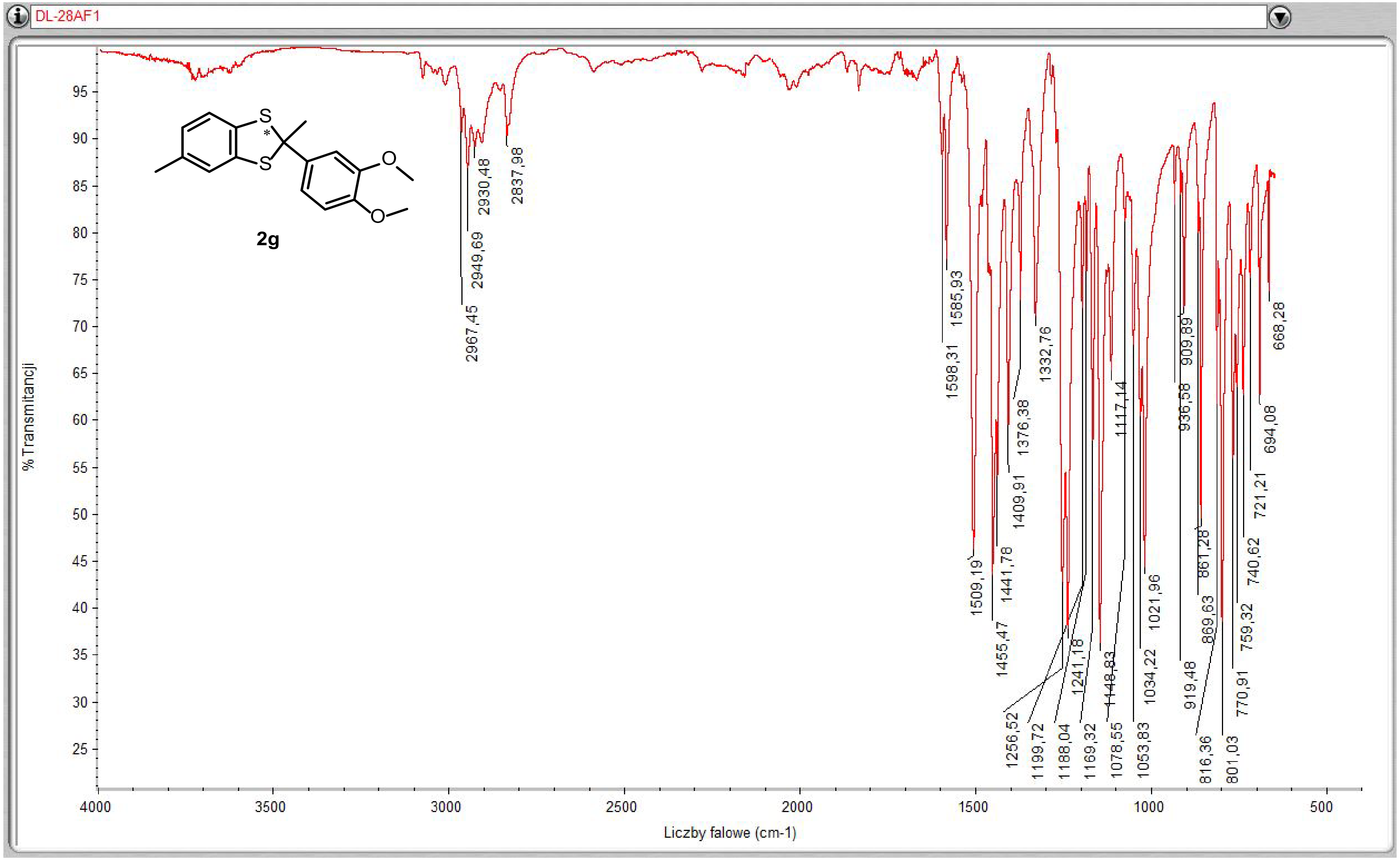
DL-28AF1
% Transmitancji
Liczby falowe (cm-1)
2g
2967,45
2949,69
2930,48
2837,98
1598,31
1585,93
1509,19
1455,47
1441,78
1409,91
1376,38
1332,76
1256,52
1241,18
1199,72
1188,04
1169,32
1148,83
1117,14
1078,55
1053,83
1034,22
1021,96
936,58
919,48
909,89
869,63
861,28
816,36
801,03
770,91
759,32
740,62
721,21
694,08
668,28

DL-28_A_F1 1H_CDCl3 + 0.03% TMS, 25 st. C

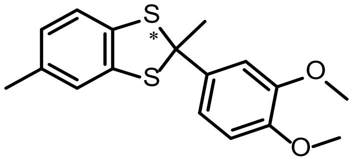

2g

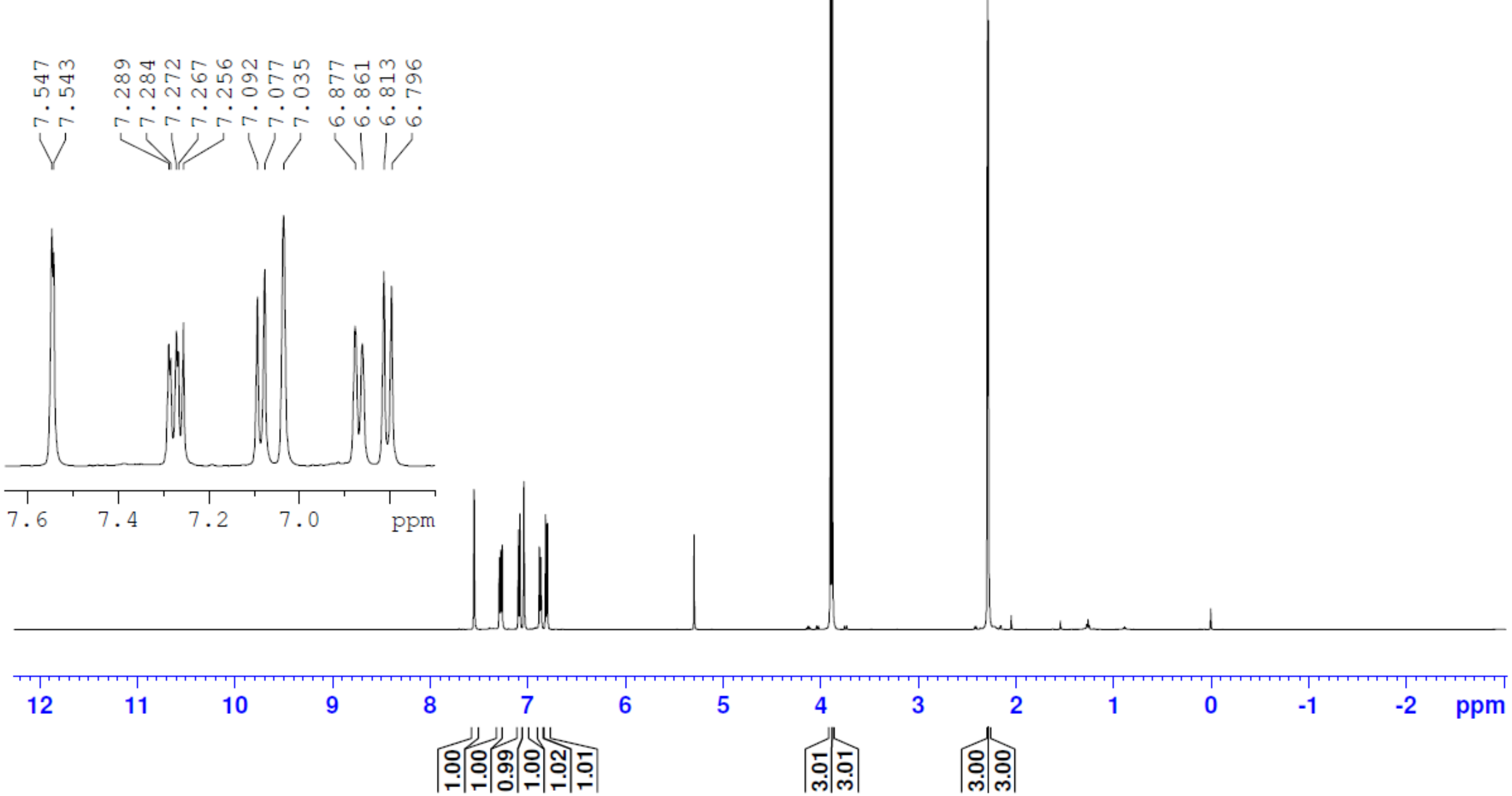

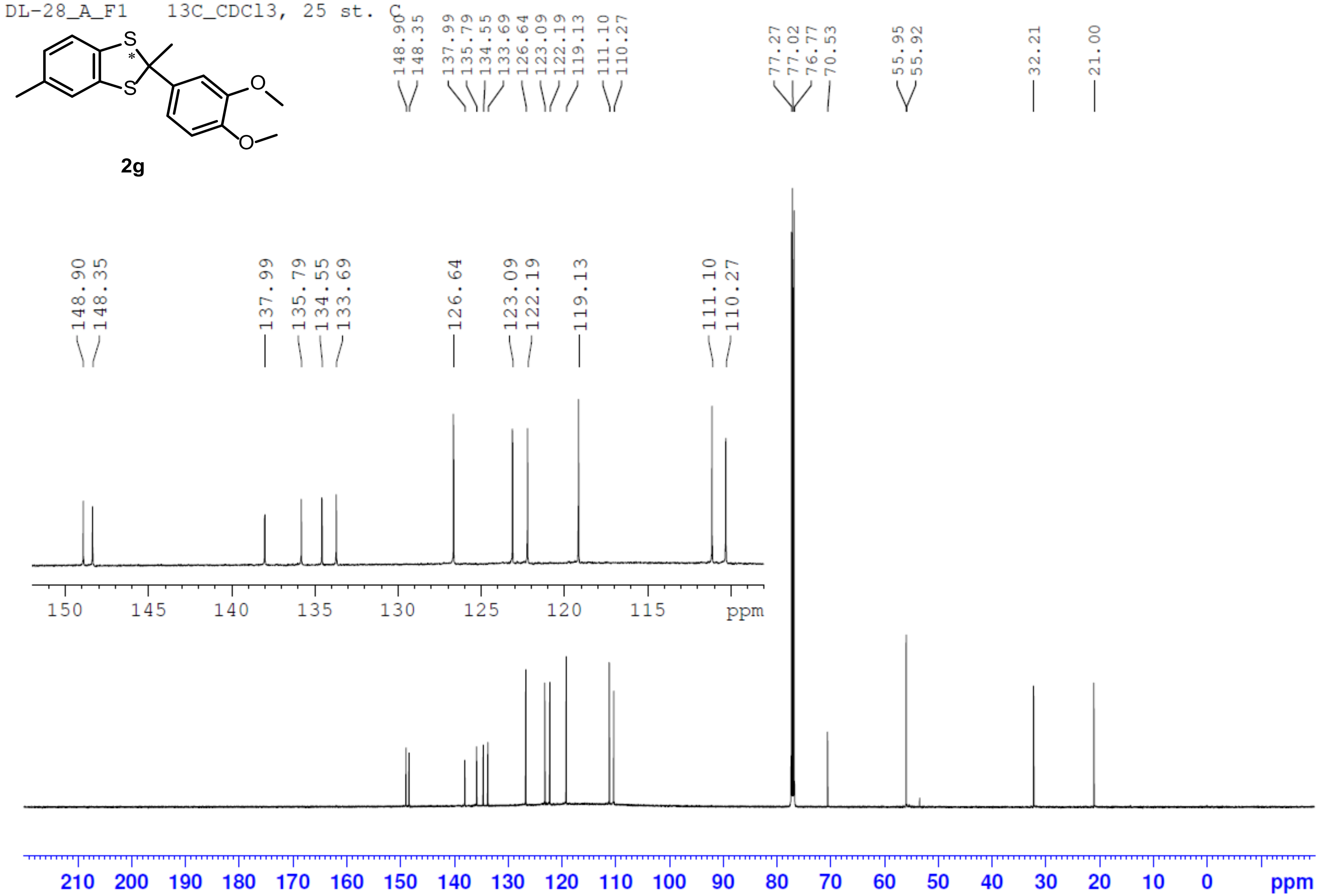
DL-28_A_F1 13C_CDCl3, 25 st. C
2g
148.90
148.35
137.99
135.79
134.55
133.69
126.64
123.09
122.19
119.13
111.10
110.27
77.27
77.02
76.77
70.53
55.95
55.92
32.21
21.00
150 145 140 135 130 125 120 115 ppm
210 200 190 180 170 160 150 140 130 120 110 100 90 80 70 60 50 40 30 20 10 0 ppm

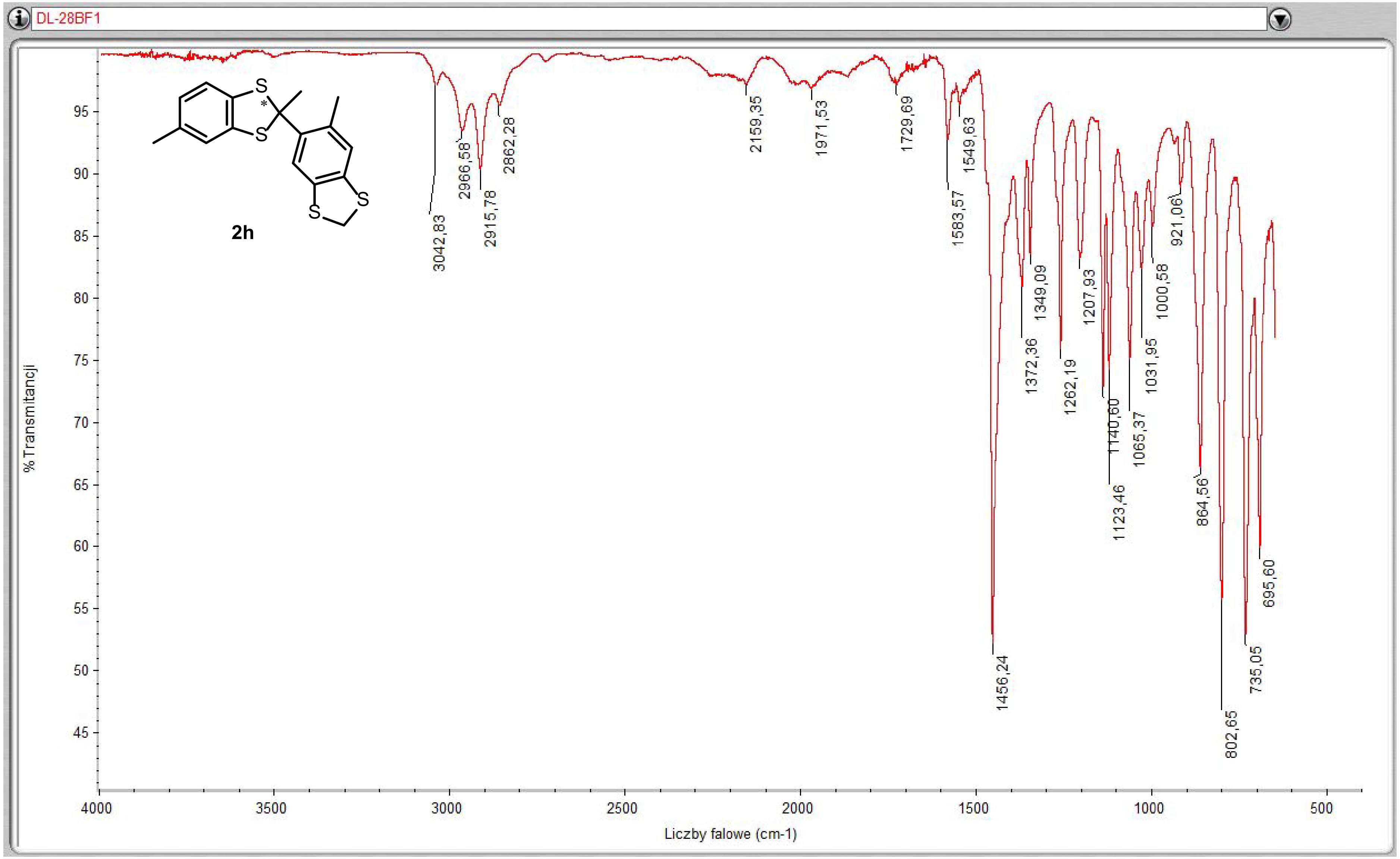
DL-28BF1
2h
% Transmitancji
Liczby falowe (cm-1)
3042,83
2966,58
2915,78
2862,28
2159,35
1971,53
1729,69
1583,57
1549,63
1456,24
1372,36
1349,09
1262,19
1207,93
1140,60
1123,46
1065,37
1031,95
1000,58
921,06
864,56
802,65
735,05
695,60

DL-28_B_F1 1H_CDCl3 + 0.03% TMS, 25 st. C

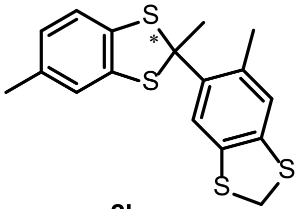

2h

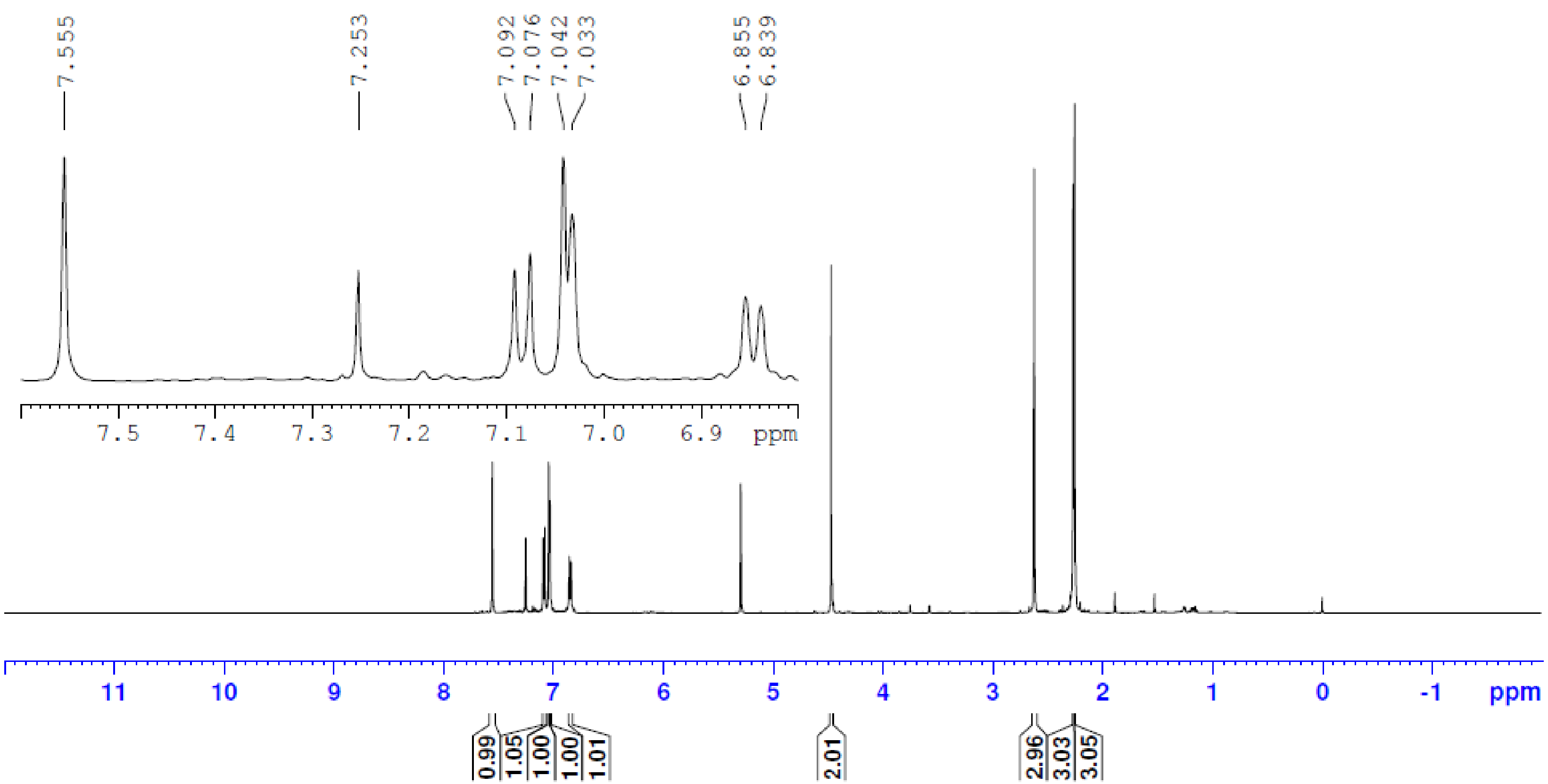

DL-28_B_F1 13C_CDCl3, 25 st. C

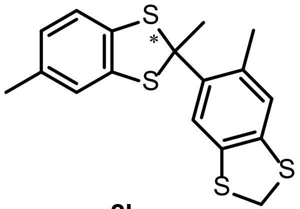

2h

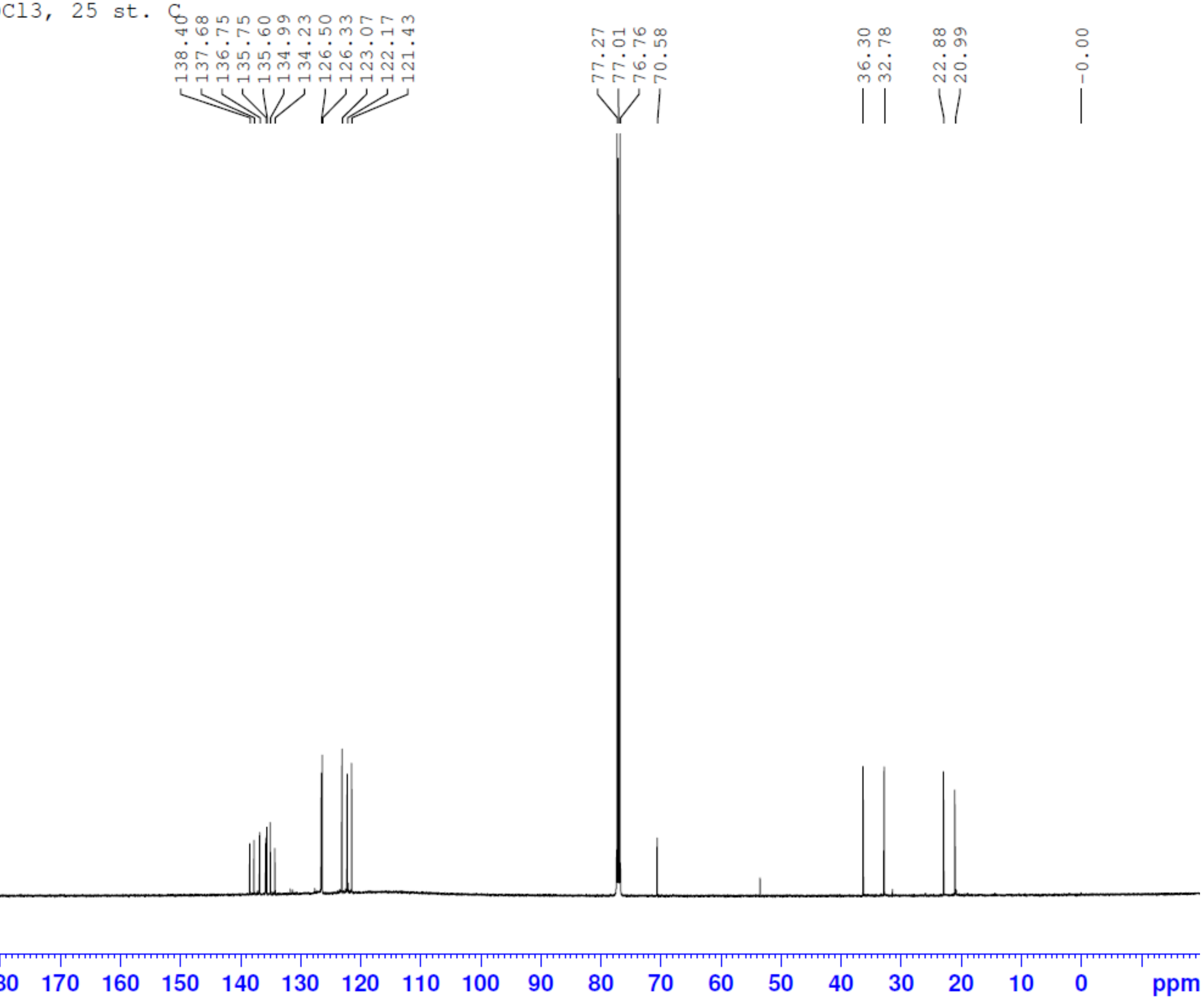

DL-28_B_F1 13C_CDCl3, 25 st. C

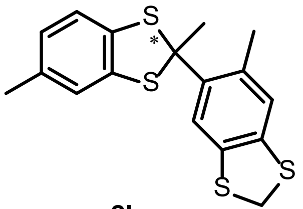

2h

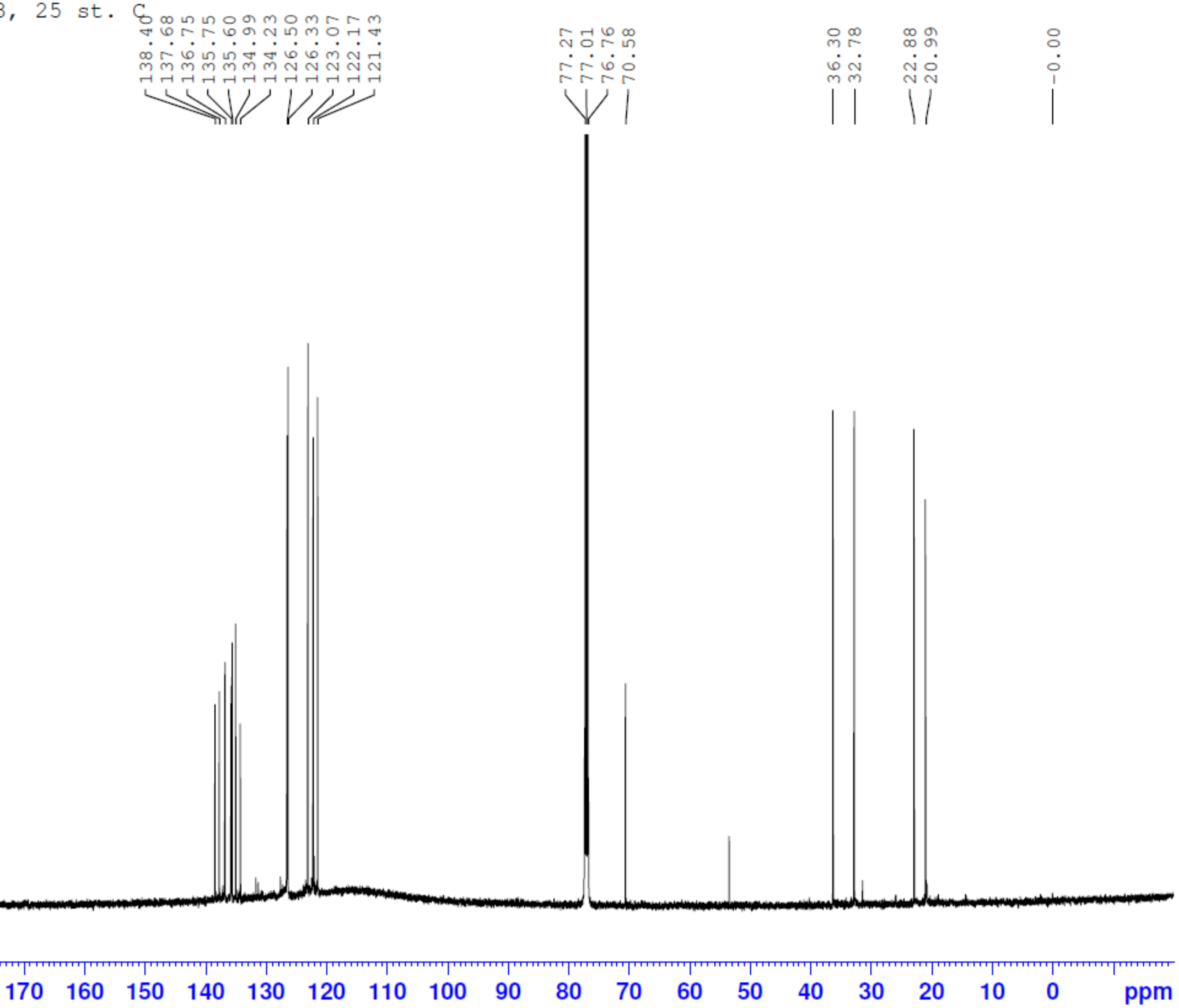

DL-28_B_F1   13C_CDCl3, 25 st. C

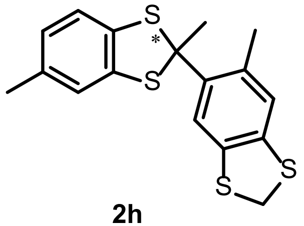

2h

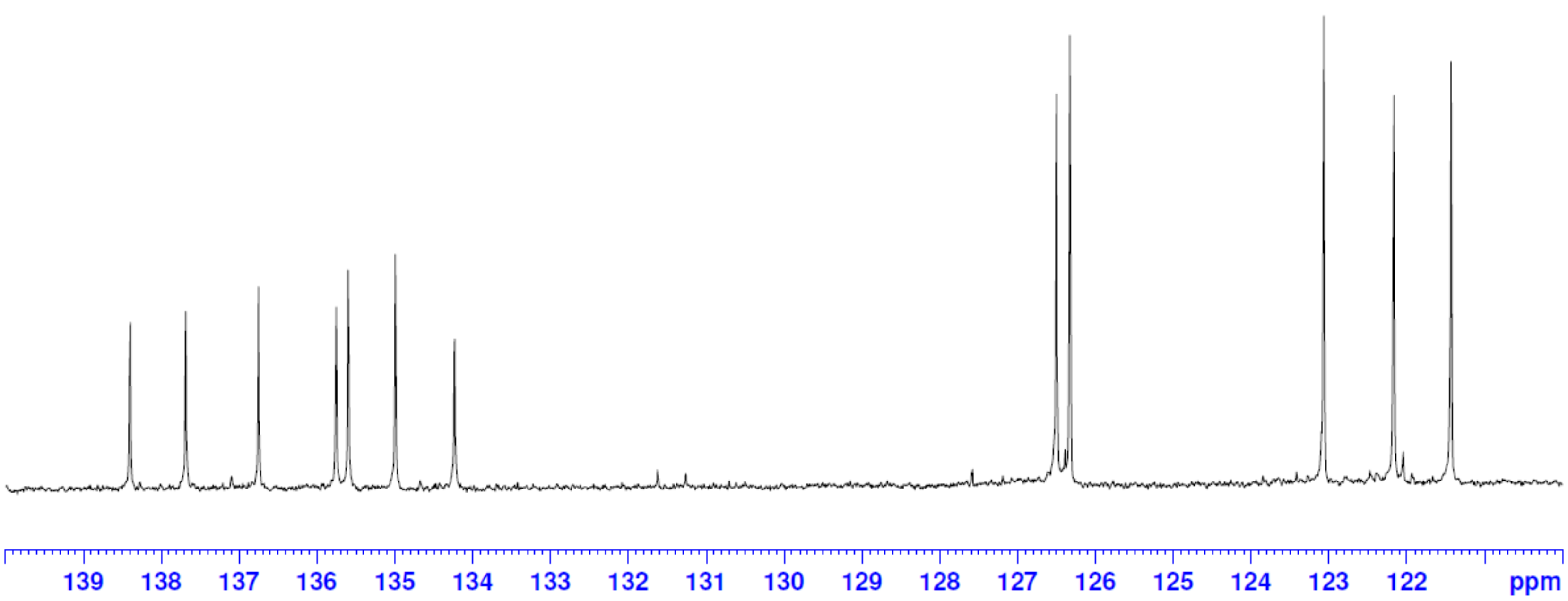

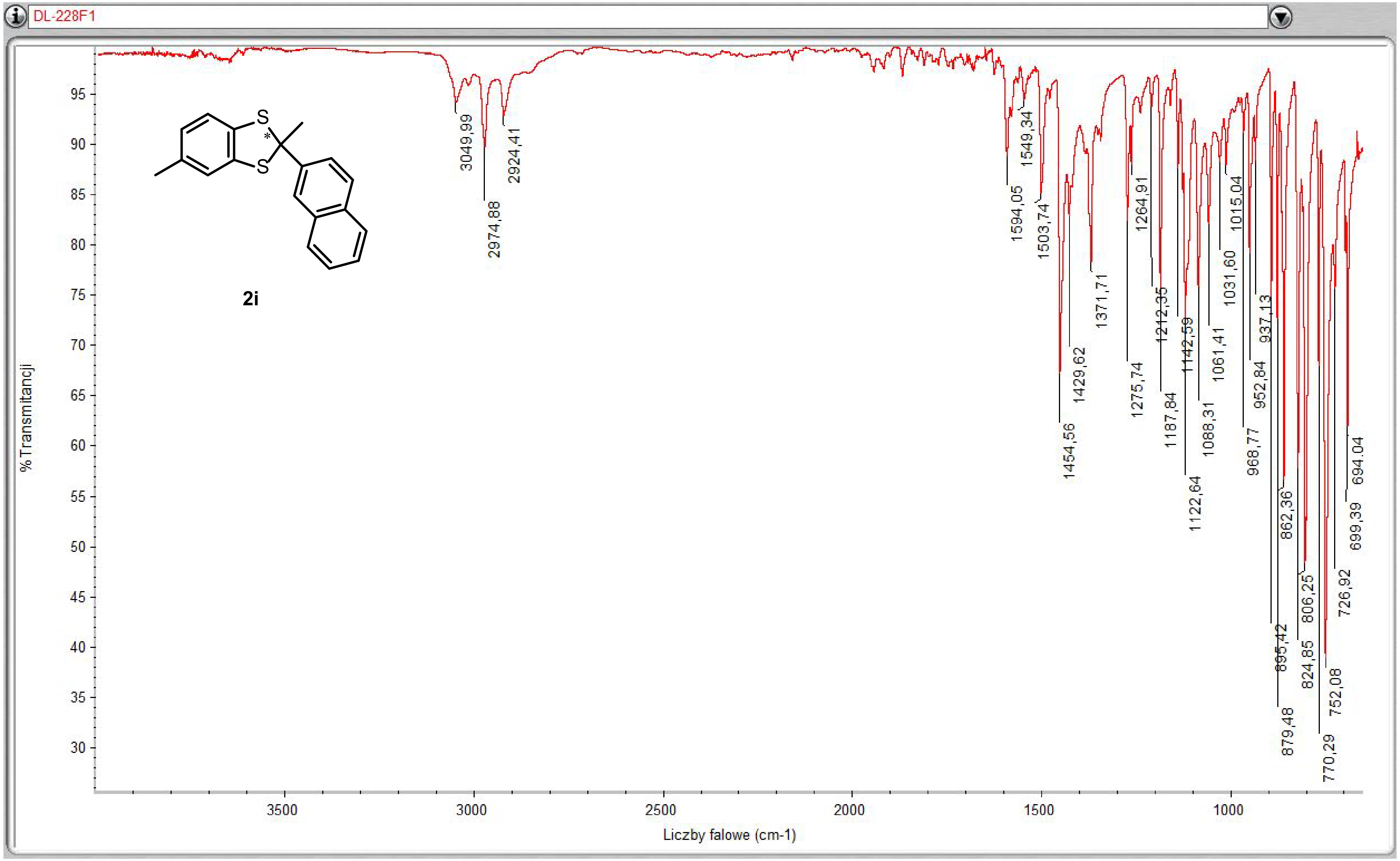
DL-228F1
2i
% Transmitancji
Liczby falowe (cm-1)
3049,99
2974,88
2924,41
1594,05
1549,34
1503,74
1454,56
1429,62
1371,71
1275,74
1264,91
1212,35
1187,84
1142,59
1122,64
1088,31
1061,41
1031,60
1015,04
968,77
952,84
937,13
895,42
879,48
862,36
824,85
806,25
770,29
752,08
726,92
699,39
694,04

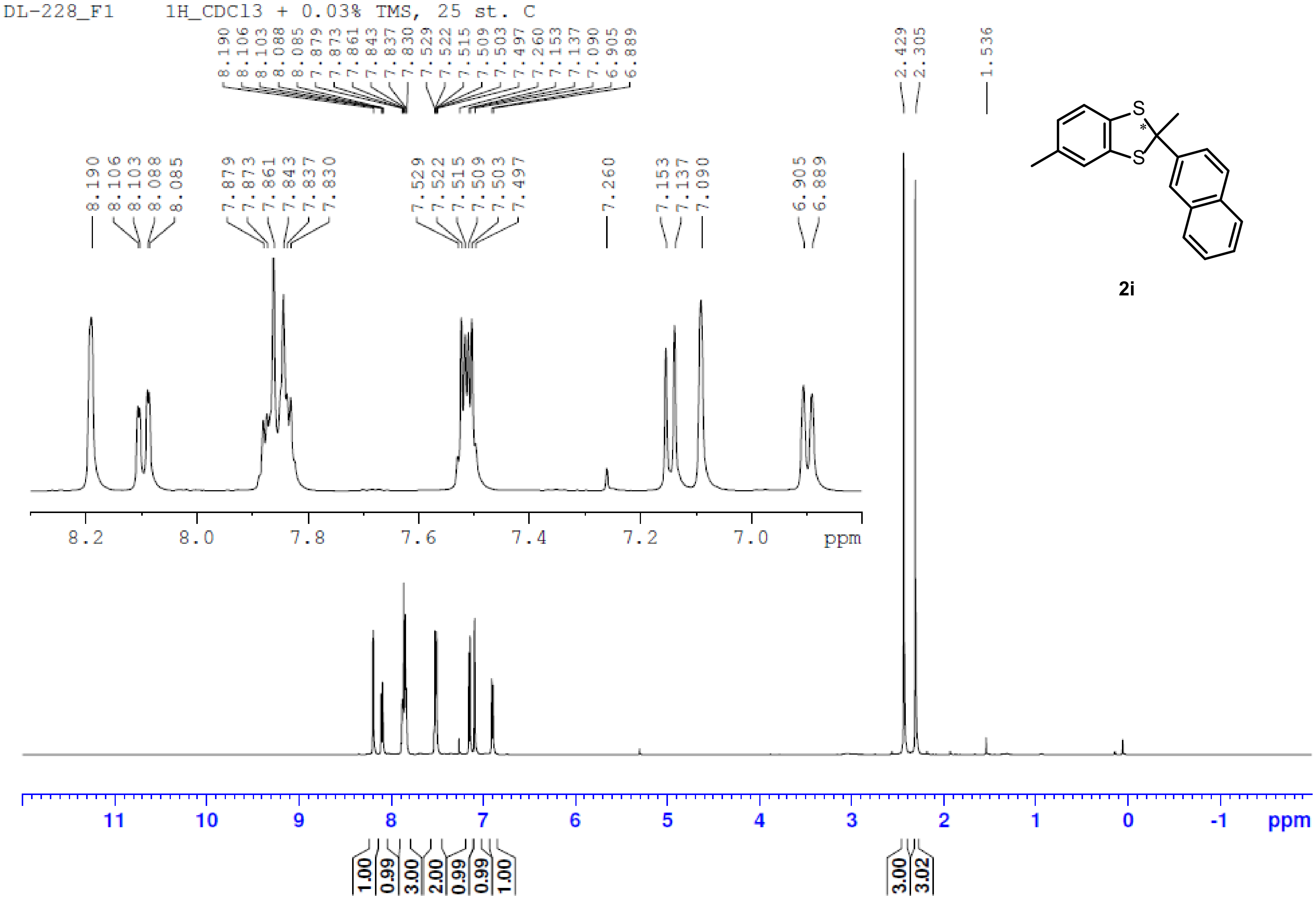
DL-228_F1 1H_CDCl3 + 0.03% TMS, 25 st. C
8.190 8.106 8.103 8.088 8.085 7.879 7.873 7.861 7.843 7.837 7.830 7.529 7.522 7.515 7.509 7.503 7.497 7.260 7.153 7.137 7.090 6.905 6.889
2.429 2.305
1.536
S
S
*
2i
8.2 8.0 7.8 7.6 7.4 7.2 7.0 ppm
11 10 9 8 7 6 5 4 3 2 1 0 -1 ppm
1.00 0.99 3.00 2.00 0.99 0.99 1.00
3.00 3.02

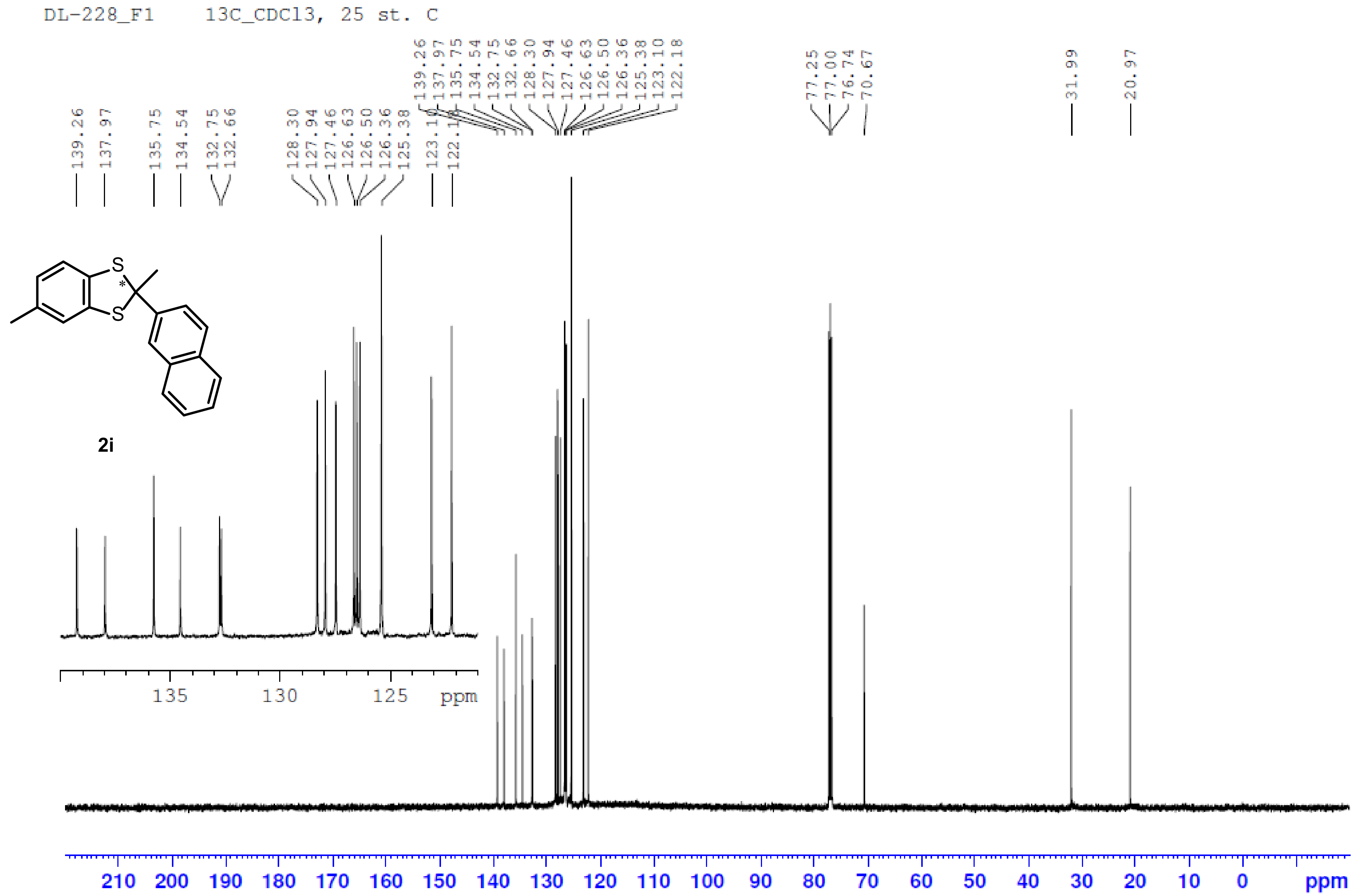
DL-228_F1 13C_CDCl3, 25 st. C
139.26
137.97
135.75
134.54
132.75
132.66
128.30
127.94
127.46
126.63
126.50
126.36
125.38
123.10
122.18
77.25
77.00
76.74
70.67
31.99
20.97
S
S
*
2i
135
130
125
ppm
210 200 190 180 170 160 150 140 130 120 110 100 90 80 70 60 50 40 30 20 10 0
ppm

DL-228_F1   13C_CDCl3 + 0.03% TMS, 25 st. C

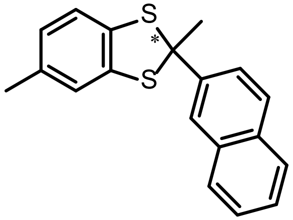

2i

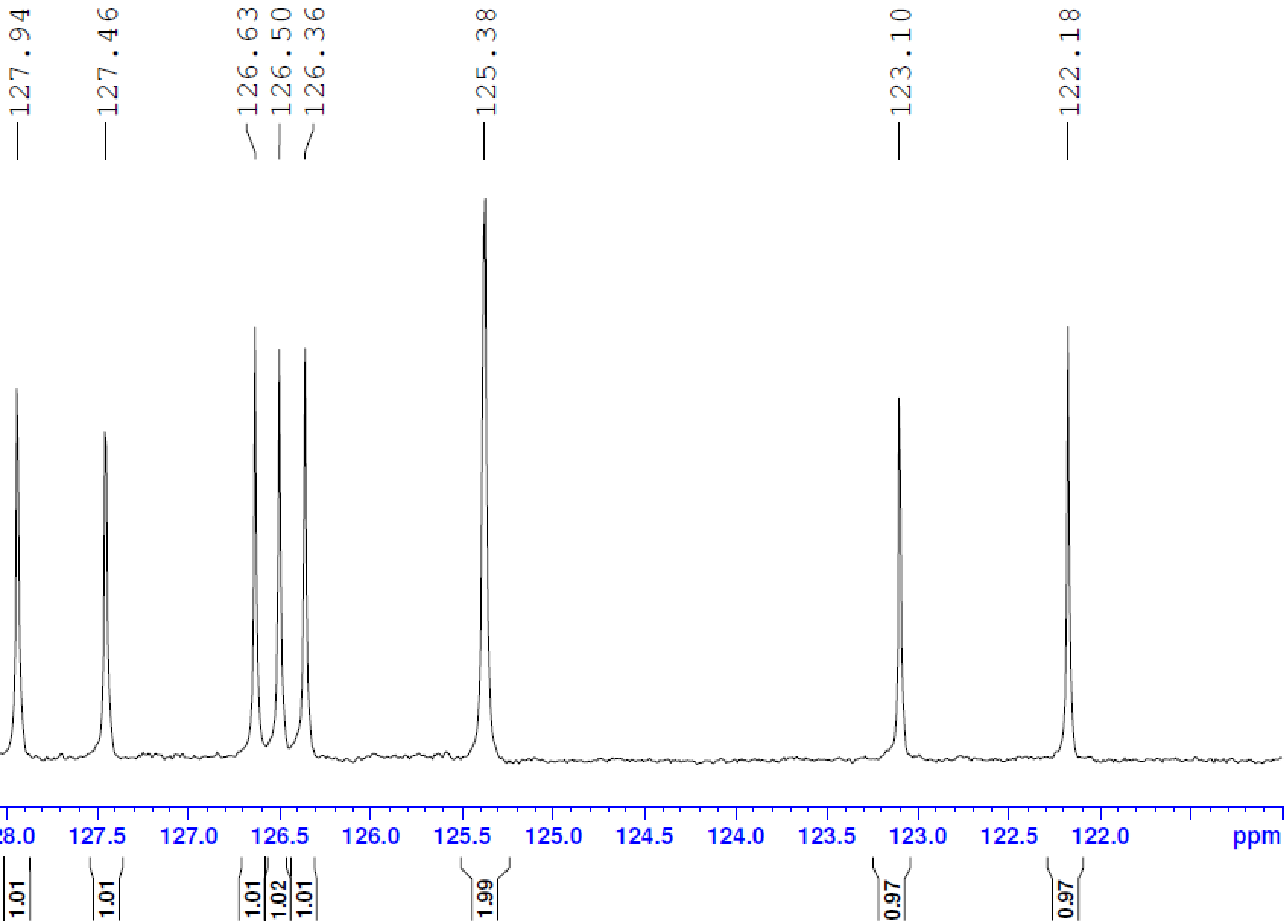

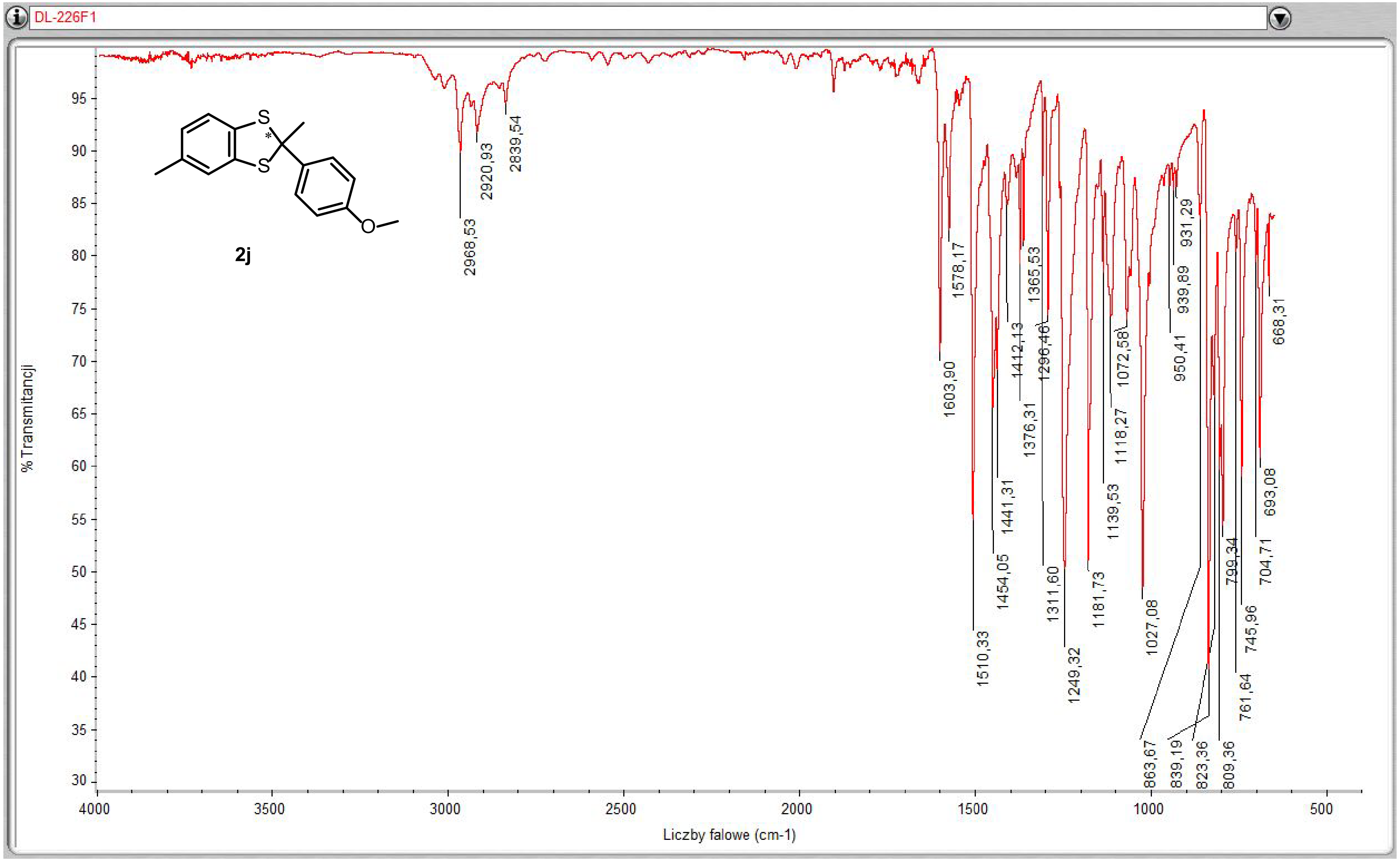
DL-226F1
2j
% Transmitancji
Liczby falowe (cm-1)
2968,53
2920,93
2839,54
1603,90
1578,17
1510,33
1454,05
1441,31
1412,13
1376,31
1365,53
1311,60
1296,46
1249,32
1181,73
1139,53
1118,27
1072,58
1027,08
950,41
939,89
931,29
863,67
839,19
823,36
809,36
799,34
761,64
745,96
704,71
693,08
668,31

DL-226_F1 1H_CDCl3, 25 st. C

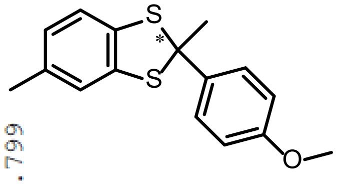

2j

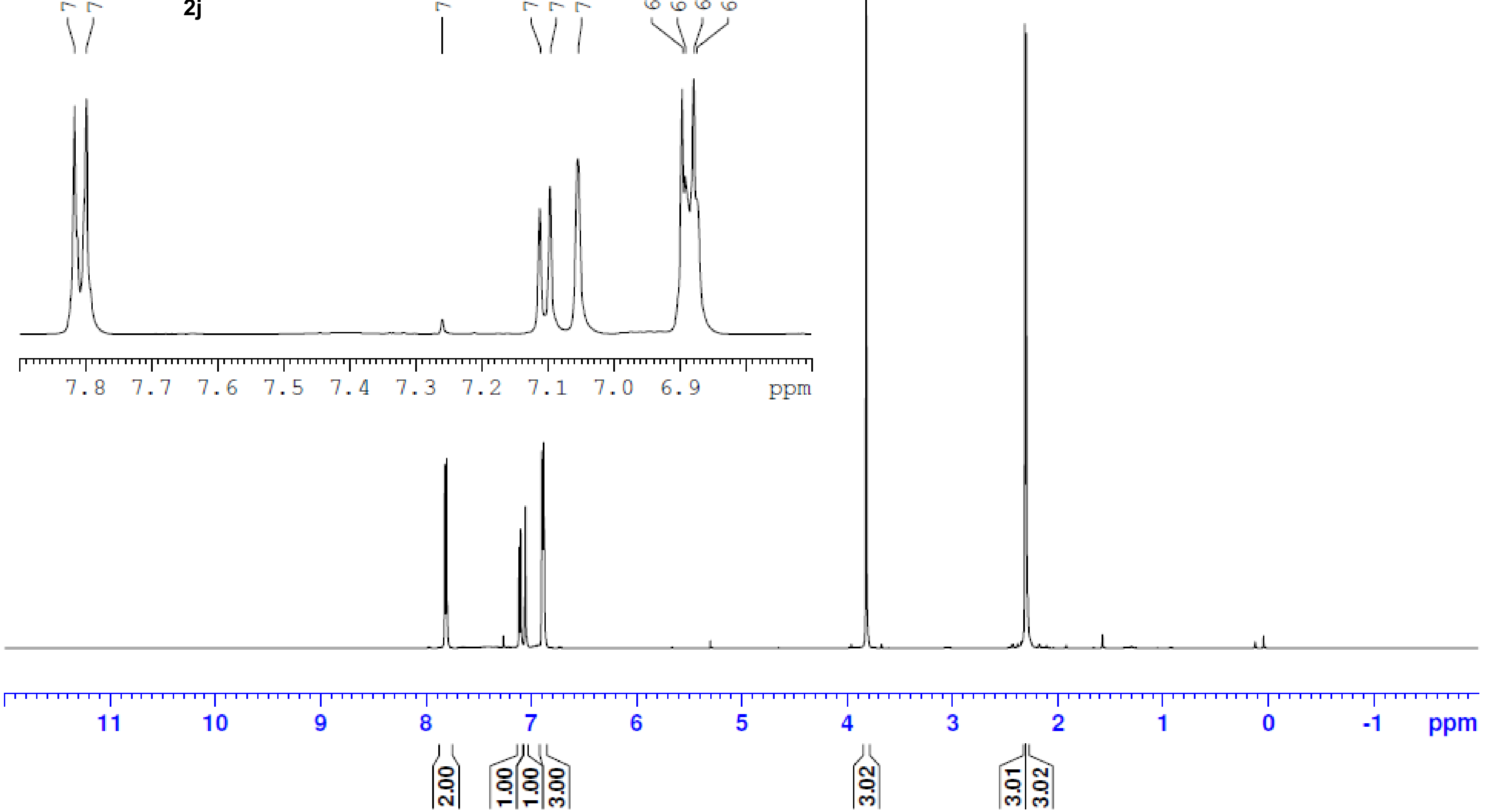

DL-226_F1 13C_CDCl3 +0.03% TMS, 25 st. C

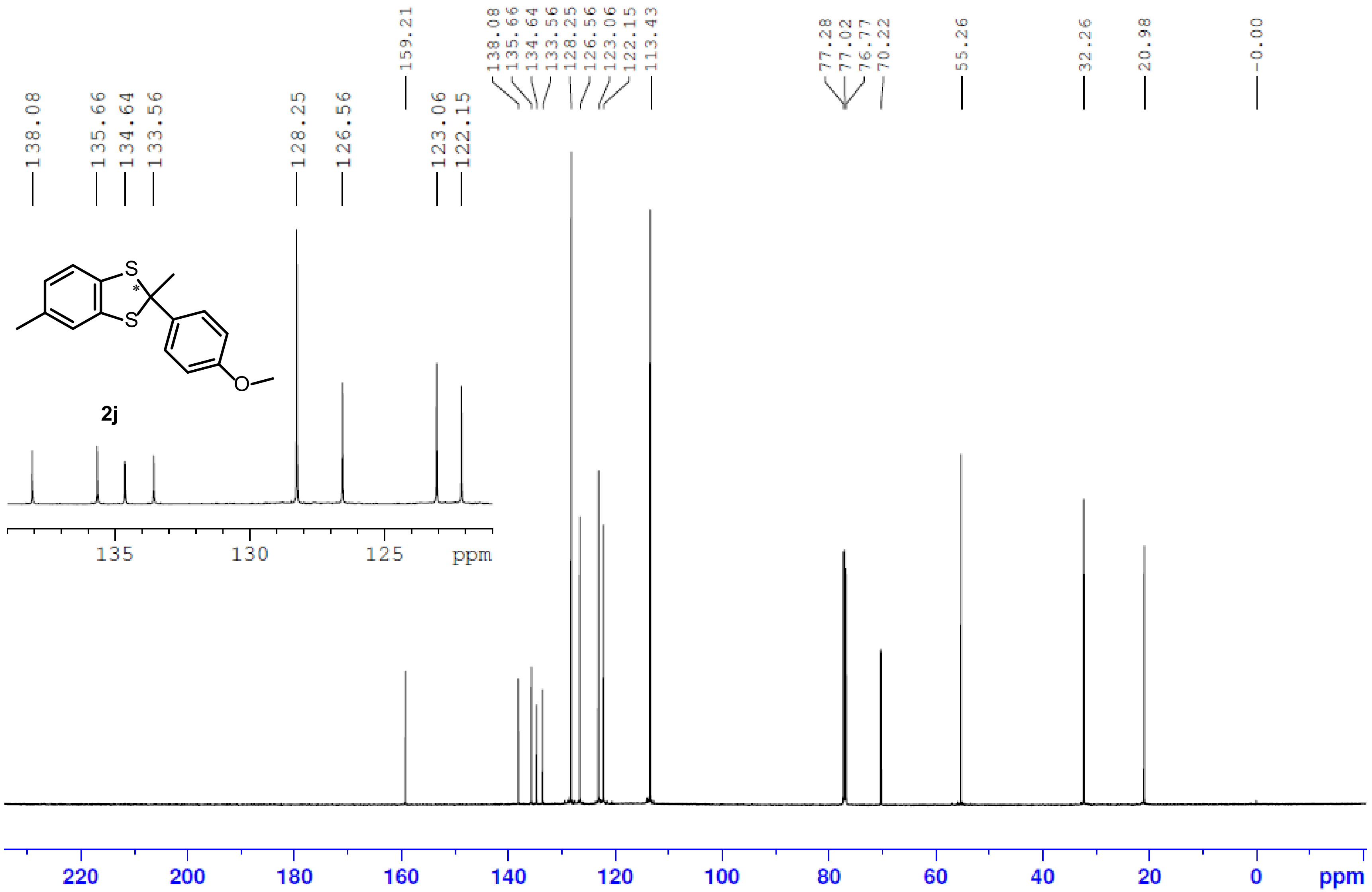

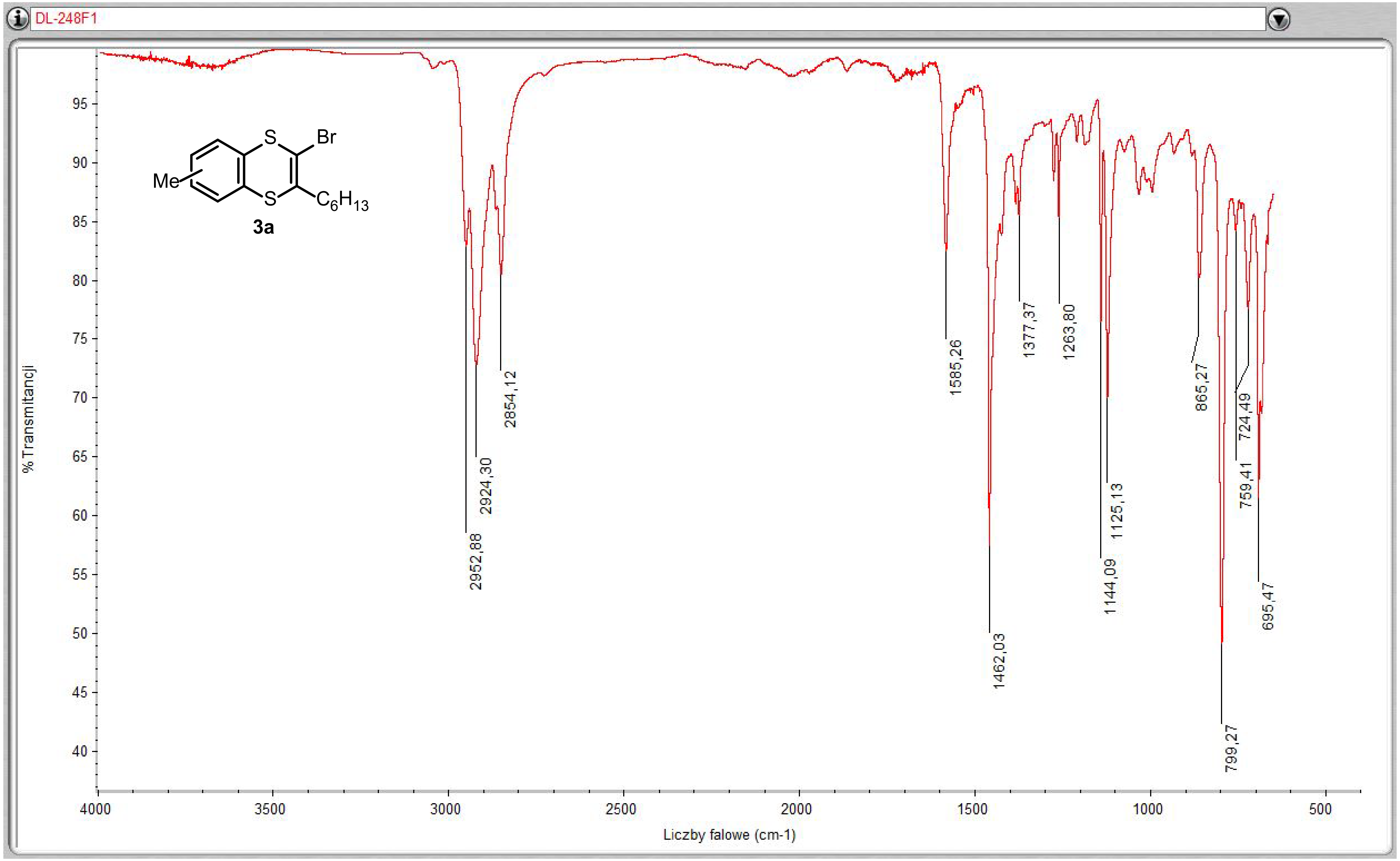
DL-248F1
S
Br
Me
S
C6H13
3a
% Transmitancji
Liczby falowe (cm-1)
2952,88
2924,30
2854,12
1585,26
1462,03
1377,37
1263,80
1144,09
1125,13
865,27
799,27
759,41
724,49
695,47
4000
3500
3000
2500
2000
1500
1000
500
95
90
85
80
75
70
65
60
55
50
45
40

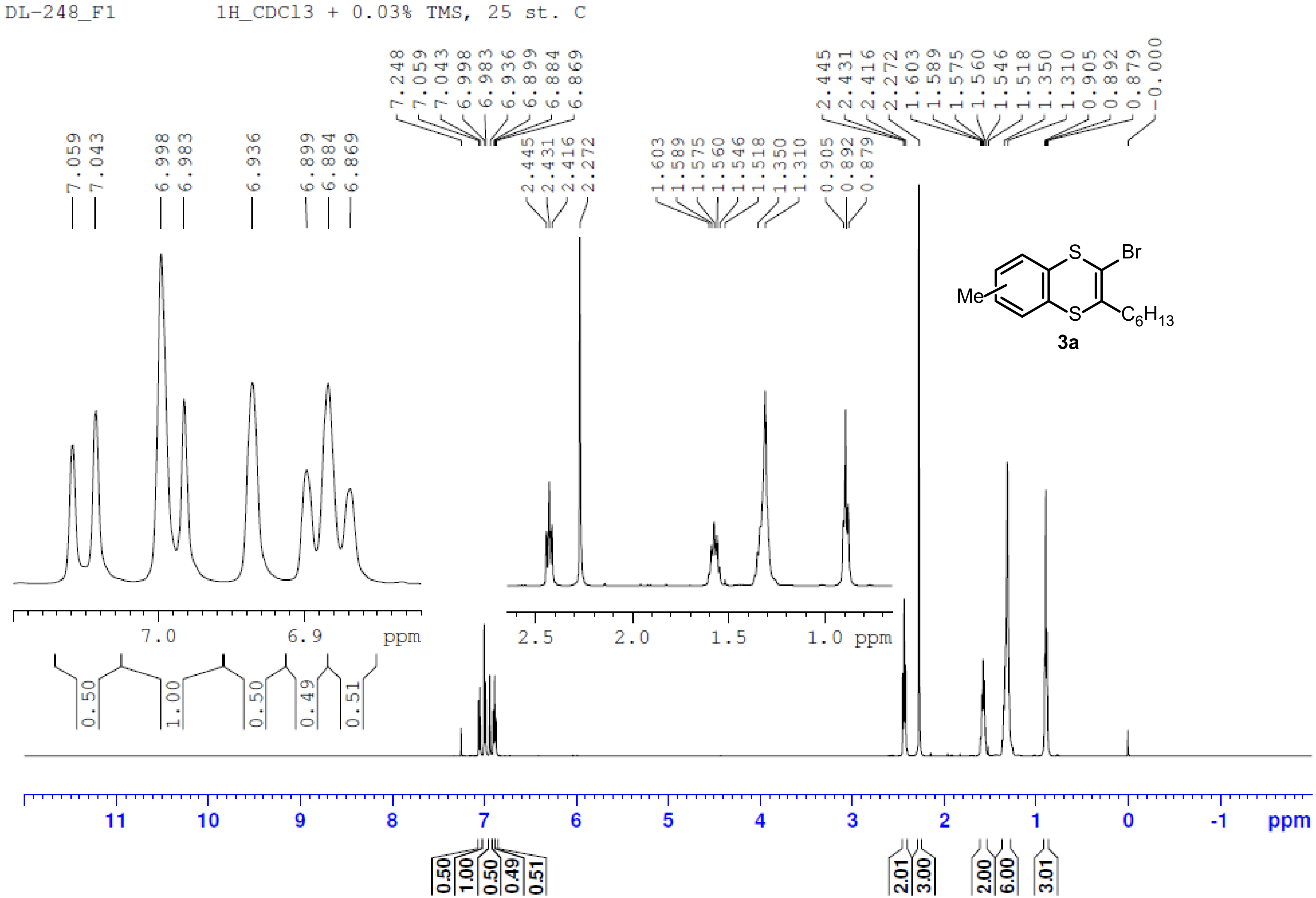

DL-248_F1 1H_CDCl3 + 0.03% TMS, 25 st. C
7.248 7.059 7.043 6.998 6.983 6.936 6.899 6.884 6.869
2.445 2.431 2.416 2.272 1.603 1.589 1.575 1.560 1.546 1.518 1.350 1.310 0.905 0.892 0.879 -0.000
Me
S
Br
S
C_6H_{13}
3a
0.50 1.00 0.50 0.49 0.51
2.01 3.00 2.00 6.00 3.01
ppm

DL-248_F1 13C_CDCl3 + 0.03% TMS, 25 st. C

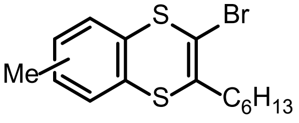

3a

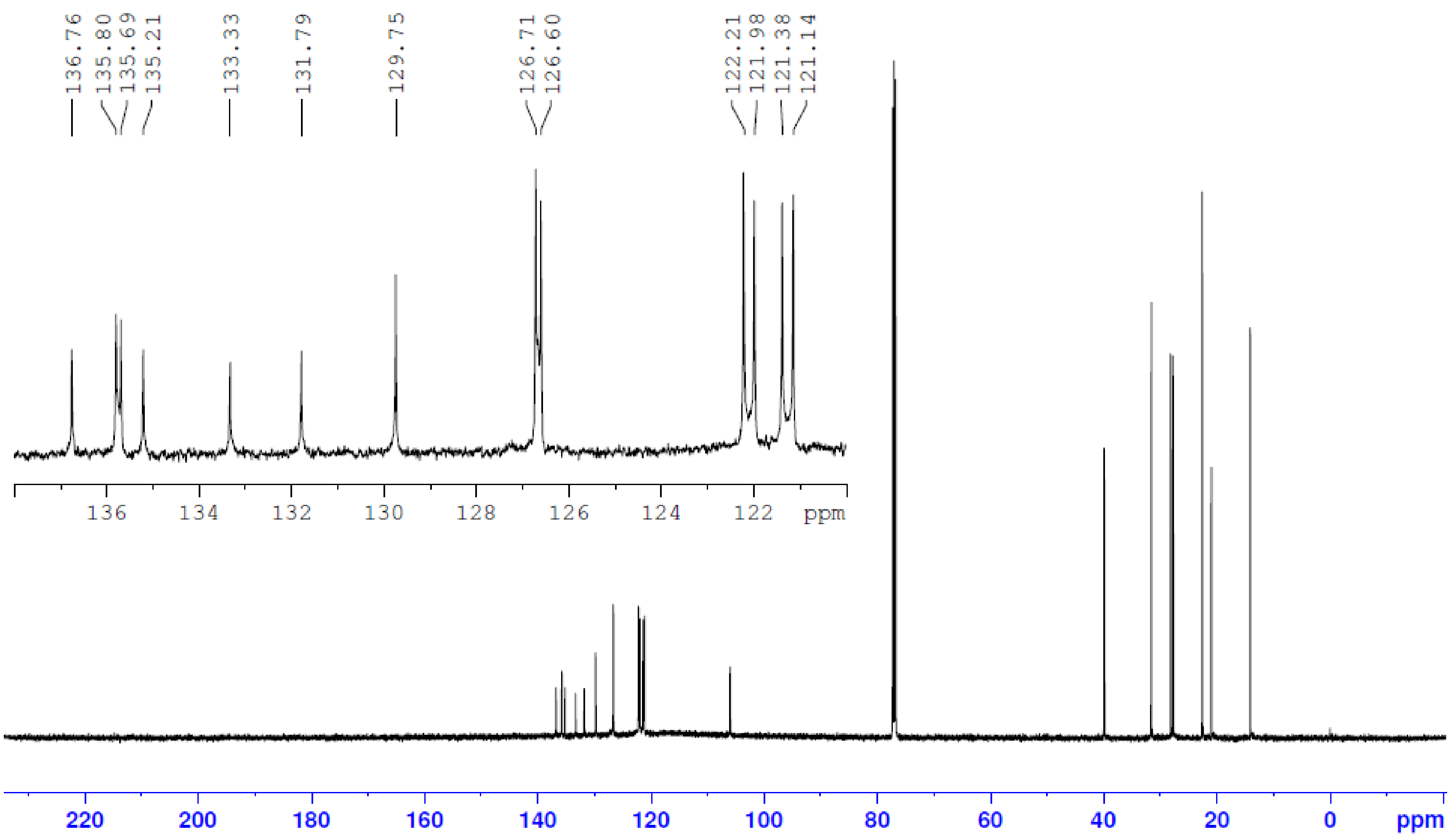

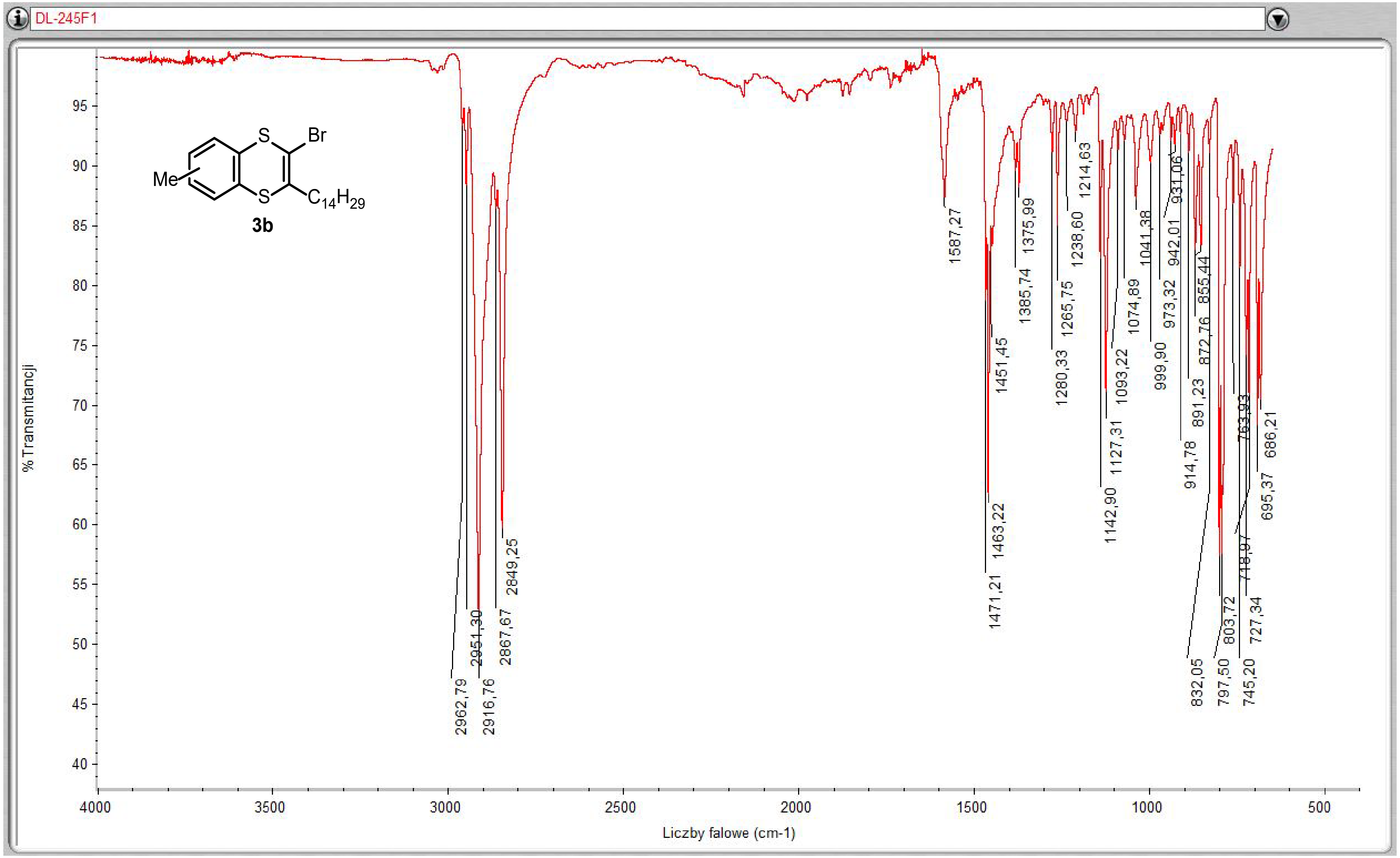

DL-245F1
3b
Me
S
Br
$C_{14}H_{29}$
% Transmitancji
Liczby falowe (cm-1)
2962,79
2951,30
2916,76
2867,67
2849,25
1587,27
1471,21
1463,22
1451,45
1385,74
1375,99
1280,33
1265,75
1238,60
1214,63
1142,90
1127,31
1093,22
1074,89
1041,38
999,90
973,32
942,01
931,06
914,78
891,23
872,76
855,44
832,05
803,72
797,50
763,93
745,20
727,34
718,97
695,37
686,21

DL-245_F1 1H_CDCl3 + 0.03% TMS, 25 st. C

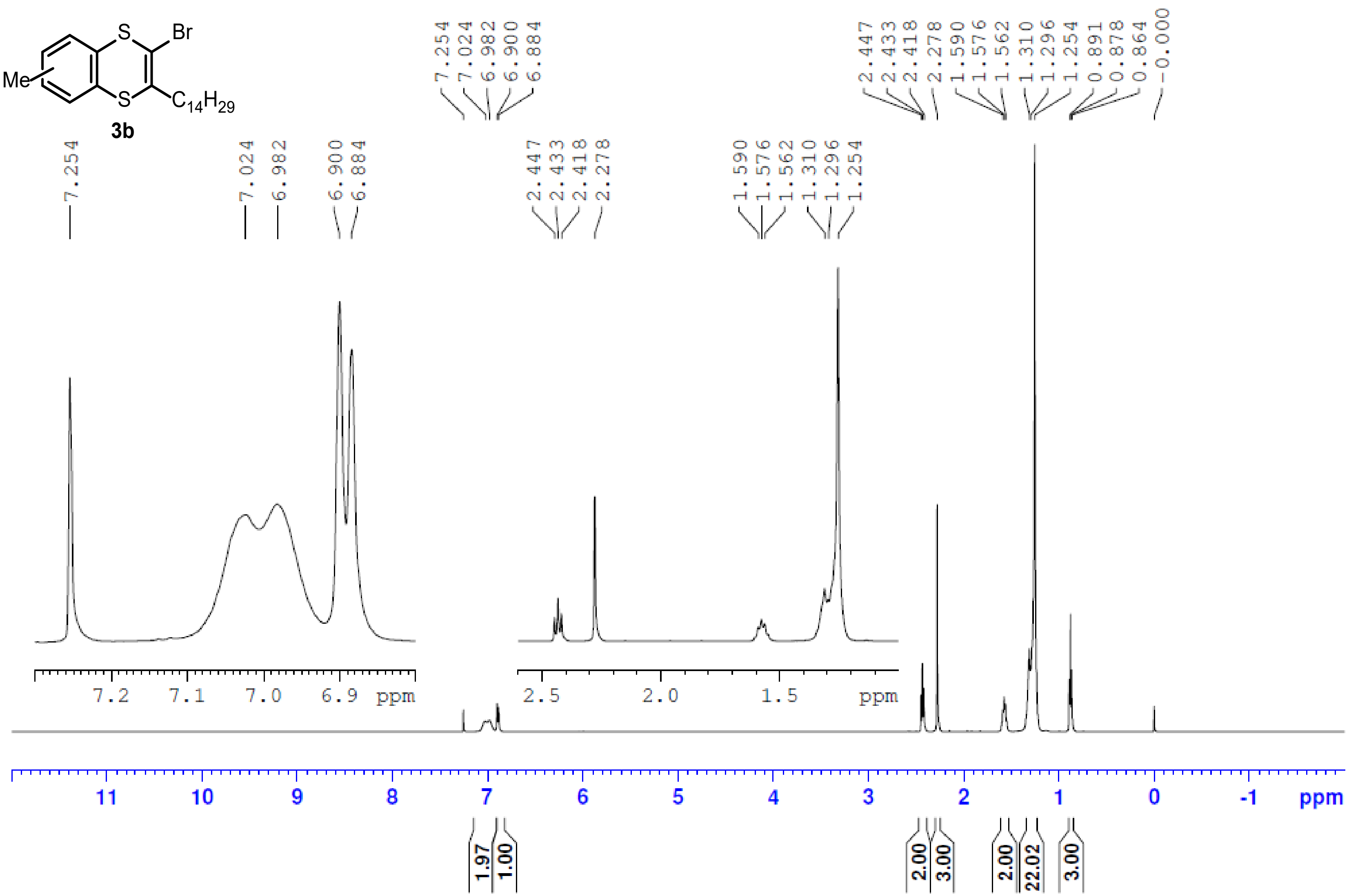

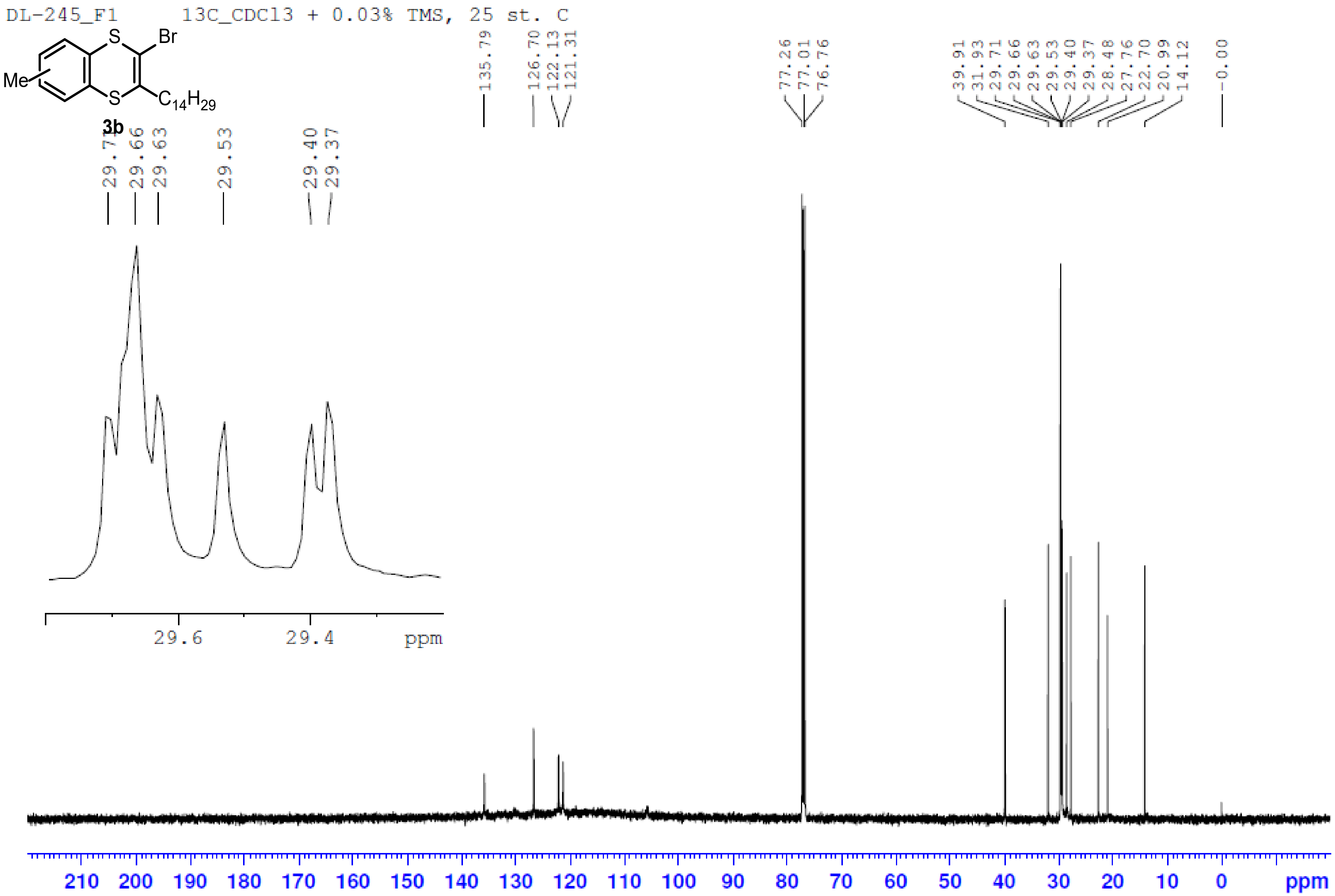

DL-245_F1 13C_CDCl3 + 0.03% TMS, 25 st. C
Br
Me
S
S
C14H29
3b
135.79
126.70
122.13
121.31
77.26
77.01
76.76
39.91
31.93
29.71
29.66
29.63
29.53
29.40
29.37
28.48
27.76
22.70
20.99
14.12
-0.00
29.71
29.66
29.63
29.53
29.40
29.37
29.6
29.4
ppm
210 200 190 180 170 160 150 140 130 120 110 100 90 80 70 60 50 40 30 20 10 0 ppm

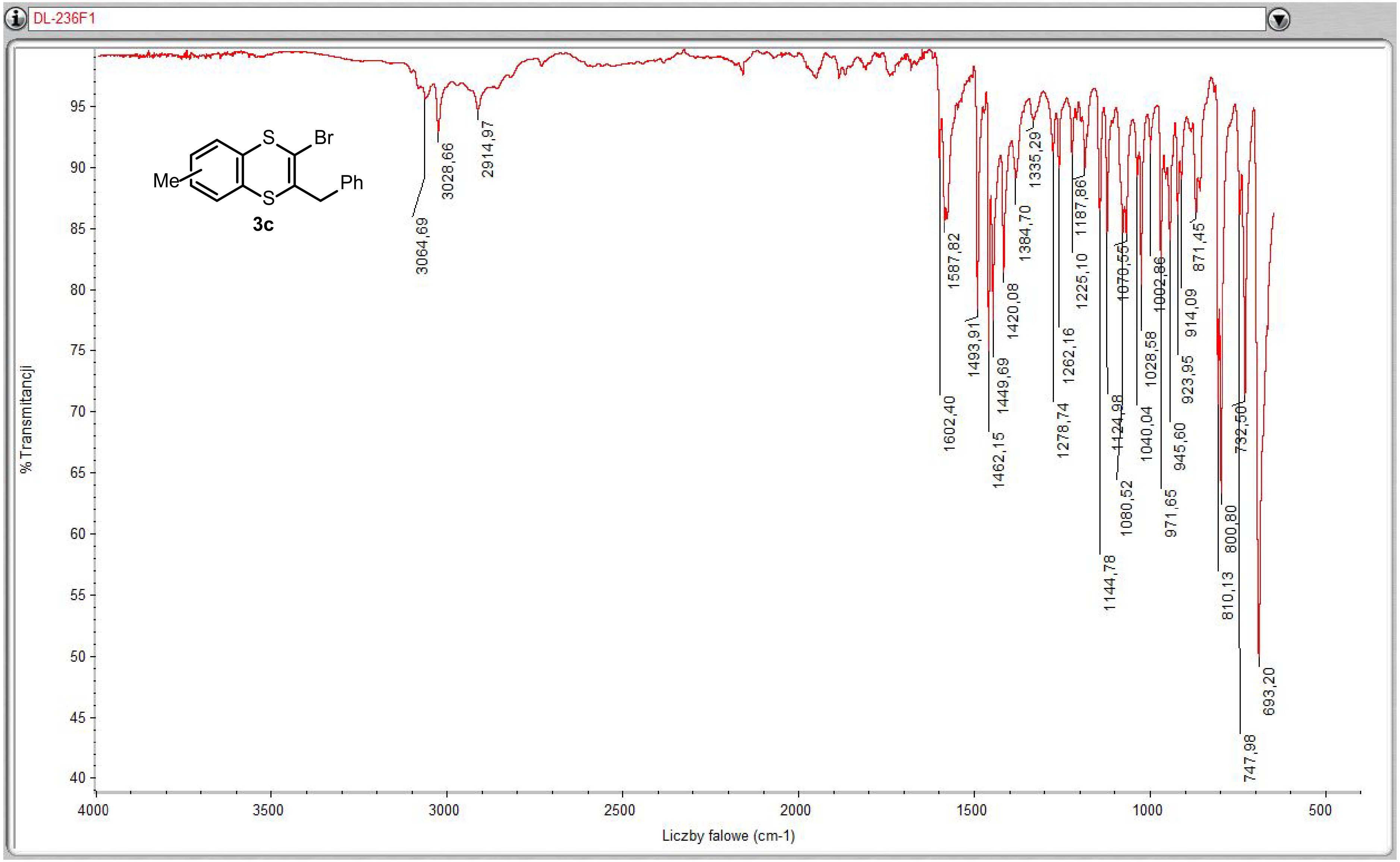
DL-236F1
%Transmitancji
Liczby falowe (cm-1)
Me
S
Br
S
Ph
3c
3064,69
3028,66
2914,97
1602,40
1587,82
1493,91
1462,15
1449,69
1420,08
1384,70
1335,29
1278,74
1262,16
1225,10
1187,86
1144,78
1124,98
1080,52
1070,55
1040,04
1028,58
1002,86
971,65
945,60
923,95
914,09
871,45
810,13
800,80
747,98
732,50
693,20

DL-236F1F1K 1H_CDCl3 + 0.03% TMS, 25 st. C

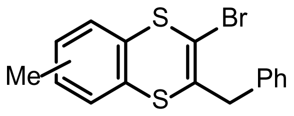

3c

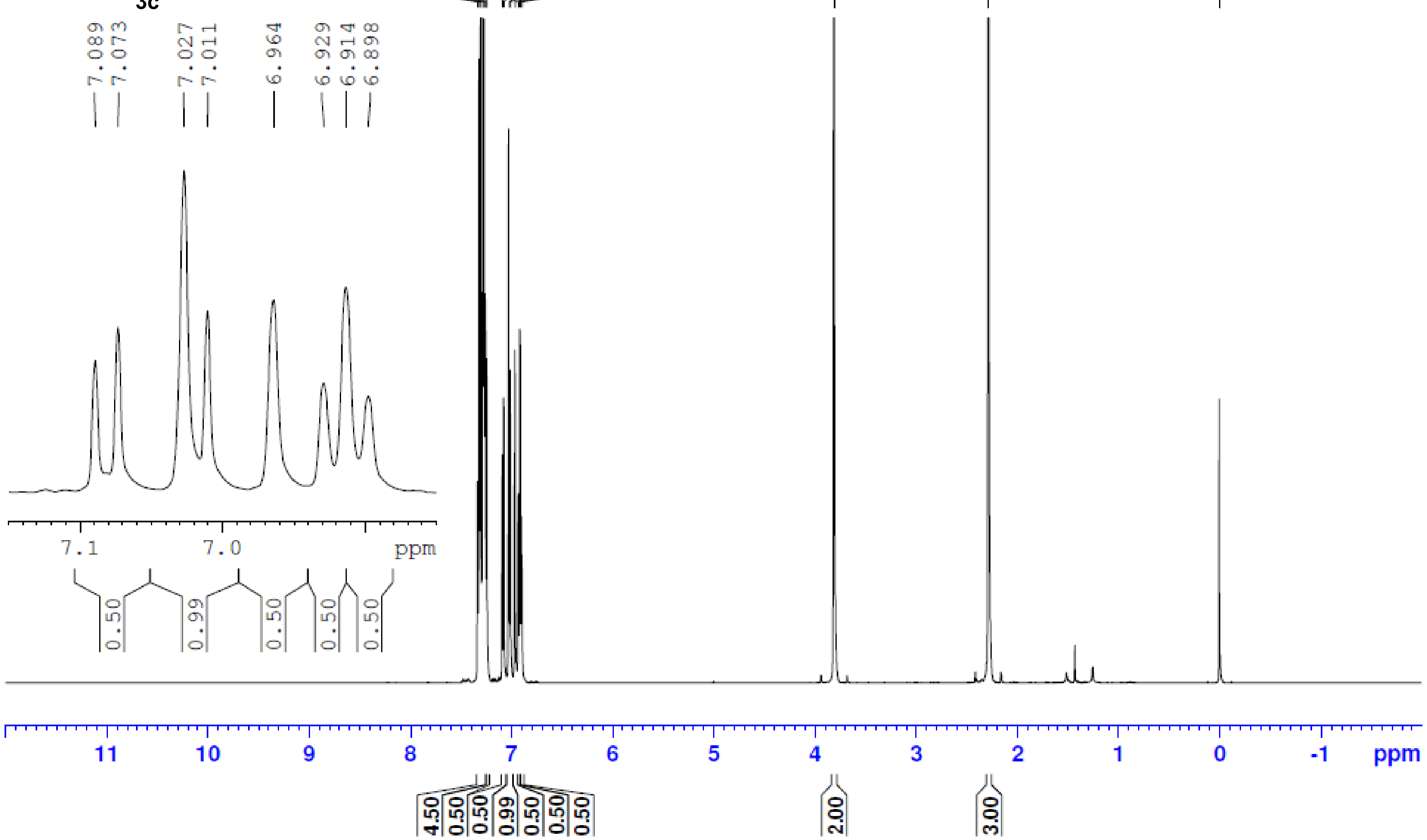

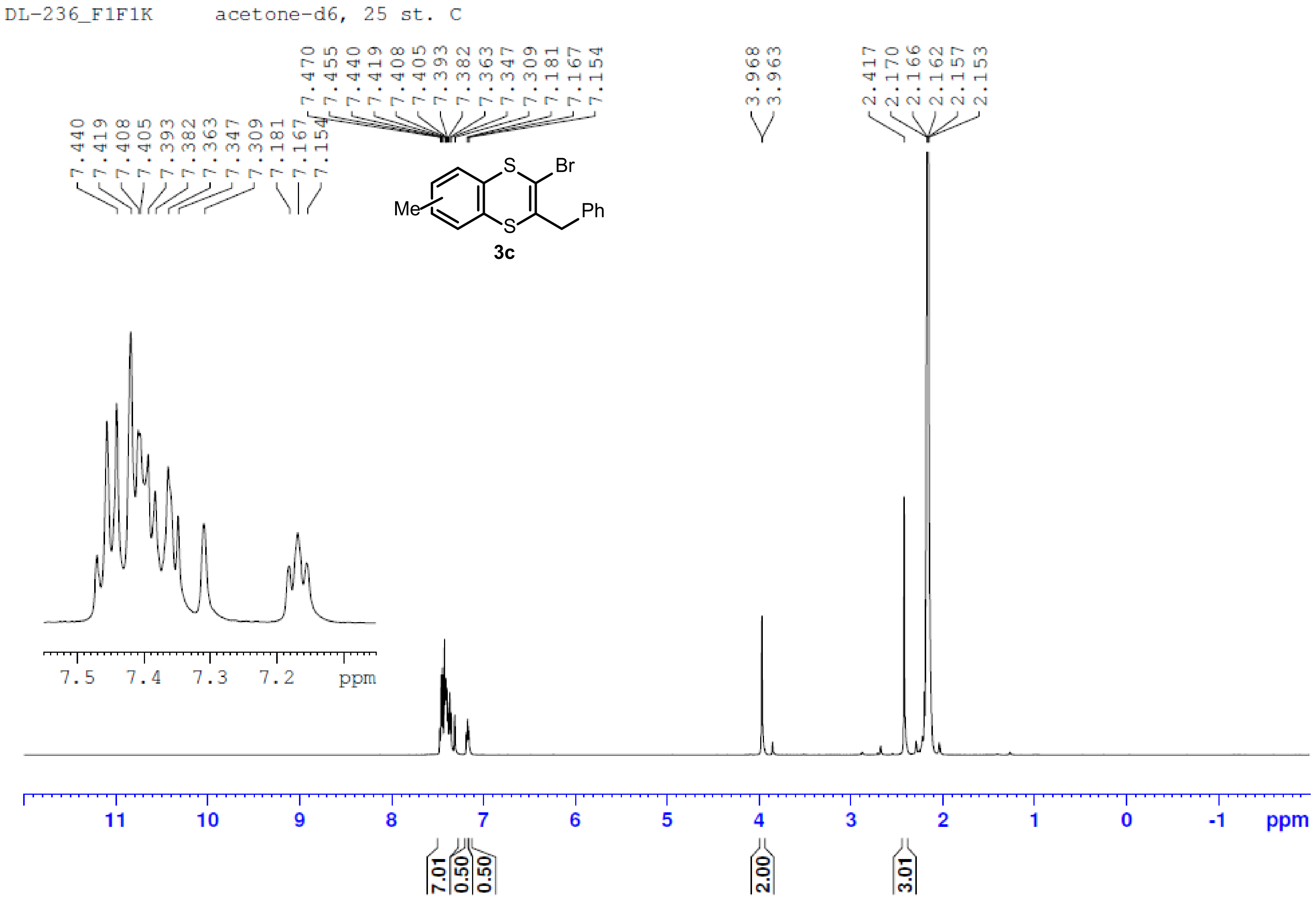
DL-236_F1F1K acetone-d6, 25 st. C
7.470
7.455
7.440
7.419
7.408
7.405
7.393
7.382
7.363
7.347
7.309
7.181
7.167
7.154
3.968
3.963
2.417
2.170
2.166
2.162
2.157
2.153
S
Br
Me
S
Ph
3c
ppm
7.01
0.50
0.50
2.00
3.01

DL-236 13C_CDCl3 + 0.03% TMS, 25 st. C

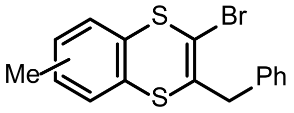

3c

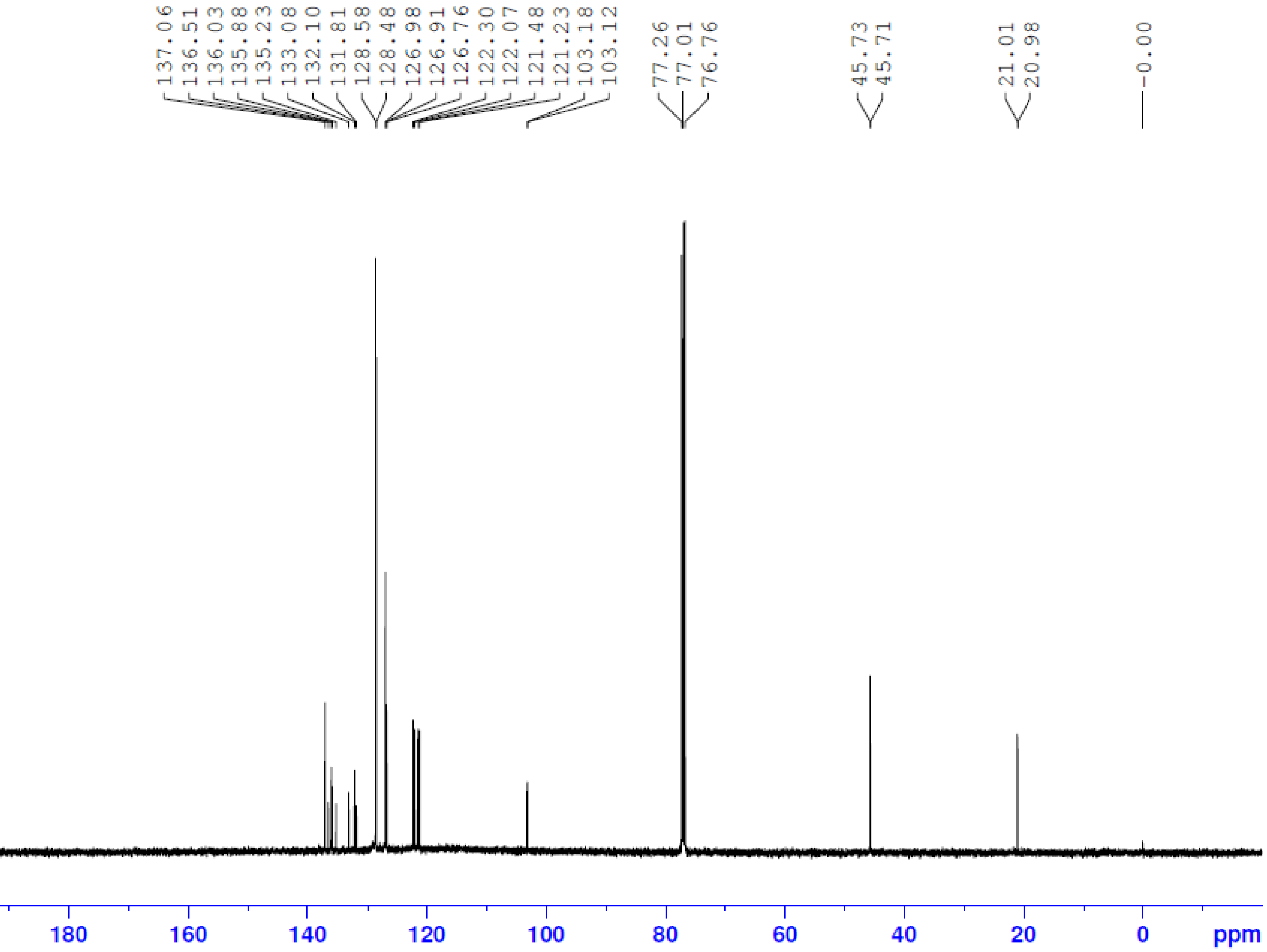

DL-236 gHSQC_CDCl3 + 0.03% TMS, 25 st. C

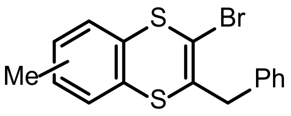

3c

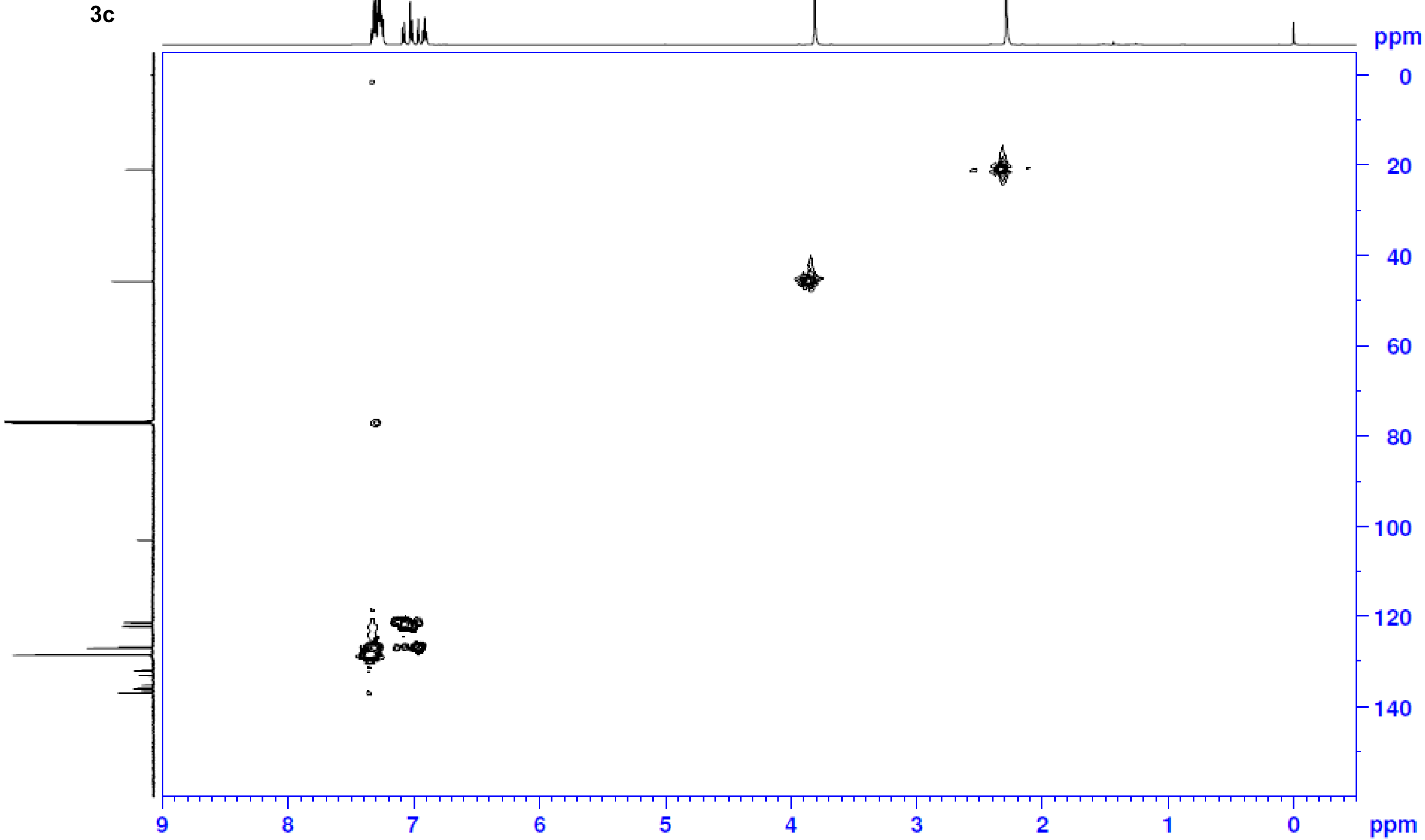

DL-236 gHMBC_CDCl3 + 0.03% TMS, 25 st. C

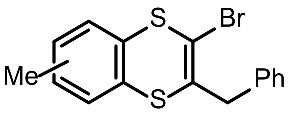

3c

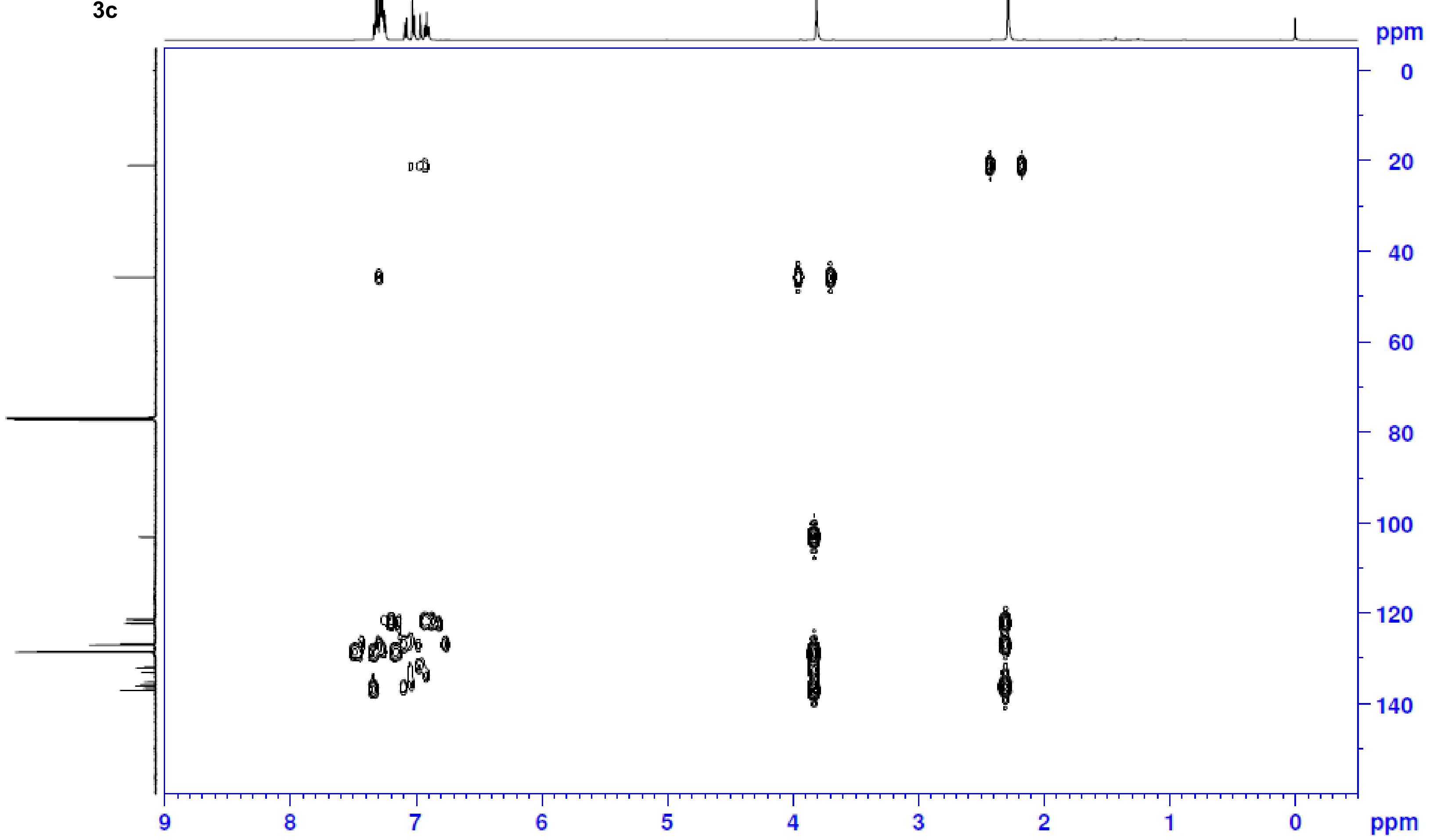

DL-236 gCOSY_CDCl3 + 0.03% TMS, 25 st. C

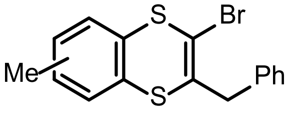

3c

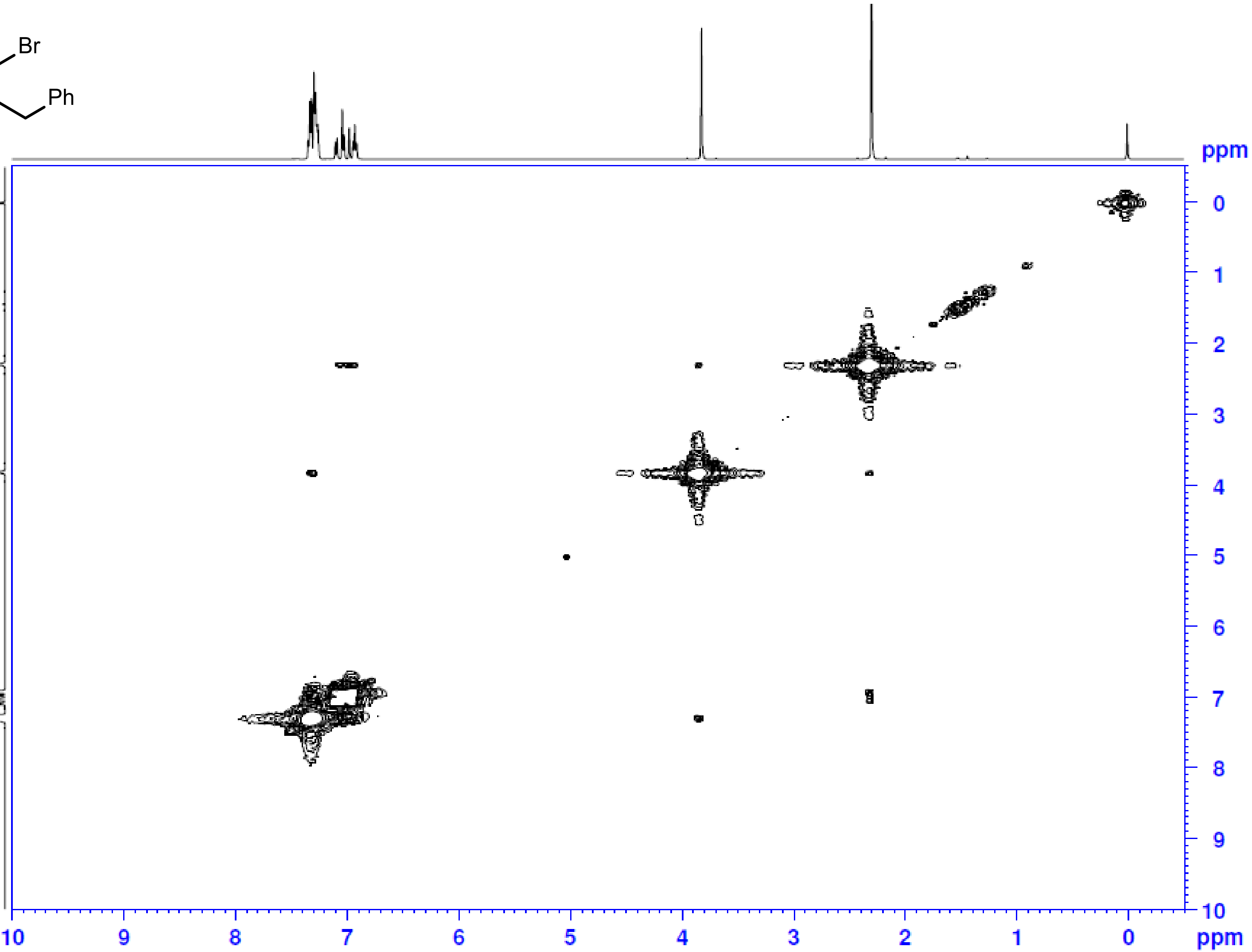

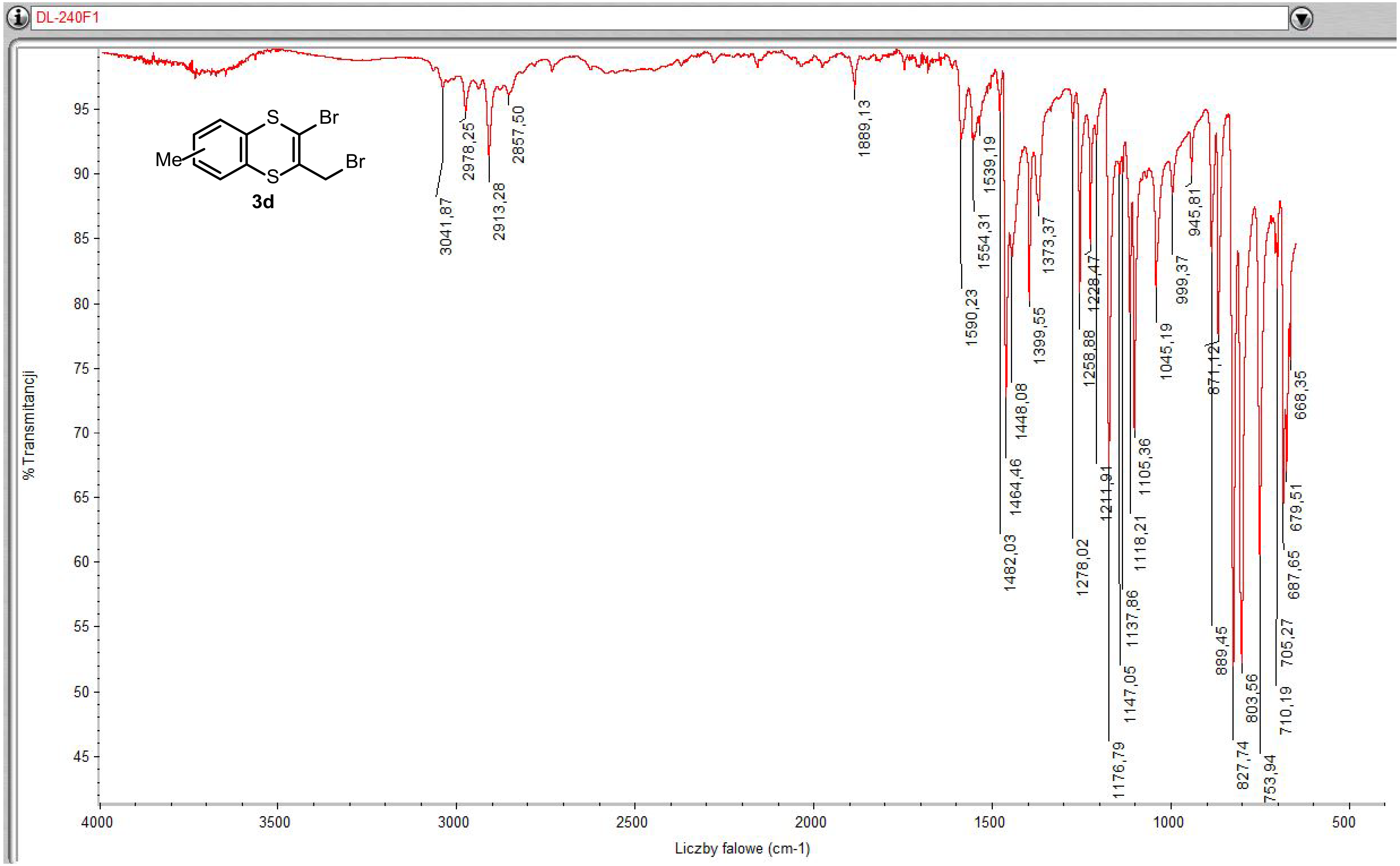
DL-240F1
% Transmitancji
Liczby falowe (cm-1)
3d
Me
S
Br
3041,87
2978,25
2913,28
2857,50
1889,13
1590,23
1554,31
1539,19
1482,03
1464,46
1448,08
1399,55
1373,37
1278,02
1258,88
1228,47
1211,91
1176,79
1147,05
1137,86
1118,21
1105,36
1045,19
999,37
945,81
889,45
871,12
827,74
803,56
753,94
710,19
705,27
687,65
679,51
668,35

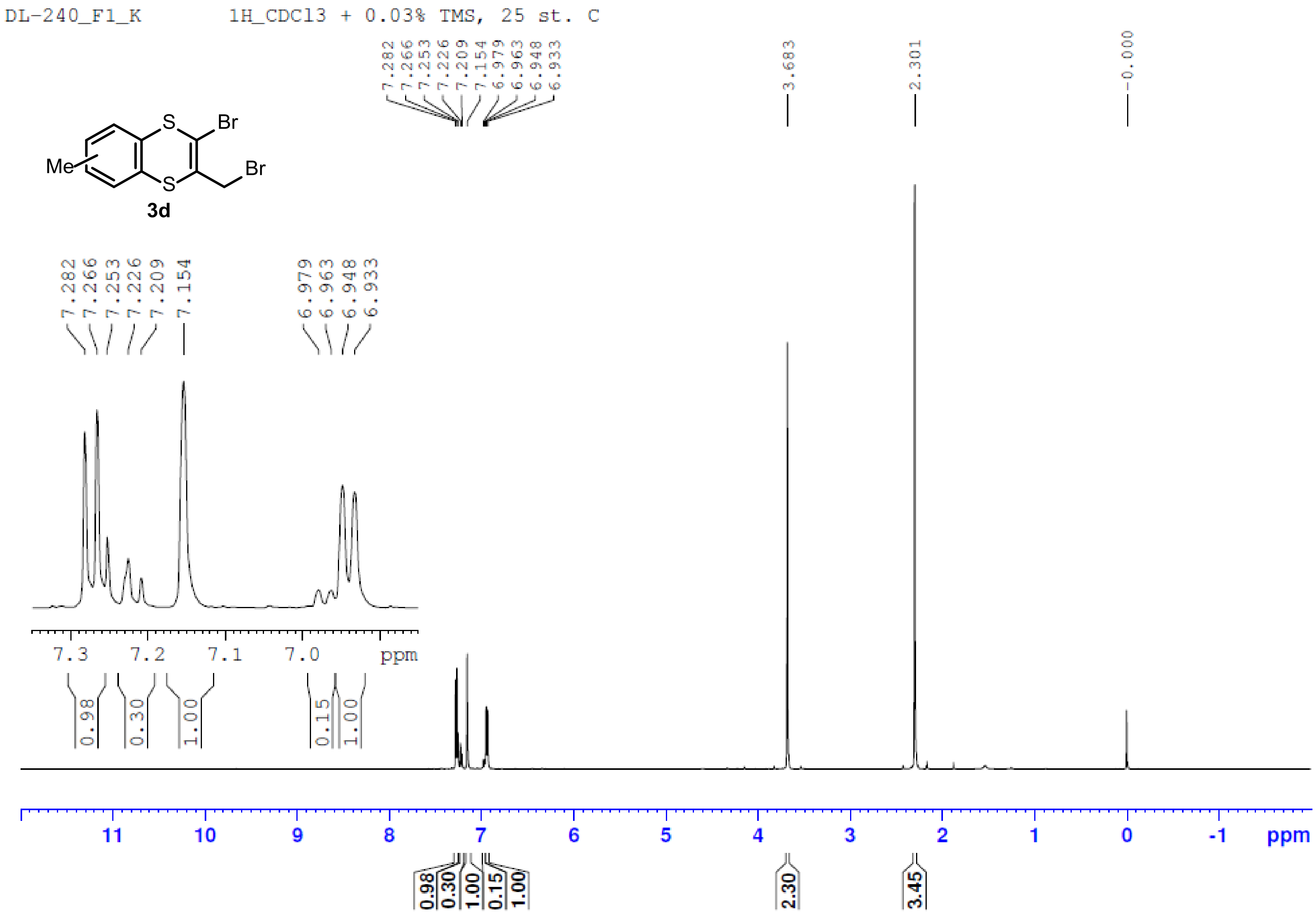

DL-240_F1_K 1H_CDCl3 + 0.03% TMS, 25 st. C
7.282
7.266
7.253
7.226
7.209
7.154
6.979
6.963
6.948
6.933
3.683
2.301
-0.000
S
Br
Me
S
Br
3d
7.3
7.2
7.1
7.0
ppm
0.98
0.30
1.00
0.15
1.00
11
10
9
8
7
6
5
4
3
2
1
0
-1
ppm
2.30
3.45

DL-240_F1_K_K 13C_CDCl3 + 0.03% TMS, 25 st. C

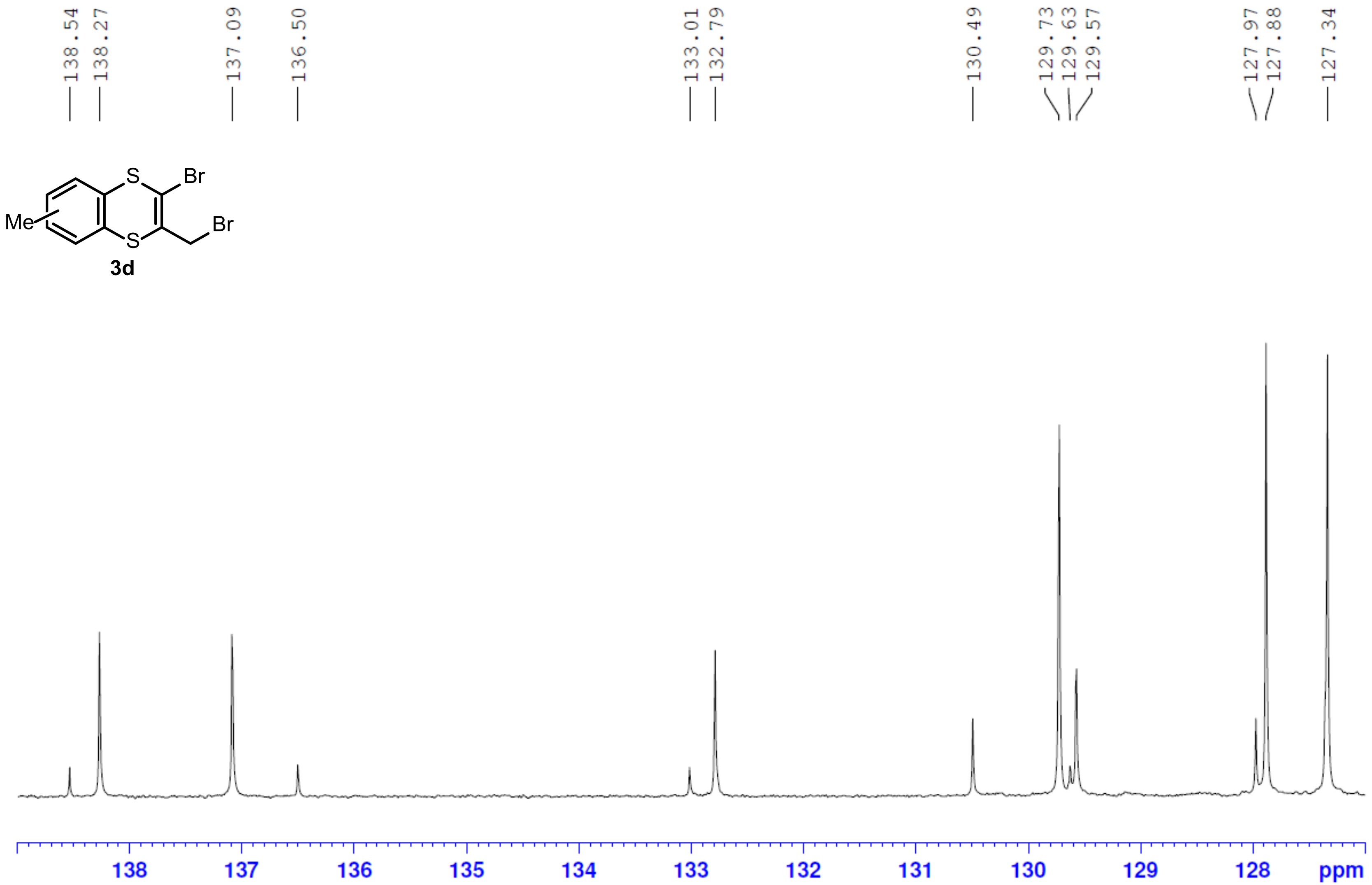

DL-240_F1_K_K 13C_CDCl3 + 0.03% TMS, 25 st. C

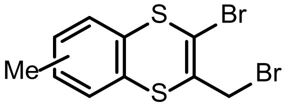

3d

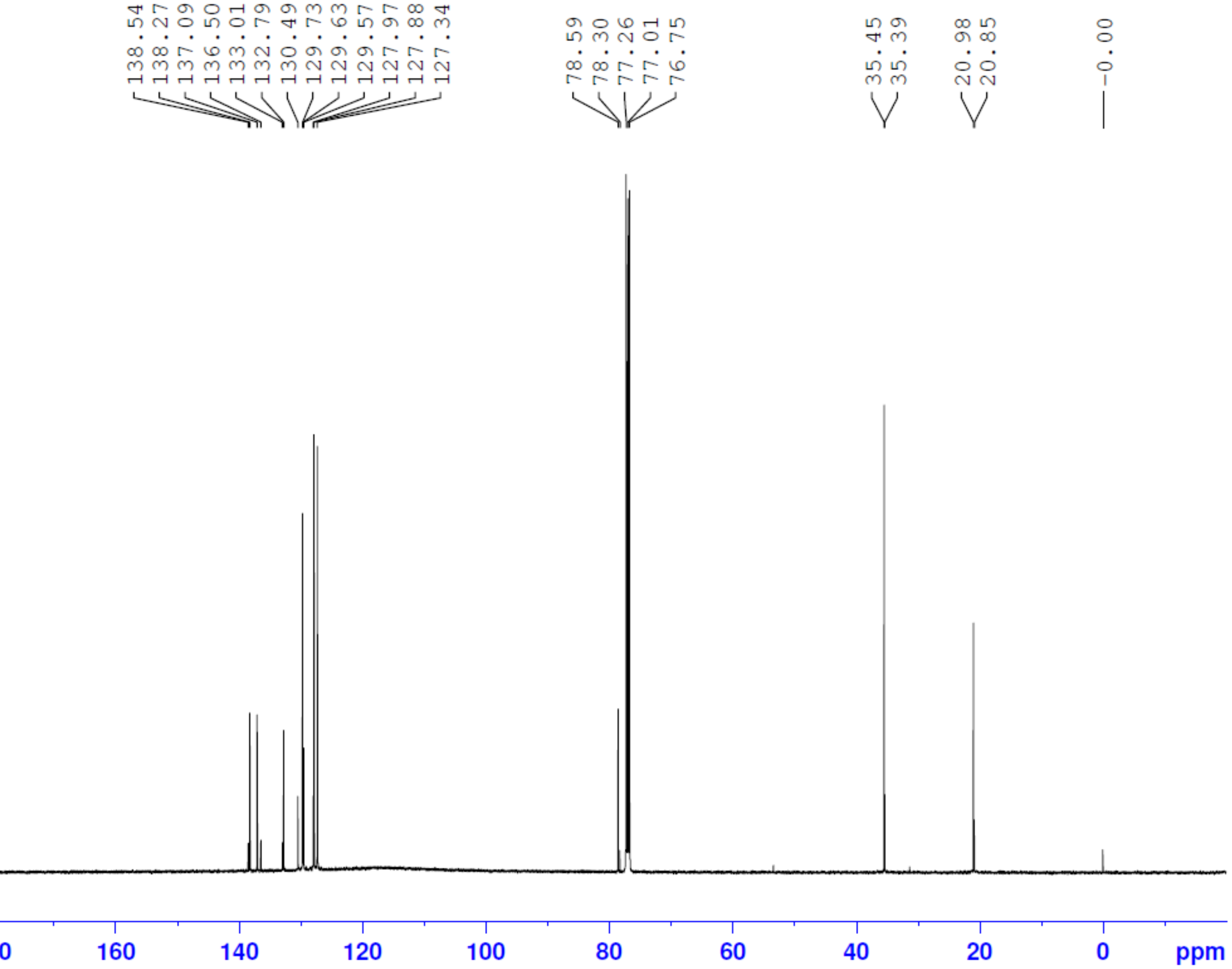

DL-240_F1_K_K gCOSY_CDCl3 + 0.03% TMS, 25 st. C

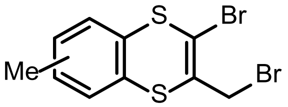

3d

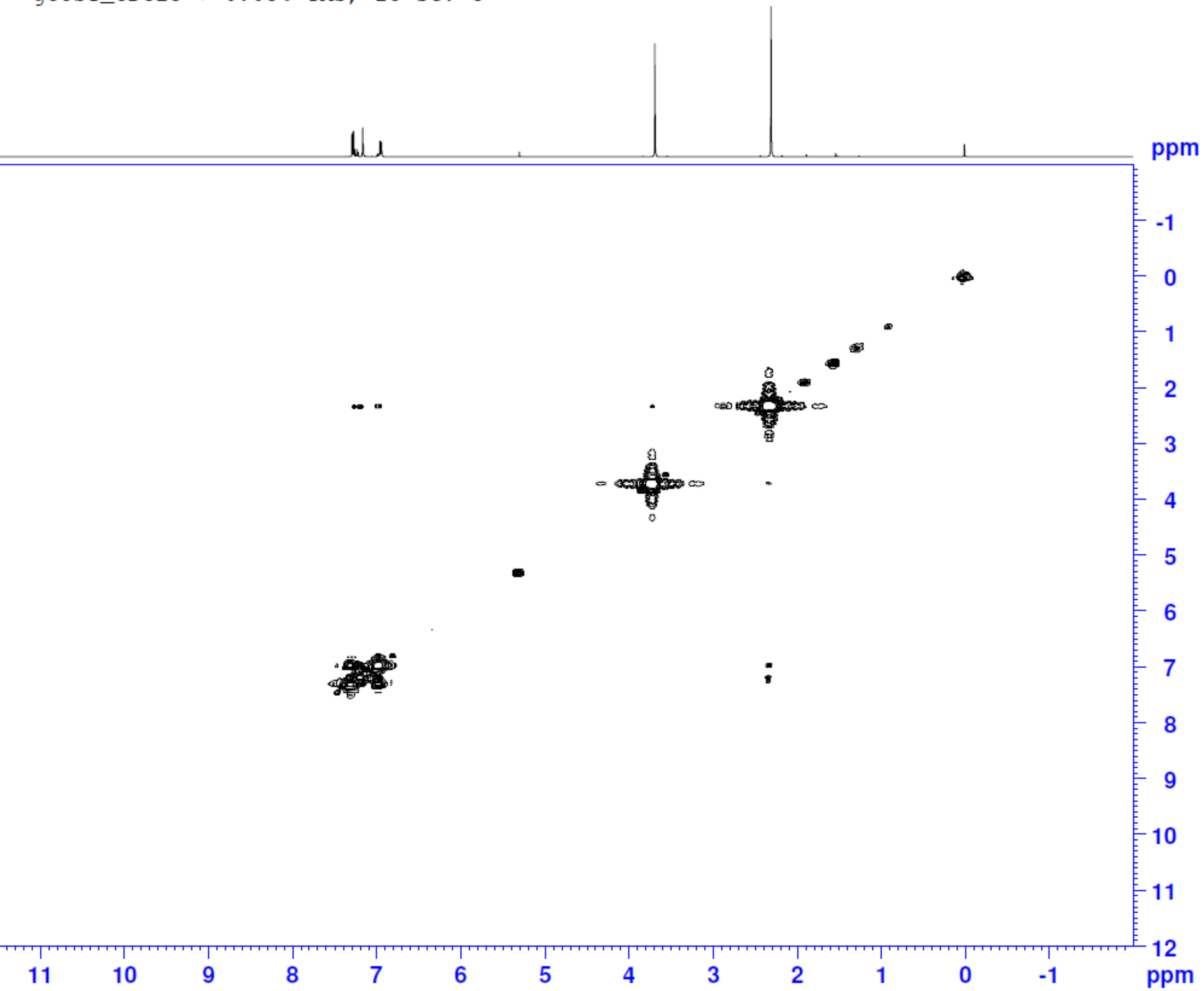

DL-240_F1_K_K gHSQC_CDCl3 + 0.03% TMS, 25 st. C

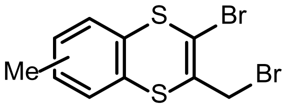

3d

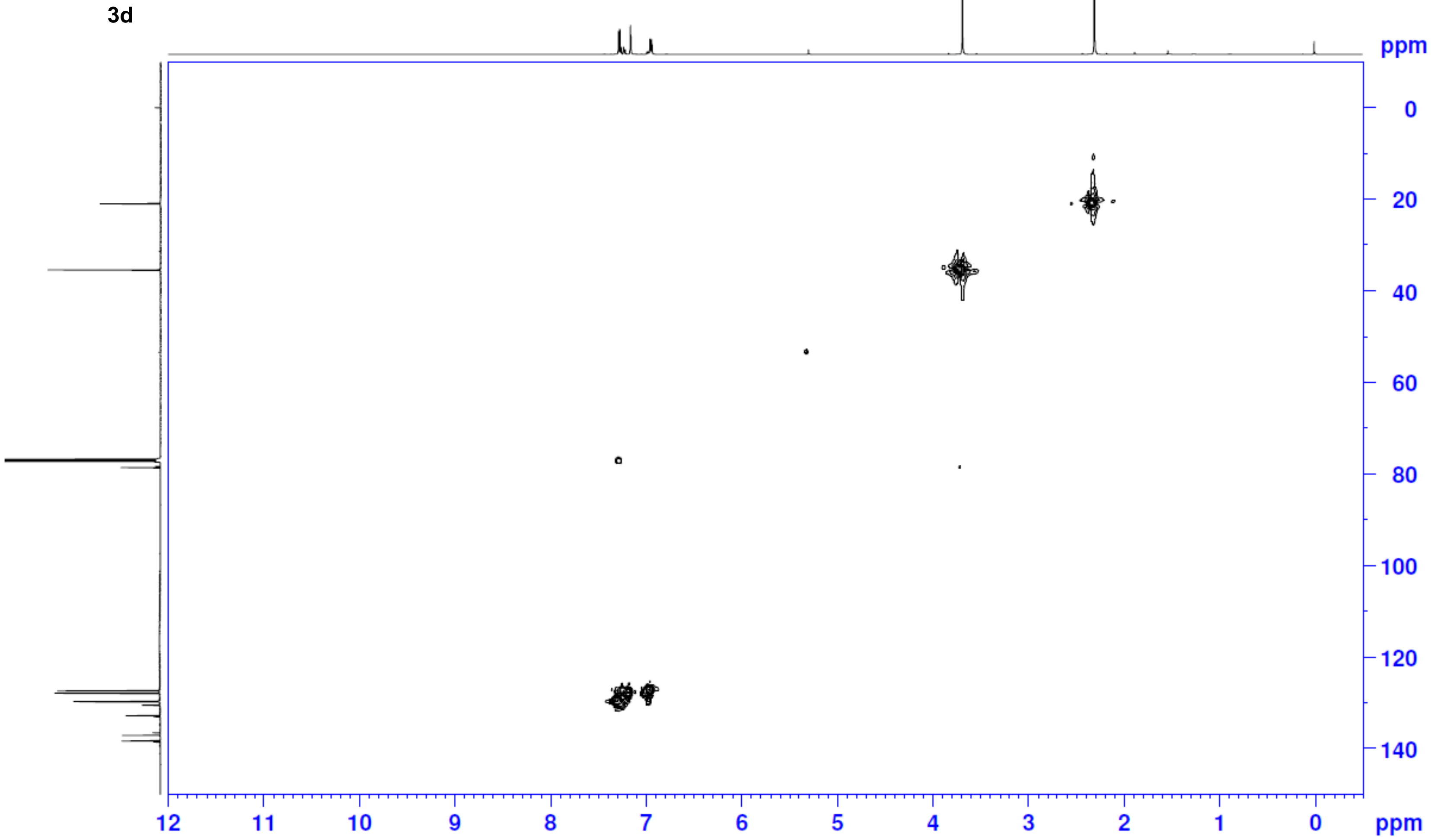

DL-240_F1_K_K gHMBC_CDCl3 + 0.03% TMS, 25 st. C

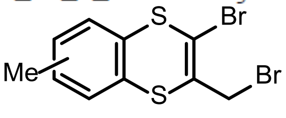

3d

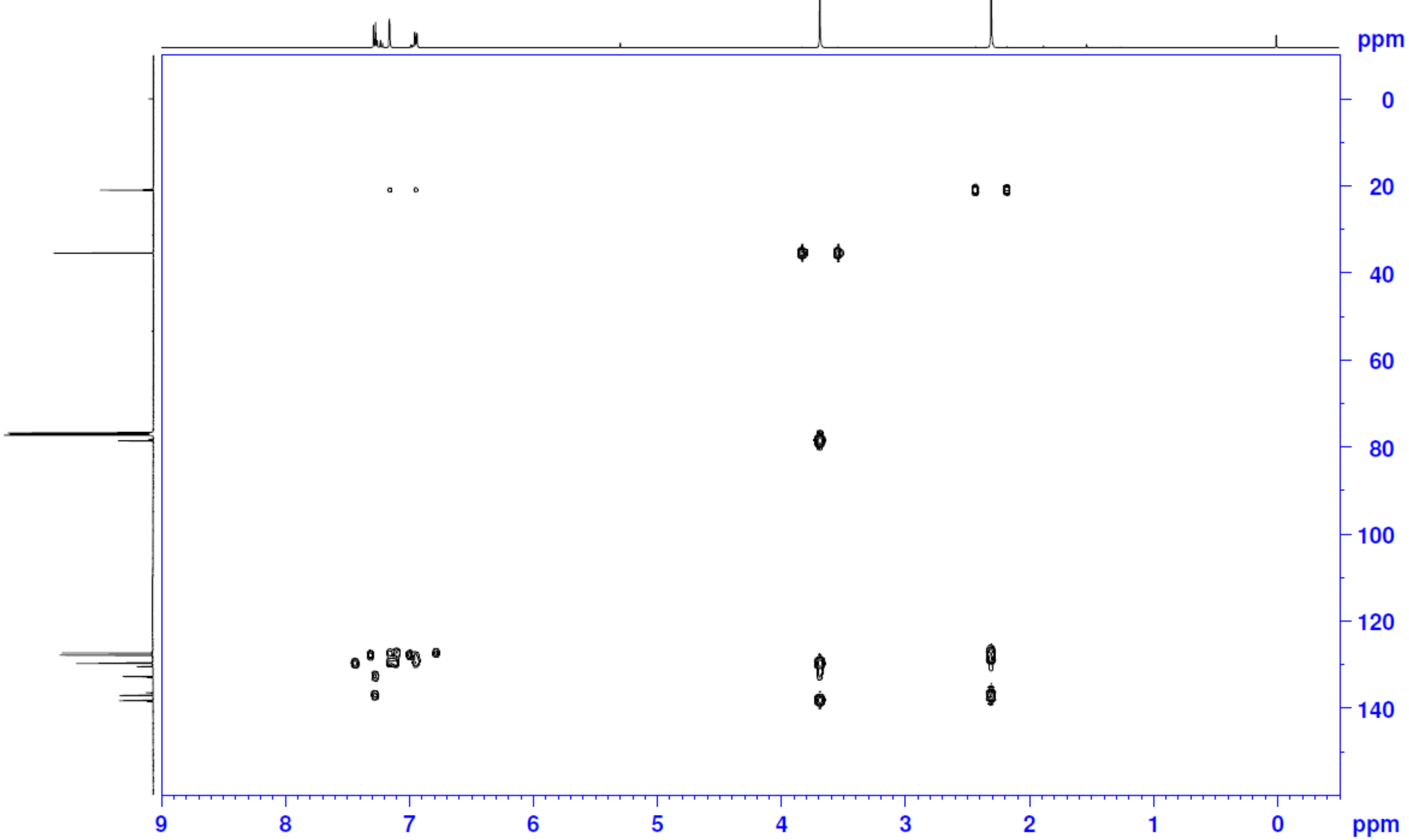

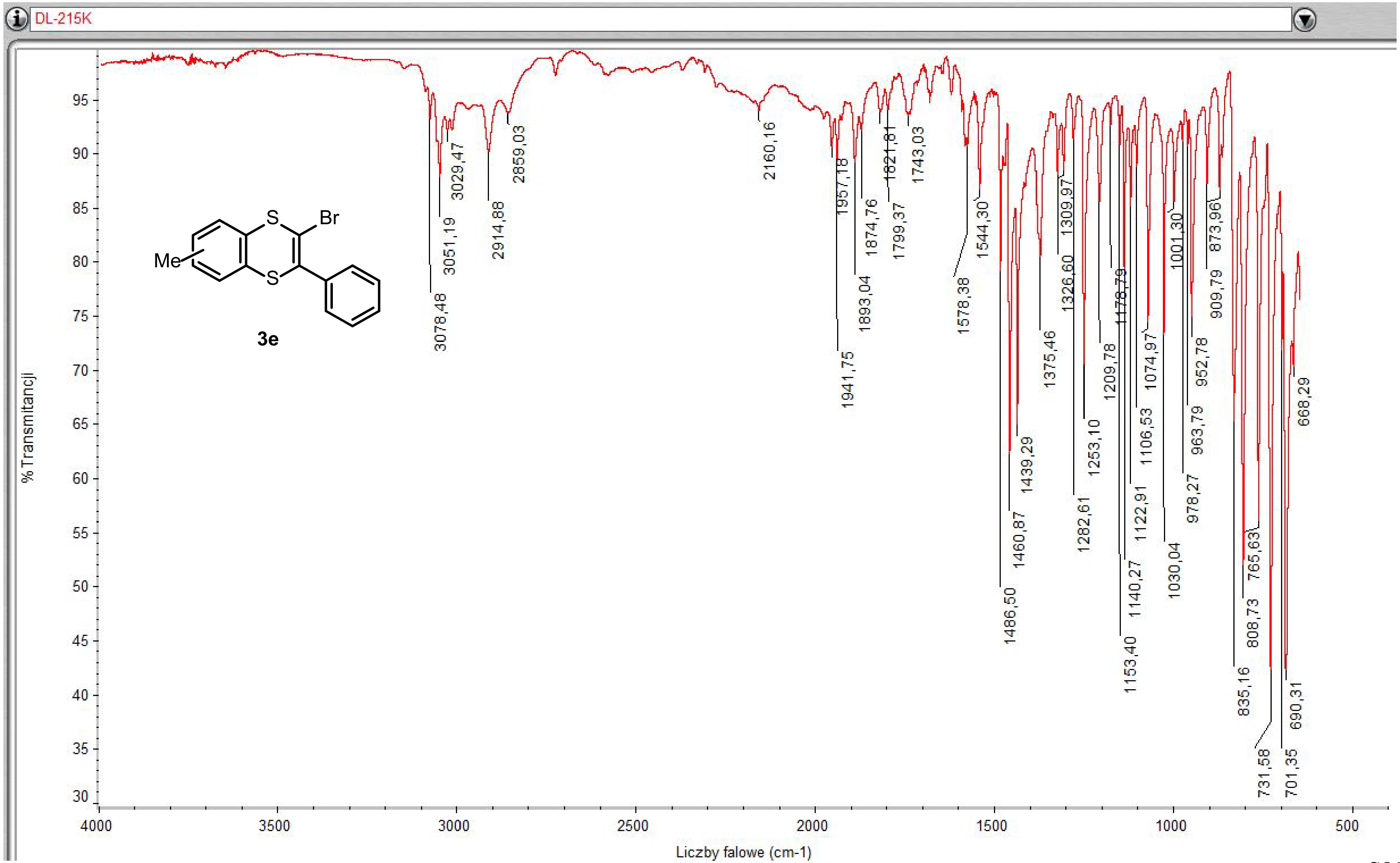
DL-215K
% Transmitancji
Liczby falowe (cm-1)
Me
S
Br
3e
3078,48
3051,19
3029,47
2914,88
2859,03
2160,16
1957,18
1941,75
1893,04
1874,76
1821,81
1799,37
1743,03
1578,38
1544,30
1486,50
1460,87
1439,29
1375,46
1326,60
1309,97
1282,61
1253,10
1209,78
1178,79
1153,40
1140,27
1122,91
1106,53
1074,97
1030,04
1001,30
978,27
963,79
952,78
909,79
873,96
835,16
808,73
765,63
731,58
701,35
690,31
668,29

DL-215_K2 1H_CDCl3 + 0.03% TMS, 25 st. C

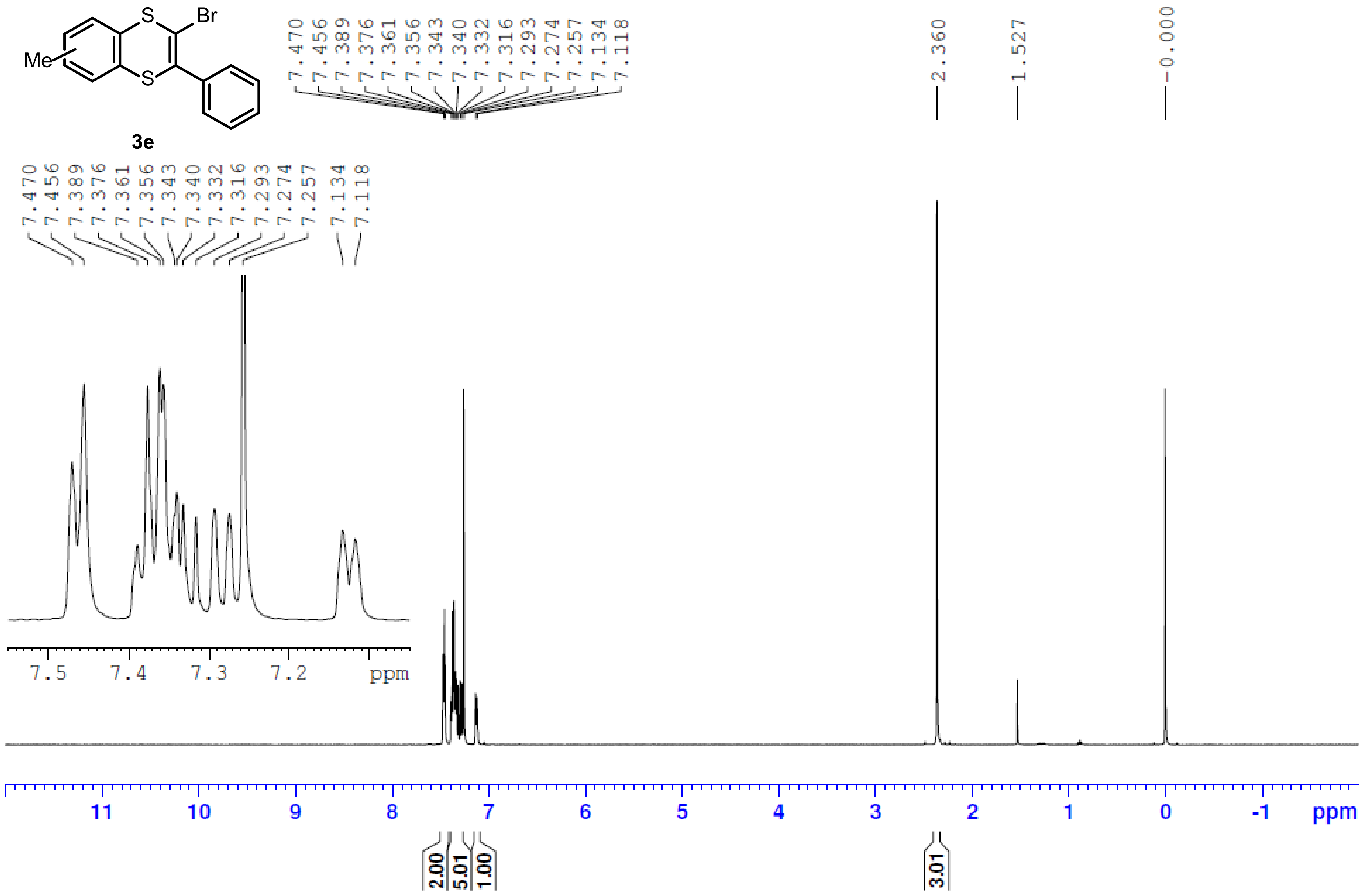

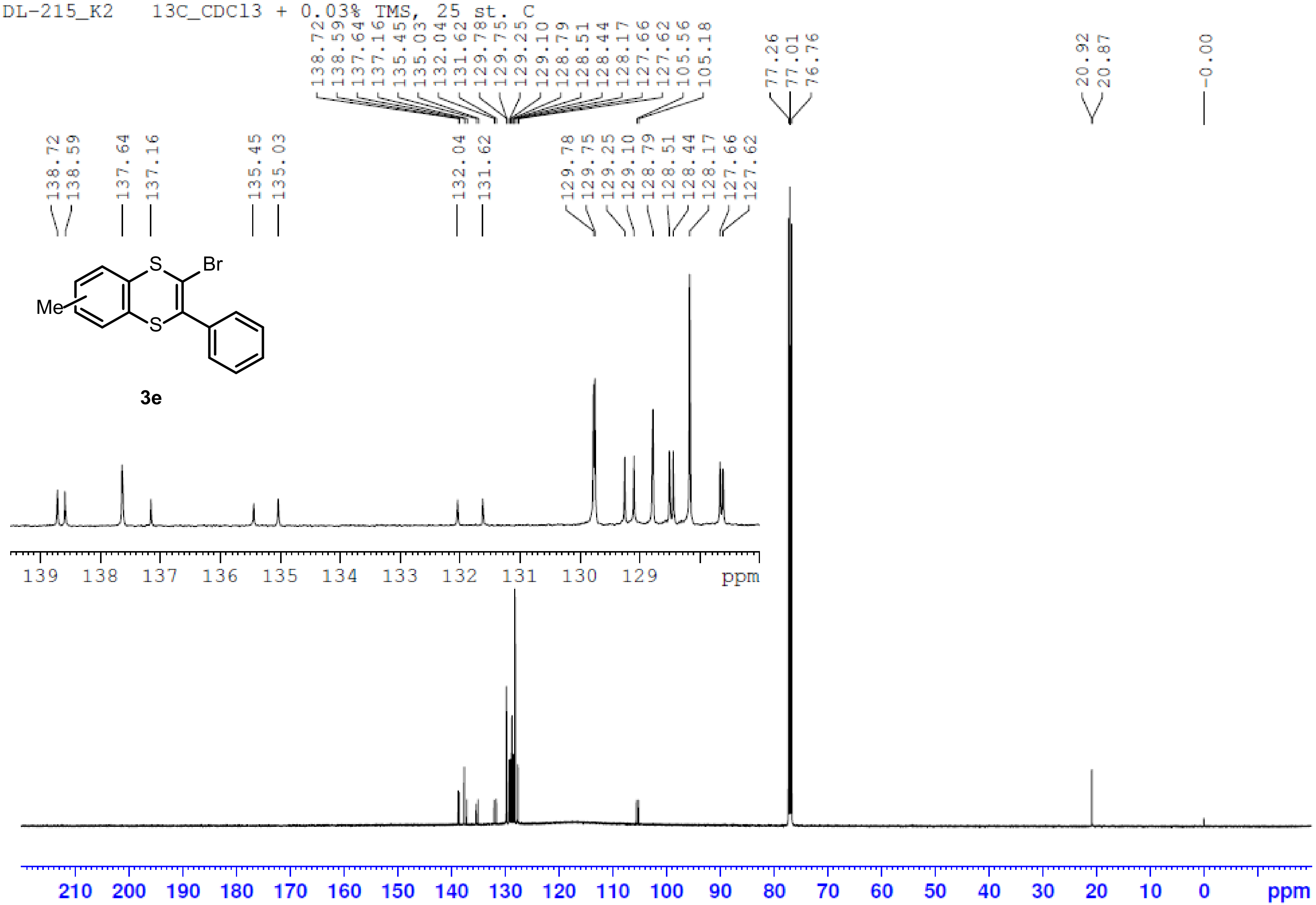
DL-215_K2 13C_CDCl3 + 0.03% TMS, 25 st. C
138.72 138.59 137.64 137.16 135.45 135.03 132.04 131.62 129.78 129.75 129.25 129.10 128.79 128.51 128.44 128.17 127.66 127.62 105.56 105.18
77.26 77.01 76.76
20.92 20.87
-0.00
S
Br
Me
S
ppm
210 200 190 180 170 160 150 140 130 120 110 100 90 80 70 60 50 40 30 20 10 0 ppm
3e

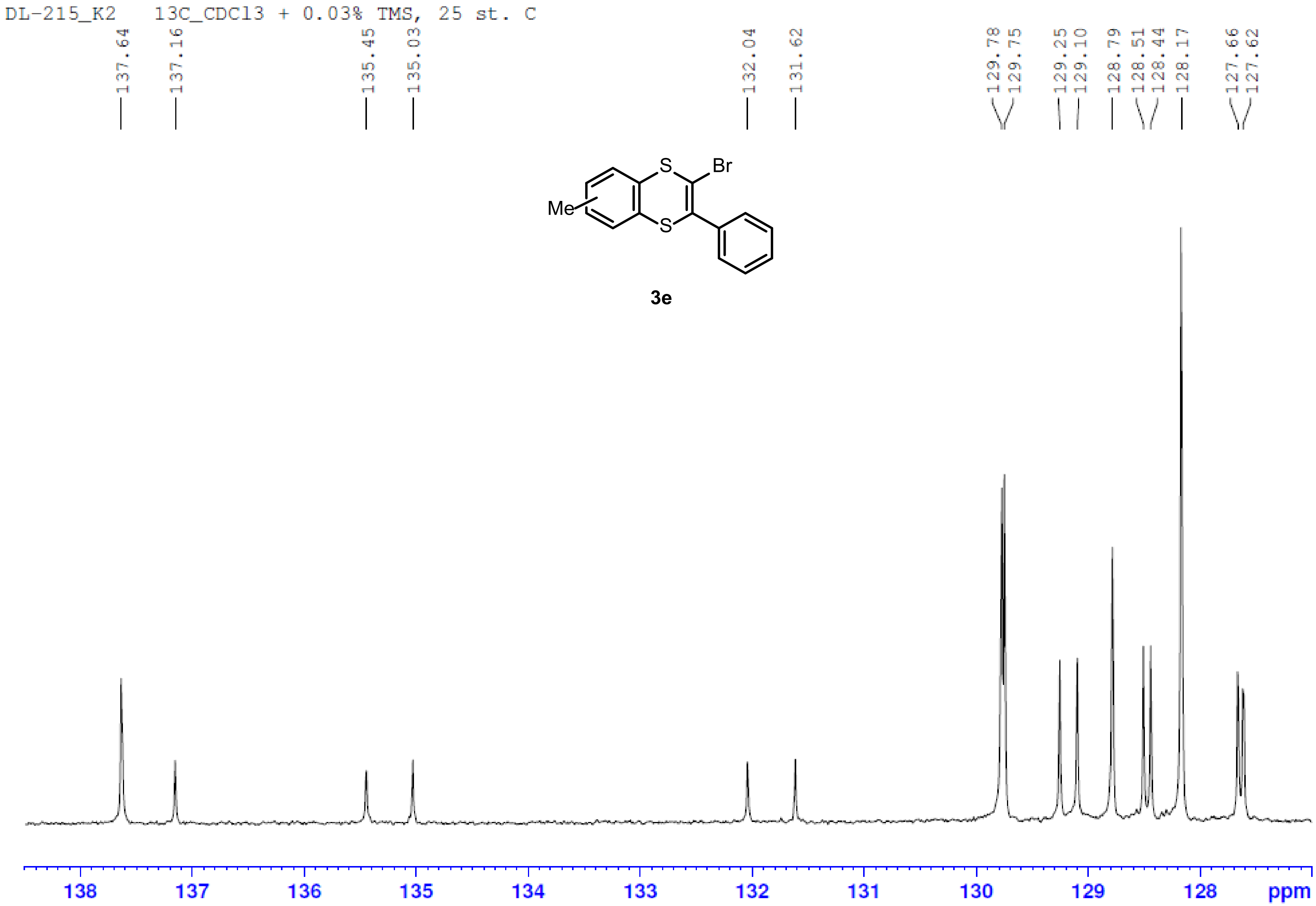
DL-215_K2 13C_CDCl3 + 0.03% TMS, 25 st. C
137.64
137.16
135.45
135.03
132.04
131.62
129.78
129.75
129.25
129.10
128.79
128.51
128.44
128.17
127.66
127.62
Me
S
S
Br
3e
138
137
136
135
134
133
132
131
130
129
128
ppm

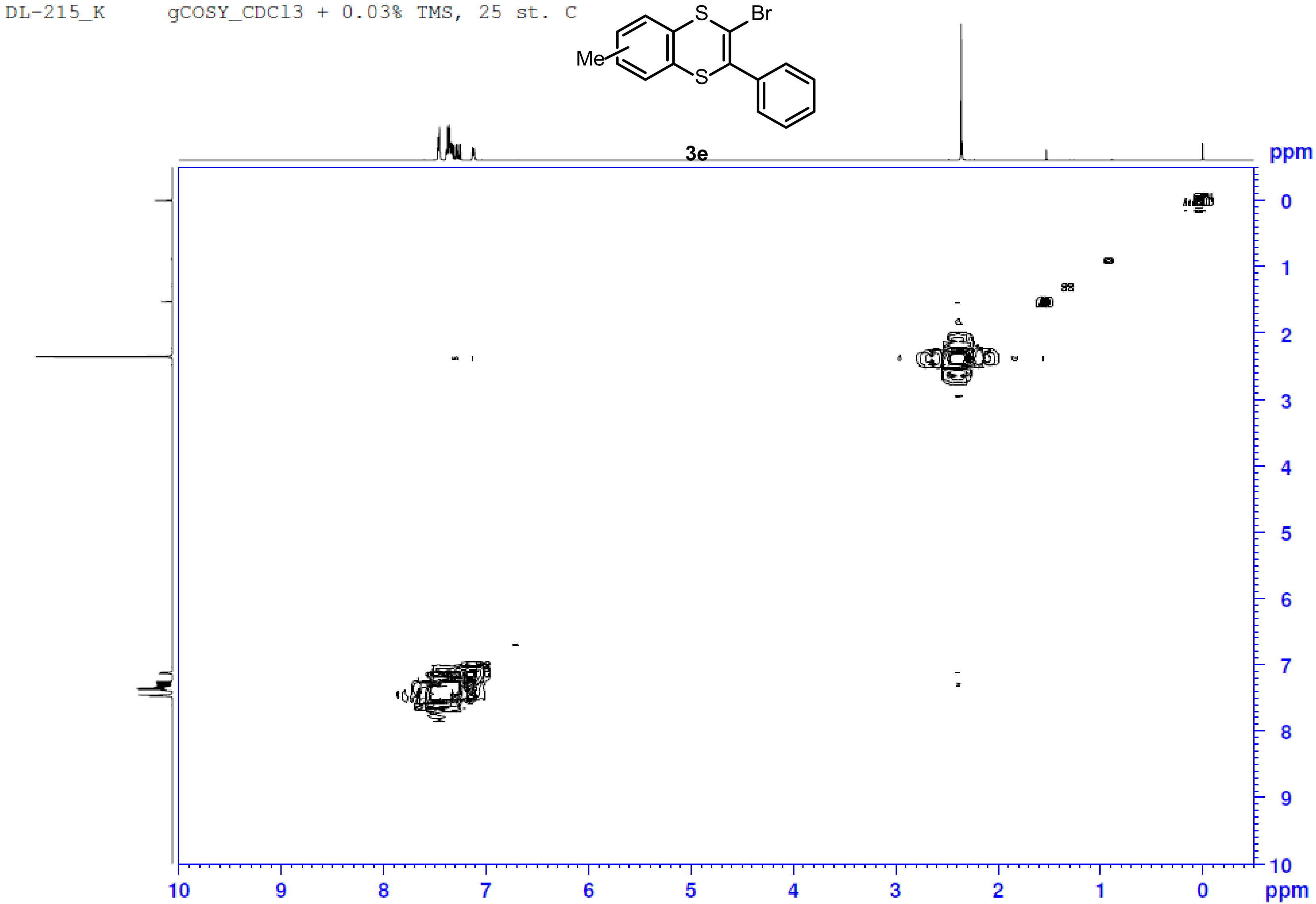
DL-215_K gCOSY_CDCl3 + 0.03% TMS, 25 st. C
S
Br
Me
S
3e
ppm
ppm

DL-237_F1 1H_CDCl3 + 0.03% TMS, 25 st. C

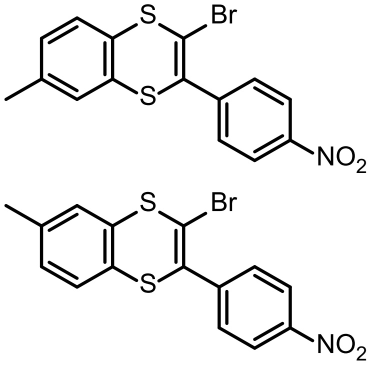

3f

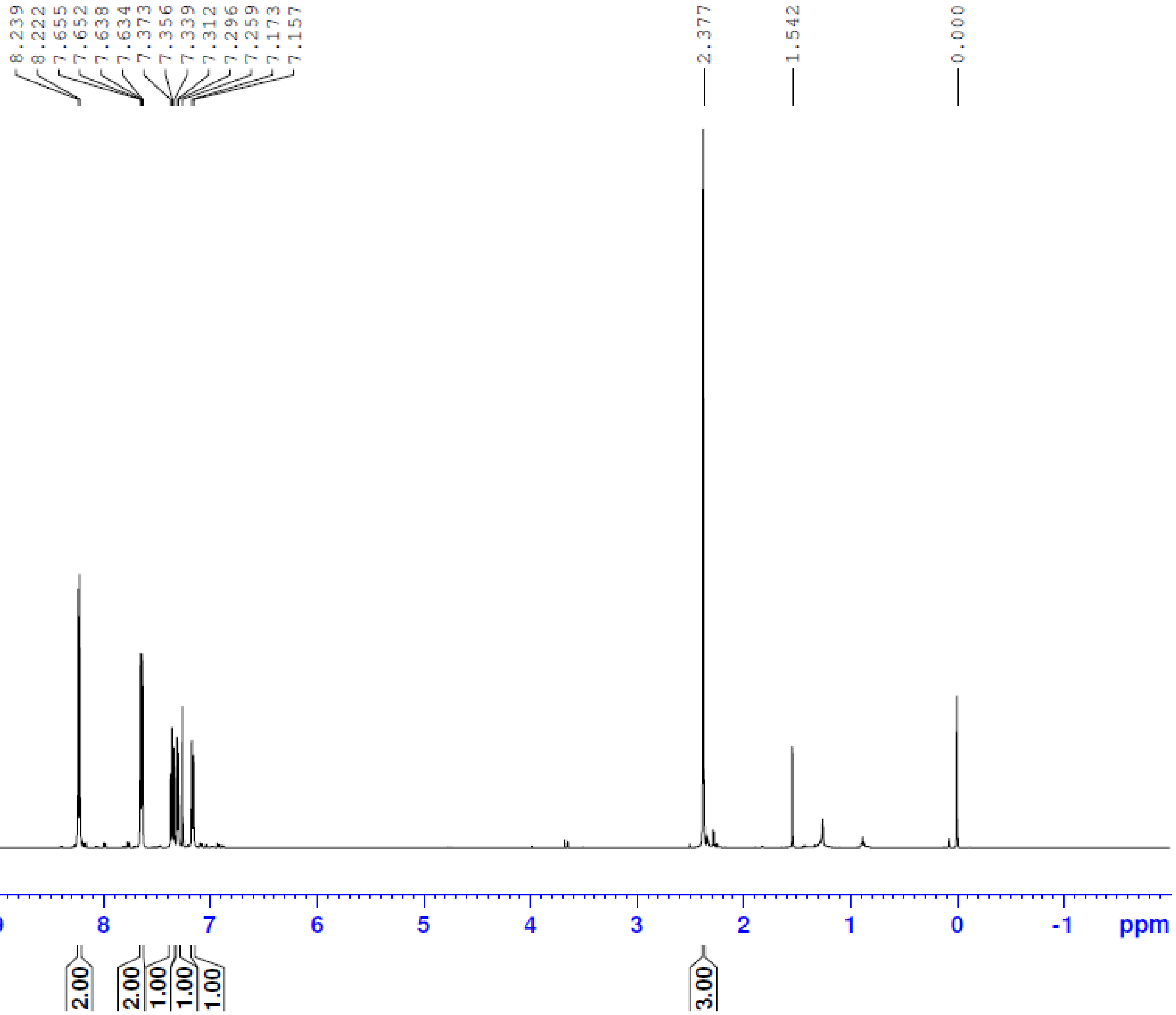

DL-237_F1 1H_CDCl3 + 0.03% TMS, 25 st. C

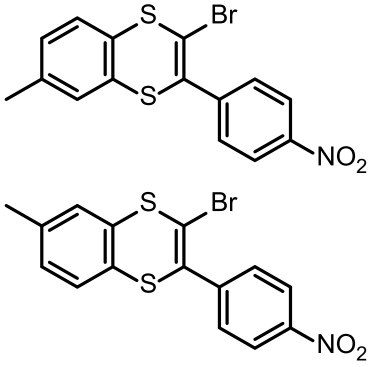

3f

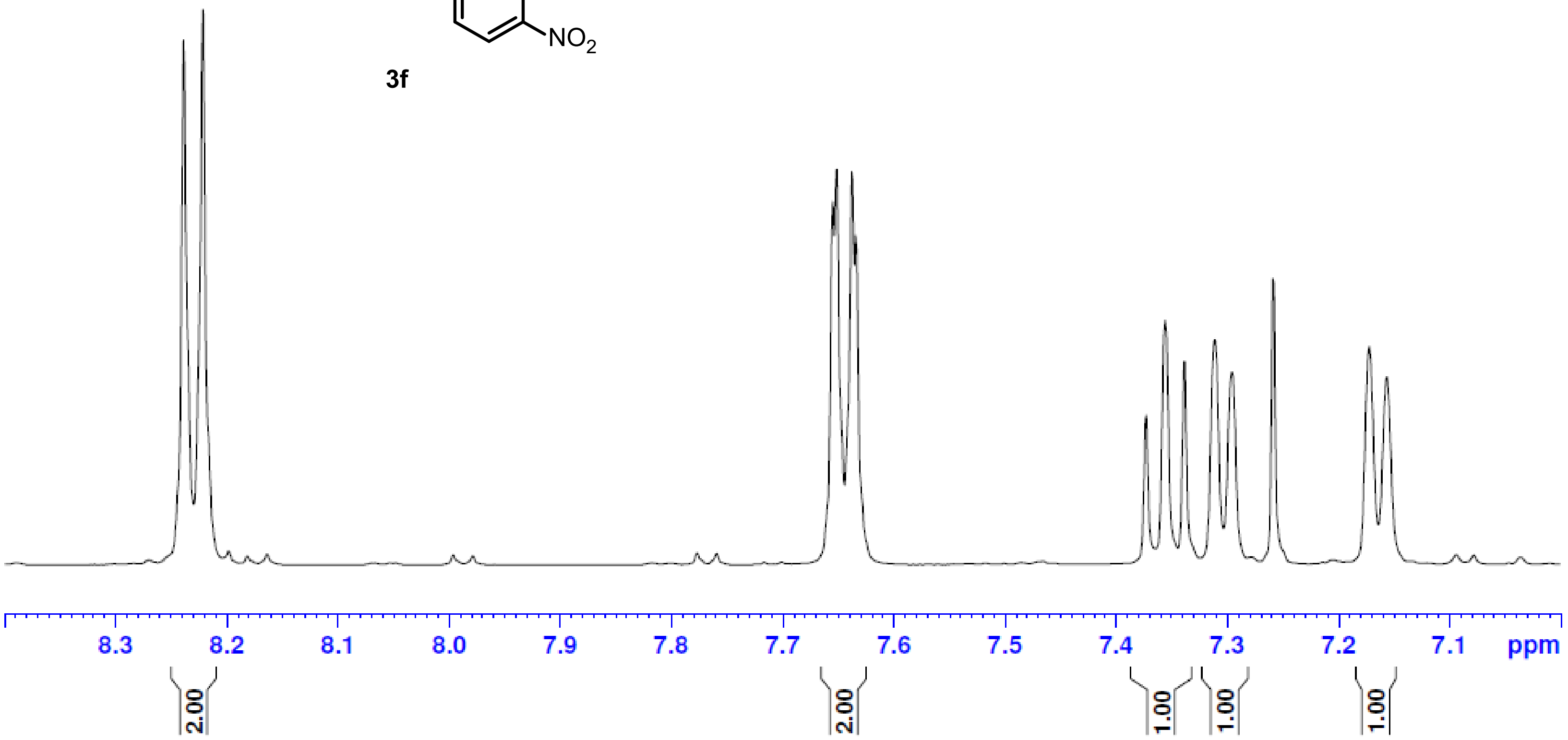

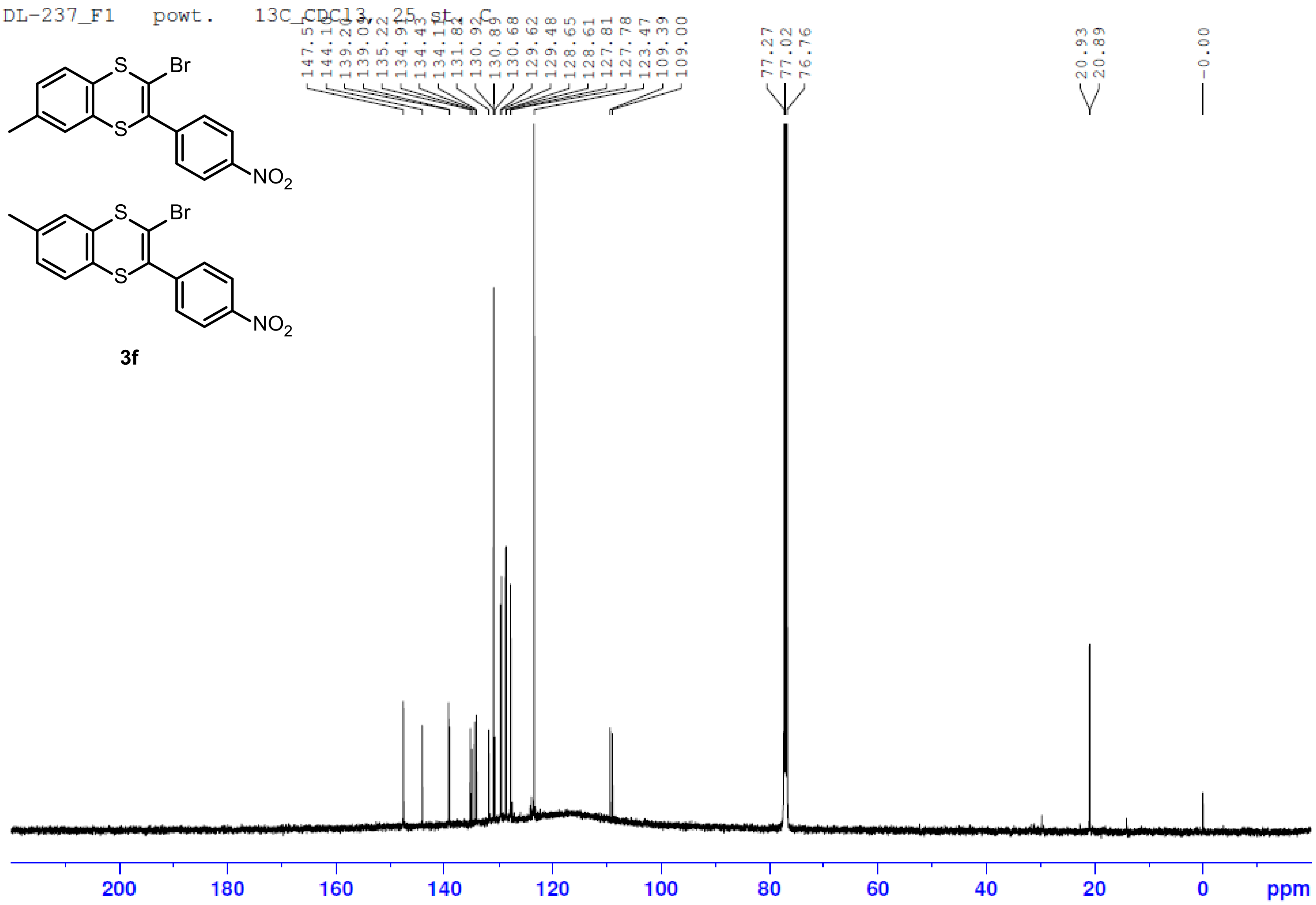
DL-237_F1 powt. 13C_CDCl3, 25 st. C
135.22
134.43
131.82
130.92
130.89
130.68
129.62
129.48
128.65
128.61
127.81
127.78
123.47
109.39
109.00
77.27
77.02
76.76
20.93
20.89
-0.00
S
Br
S
NO2
3f
200
180
160
140
120
100
80
60
40
20
0
ppm

DL-237_F1 powt. 13C_CDCl3, 25 st. C

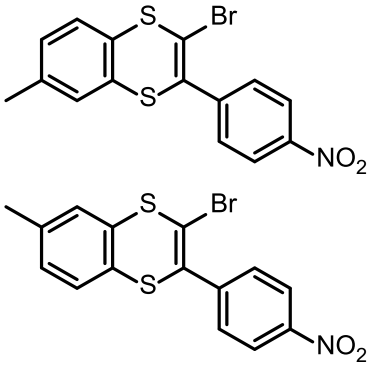

3f

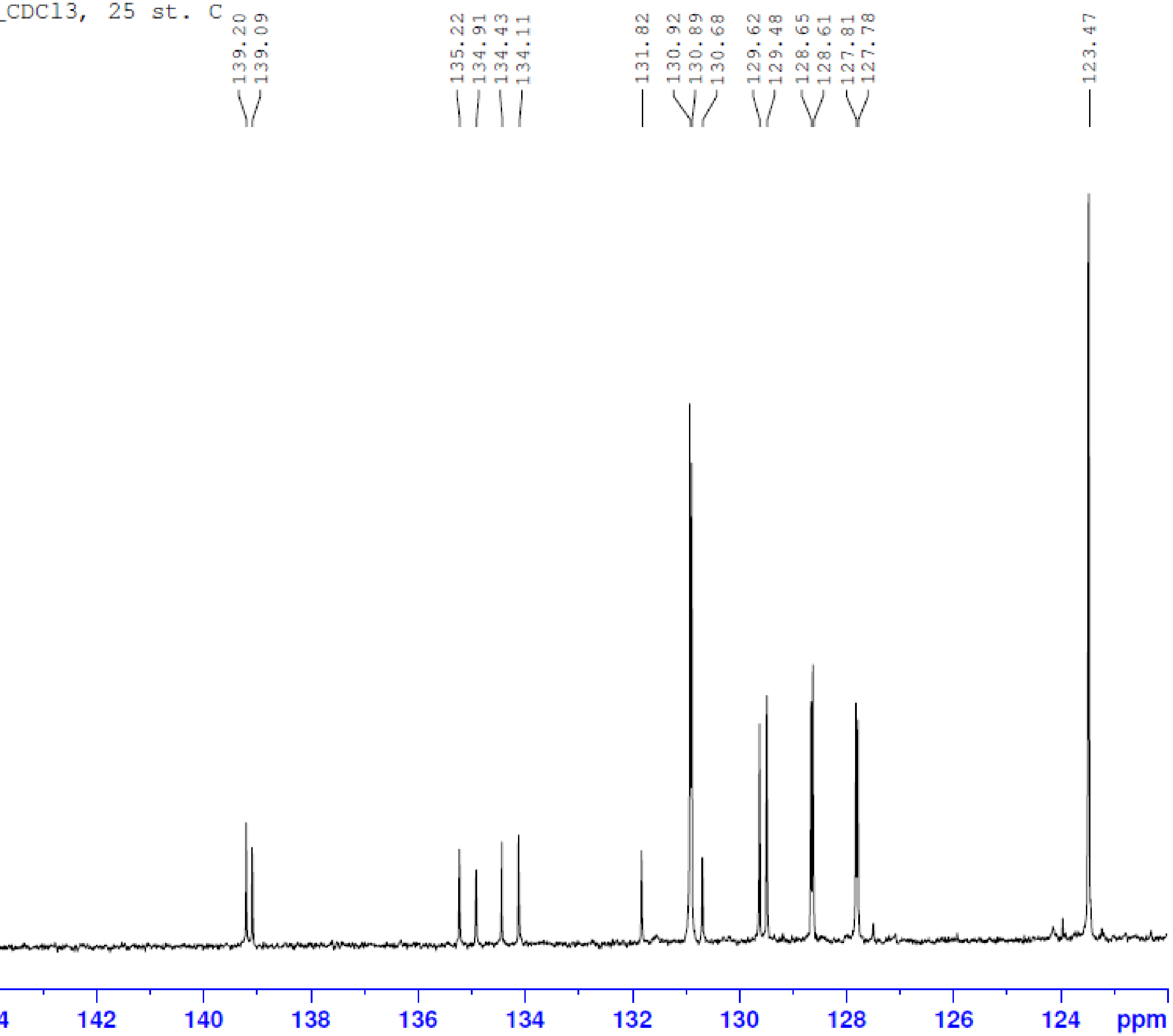

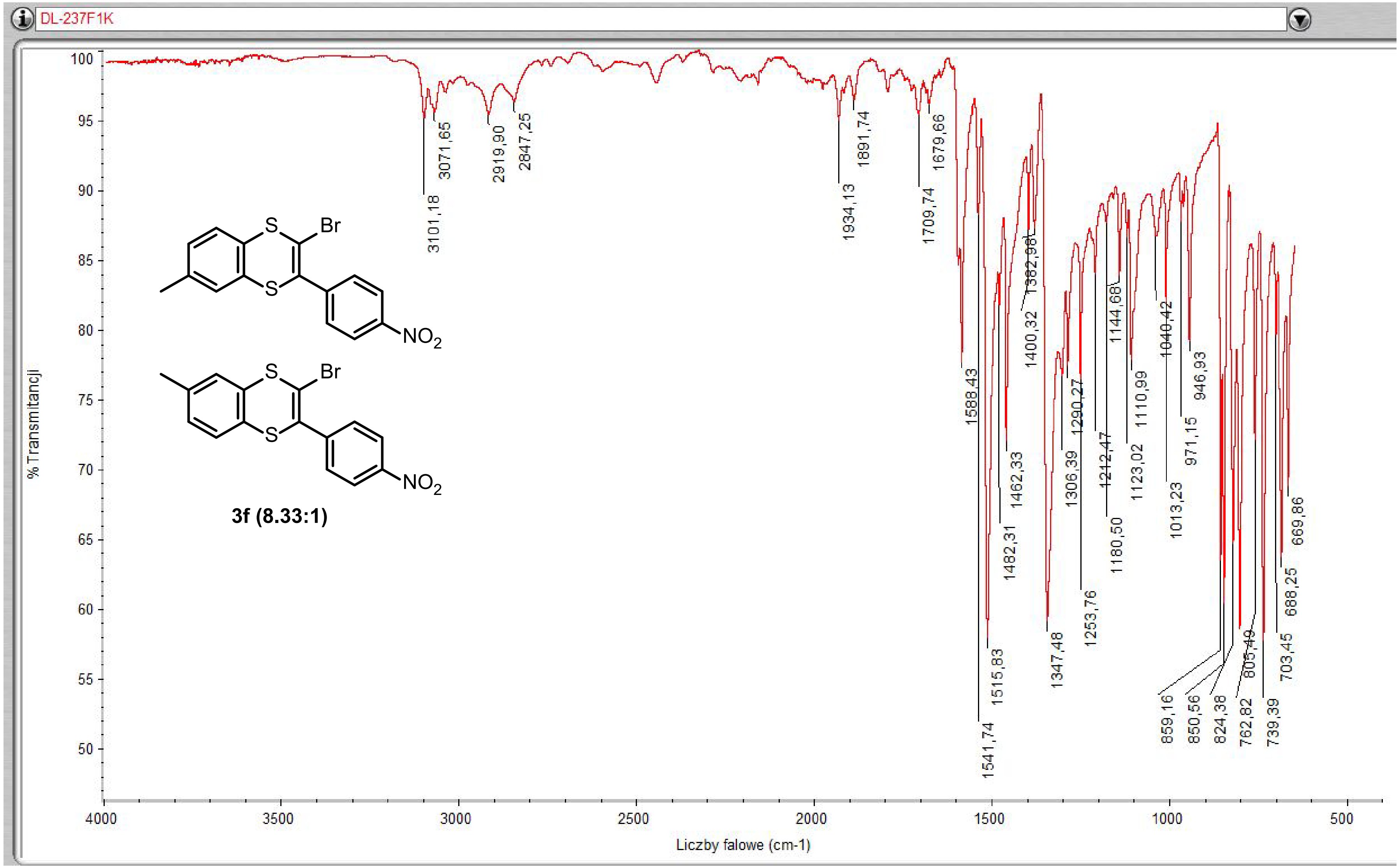
DL-237F1K
% Transmitancji
Liczby falowe (cm-1)
3101,18
3071,65
2919,90
2847,25
1934,13
1891,74
1709,74
1679,66
1588,43
1541,74
1515,83
1482,31
1462,33
1400,32
1382,98
1347,48
1306,39
1290,27
1253,76
1212,47
1180,50
1144,68
1123,02
1110,99
1040,42
1013,23
971,15
946,93
859,16
850,56
824,38
805,49
762,82
739,39
703,45
688,25
669,86
3f (8.33:1)

DL-237_F1_K   1H_CDCl3 + 0.03% TMS, 25 st. C

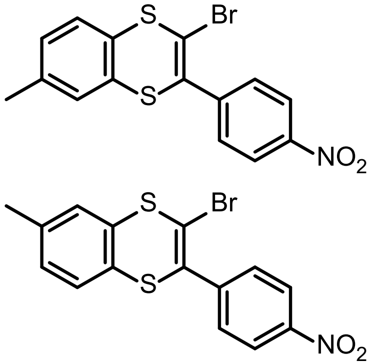

3f (8.33:1)

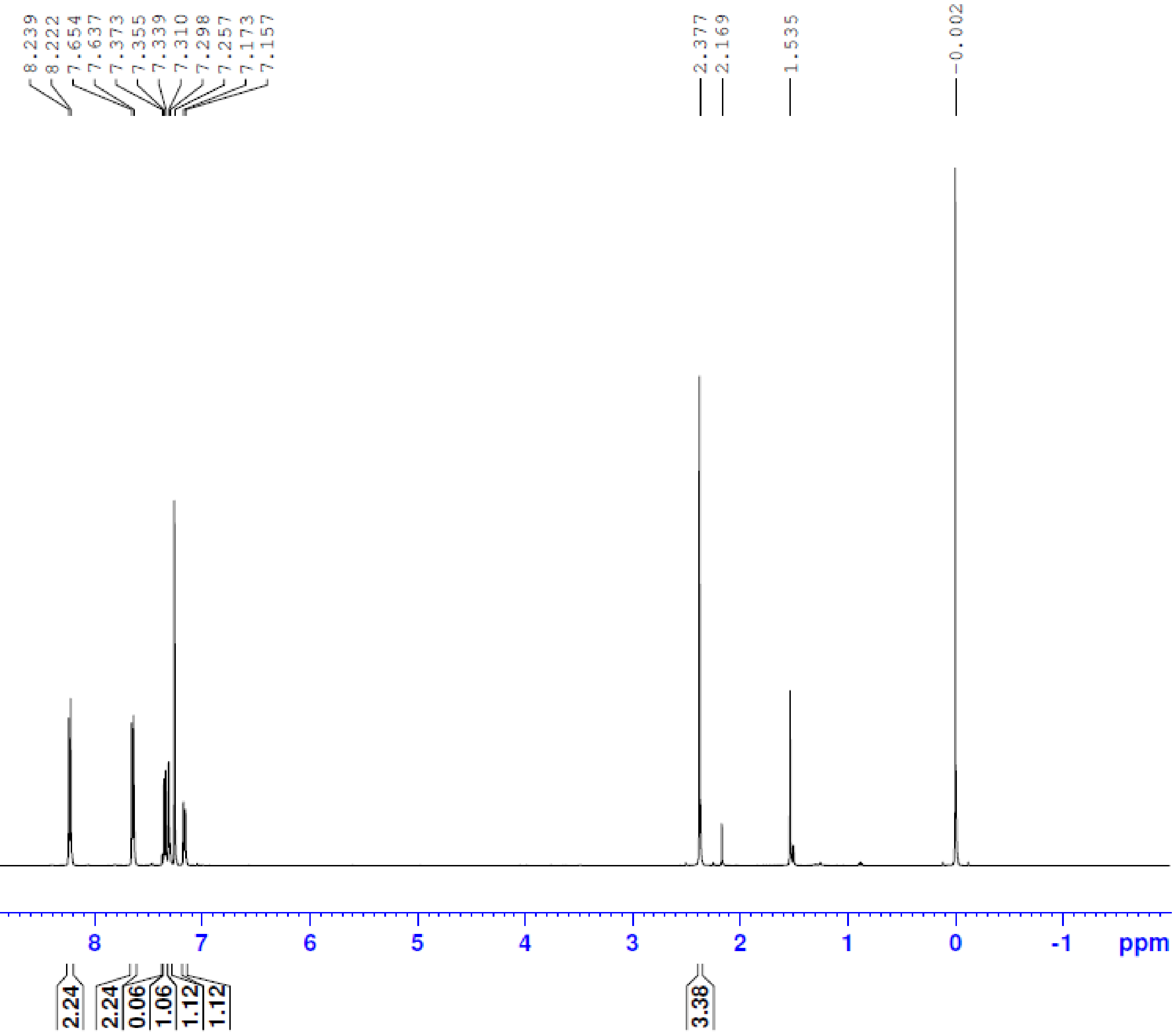

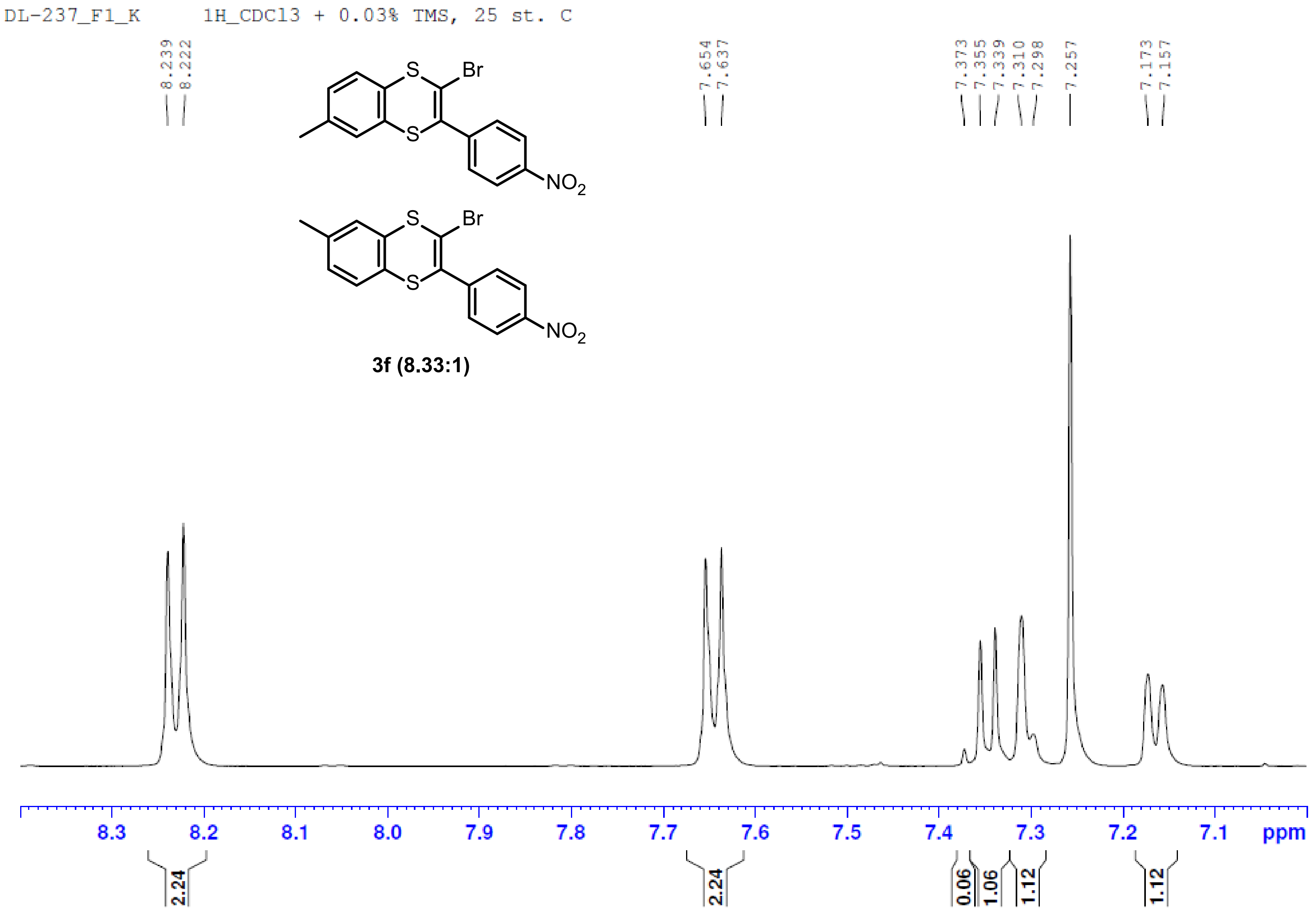
DL-237_F1_K 1H_CDCl3 + 0.03% TMS, 25 st. C
8.239
8.222
7.654
7.637
7.373
7.355
7.339
7.310
7.298
7.257
7.173
7.157
S
Br
S
NO2
8.3
8.2
8.1
8.0
7.9
7.8
7.7
7.6
7.5
7.4
7.3
7.2
7.1
ppm
2.24
2.24
0.06
1.06
1.12
1.12

3f (8.33:1)

DL-231_F1_K 13C_CDCl3 + 0.03% TMS, 25 st. C

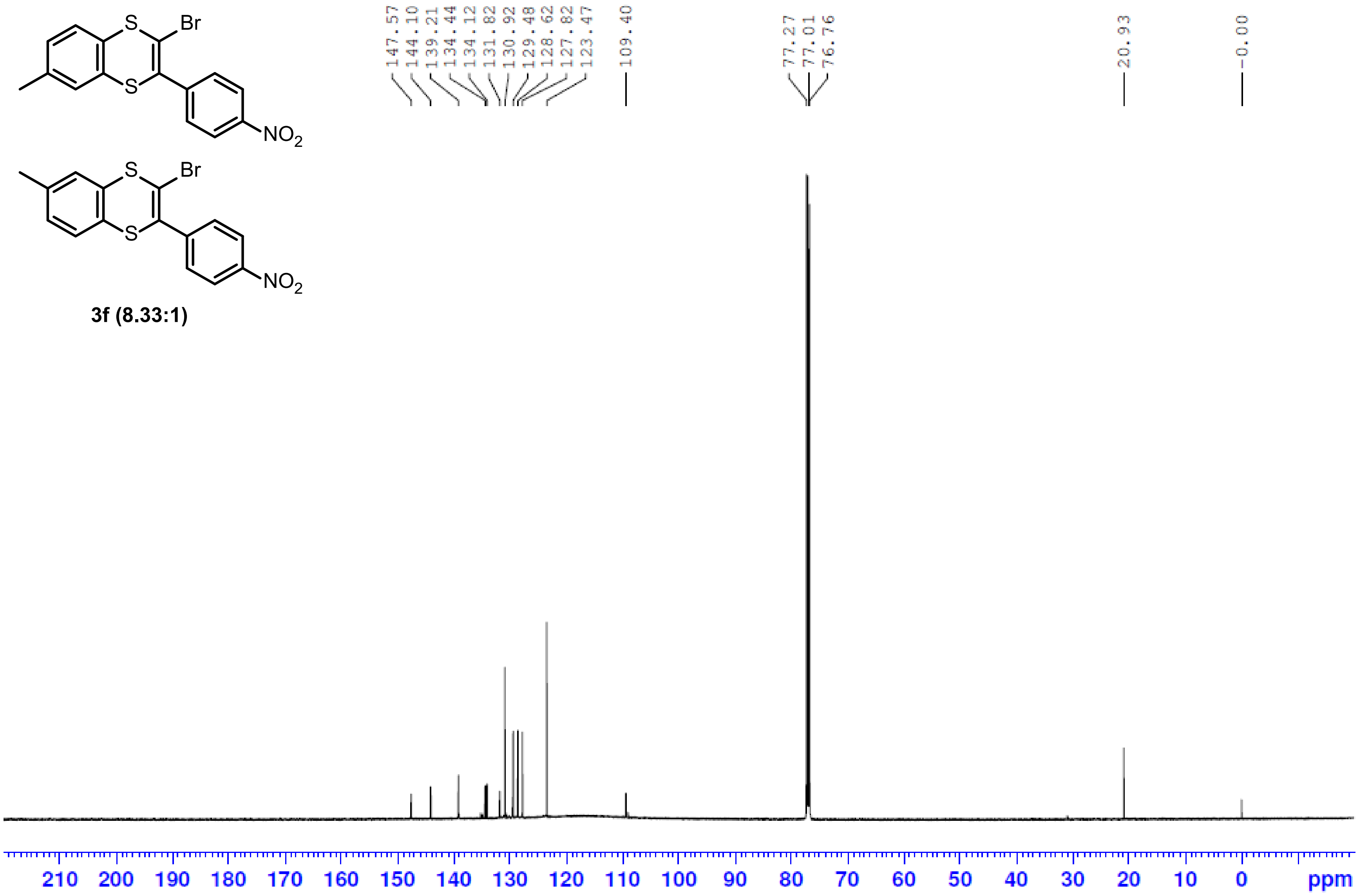

3f (8.33:1)

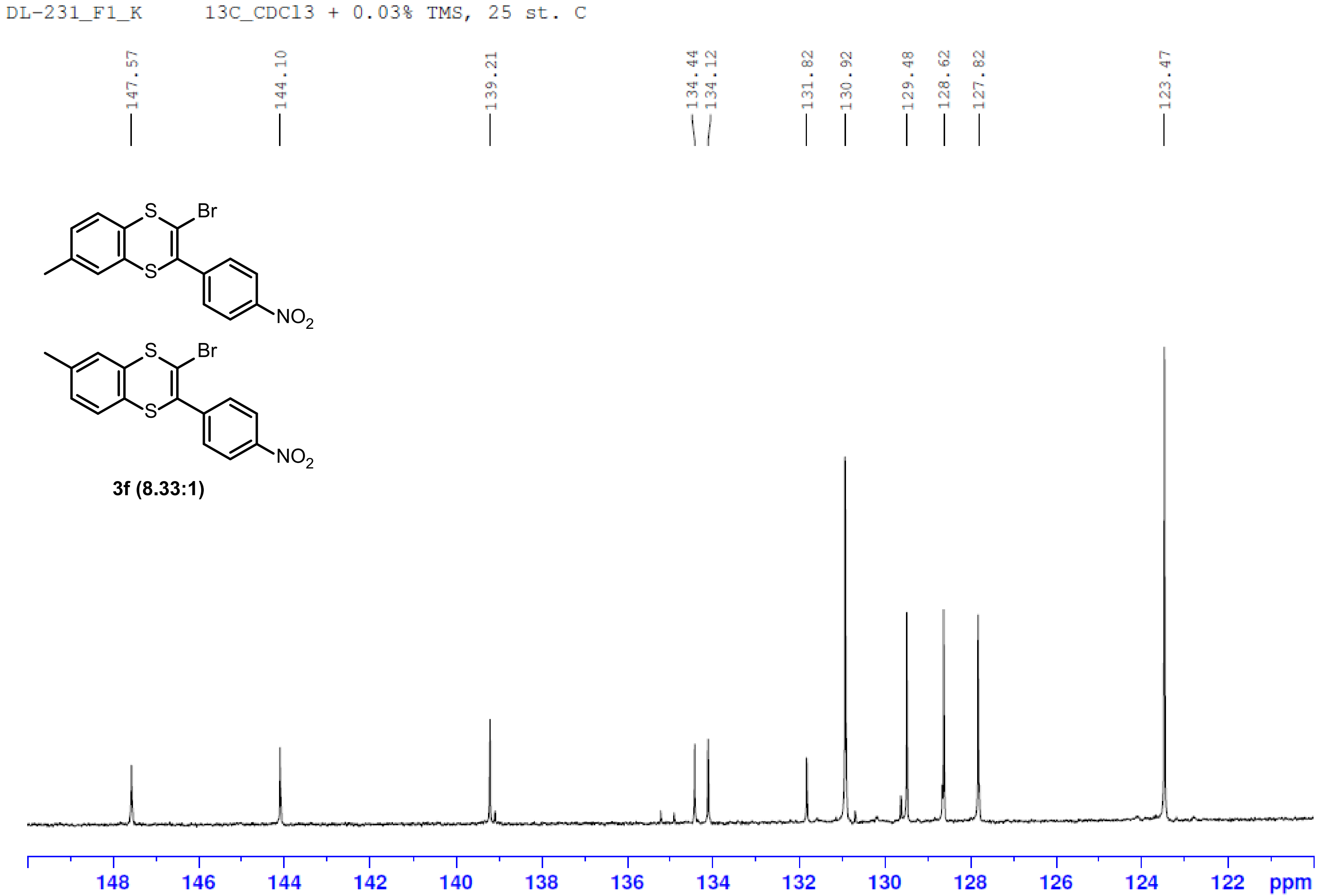
DL-231_F1_K 13C_CDCl3 + 0.03% TMS, 25 st. C
147.57
144.10
139.21
134.44
134.12
131.82
130.92
129.48
128.62
127.82
123.47
S
Br
S
NO2
S
Br
S
NO2
3f (8.33:1)
148
146
144
142
140
138
136
134
132
130
128
126
124
122
ppm

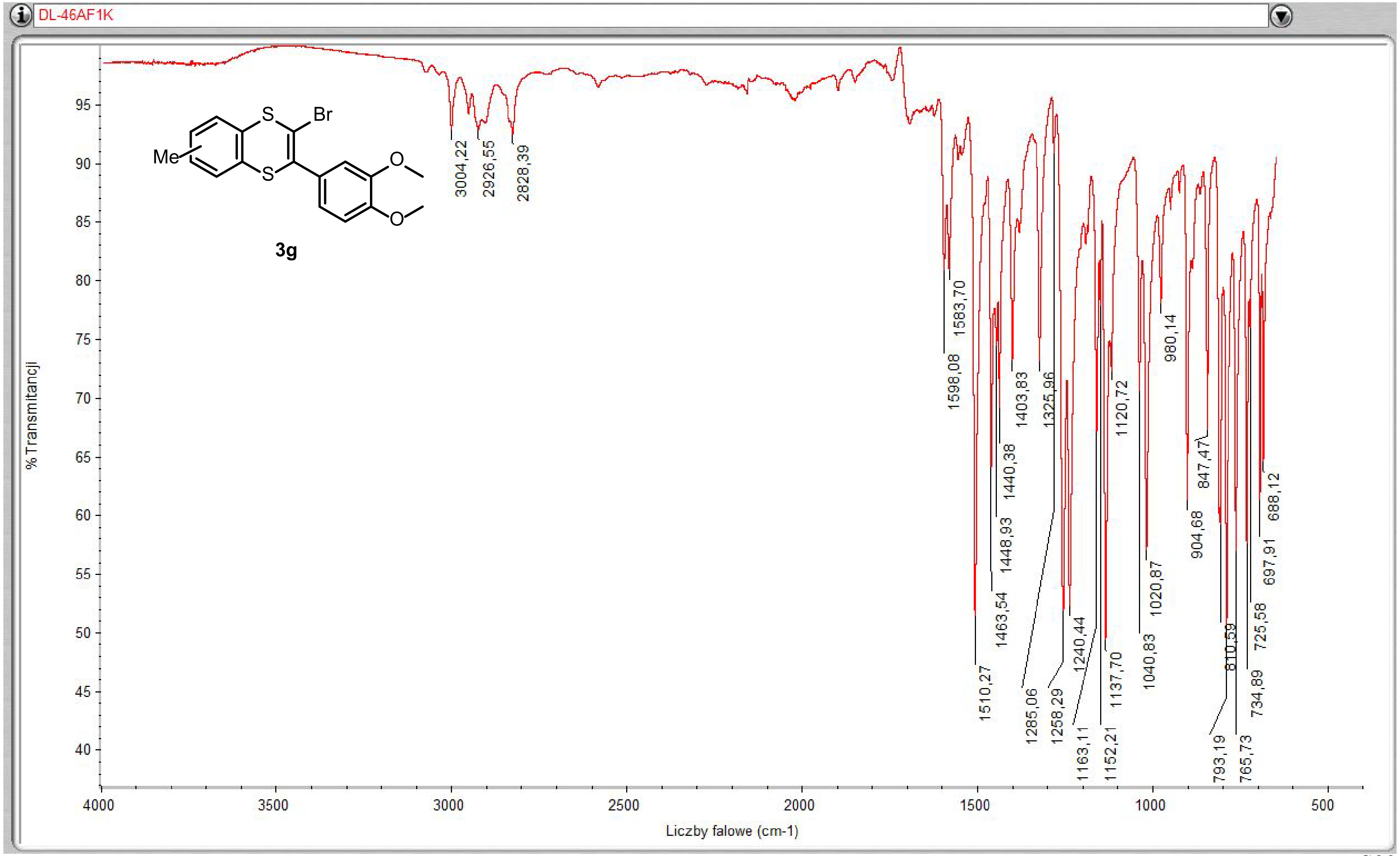
DL-46AF1K
Br
S
S
Me
O
O
3g
%Transmitancji
Liczby falowe (cm-1)
3004,22
2926,55
2828,39
1598,08
1583,70
1510,27
1463,54
1448,93
1440,38
1403,83
1325,96
1285,06
1258,29
1240,44
1163,11
1152,21
1137,70
1120,72
1040,83
1020,87
980,14
904,68
847,47
810,59
793,19
765,73
734,89
725,58
697,91
688,12

DL-46_A_F1 1H_CDCl3 + 0.03% TMS, 25 st. C

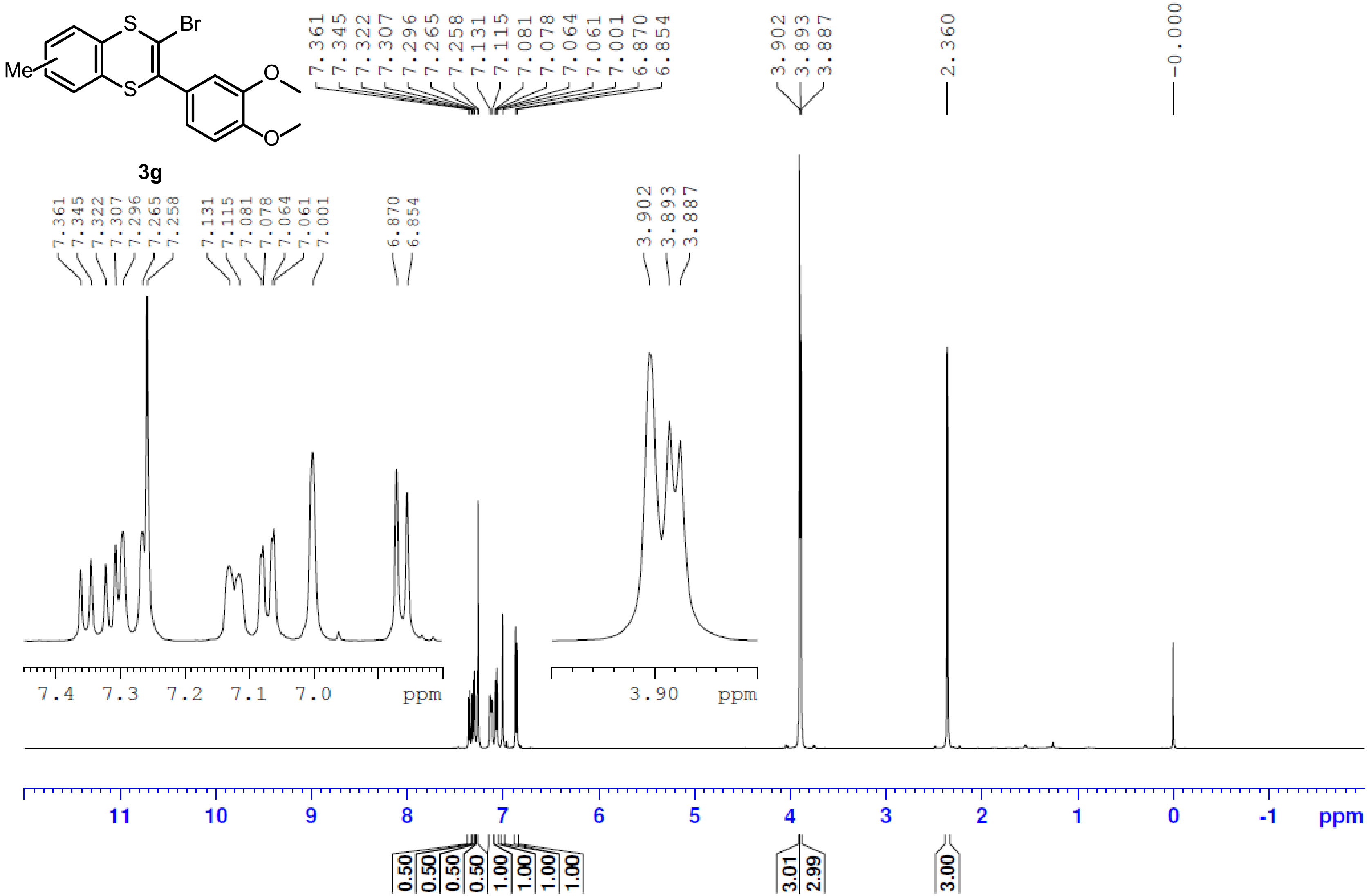

DL-46_A_F1   1H_CDCl3 + 0.03% TMS, 25 st. C

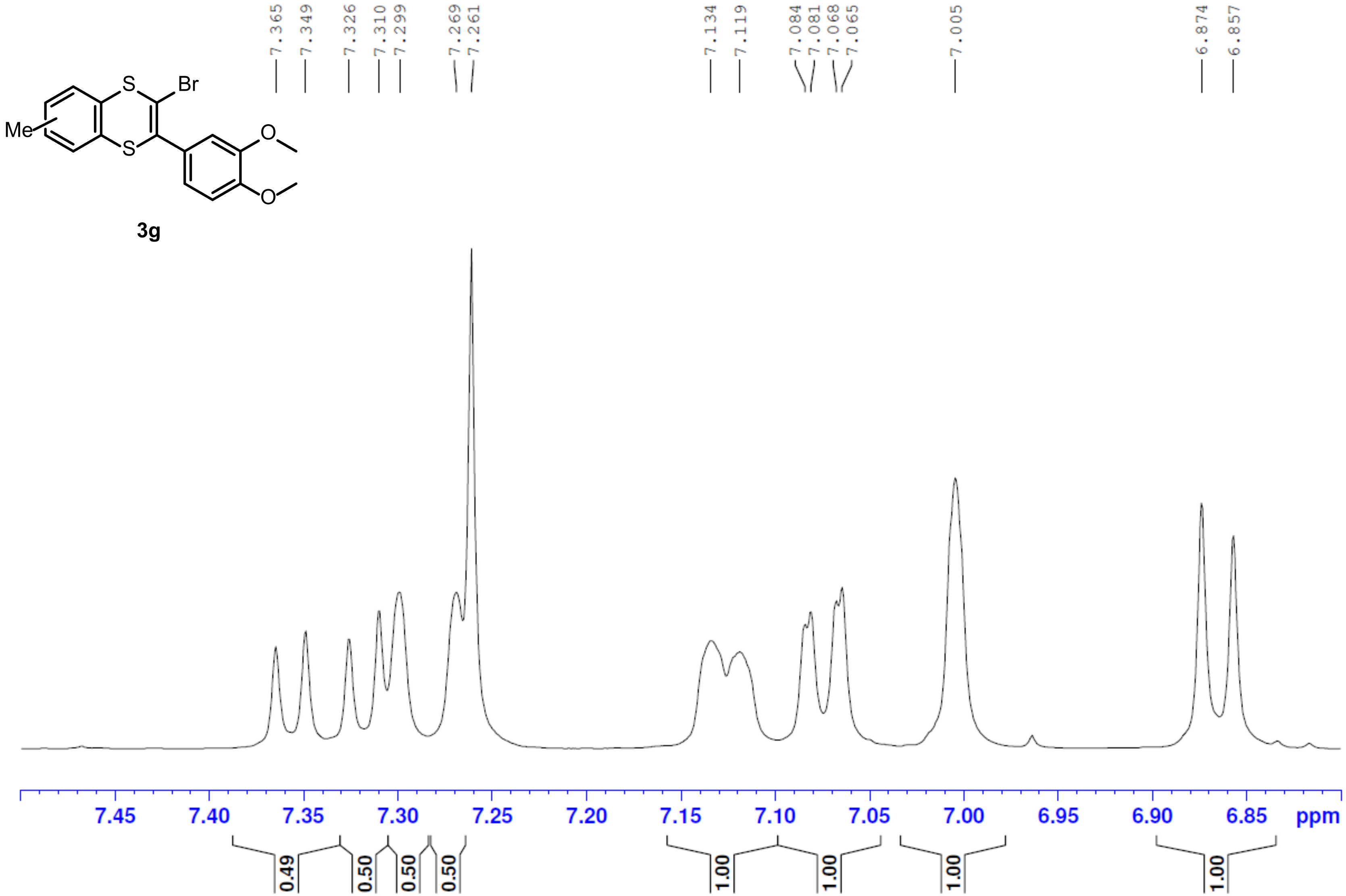

3g

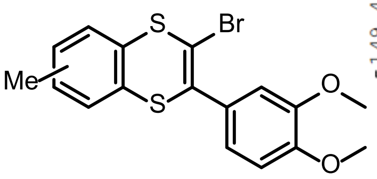
S
Br
Me
S
O
O

3g

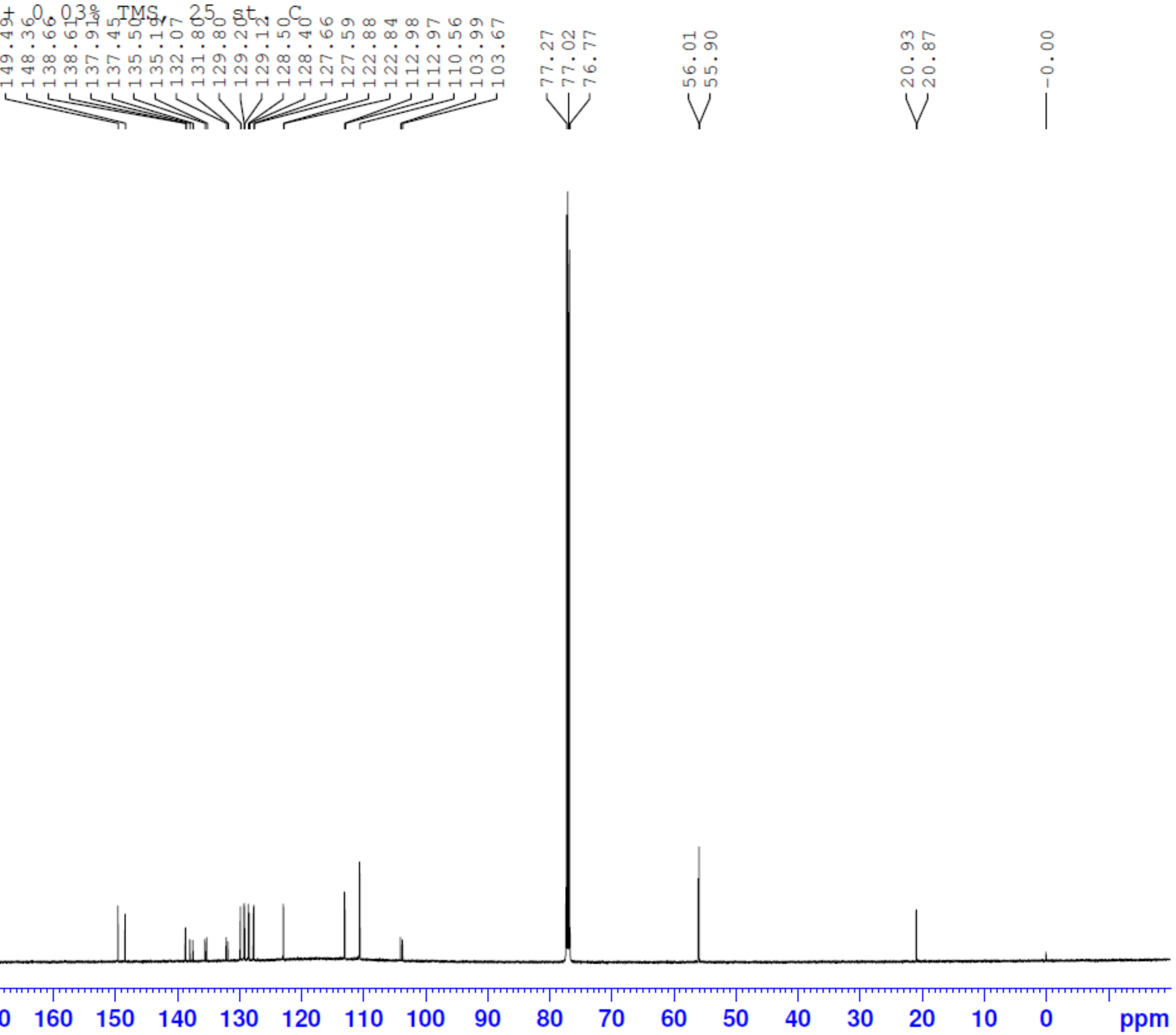
DL-46_A_F1 13C_CDCl3 + 0.03% TMS, 25 st. C
149.49
148.36
138.66
138.61
137.91
137.45
135.50
135.17
132.07
131.80
129.80
129.20
129.12
128.50
128.40
127.66
127.59
122.88
122.84
112.98
112.97
110.56
103.99
103.67
77.27
77.02
76.77
56.01
55.90
20.93
20.87
-0.00
210 200 190 180 170 160 150 140 130 120 110 100 90 80 70 60 50 40 30 20 10 0 ppm

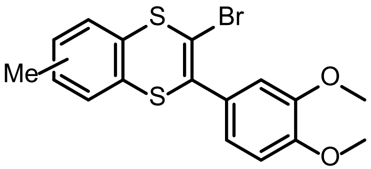

3g

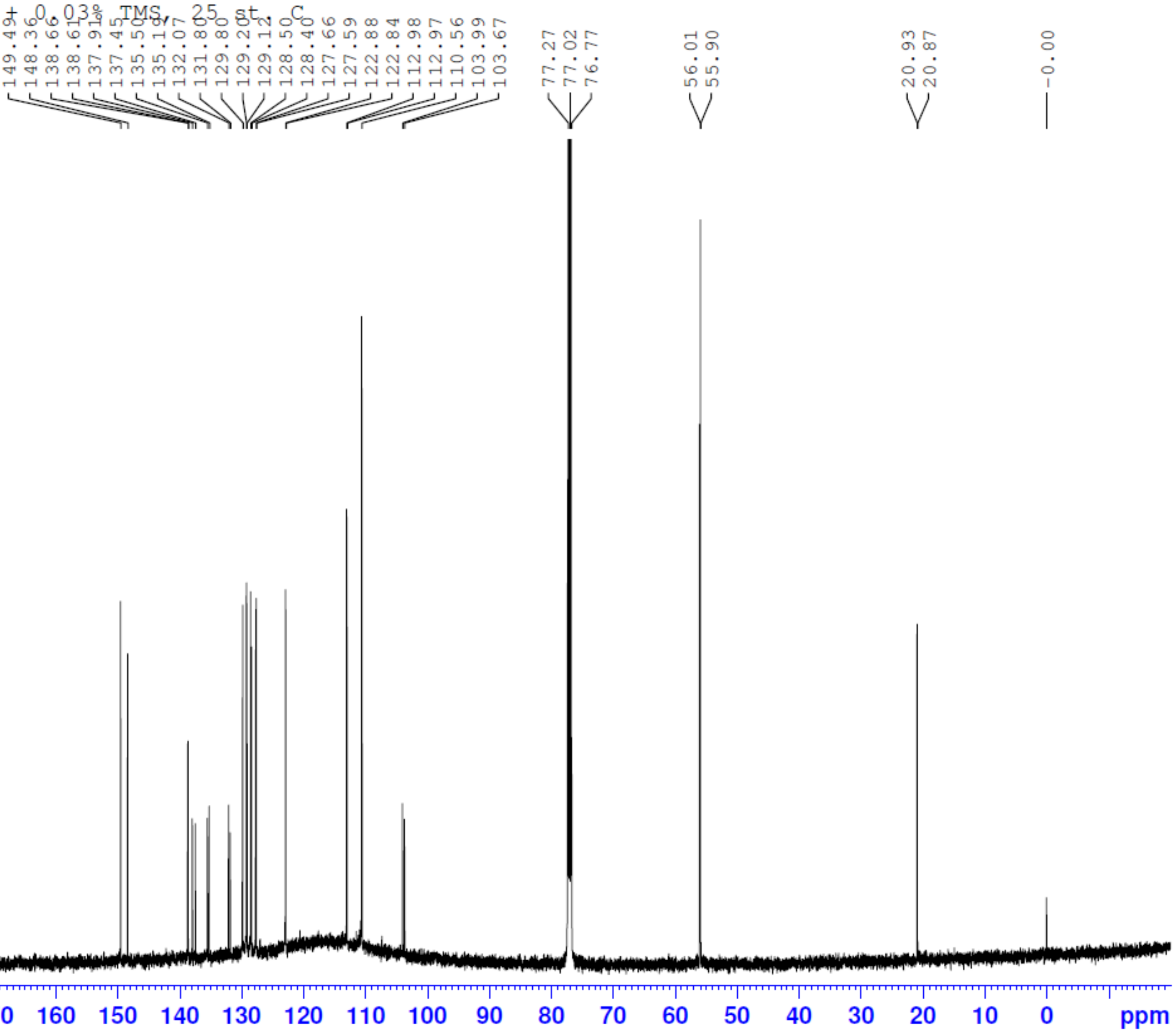
DL-46_A_F1 13C_CDCl3 + 0.03% TMS, 25 st. C
149.49
148.36
138.66
138.61
137.91
137.45
135.50
135.17
132.07
131.80
129.80
129.20
129.12
128.50
128.40
127.66
127.59
122.88
122.84
112.98
112.97
110.56
103.99
103.67
77.27
77.02
76.77
56.01
55.90
20.93
20.87
-0.00
210 200 190 180 170 160 150 140 130 120 110 100 90 80 70 60 50 40 30 20 10 0 ppm

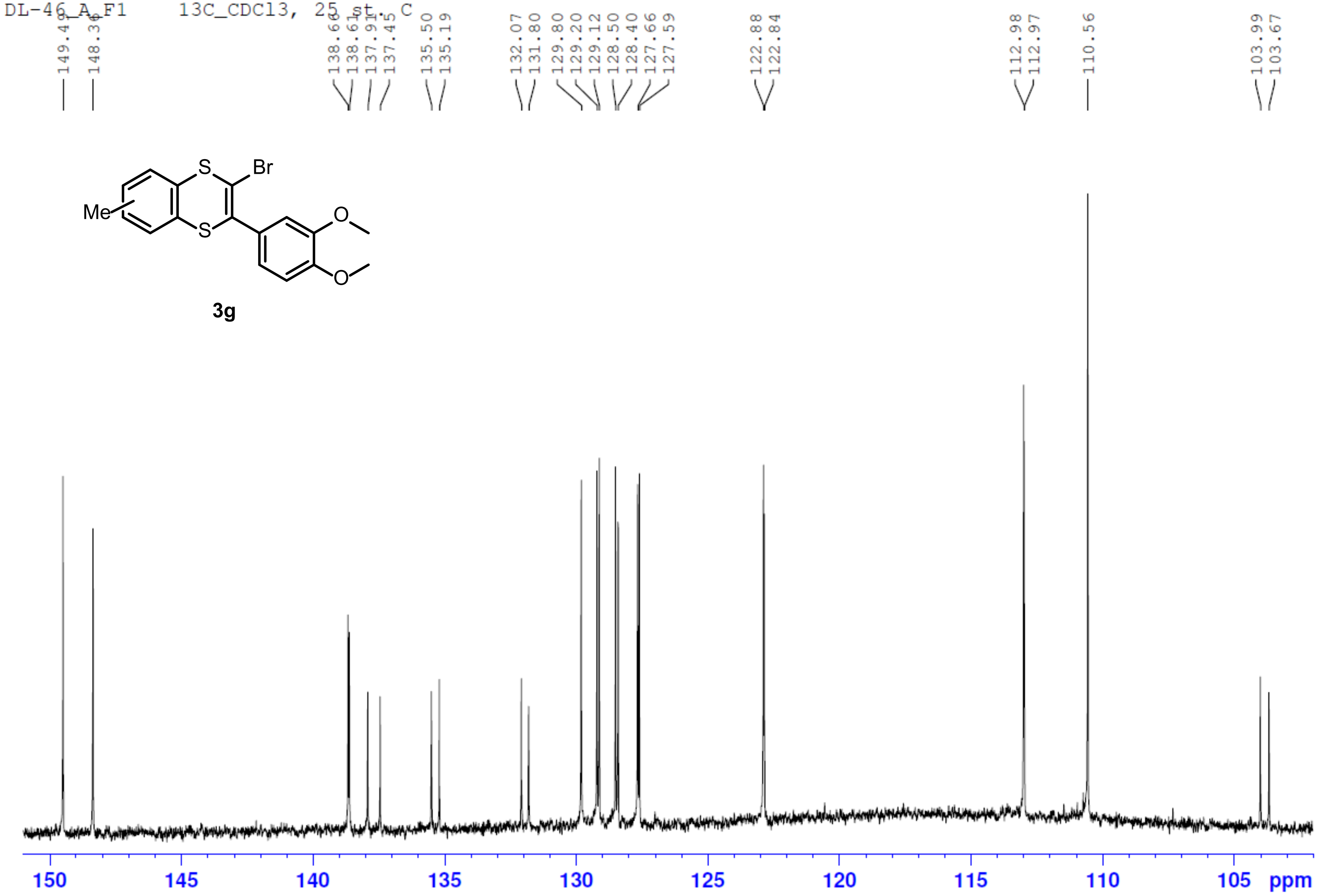
DL-46_A_F1 13C_CDCl3, 25 st. C
149.49
148.36
138.66
138.61
137.91
137.45
135.50
135.19
132.07
131.80
129.80
129.20
129.12
128.50
128.40
127.66
127.59
122.88
122.84
112.98
112.97
110.56
103.99
103.67
S
Br
Me
S
O
O
3g
150
145
140
135
130
125
120
115
110
105
ppm

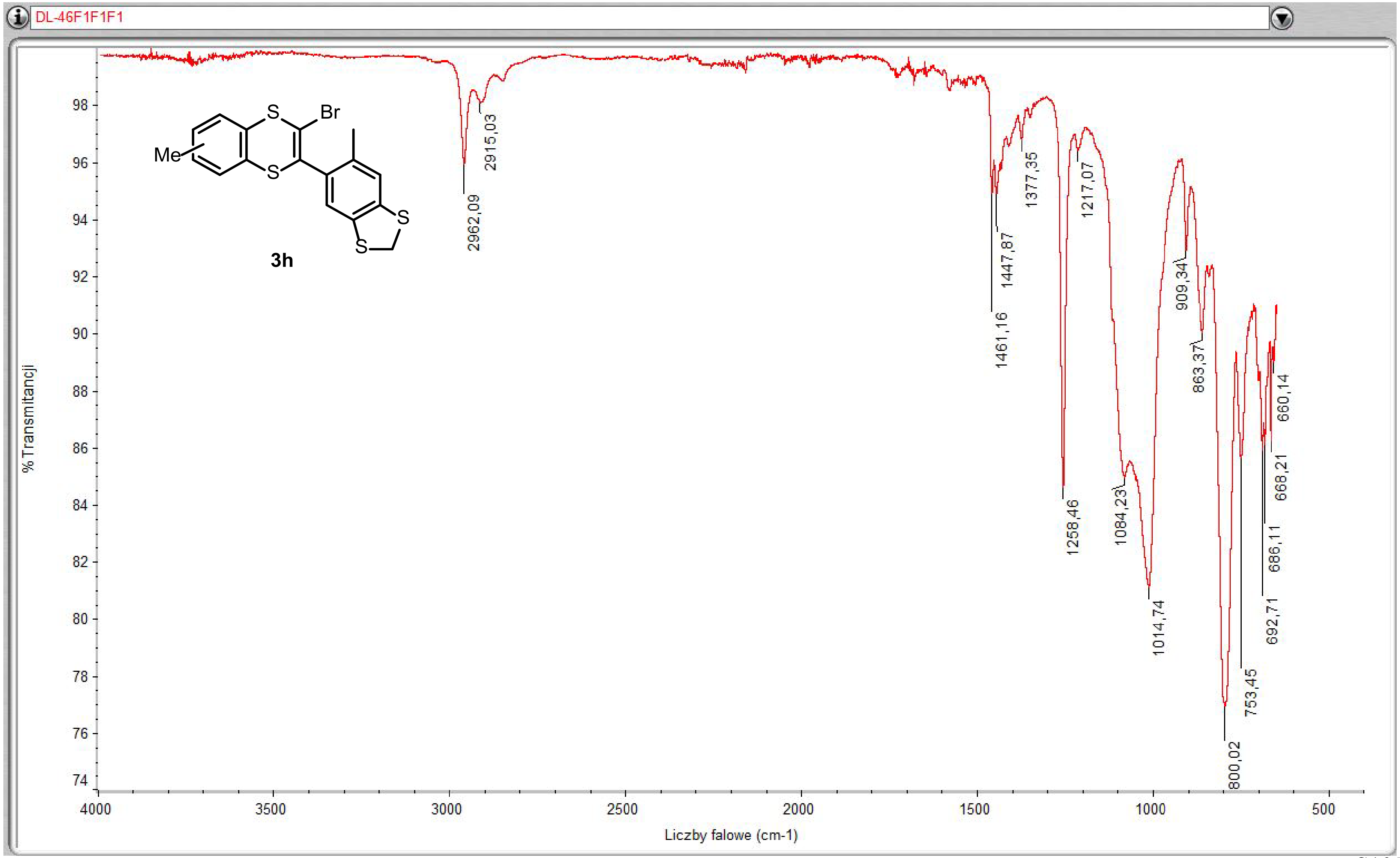

DL-46F1F1F1
3h
% Transmitancji
Liczby falowe (cm-1)
2962,09
2915,03
1461,16
1447,87
1377,35
1258,46
1217,07
1084,23
1014,74
909,34
863,37
800,02
753,45
692,71
686,11
668,21
660,14

DL-46-B_F1F1F1 1H_CDCl3 + 0.03% TMS, 25 st.C

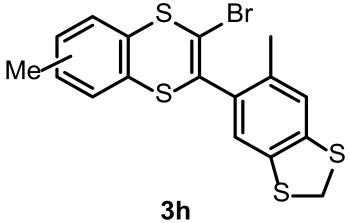

3h

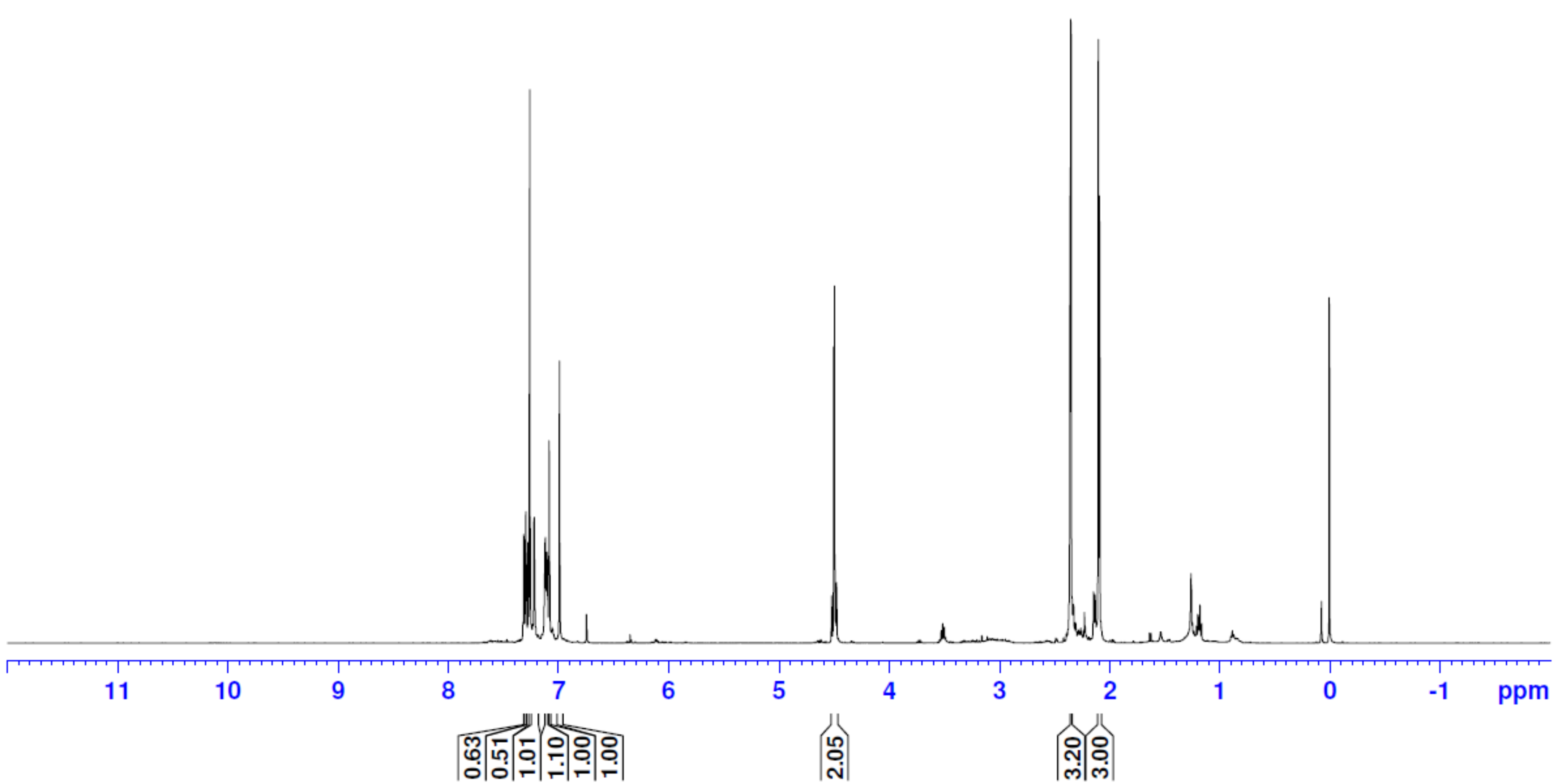

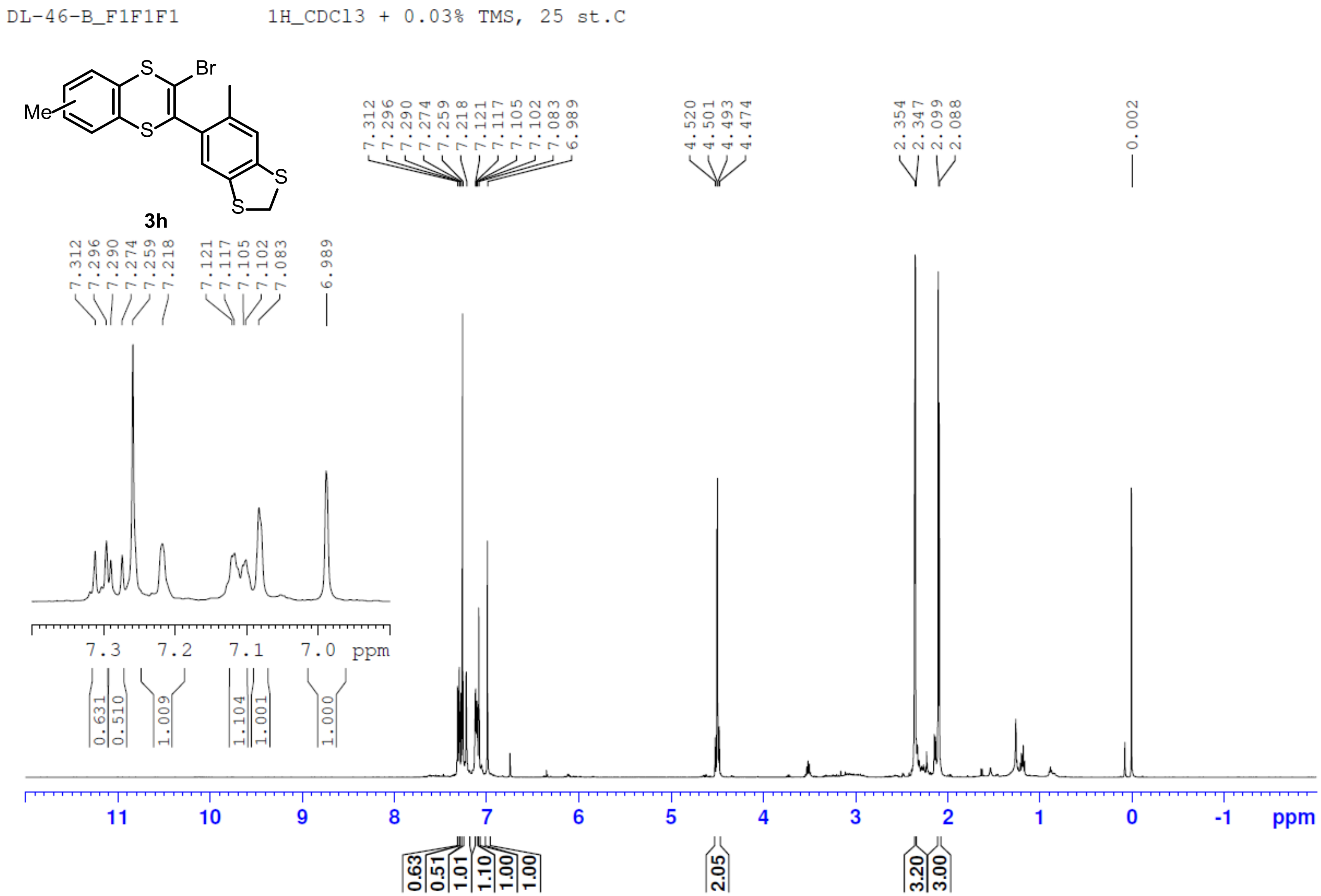
DL-46-B_F1F1F1 1H_CDCl3 + 0.03% TMS, 25 st.C
Br
S
S
Me
3h
7.312
7.296
7.290
7.274
7.259
7.218
7.121
7.117
7.105
7.102
7.083
6.989
4.520
4.501
4.493
4.474
2.354
2.347
2.099
2.088
0.002
7.3
7.2
7.1
7.0
ppm
0.631
0.510
1.009
1.104
1.001
1.000
11
10
9
8
7
6
5
4
3
2
1
0
-1
ppm
0.63
0.51
1.01
1.10
1.00
1.00
2.05
3.20
3.00

DL-46-B_F1F1F1   13C_CDCl3, 25 st C

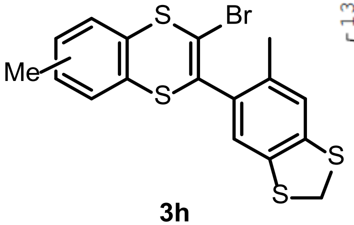

3h

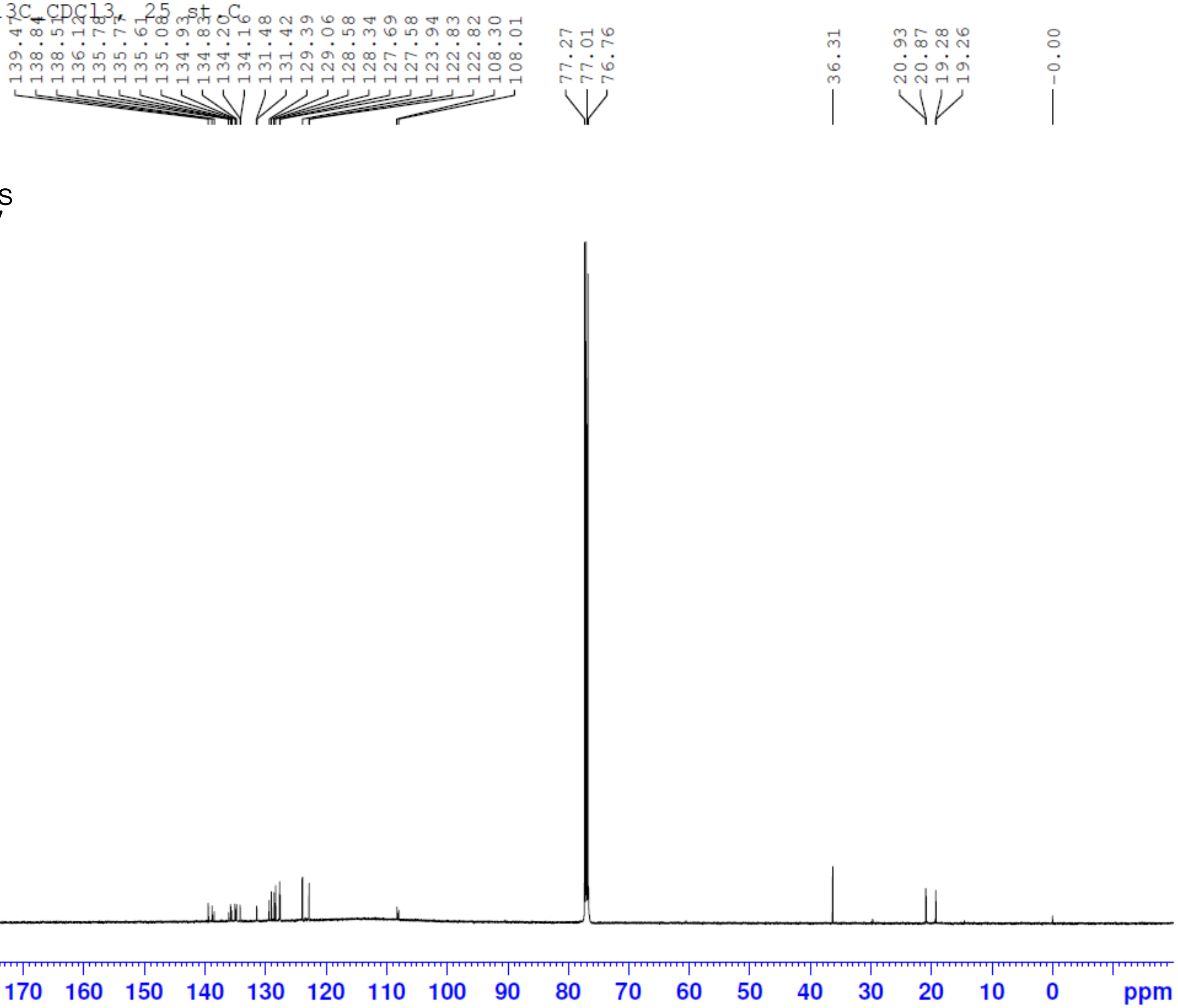

DL-46-B_F1F1F1 13C_CDCl3, 25 st C

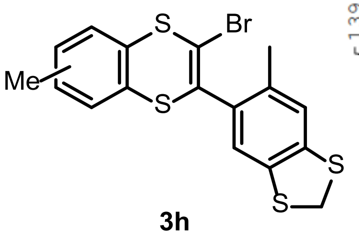

3h

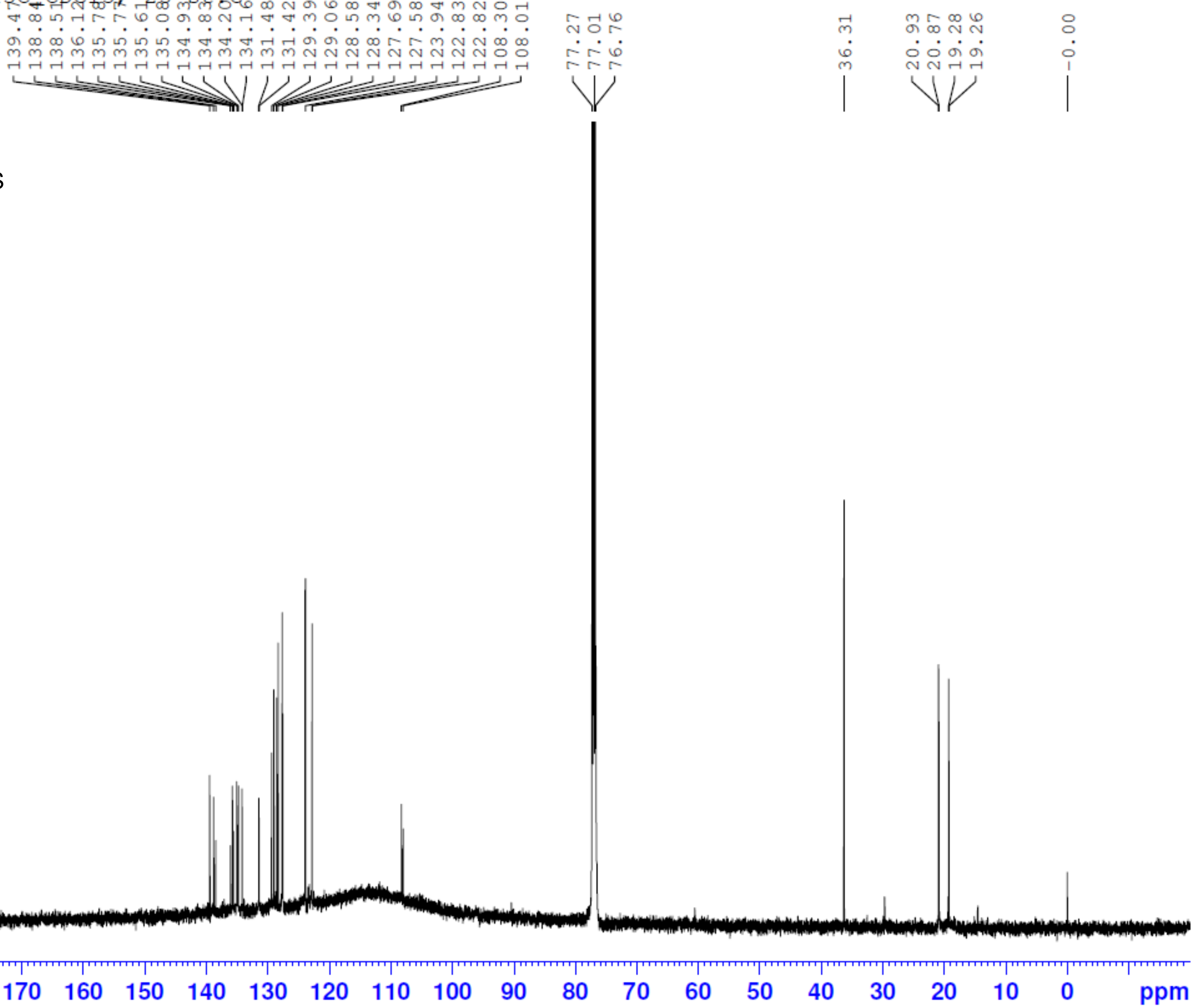

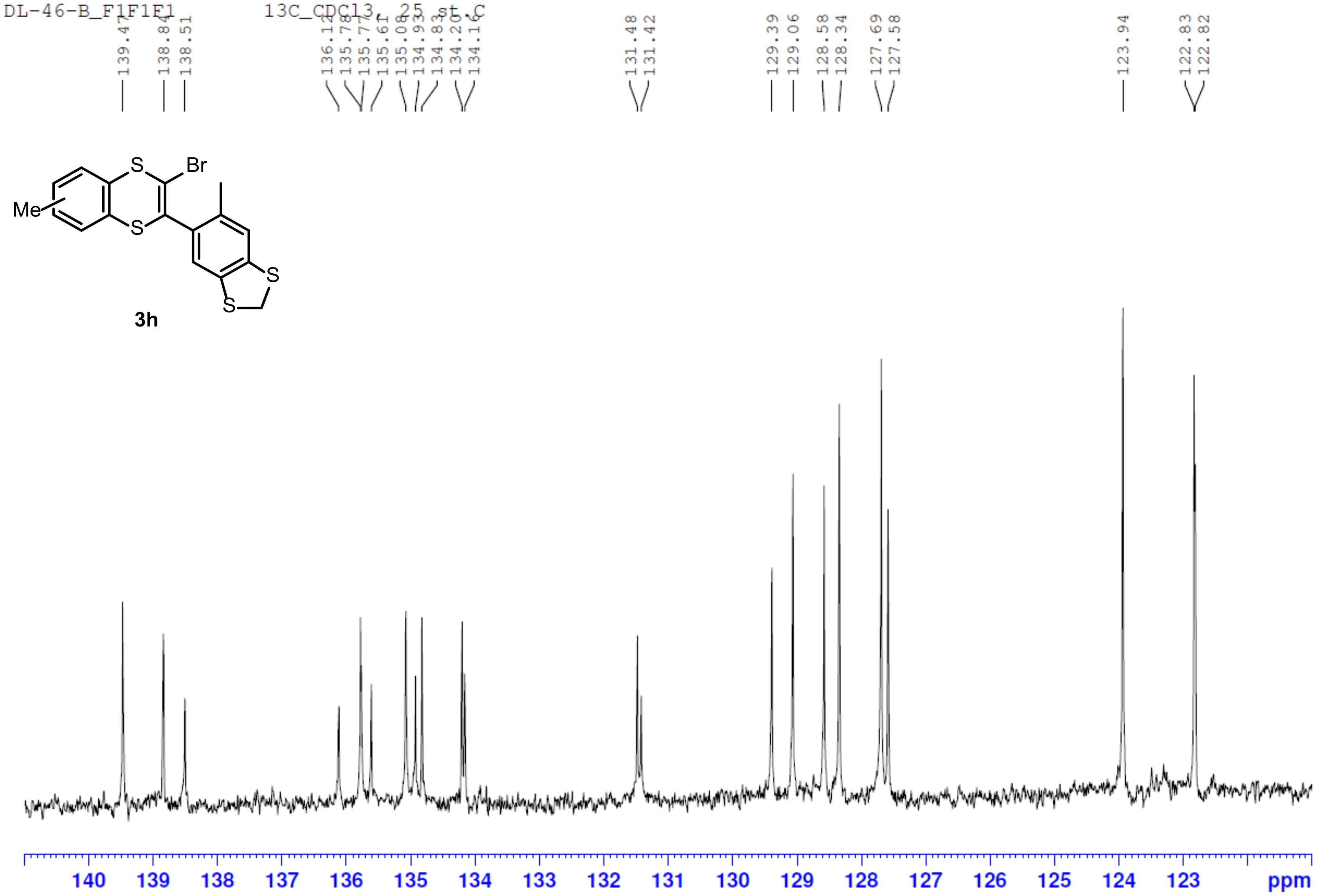
DL-46-B_F1F1F1
13C_CDCl3, 25 st.C
139.47
138.84
138.51
136.12
135.78
135.77
135.61
135.08
134.93
134.83
134.20
134.16
131.48
131.42
129.39
129.06
128.58
128.34
127.69
127.58
123.94
122.83
122.82
S
Br
Me
S
S
S
3h
140 139 138 137 136 135 134 133 132 131 130 129 128 127 126 125 124 123 ppm

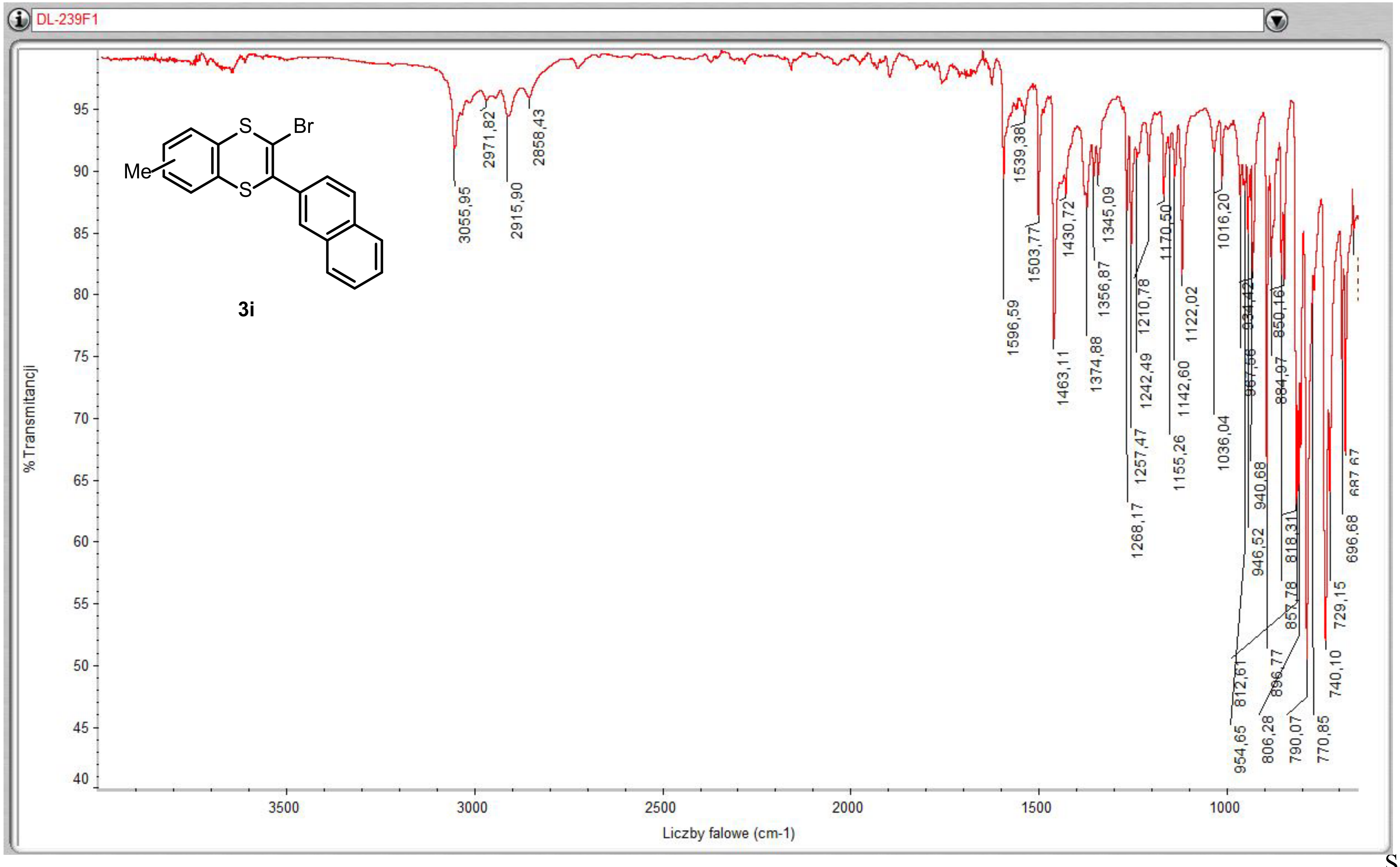
DL-239F1
3i
%Transmitancji
Liczby falowe (cm-1)
3055,95
2971,82
2915,90
2858,43
1596,59
1539,38
1503,77
1463,11
1430,72
1374,88
1356,87
1345,09
1268,17
1257,47
1242,49
1210,78
1170,50
1155,26
1142,60
1122,02
1036,04
1016,20
954,65
946,52
940,68
812,61
806,28
896,77
884,97
857,78
850,16
818,31
790,07
770,85
740,10
729,15
696,68

DL-239_F1_K   1H_CDCl3, 25 st. C

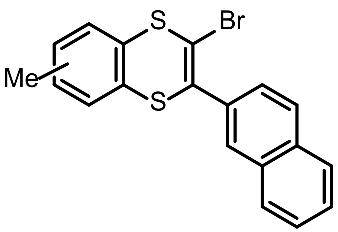

3i

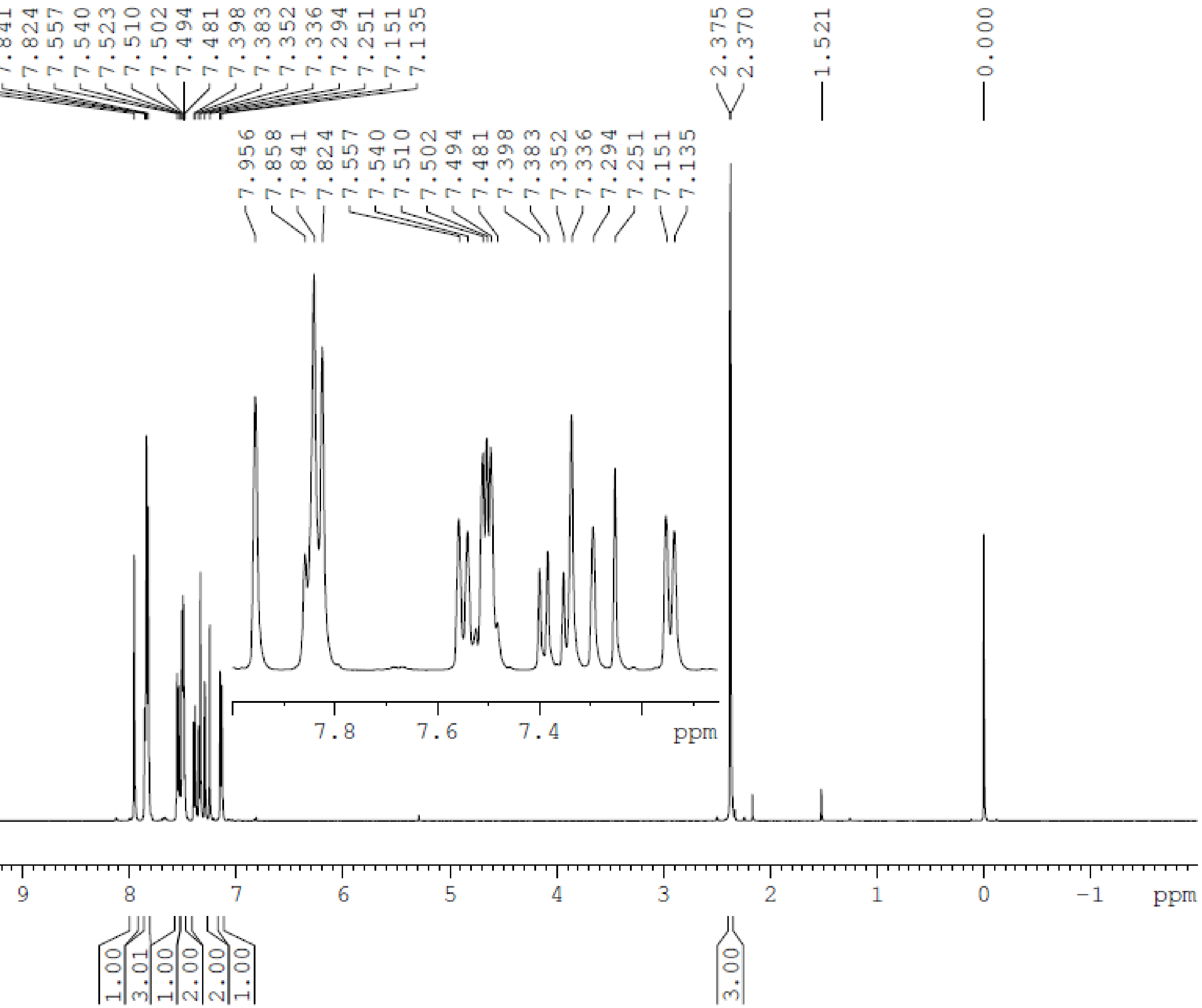

DL-239_F1_K 1H_CDCl3 + 0.03% TMS, 25 st. C

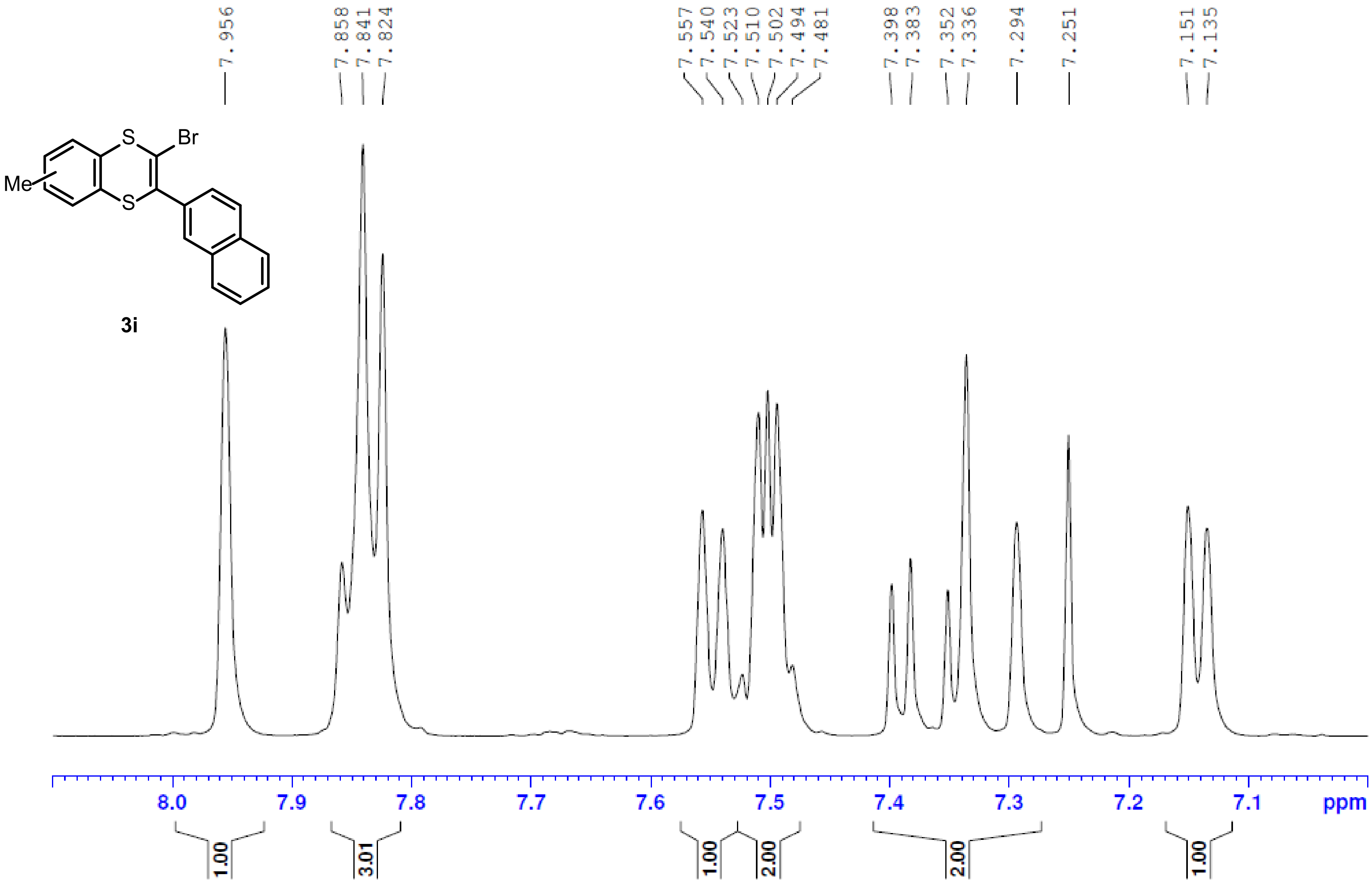

3i

DL-239_F1_K 13C_CDCl3 + 0.03% TMS, 25 st. C

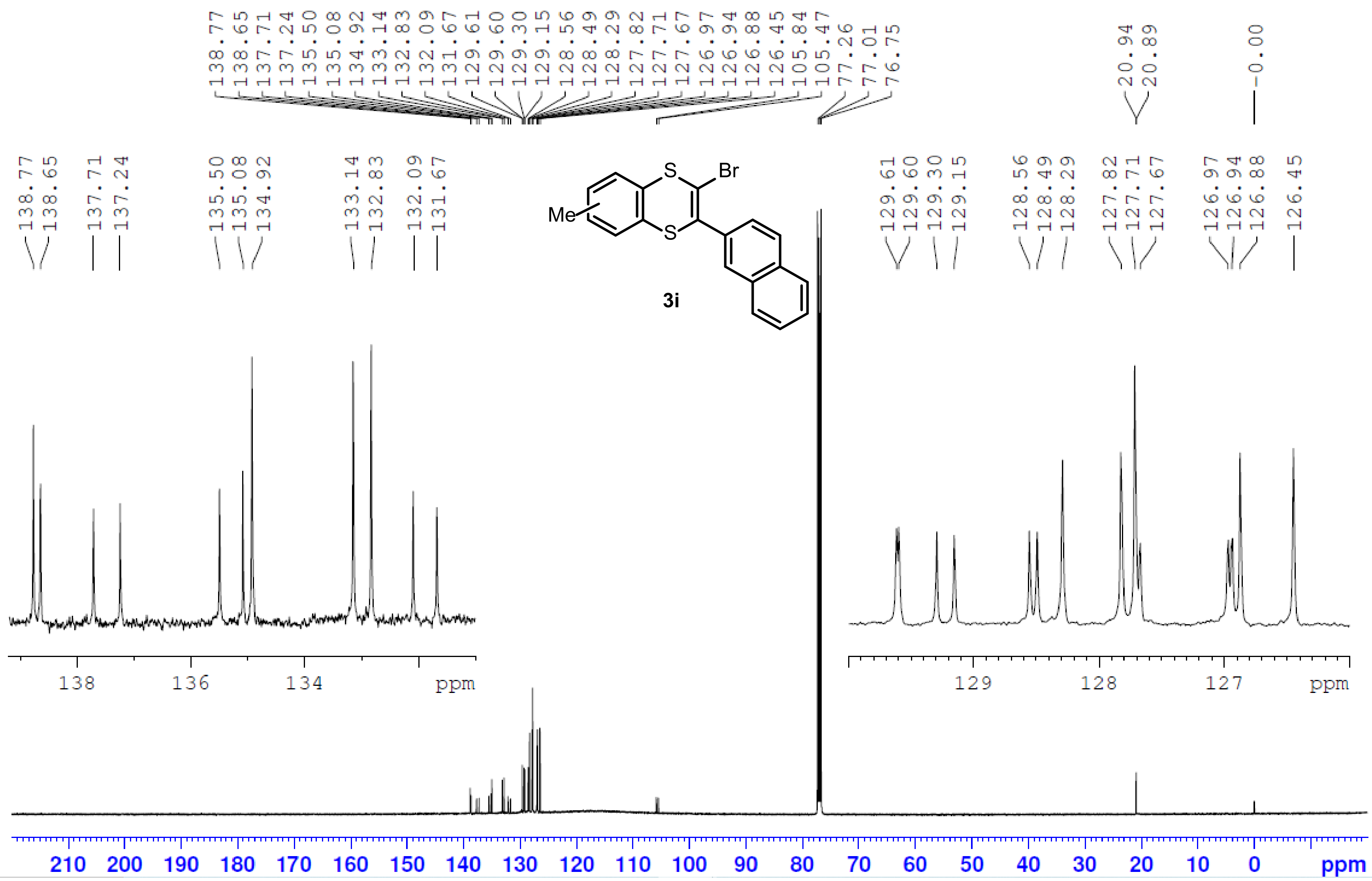

DL-239_F1_K gHSQC_CDCl3 + 0.03% TMS, 25 st. C

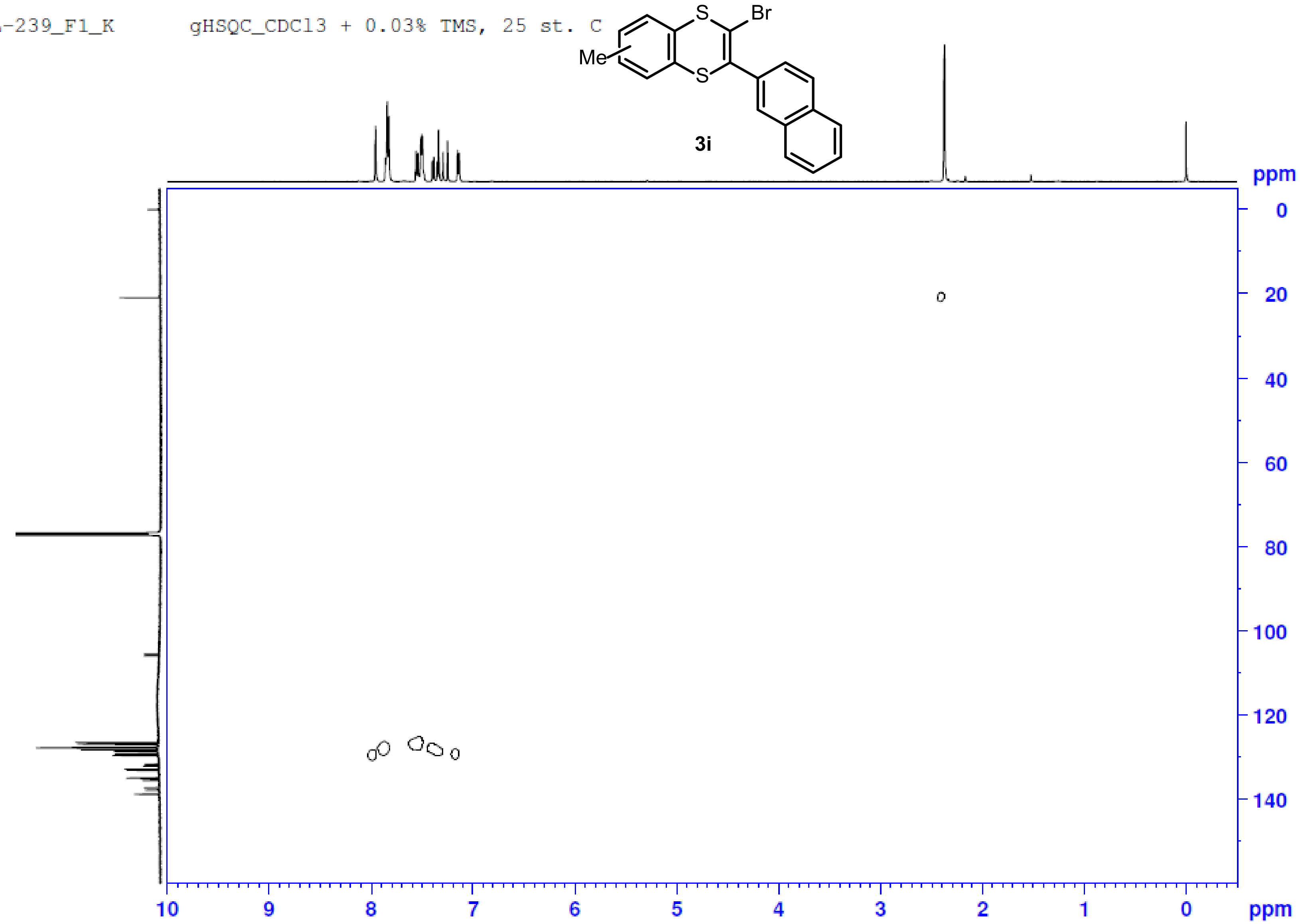

DL-239_F1_K gHMBC_CDCl3 + 0.03% TMS, 25 st. C

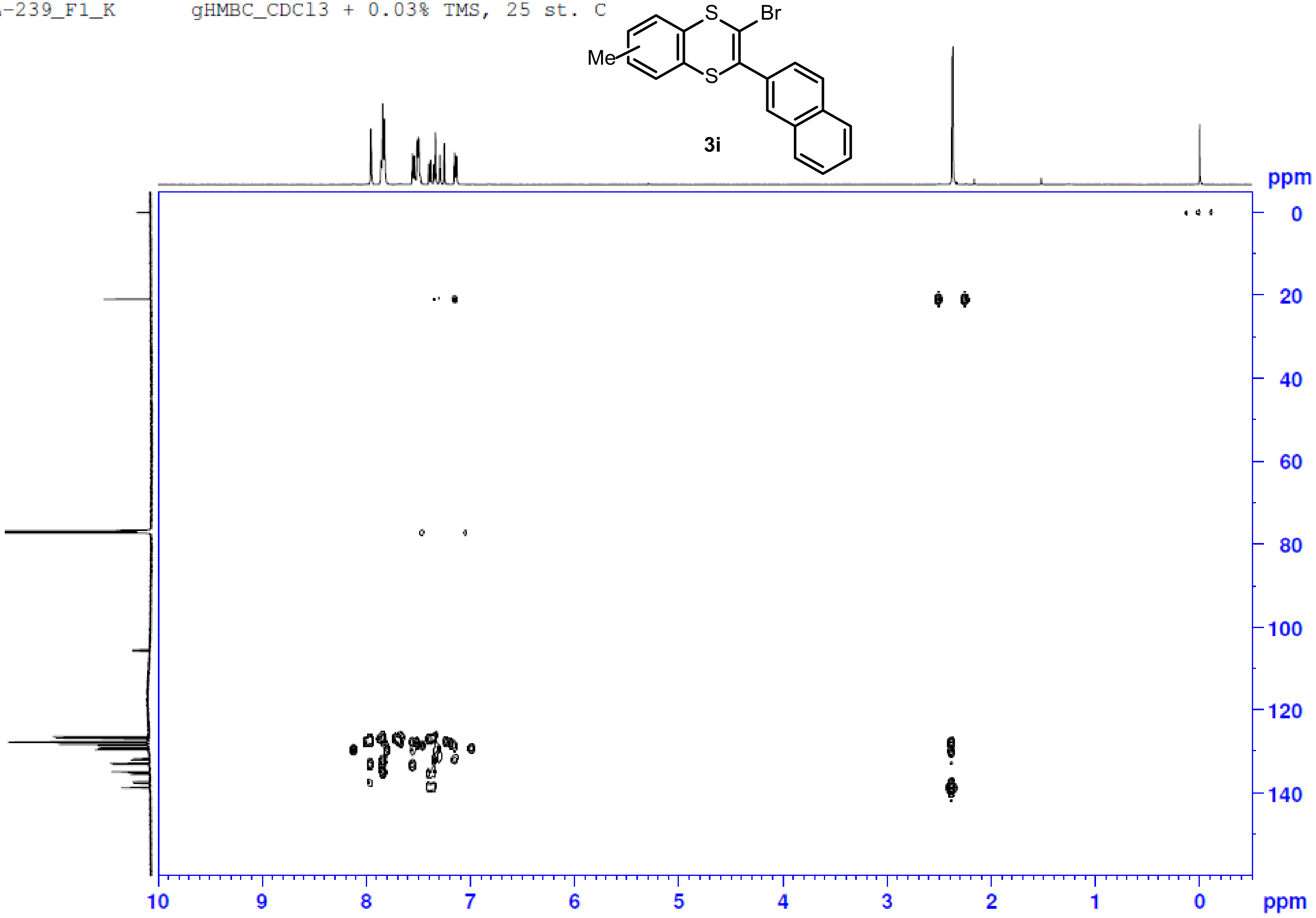

DL-238_F1 1H_CDCl3 + 0.03% TMS 25 st. C

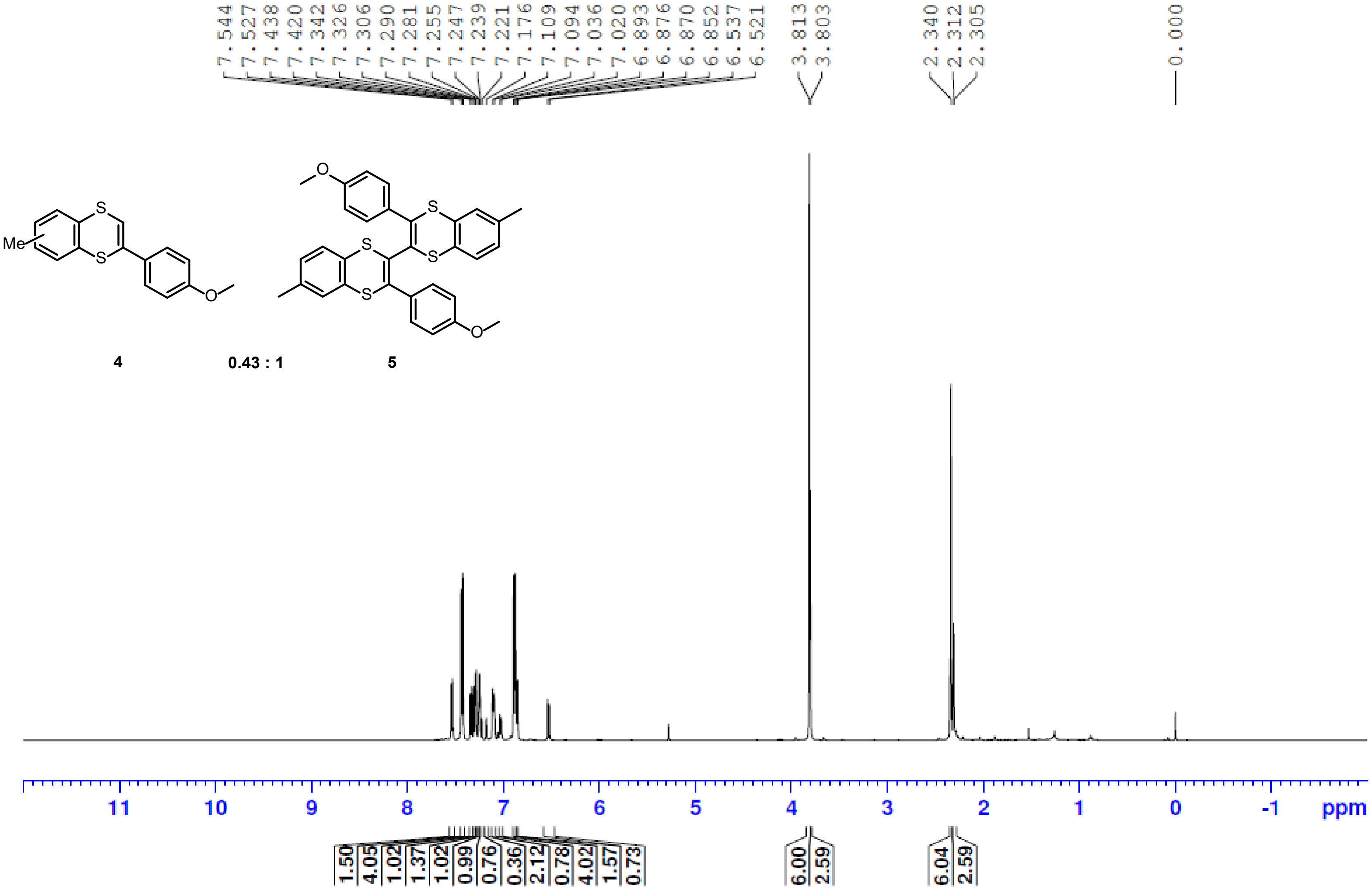

DL-238_F1 1H_CDCl3 + 0.03% TMS 25 st. C

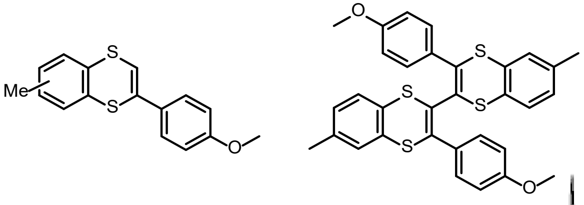

4 0.43 : 1 5

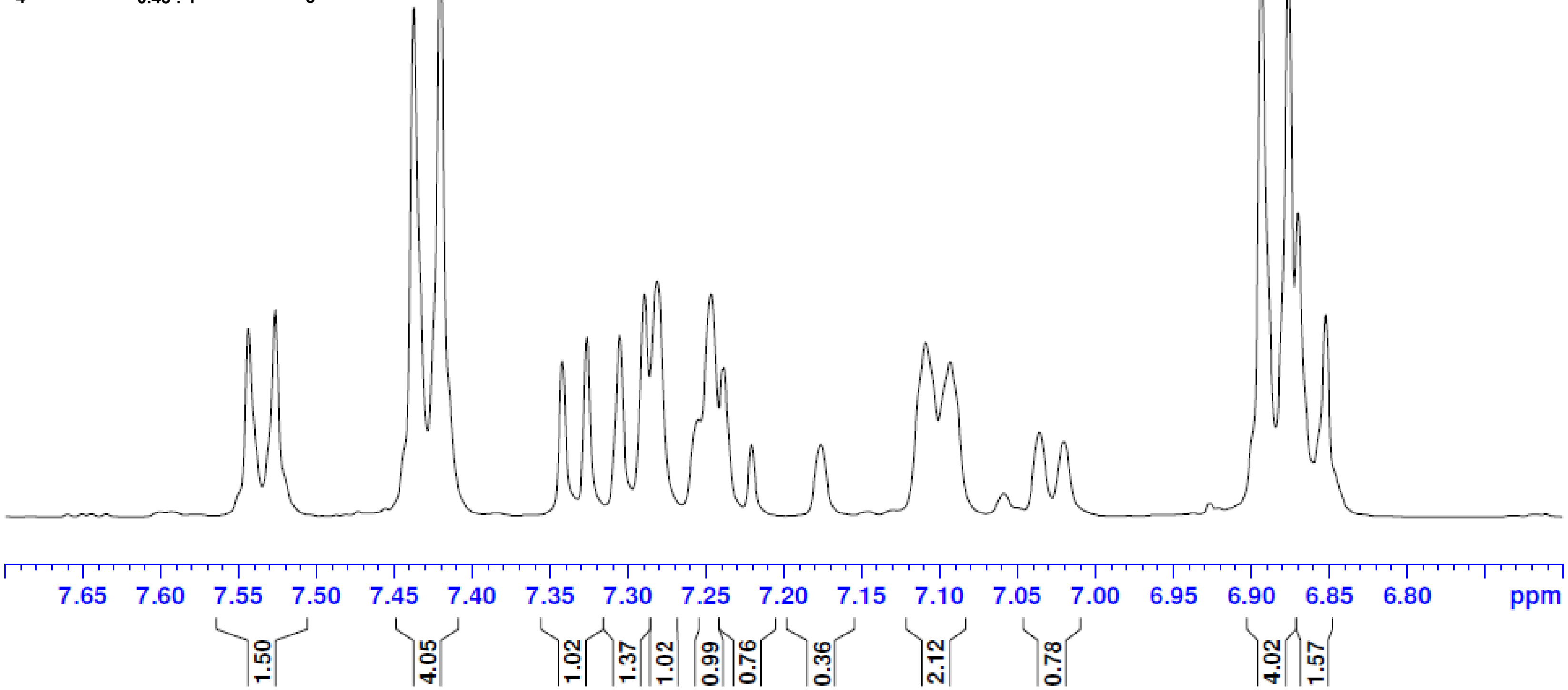

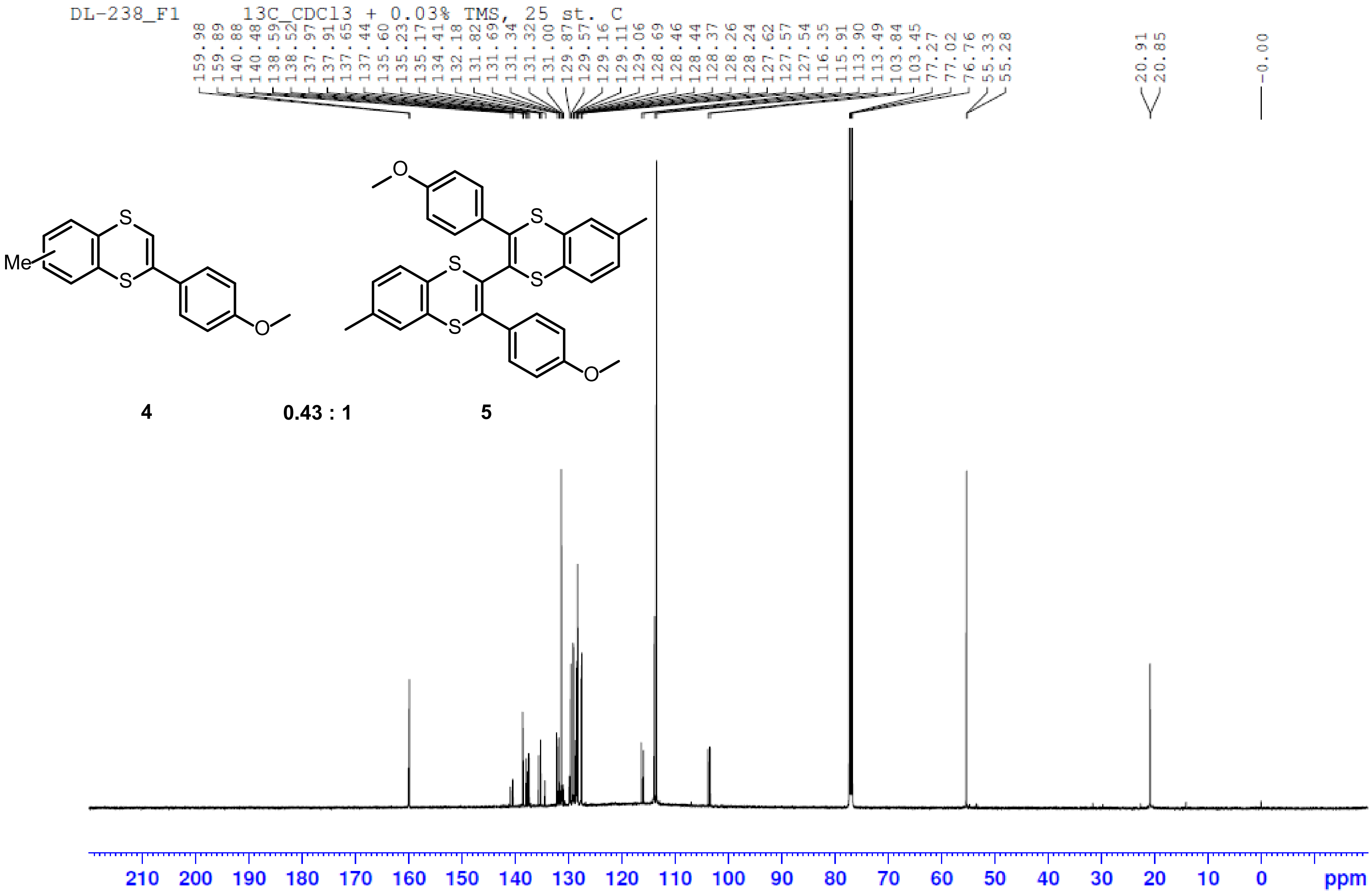
DL-238_F1 13C_CDCl3 + 0.03% TMS, 25 st. C
159.98 159.89 140.88 140.48 138.59 138.52 137.97 137.91 137.65 137.44 135.60 135.23 135.17 134.41 132.18 131.82 131.69 131.34 131.32 131.00 129.87 129.57 129.16 129.11 129.06 128.69 128.46 128.44 128.37 128.26 128.24 127.62 127.57 127.54 116.35 115.91 113.90 113.49 103.84 103.45 77.27 77.02 76.76 55.33 55.28
20.91 20.85
-0.00
Me
S
O
4
0.43 : 1
5
210 200 190 180 170 160 150 140 130 120 110 100 90 80 70 60 50 40 30 20 10 0 ppm

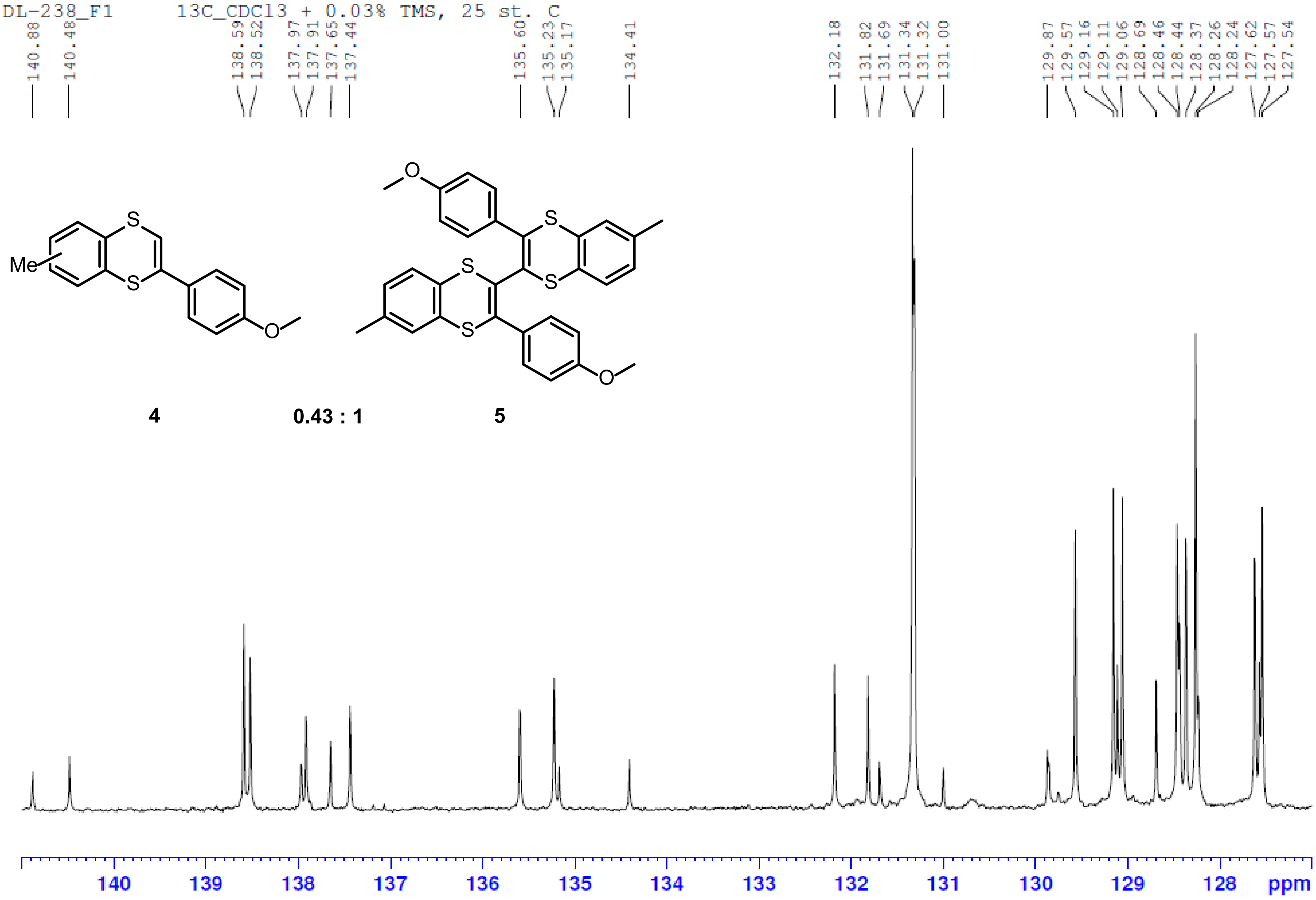
DL-238_F1 13C_CDCl3 + 0.03% TMS, 25 st. C
140.88
140.48
138.59
138.52
137.97
137.91
137.65
137.44
135.60
135.23
135.17
134.41
132.18
131.82
131.69
131.34
131.32
131.00
129.87
129.57
129.16
129.11
129.06
128.69
128.46
128.44
128.37
128.26
128.24
127.62
127.57
127.54
Me
S
O
4
0.43 : 1
5
140
139
138
137
136
135
134
133
132
131
130
129
128
ppm

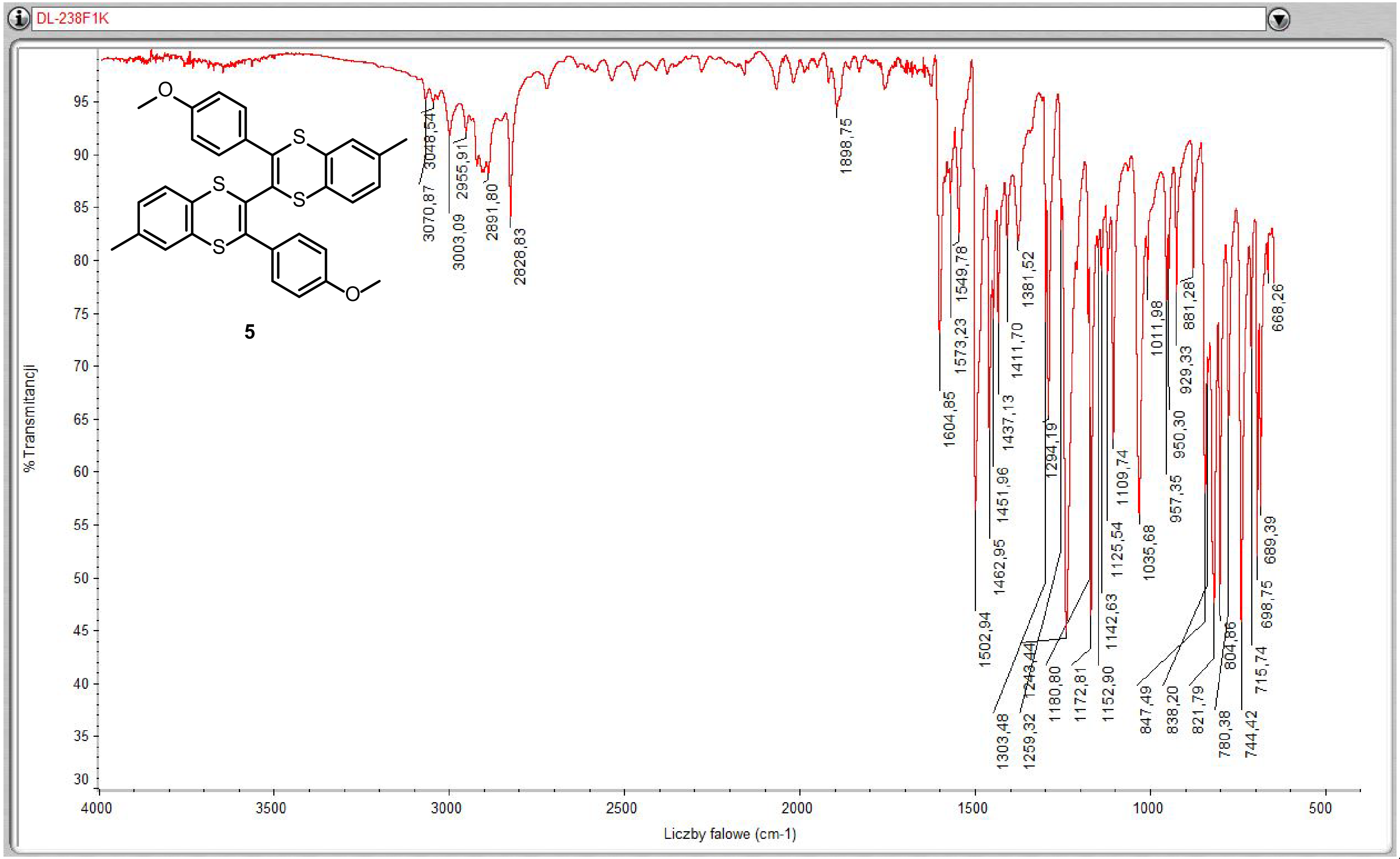
DL-238F1K
5
% Transmitancji
Liczby falowe (cm-1)
3070,87
3048,54
3003,09
2955,91
2891,80
2828,83
1898,75
1604,85
1573,23
1549,78
1502,94
1462,95
1451,96
1437,13
1411,70
1381,52
1303,48
1294,19
1259,32
1243,44
1180,80
1172,81
1152,90
1142,63
1125,54
1109,74
1035,68
1011,98
957,35
950,30
929,33
881,28
847,49
838,20
821,79
804,86
780,38
744,42
715,74
698,75
689,39
668,26

DL-157_B_F1_K2 1H_CDCl3 + 0.03% TMS, 25 st.C

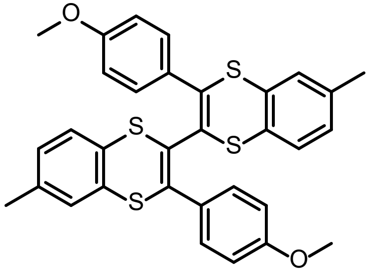

5

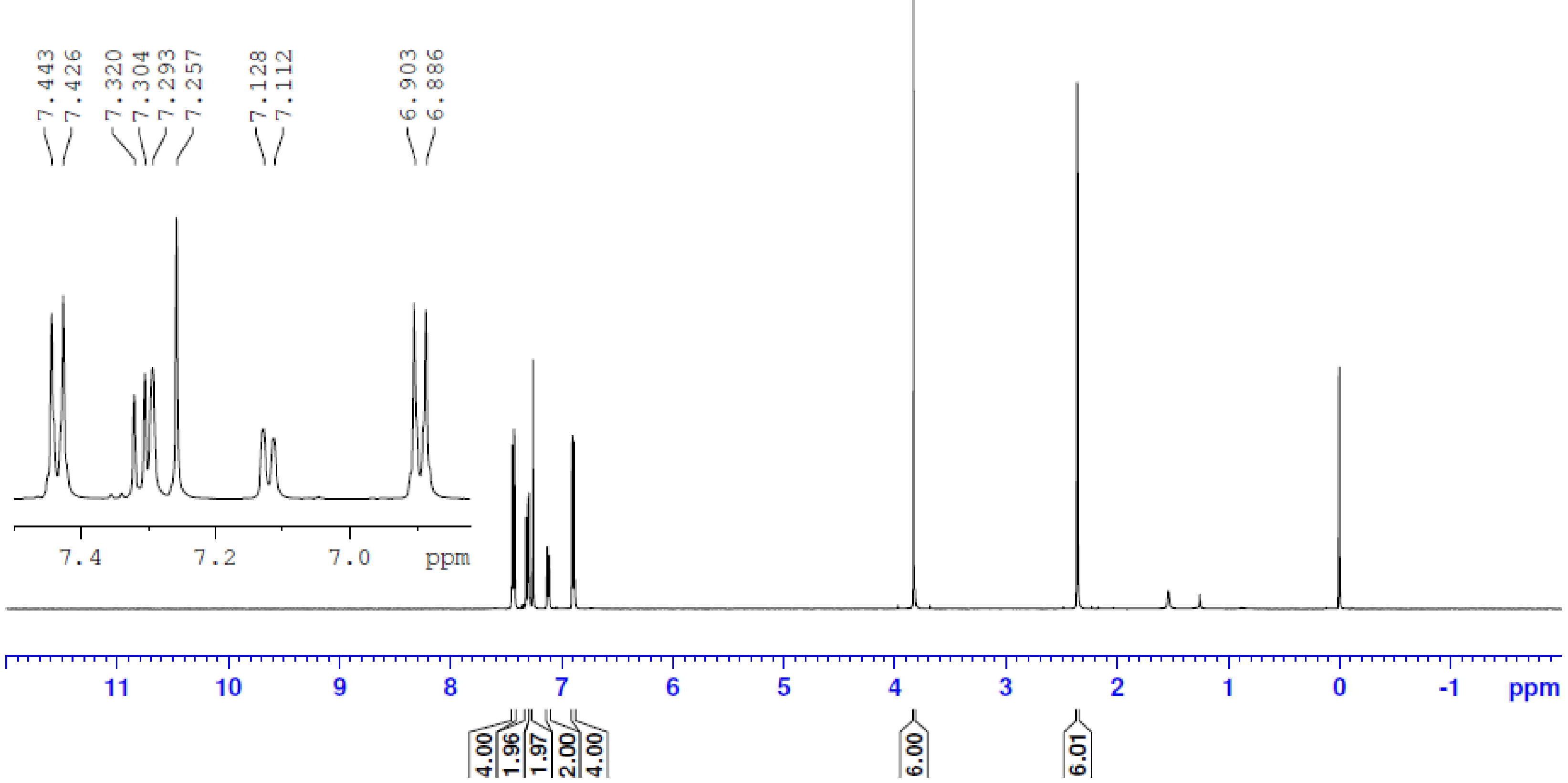

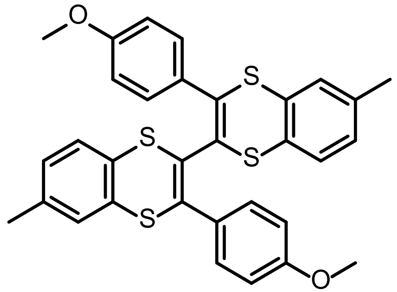

5

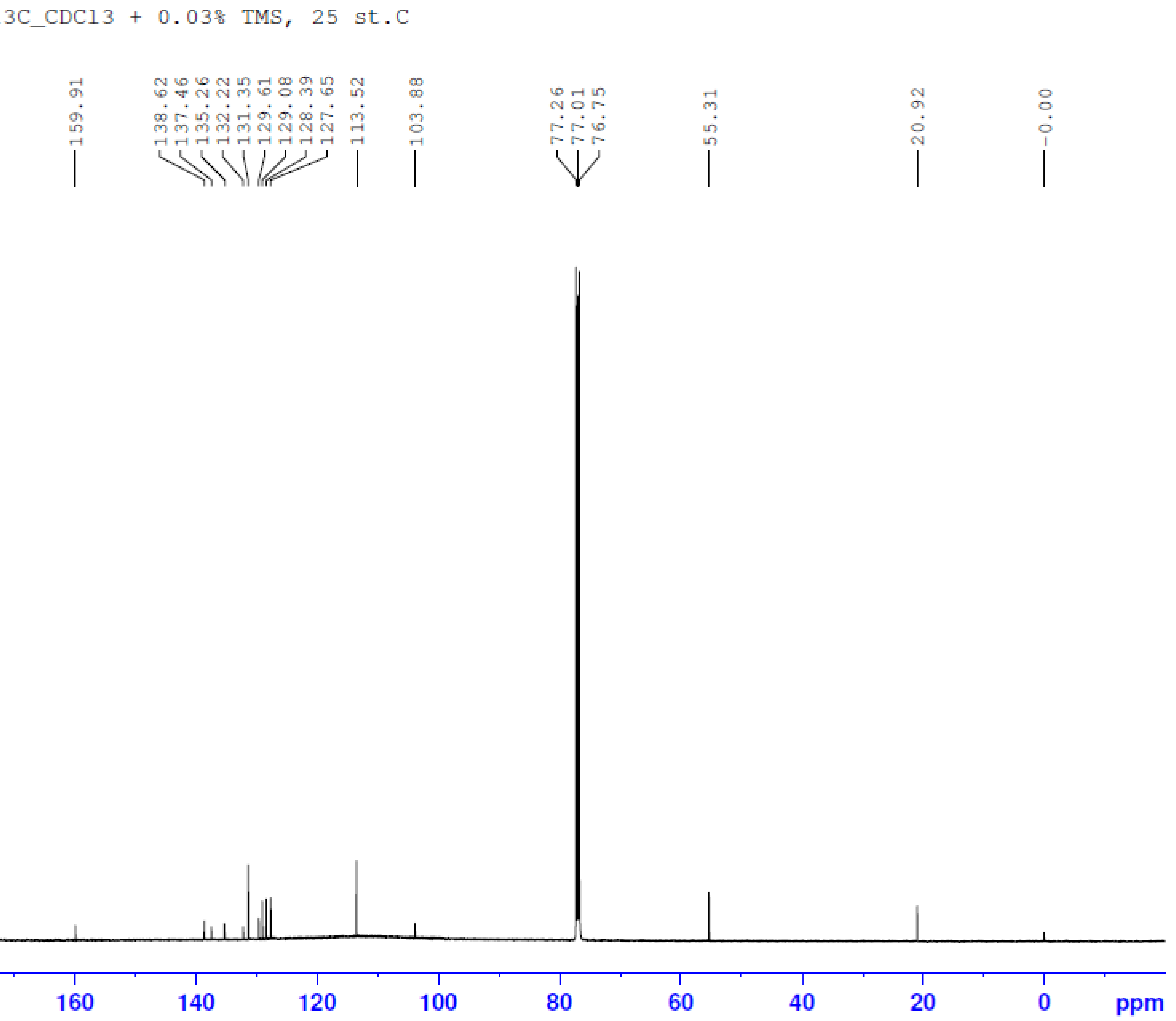

DL-157_B_F1_K2
13C_CDCl3 + 0.03% TMS, 25 st.C
159.91
138.62
137.46
135.26
132.22
131.35
129.61
129.08
128.39
127.65
113.52
103.88
77.26
77.01
76.75
55.31
20.92
-0.00
200
180
160
140
120
100
80
60
40
20
0
ppm

DL-157_B_F1_K2 13C_CDCl3 + 0.03% TMS, 25 st.C

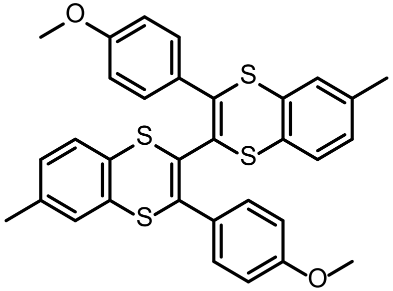

5

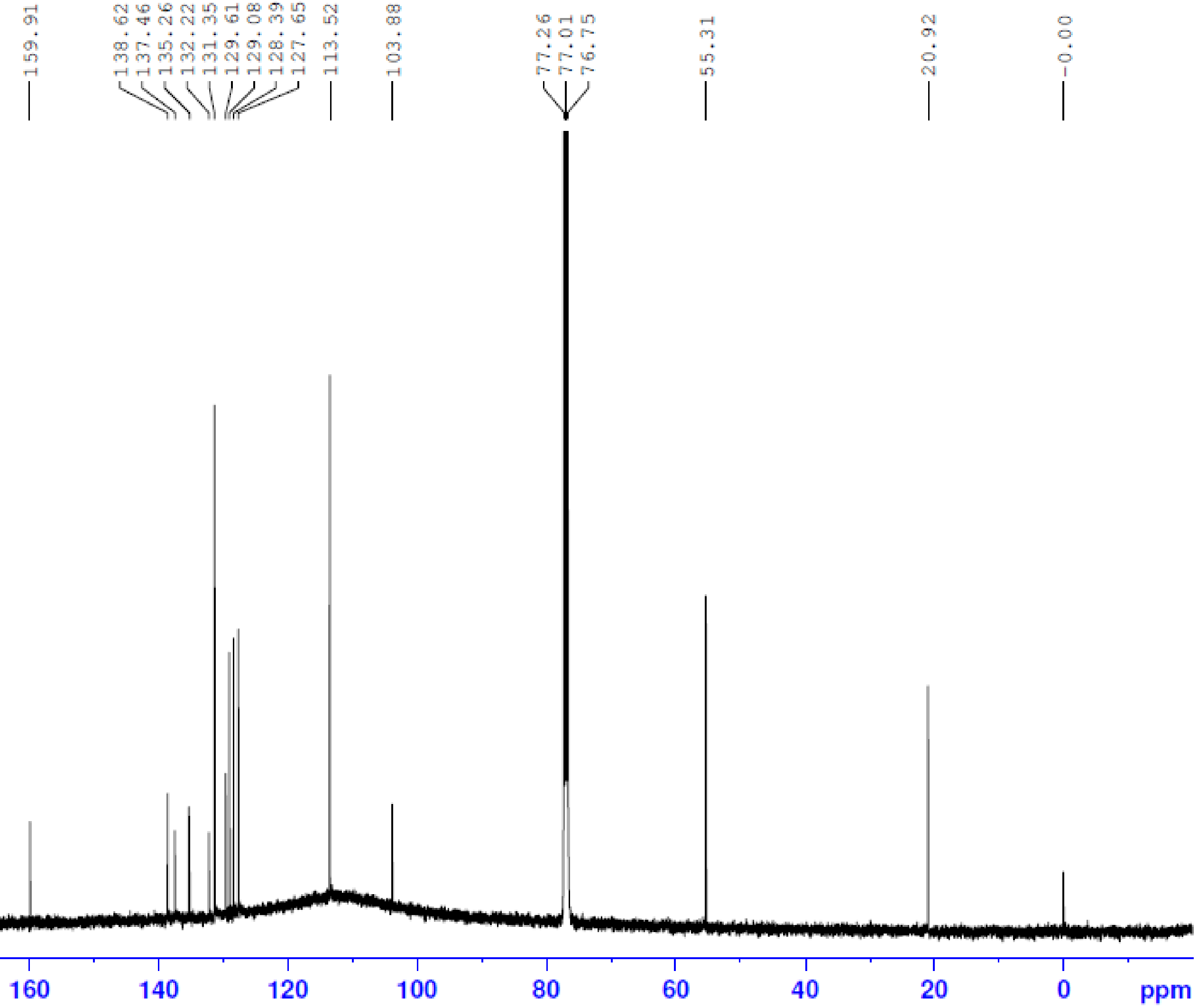

DL-238_F1F1_K gCOSY_CDCl3 + 0.03% TMS, 25 st. C

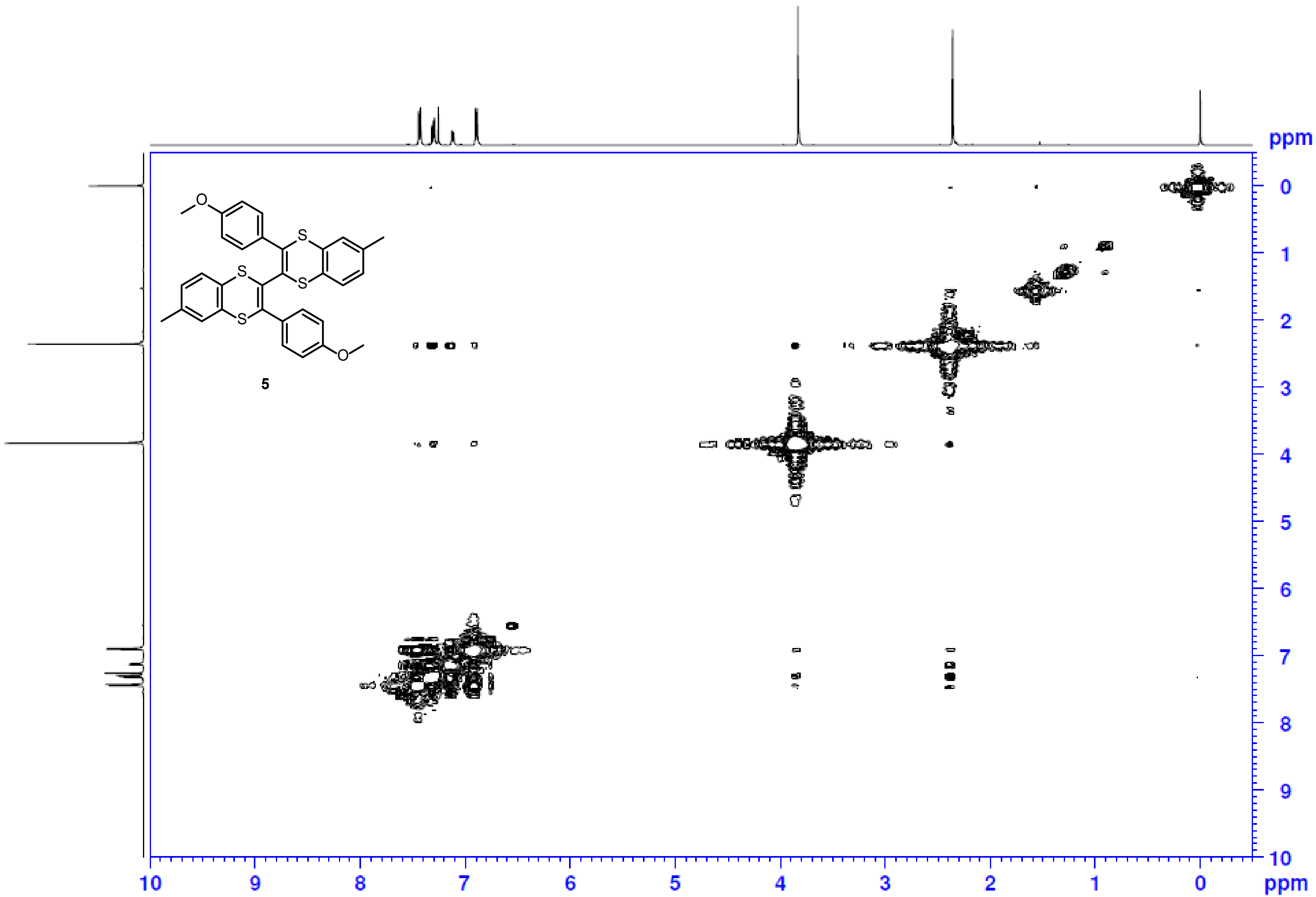

5

DL-238_F1F1_K gHSQC_CDCl3 + 0.03% TMS, 25 st. C

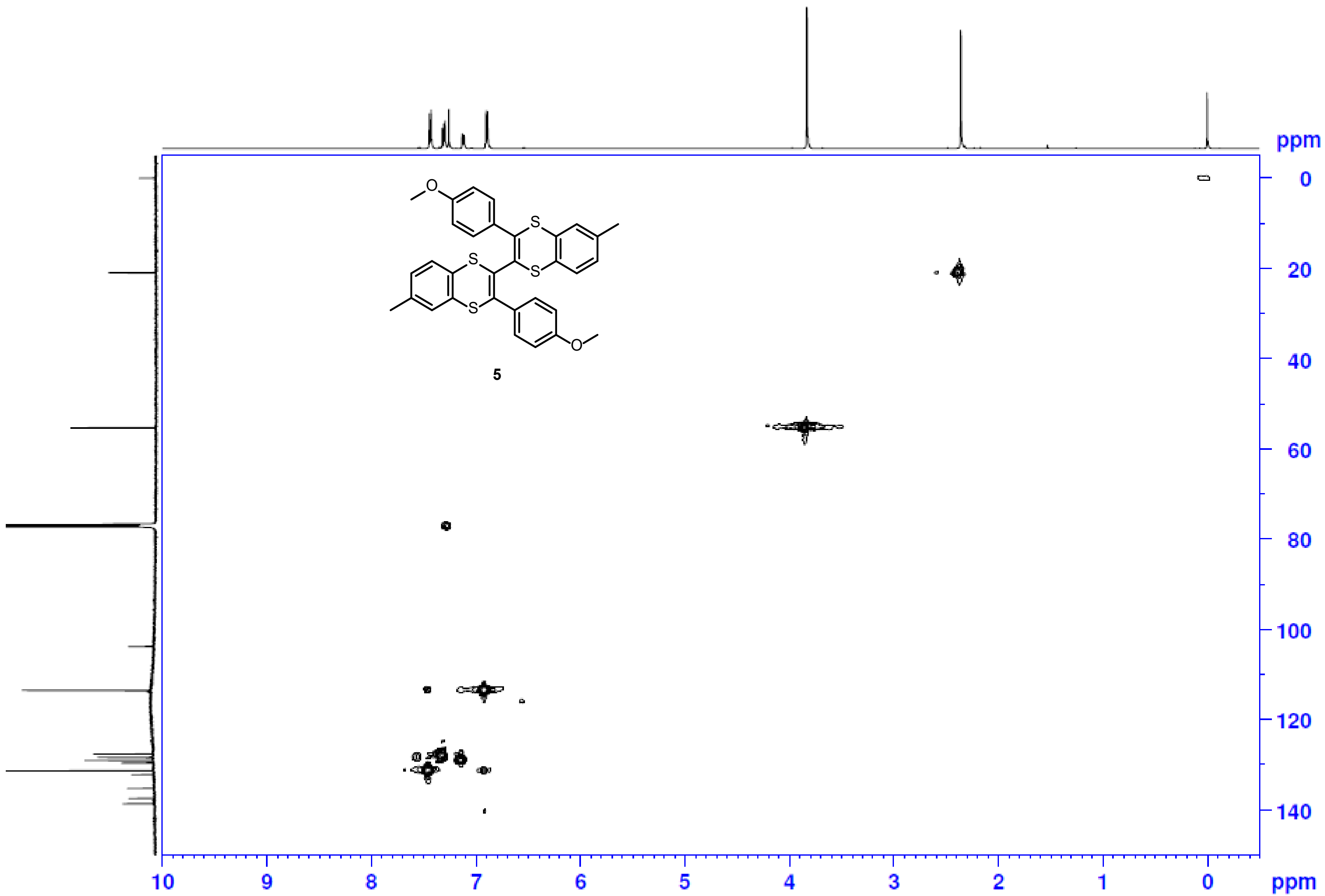

5

DL-238_F1F1_K gHMBC_CDCl3 + 0.03% TMS, 25 st. C

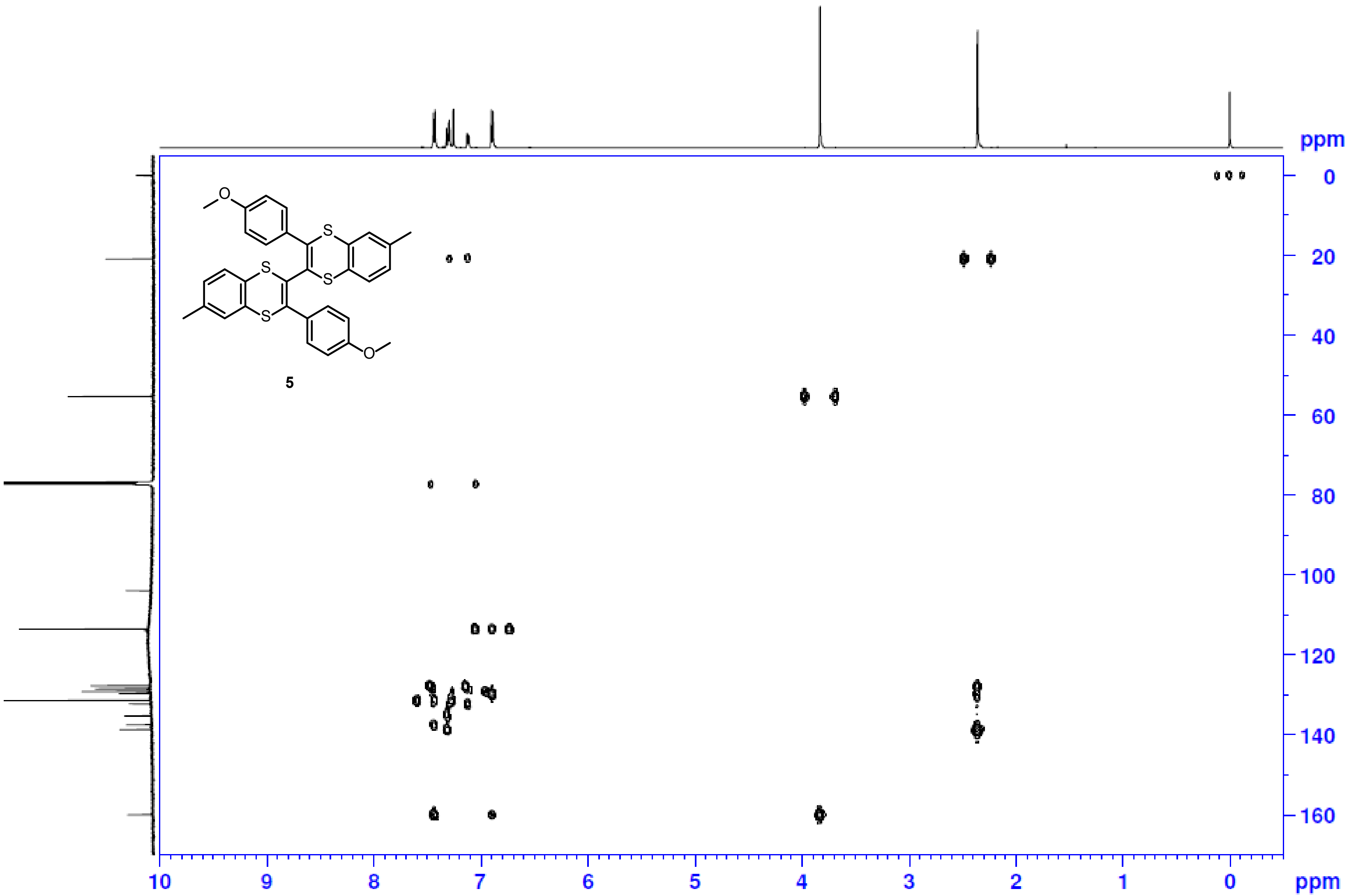

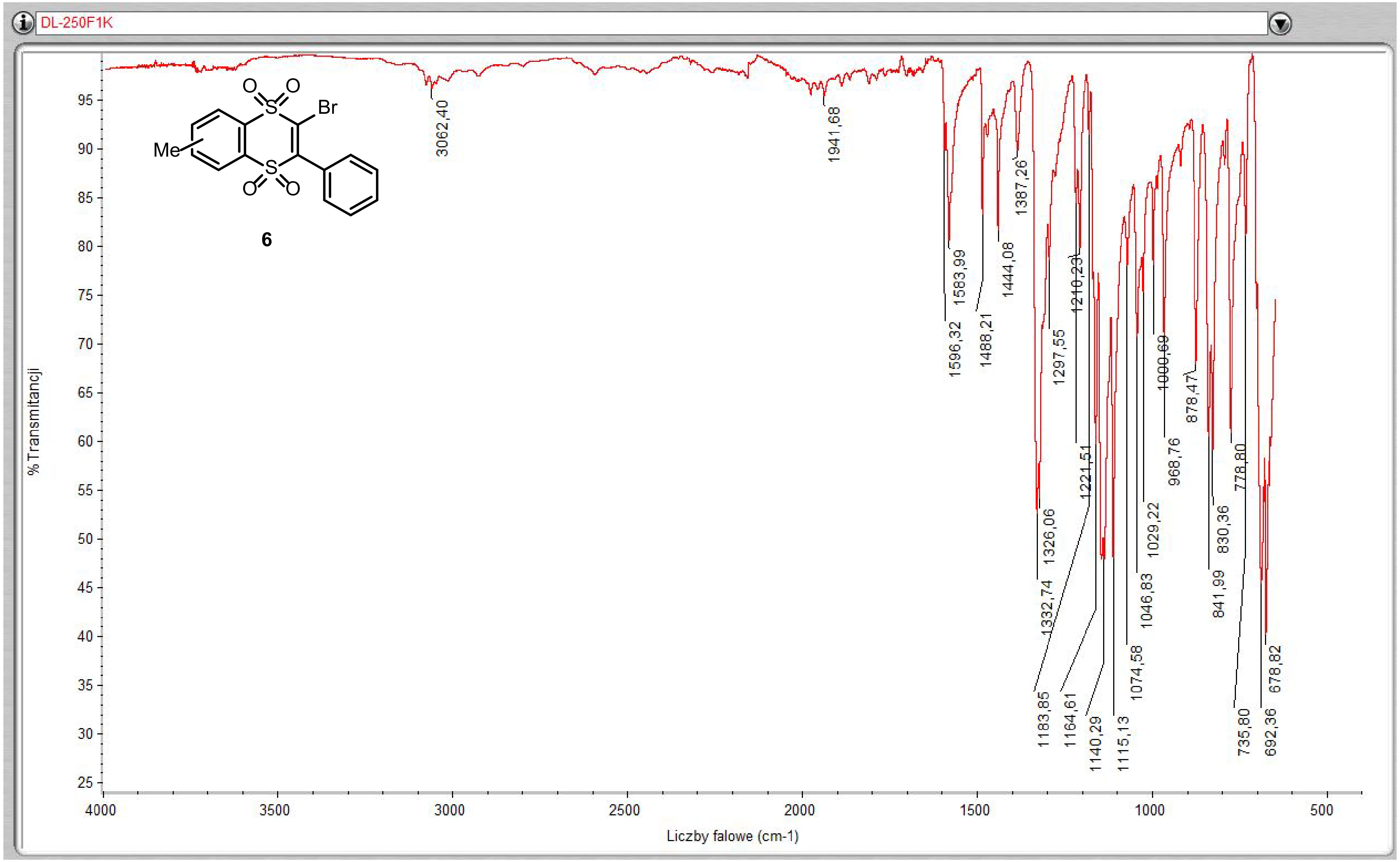

DL-250F1K
Br
Me
6
% Transmitancji
Liczby falowe (cm-1)
3062,40
1941,68
1596,32
1583,99
1488,21
1444,08
1387,26
1332,74
1326,06
1297,55
1221,51
1210,23
1183,85
1164,61
1140,29
1115,13
1074,58
1046,83
1029,22
1000,69
968,76
878,47
841,99
830,36
778,80
735,80
692,36
678,82

DL-250_F1 1H_CDCl3 + 0.03%, 25 st. C

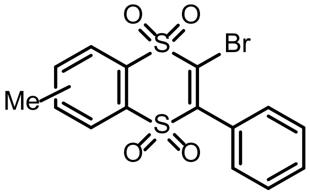

6

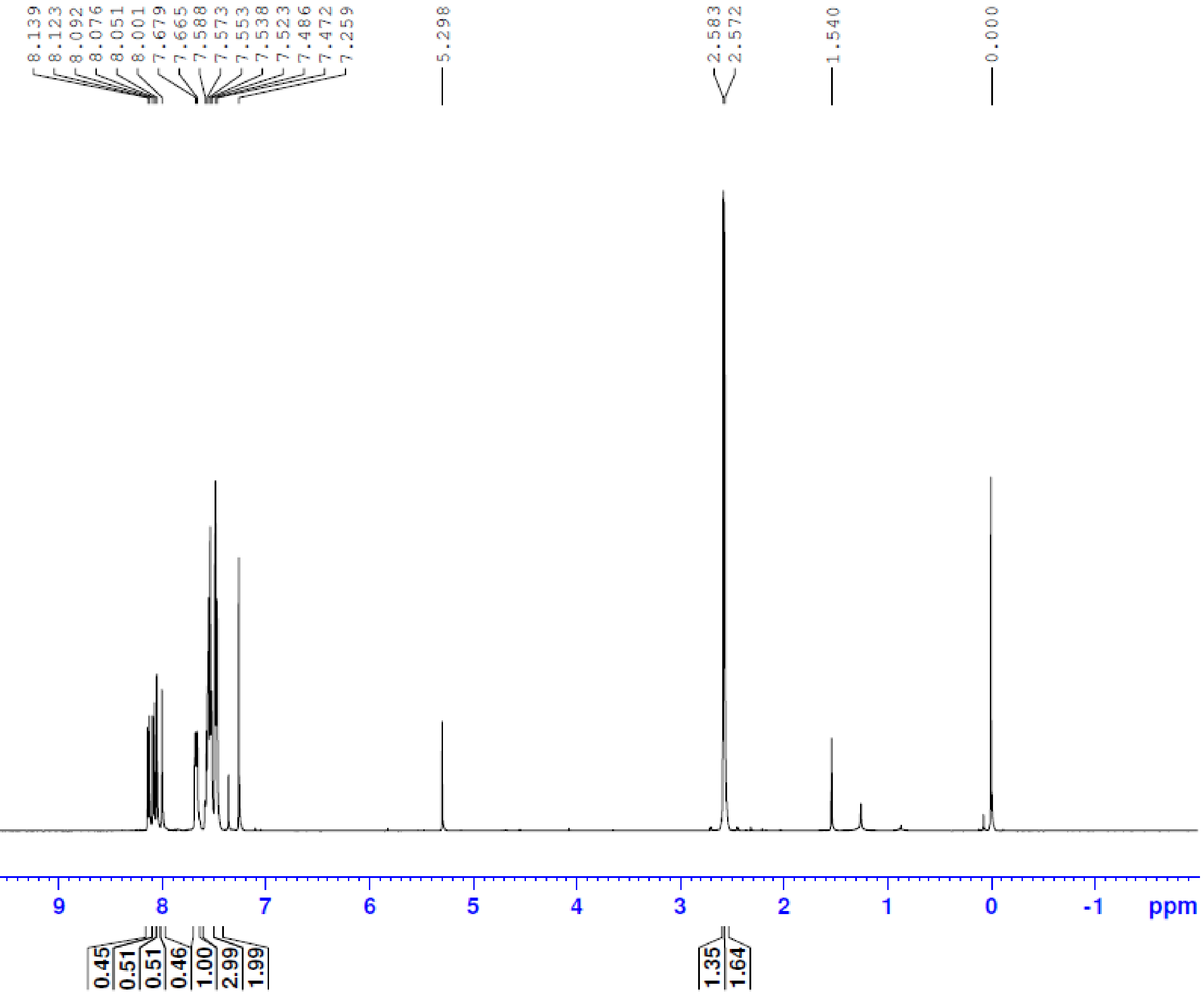

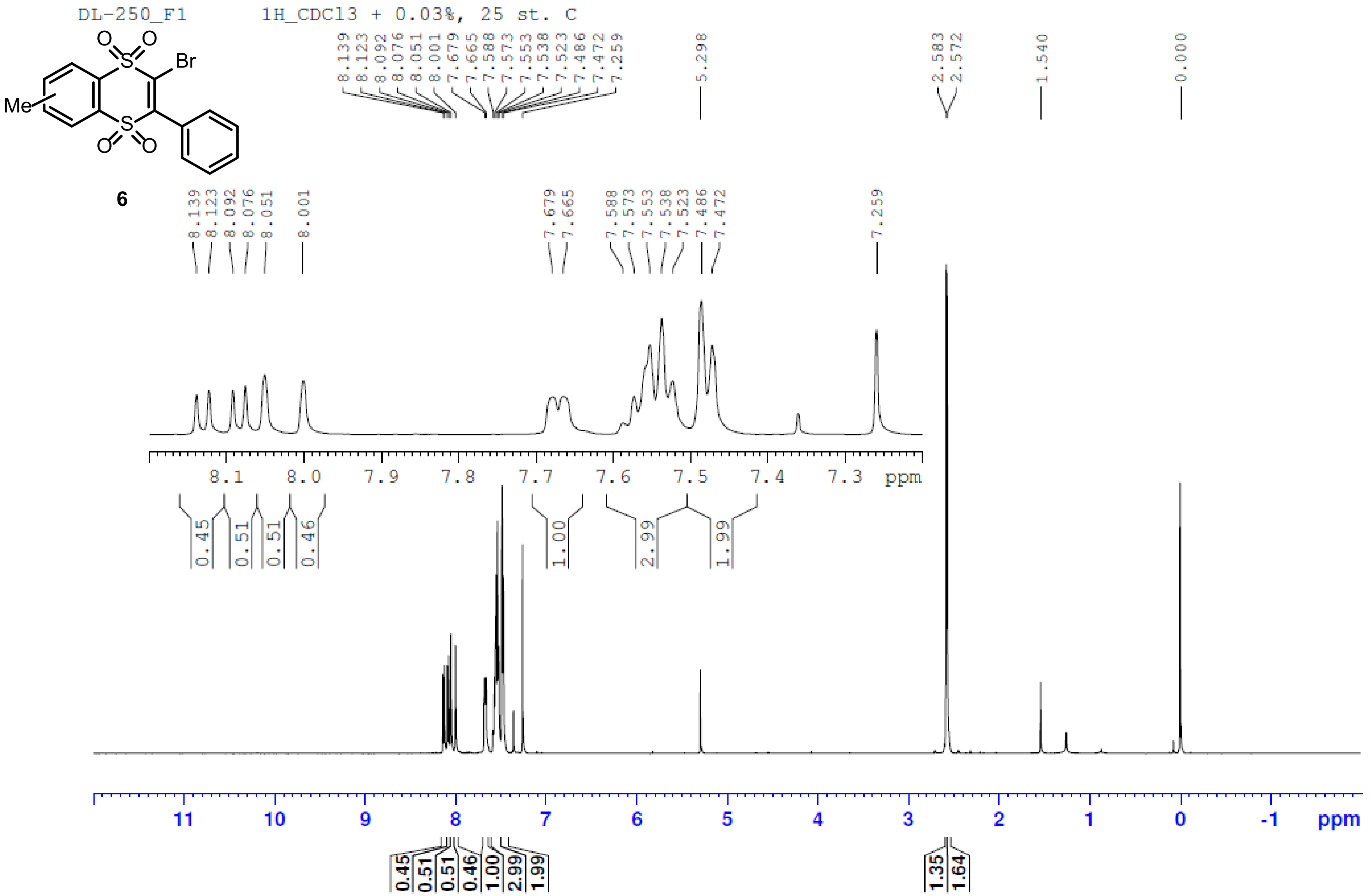
DL-250_F1
1H_CDCl3 + 0.03%, 25 st. C
Br
Me
6
8.139
8.123
8.092
8.076
8.051
8.001
7.679
7.665
7.588
7.573
7.553
7.538
7.523
7.486
7.472
7.259
5.298
2.583
2.572
1.540
0.000
0.45
0.51
0.51
0.46
1.00
2.99
1.99
1.35
1.64
ppm

DL-250_F1 13C_CDCl3 + 0.03% TMS, 25 st. C

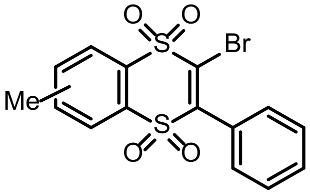

6

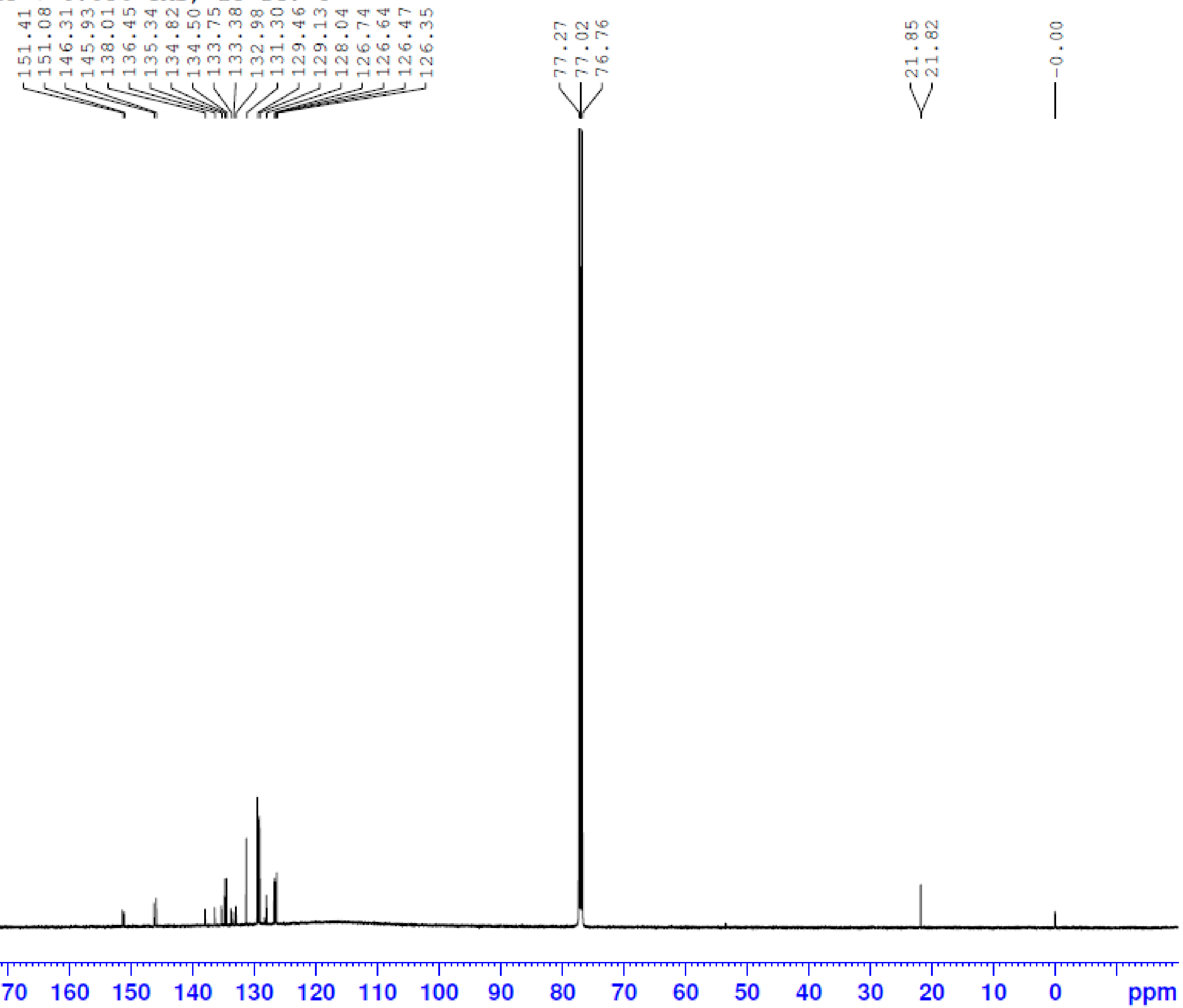

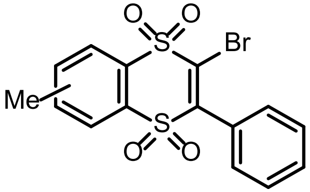
O
O
S
Br
Me
S
O
O

6

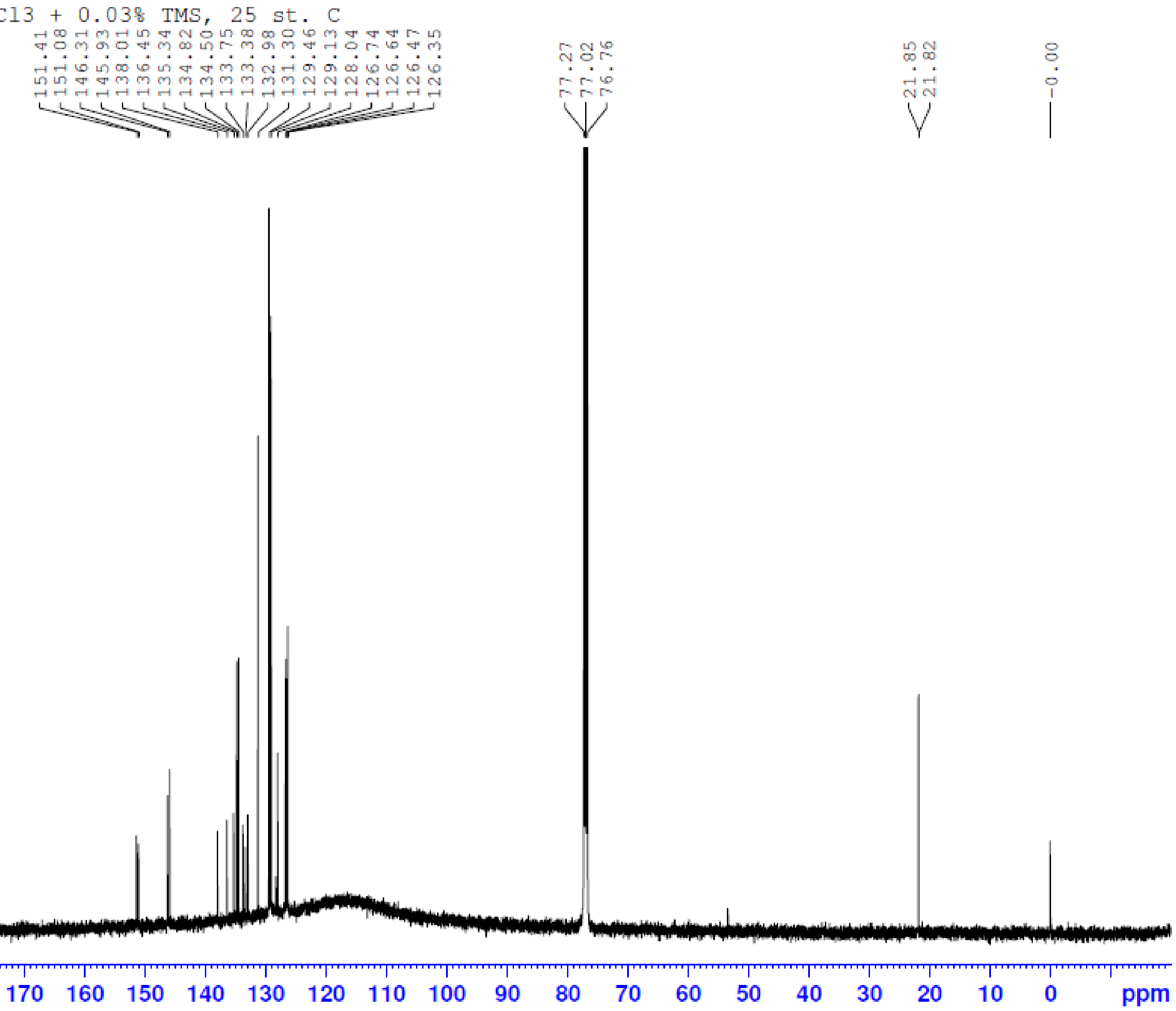
DL-250_F1 13C_CDCl3 + 0.03% TMS, 25 st. C
151.41
151.08
146.31
145.93
138.01
136.45
135.34
134.82
134.50
133.75
133.38
132.98
131.30
129.46
129.13
128.04
126.74
126.64
126.47
126.35
77.27
77.02
76.76
21.85
21.82
-0.00
210 200 190 180 170 160 150 140 130 120 110 100 90 80 70 60 50 40 30 20 10 0 ppm

DL-250_F1 13C_CDCl3 + 0.03% TMS, 25 st. C

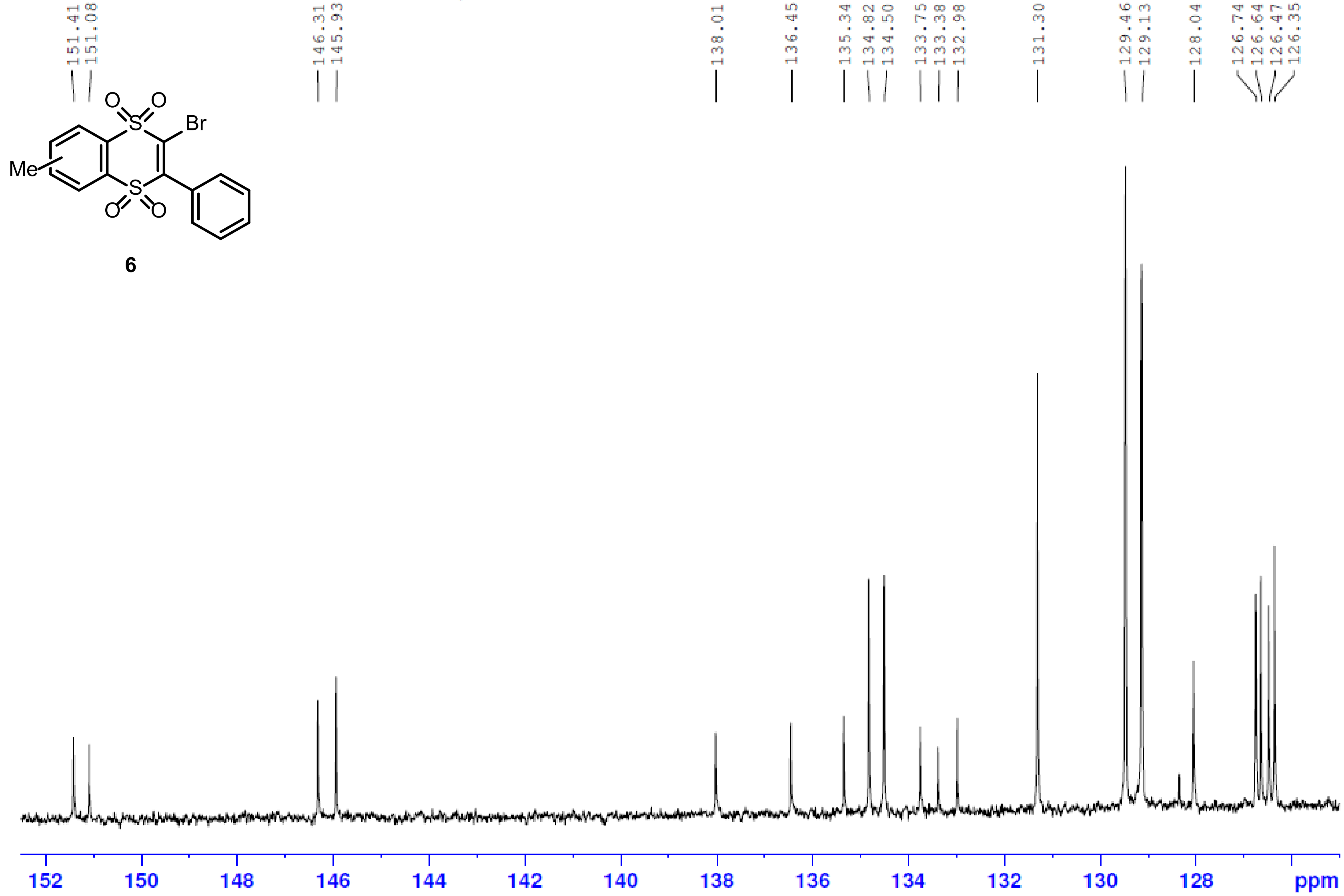

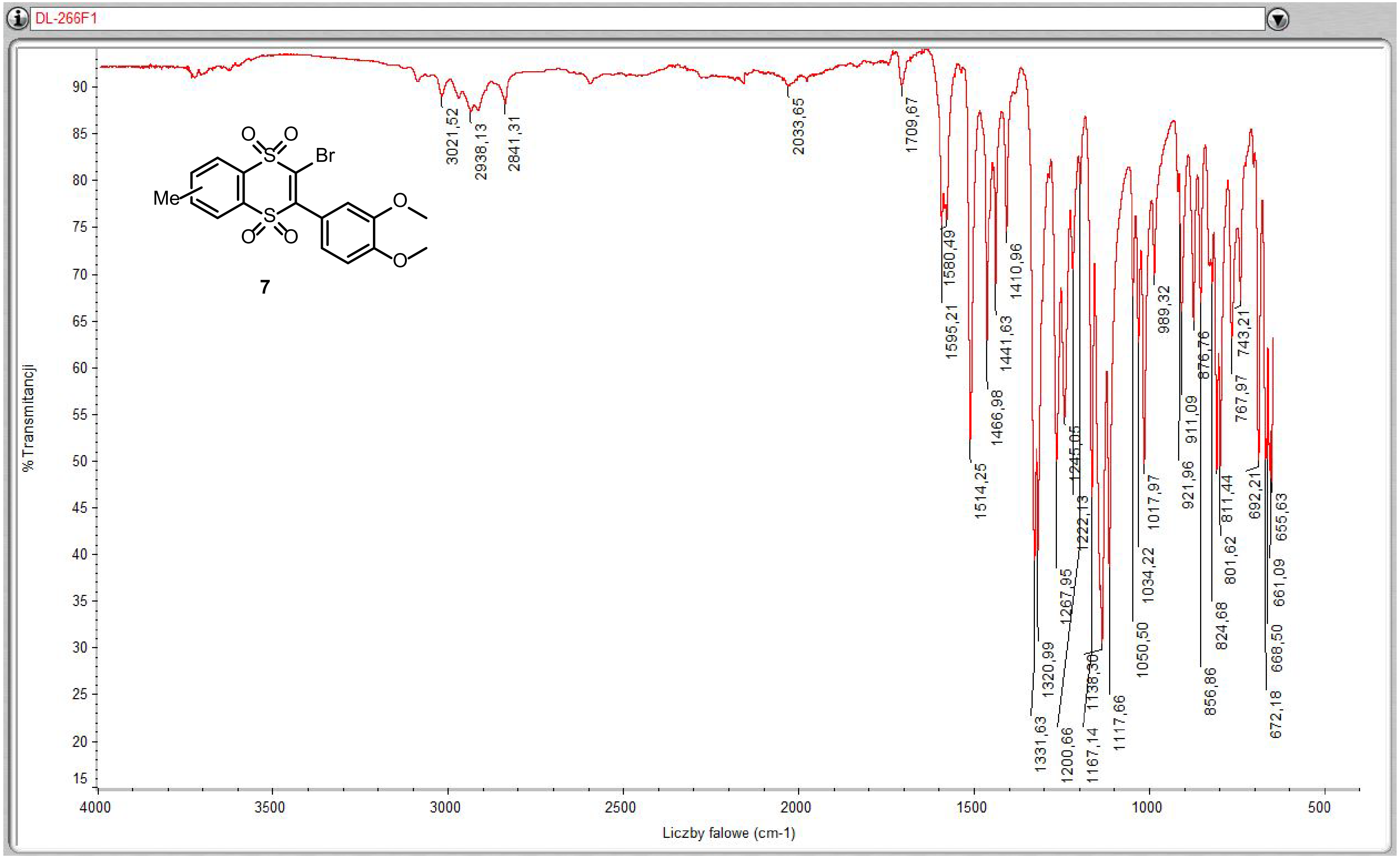

DL-266F1
% Transmitancji
Liczby falowe (cm-1)
Me
Br
O
S
7
3021,52
2938,13
2841,31
2033,65
1709,67
1580,49
1595,21
1514,25
1466,98
1441,63
1410,96
1331,63
1320,99
1267,95
1245,05
1222,13
1200,66
1167,14
1138,30
1117,66
1050,50
1034,22
1017,97
989,32
921,96
911,09
876,76
856,86
824,68
811,44
801,62
767,97
743,21
692,21
672,18
668,50
661,09
655,63

DL-266_B_F1   1H_CDCl3, 25 st. C

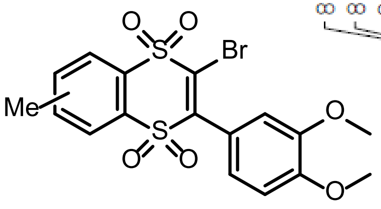

7

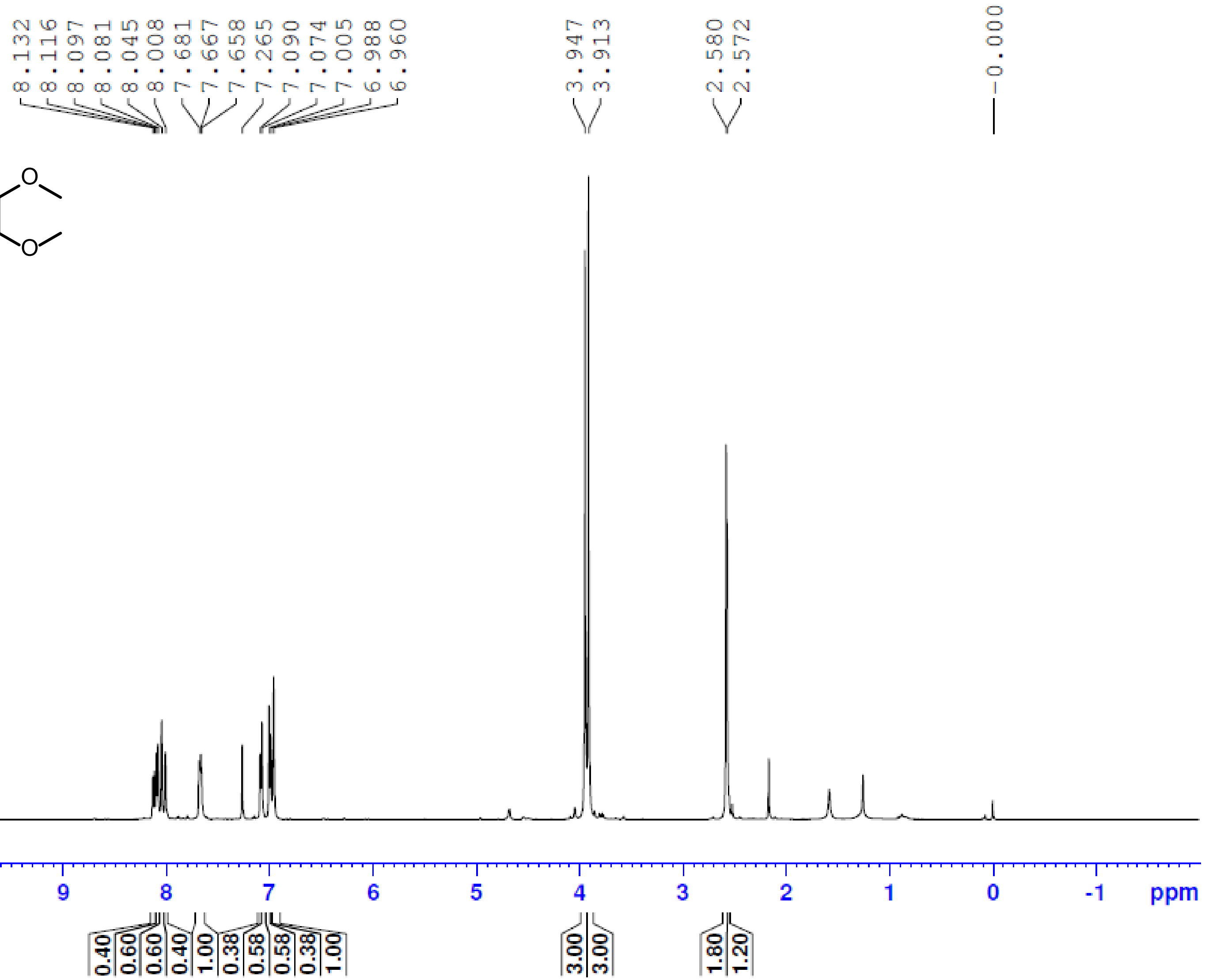

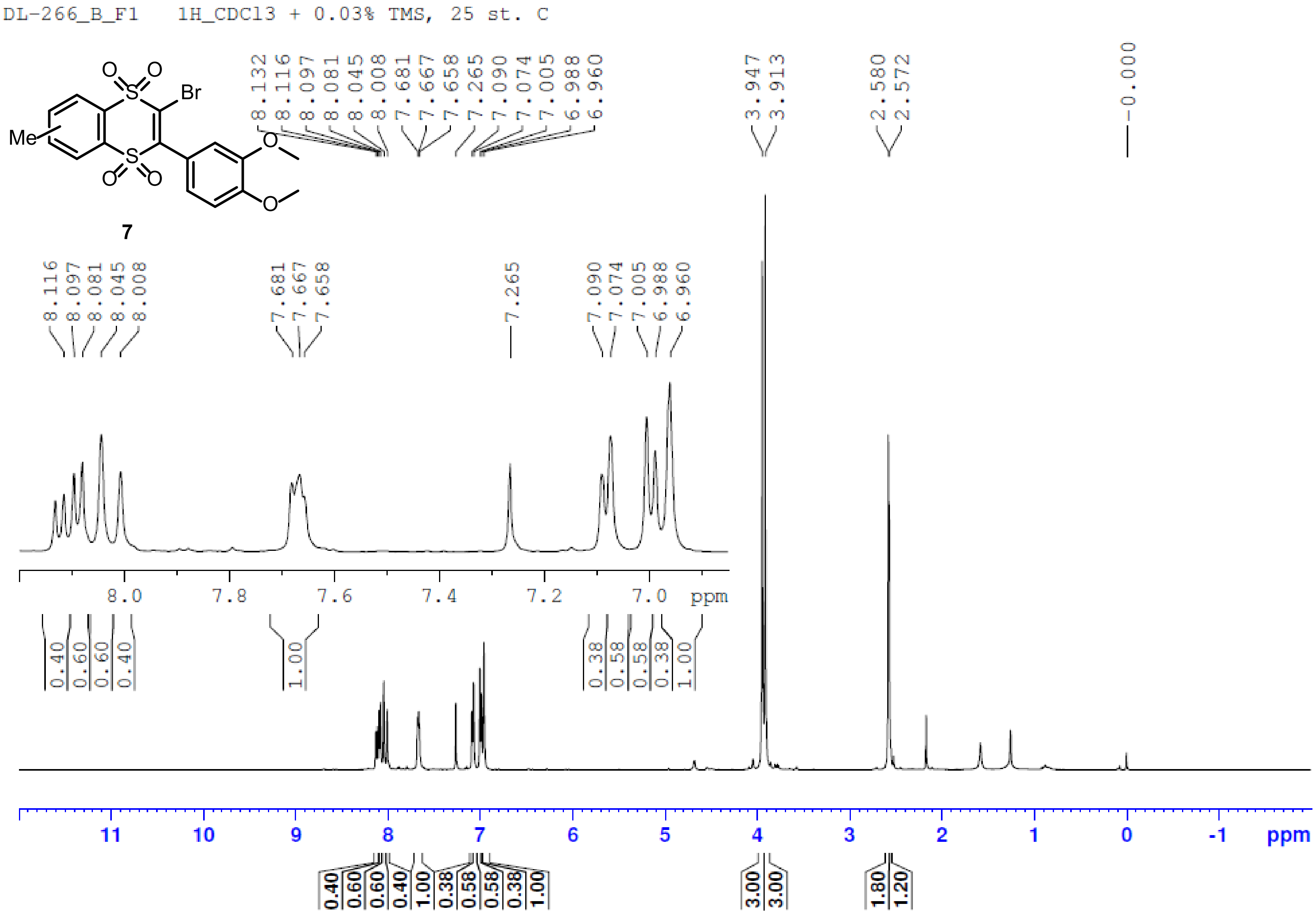
DL-266_B_F1 1H_CDCl3 + 0.03% TMS, 25 st. C
Br
Me
7
8.132 8.116 8.097 8.081 8.045 8.008 7.681 7.667 7.658 7.265 7.090 7.074 7.005 6.988 6.960
3.947 3.913
2.580 2.572
-0.000
ppm
0.40 0.60 0.60 0.40 1.00 0.38 0.58 0.58 0.38 1.00 3.00 3.00 1.80 1.20

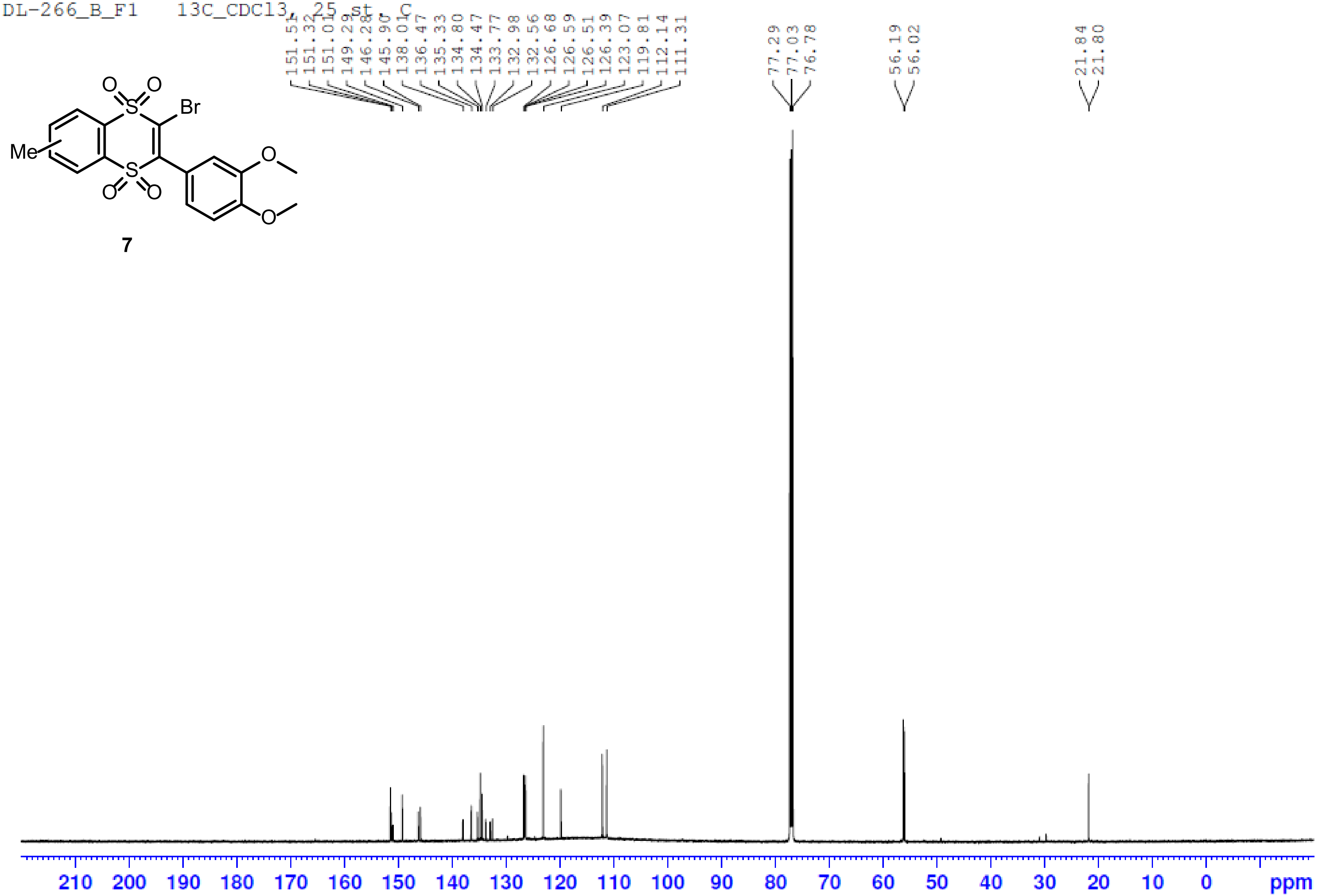
DL-266_B_F1 13C_CDCl3, 25 st, C
151.51
151.32
151.01
149.29
146.28
145.90
138.01
136.47
135.33
134.80
134.47
133.77
132.98
132.56
126.68
126.59
126.51
126.39
123.07
119.81
112.14
111.31
77.29
77.03
76.78
56.19
56.02
21.84
21.80
Br
Me
7
210 200 190 180 170 160 150 140 130 120 110 100 90 80 70 60 50 40 30 20 10 0 ppm

DL-266_B_F1 13C_CDCl3, 25 st. C

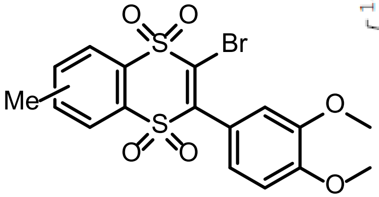

7

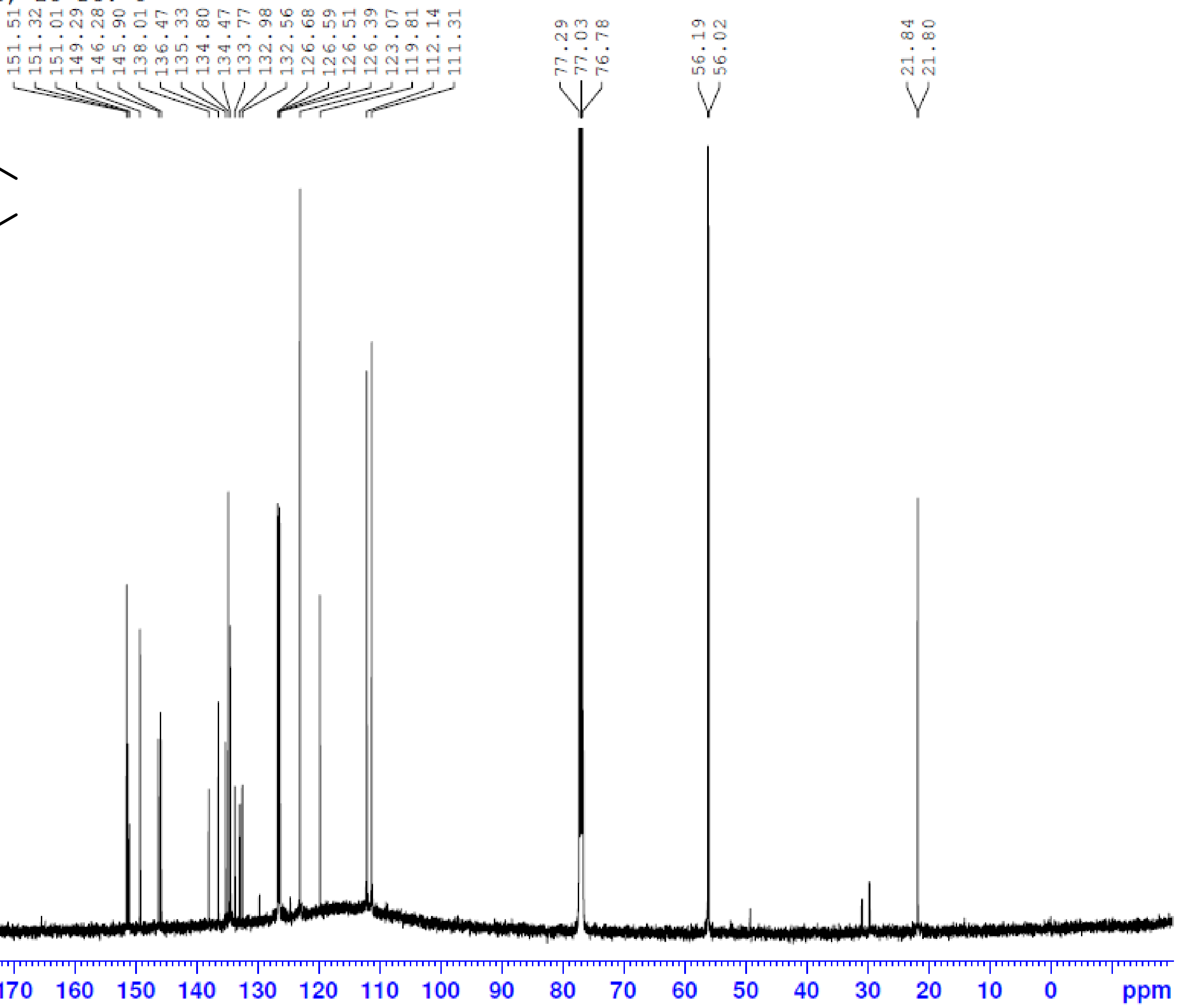

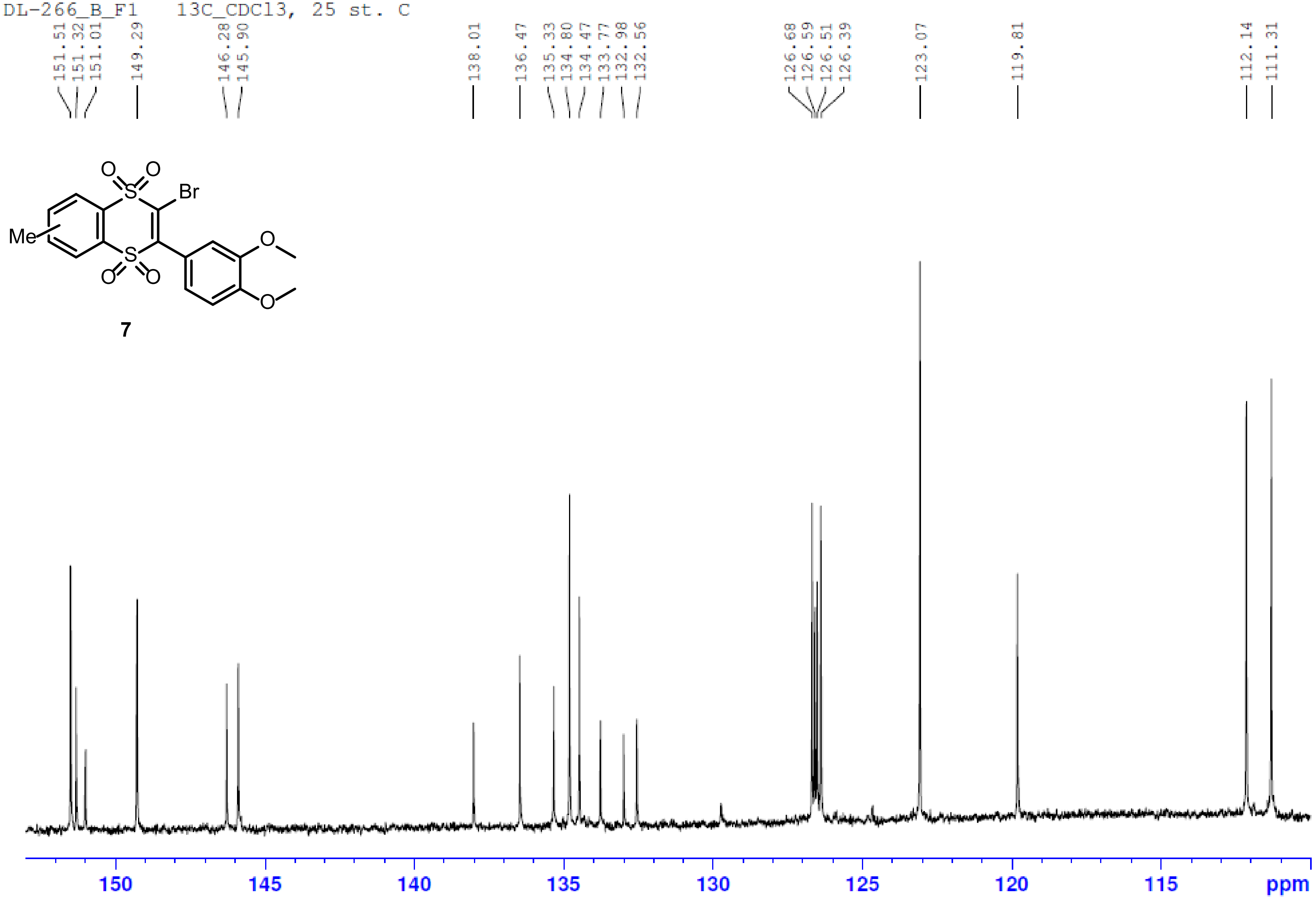
DL-266_B_F1 13C_CDCl3, 25 st. C
151.51
151.32
151.01
149.29
146.28
145.90
138.01
136.47
135.33
134.80
134.47
133.77
132.98
132.56
126.68
126.59
126.51
126.39
123.07
119.81
112.14
111.31
Br
Me
7
150
145
140
135
130
125
120
115
ppm
